# Silica Nanoparticle/Fluorescent Dye Assembly Capable of Ultrasensitively Detecting Airborne Triacetone Triperoxide: Proof-of-Concept Detection of Improvised Explosive Devices in the Workroom

**DOI:** 10.1021/acsami.3c05931

**Published:** 2023-06-21

**Authors:** Andrea Revilla-Cuesta, Irene Abajo-Cuadrado, María Medrano, Mateo M. Salgado, Manuel Avella, María Teresa Rodríguez, José García-Calvo, Tomás Torroba

**Affiliations:** † Department of Chemistry, Faculty of Science, University of Burgos, 09001 Burgos, Spain; ‡ Electron Microscopy Lab, IMDEA Materials Institute, Eric Kandel, 2, Tecnogetafe, 28906 Getafe, Madrid, Spain

**Keywords:** triacetone triperoxide, improvised explosive devices, chemical sensors, fluorescent materials, vapor phase detection, aggregation-induced emission materials

## Abstract

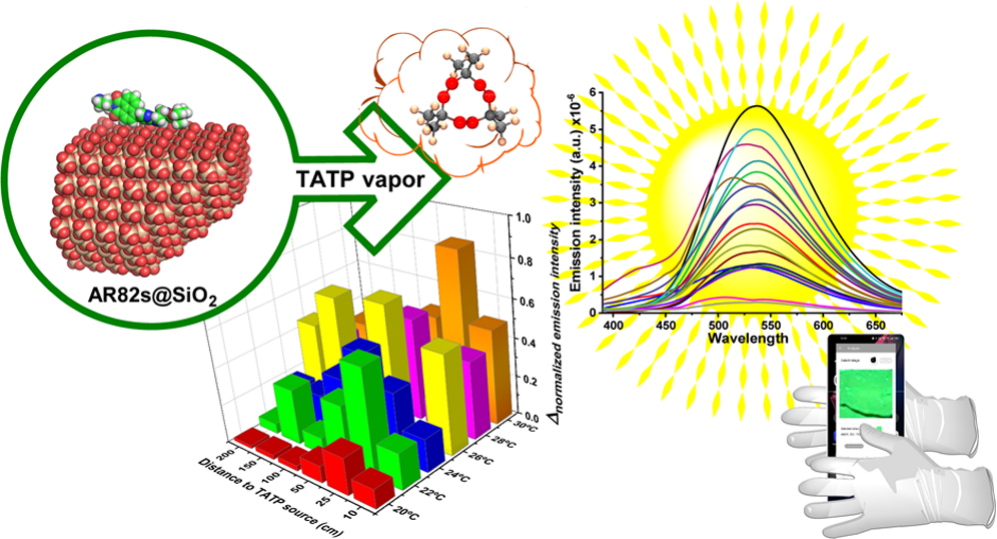

We describe the proof of concept of a portable testing setup for the detection of triacetone triperoxide (TATP), a common component in improvised explosive devices. The system allows for field testing and generation of real-time results to test for TATP vapor traces in air by simply using circulation of the air samples through the sensing mechanism under the air conditioning system of an ordinary room. In this way, the controlled trapping of the analyte in the chemical sensor gives reliable results at extremely low concentrations of TATP in air under real-life conditions, suitable for daily use in luggage storage for airlines or a locker room for a major sporting event. The reported fluorescent methodology is very sensitive and selective, allowing for the trapping of triacetone triperoxide in the chemical sensor to give reliable results at very low concentrations in air under ambient conditions, by comparing the fluorescence of the material before and after exposition to TATP traces in air.

## Introduction

A major threat in modern society is constituted by the so-called improvised explosive devices (IEDs).^1−6^ The explosives contained in IEDs were commonly used in war scenarios but some years ago they were also used in situations of everyday life, therefore constituting a threat to the lives of countless people. Methods for the quick detection of explosive devices are needed before they cause damage, but there are very few methods for the detection of some explosives used in IEDs.^7−11^ Indeed, the most advanced spectroscopy devices are very sensitive and selective to identify the chemicals used in IEDs, but they are typically too heavy for portable devices and too much expensive for widespread use.^12^ The cheap technology of the cotton swab^13^ requires physical access to the substance that might be hidden or not easily accessible. There is a long tradition of the use of animals in the detection of explosives, particularly trained dogs,^14^ but some explosives without a characteristic smell go undetected by animals. Electronic noses, made up of artificial sensor arrays, constitute a significant solution to replace the natural senses^15,16^ for reliable monitorization of diverse environments. Appropriate portable devices should alert the presence of explosives within minutes. Triacetone triperoxide (TATP) is synthesized directly from readily available starting materials;^17^ for this reason, it has been frequently used in the preparation of IEDs. TATP sublimes easily at room temperature, but, because of the lack of aromatic nitro groups, its presence in the vapor phase is not detected by using common methods for explosives.^18^ Common explosives containing nitro groups, such as trinitrotoluene (TNT), have a large variety of fluorescent methods for their detection, including our contribution to the field^19^ and from other groups.^20−23^ Another explosive employed in IEDs which is also important, hexamethylene triperoxide diamine (HMTD), has a much lower vapor pressure than TATP. Unfortunately, the detection of HMTD in the vapor phase is not possible.^24^ For the detection of TATP, the most common methods are mass spectrometry,^25^ ion mobility spectrometry or related technologies,^26−28^ and multiphoton spectroscopy.^29^ Optional complementary methods are chemically modified nanosensor arrays,^30^ or portable optical methods based on colorimetric sensor arrays, in this case to detect the hydrogen peroxide (H_2_O_2_) produced by decomposition of the TATP.^31−33^ Alternative indirect detection methods, by detection of H_2_O_2_ from TATP, and the oxidative processes linked to them, are frequently used for fluorometric sensing,^34−46^ as well as detection of acetone from decomposition of TATP.^37,38^ The direct detection of TATP has also been achieved by fluorescence quenching.^39^ Analytical methods for the detection of TATP (or HMTD) in different scenarios must be selective for peroxides, appropriate for immediate analysis, and safe sampling by a qualified operator.^40^ They are expected to have very low limits of detection and high selectivity, in this way, they can detect peroxides in confined public places to prevent explosions and further damage.^41^ An extremely low limit of detection is easier to achieve with the highly volatile TATP than with the much less volatile HMTD.^42^ Most physicochemical analytical methods for peroxide-based explosives are designed in this way.^43,44^ Trace detection of TATP in the vapor phase by colorimetric or fluorometric methods, taking into account its volatility, continues to be an attractive approach for the development of chemical sensors for peroxide explosives. Some characteristics such as sensitivity and selectivity are still not well addressed. Our previous approaches consisted of solid fluorogenic sensors for the sensitive and selective detection of pristine TATP^45,46^ or the microwave detection of wet-TATP,^47^ both in the vapor phase, and the detection of TATP gas in an air microfluidic device.^48^ After that, we have been looking for more selective and sensitive materials specifically tailored for the unambiguous detection of TATP traces in air.^49^ For that purpose, we searched among solid-supported fluorescent dyes in which an oxidation event could modulate the aggregation-induced emission (AIE) characteristics of the dyes for the detection of oxidizing species. Naphthalimides are known to have AIE effects.^50^ We selected a collection of new naphthalimide structures with donor and accepting groups in the structure and amino-protecting substituents in which the variation of substituents permitted the control of the AIE characteristics; in this way, we arrived at a new naphthalimide dye supported on the surface of silica nanoparticles with optimum features for the selective and ultrasensitive detection of traces of TATP in air. In this material, the AIE-gen fluorescent emission mechanism could be triggered with optimum performance via oxidation by only hydrophobic oxygen-containing oxidants. The system worked within a typical office room by simply placing the sensor in the room’s air stream. In this way, the material functioned as an IED-directed chemical tracking system. The results of the study are presented here.

## Results and Discussion

### Preparation of Materials

**Figure 1:**
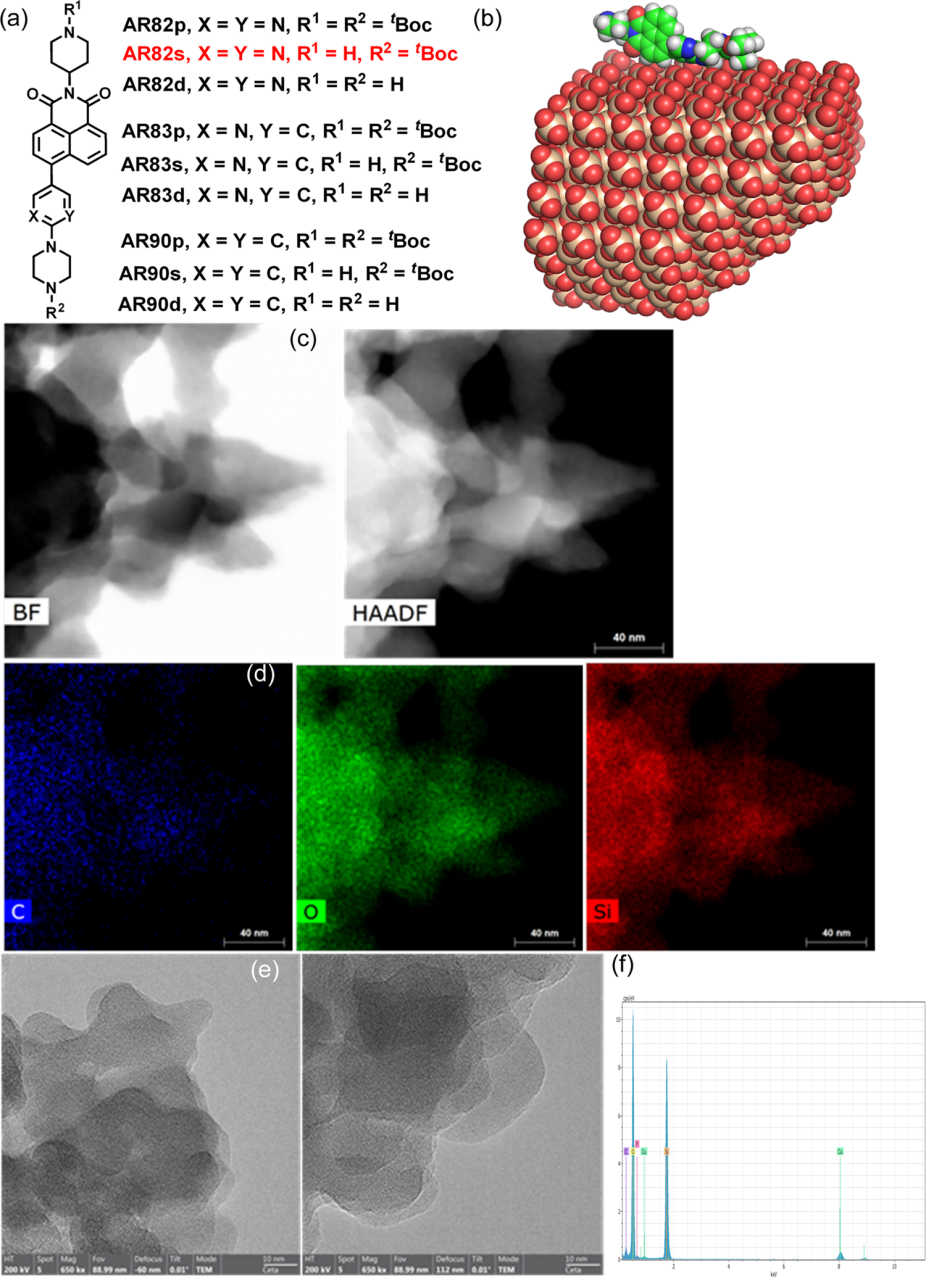
(a) Structure of chemical probes used for the study. (b) Model of the chemical probe AR82s on silica. (c, e) Structural and (d) elemental TEM images of the AR82s@SiO_2_ nanoparticles. (f) Elemental composition of the AR82s@SiO_2_ nanoparticles and high-resolution TEM images.

We synthesized a number of fluorogenic probes that were tested for several types of analytes. The synthesis was performed by using Suzuki reactions, following previous experiences in developing fluorogenic probes by carbon–carbon coupling chemistry.^51−59^ The new fluorogenic probes are shown in Figure 1. The amino-protected naphthalimides showed an increase of fluorescence in mixtures of organic solvents with increasing amounts of water (AIE) that disappeared when the structures contained unprotected amino groups. Previous examples have shown some of these characteristics.^60,61^ The selectivity of each fluorogenic probe against the most important oxygen-based oxidizing reagents was addressed in organic–aqueous solvents, with the aim to select the best candidates for an array of fluorogenic probes for oxygen-based explosives. With the new fluorogenic probes, we performed a complete characterization and a battery of preliminary tests about selectivity and sensitivity with target explosives and several expected interferents. The sensitivity of the collection of fluorogenic probes was checked in the presence of TATP and HMTD. We prepared an experimental design for this part of the work that was applied to all fluorescent probes. Then, the sensitivity of solid-supported naphthalimides was studied in the presence of gaseous traces of home-made explosive triacetone triperoxide, so the fluorogenic materials were capable of discriminate the presence and the nature of the most important oxygen-based home-made explosive, TATP. Having a previous experience in modified materials,^45,46,48,55,62−64^ we performed initial experiments by supporting the fluorescent probes on poly(dimethylsiloxane) (PDMS), but the detection limits were not good enough. Instead, silica nanoparticles afforded much better results. Next, the best fluorogenic probes developed in the previous sections were studied by quantitative sensing experiments to measure the performance of the best probes in terms of sensitivity and selectivity to trace amounts of TATP. Probes AR82s and AR82d, from the first naphthalimide series (Figure 1), both bearing the same structure and different number of protective groups in the periphery, resulted sufficiently sensitive to the presence of TATP. Furthermore, we checked the stability of both probes and found that AR82s were much more stable than AR82d; therefore, we used only AR82s for the preparation of the sensing material. AR82s-supported silica nanoparticles (AR82s@SiO_2_) were prepared by stirring under nitrogen, in the dark, 1 mg of AR82s and 100 mg of silica nanopowder, 10–20 nm particle size, in 5 mL chloroform for 30 min until there was no trace of dye in the solvent, then the solvent was evaporated, the solid was washed for three times with hexane (3 mL each), and then subjected to centrifugation and drying under a nitrogen stream to get the material (Figure 1). The unique sensitivity manifested by the AR82s/d series was not attained by the other two series of compounds (AR83s/d and AR90s/d), despite the large number of tests performed for all of the synthesized compounds under study (see the Supporting Information).

### Titration under Increasing Concentration of TATP Using a Recirculation System for TATP Vapors

**Figure 2:**
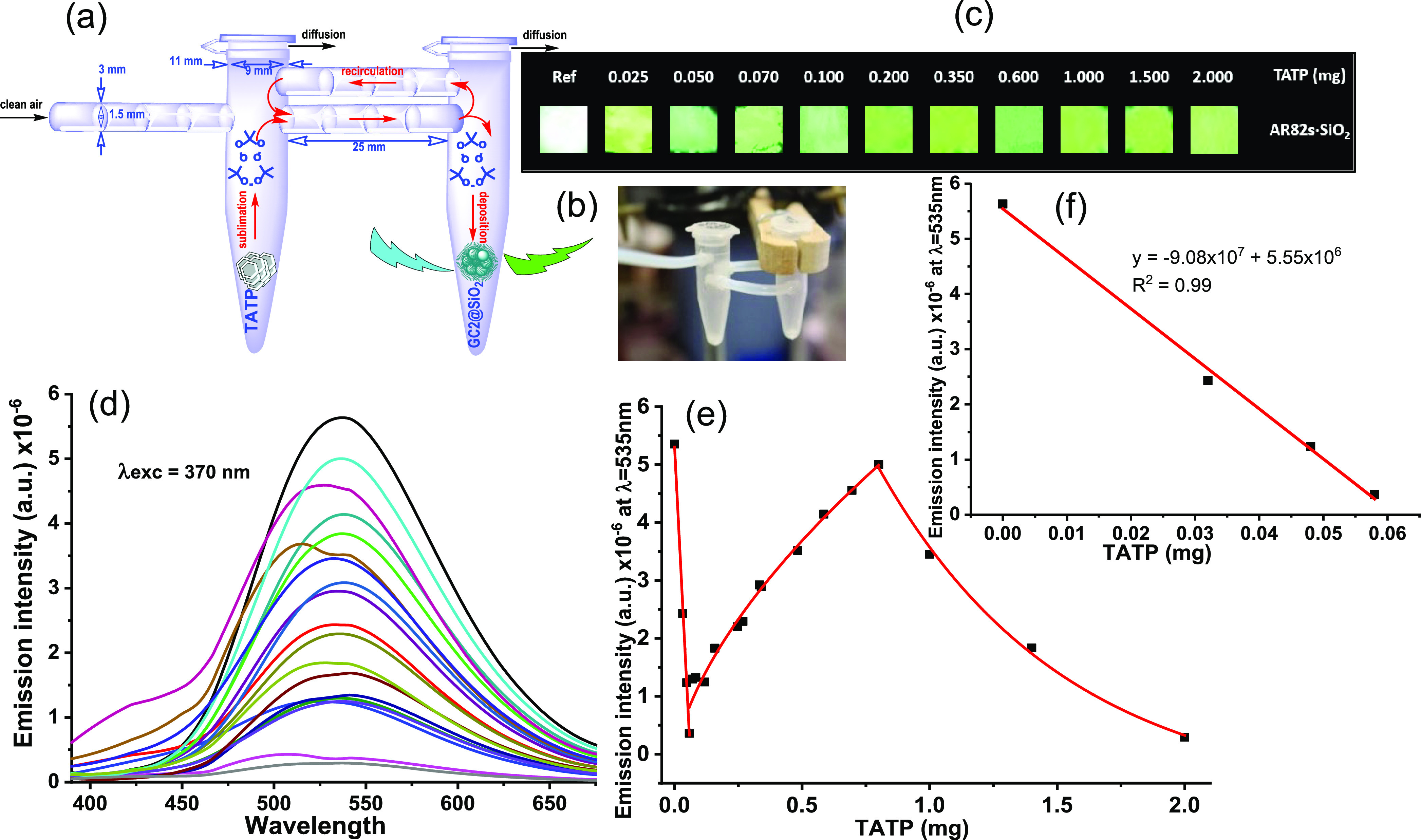
(a) Microfluidic device dimensions: Eppendorf: outer top diameter: 11 mm, inner top diameter: 9 mm, outer bottom diameter: 5 mm, inner bottom diameter: 3 mm, volume: 1.5 mL, height (with lid): 40 mm, height (without lid): 38 mm; tubing: outer diameter: 3 mm, inner diameter: 1.5 mm, length (flow tube): 25 mm, length (return tube:): 35 mm. (b) The actual aspect of the system; reproduced from ref (48) with permission from the Chinese Chemical Society (CCS), Institute of Chemistry of Chinese Academy of Sciences (IC), and the Royal Society of Chemistry. (c) Fluorescence changes with TATP gas. (d) Titration curves of AR82s@SiO_2_ and TATP vapor. (e) Fluorescent profile at 535 nm and (f) calibration for the limit of detection of AR82s@SiO_2_ nanoparticles under increasing concentrations of TATP vapor. λ_exc_ = 370 nm, λ_em_ = 535 nm.

The titration of AR82s@SiO_2_ nanoparticles was carried out by placing 15 mg of nanoparticles in an Eppendorf, covered with tin foil to avoid degradation of the nanoparticles by the action of light, and increasing amounts of TATP (from 0.025 to 2 mg) in another Eppendorf (Figure 2). Both were connected to each other and to an air stream provided by a compressor, and the microfluidic system maintained under airflow, 100 cm^3^/min, was gently warmed below 50 °C until consumption of TATP in every case. The lowest amounts of TATP took only seconds to evaporate (plus 10 min for recirculation), and the highest amounts took up to 20 min; therefore, the experiments were performed within 10 and 30 min until consumption of TATP. Then, the sensor material was subjected to fluorescence measurements in every case in an Edinburg Instrument FLS-980 fluorometer at an excitation wavelength of 370 nm at 25 °C. The results are displayed in Figure 2 as qualitative and quantitative fluorescent emission measurements. The titration plot showed an initial decrease of fluorescence in the presence of very low amounts of TATP, then an increase in fluorescence at low concentrations of TATP, and a decrease at higher concentrations. We calculated the limit of detection, LOD, from the initial titration plot values by IUPAC-consistent methods.^65^ In this case, we calculated the LOD within the values measured (different than 0) by adjusting the initial values to a mean square linear regression and using the R program.^66,67^ In this way, linear regression at low concentrations of TATP led to a LOD = 13.03 ng of TATP in absolute value or, taking into account the time of measurement and air efflux, LOD = 13.3 ng/L (57 pM). Assuming that the best LOD reported values are found in the ppb–ppm range for TATP sensing in the gas phase,^68^ this new method competes favorably in terms of sensitivity and simplicity with the best-known methods, with a LOD value of 1.4 ppb in air, the lowest found so far by artificial olfaction.^8^

### Validation in Real-Life Scenarios

**Figure 3:**
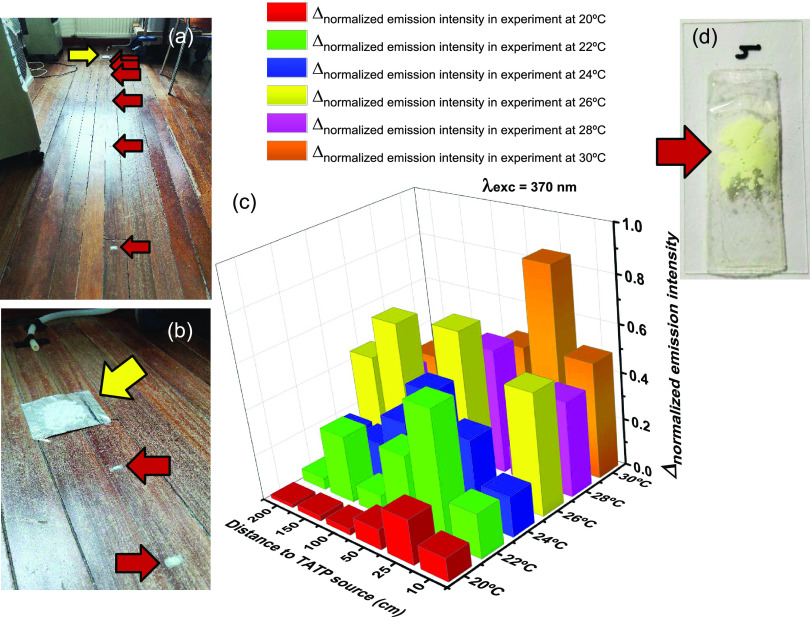
(a) Distribution of TATP sample (yellow arrow) and sensor samples (red arrows) in the room. (b) Close view of the arrangement of the TATP sample (yellow arrow) in front of the airflow outlet and the sensing material samples (red arrows). (c) Histogram plotting the differences in intensity emission in all cases before and after exposure to TATP vapors as a function of distance from the TATP source and temperature of the room, normalized to the top and reference signals. (d) A sample of sensing material showing the modified nanoparticles.

We looked for the validation of the fluorescent device to detect TATP explosive in real-life scenarios. We tested under close to realistic conditions the TATP sensor material by placing in a small room, at different temperatures, a few milligrams of solid TATP and some samples of the AR82s@SiO_2_ nanoparticles at different distances, leaving it for some time. In every case, we previously performed the experiment without TATP to have a set of comparison values. In all tests, there was a neat change of the fluorescence between the signal obtained with the nanomaterial in the presence of TATP at a certain distance, in comparison to the nanomaterial without the presence of TATP under the same ambient conditions. Subsequently, we looked for tests performed under the mildest conditions of the system to have a positive signal, under typical ambient temperatures and at increasingly longer distances from the TATP vapor source. The experiments were carried out in an office with dimensions 3.07 × 3.54 × 2.15 m^3^ (height × length × width). Inside the room, we placed appropriate air heaters to maintain constant temperature and an air compressor, which generated a gentle stream of clean air that passed over the TATP sample in the direction of the locations where the nanoparticles were positioned (Figure 3). The office had also its own central heating and ventilation system, so the airflow was not steady but subjected to the inherent small turbulences of every working office. A fixed amount of TATP (25 mg) was placed at a distance of 30 cm from the source of the airflow (100 cm^3^/min, 0.8 cm internal diameter) (Figure 3). A fixed amount of AR82s@SiO_2_ nanoparticles (0.75 mg) was placed on the surface of a cover glass, adhered to it with the help of a small amount of adhesive tape (Figure 3). This process was repeated 6 times, and the cover glasses were placed at increasing distances from the point where the TATP was located (10, 25, 50, 100, 150, and 200 cm) (Figure 3). We performed the experiments at different room temperatures between 20 and 30 °C. In order to find the best working conditions, the fluorescence changes of all experiments were normalized to the highest value and compared with the nonexposed nanomaterial samples subjected to the same room conditions, in turn normalized to the lowest fluorescence value, and the differences were represented together for comparison purposes. To compare the effect of TATP on time, we adjusted the values to 30 min in all cases. In a representative experiment, 0.2523 g of TATP was weighed and placed in the room at 26 °C for 30 min: Of the 0.2523 g of TATP initially weighed, only 0.0386 g evaporated under the temperature and time conditions used in this experiment, and the remaining 0.2167 g of TATP remained as solid in the original sample; this gave a maximum concentration of 1.65 mg/m^3^ in the air of the room, supposing that all TATP evaporated during the experiment remained in the room, which is not a sealed system. Comparative photographs of selected experiments taken under UV light (366 nm) before and after exposure to TATP vapors are shown in Figures S71 and S72 and the normalized emission intensities of all individual experiments are shown in Figures S73 and S74. Figure 3 shows a 3D histogram plotting the differences in intensity emission in all cases, before and after exposure to TATP vapors, as a function of distance from the TATP source and temperature of the room, normalized to the top and reference signals. Generally speaking, there was a clear increase in fluorescence from the solid sensor samples, with several variations related to the inherent turbulence of the air stream, combined to the air heating system, and the specific sensitivity of the nanoparticles to the amounts of TATP detected. The detection of TATP in air was well assured in quantities of around one milligram per cubic meter of air in an ordinary room where there was air exchange, central heating, and consequently inherent air turbulence; this means that not all samples received proportional amounts of TATP in the gas phase. Positive detection was therefore guaranteed by at least three neat increases in the fluorescence within 10 cm and 2 m from the TATP source (Figure 3). Hence, the detection system is suitable for practical measurements in standard real-life scenarios.

**Figure 4:**
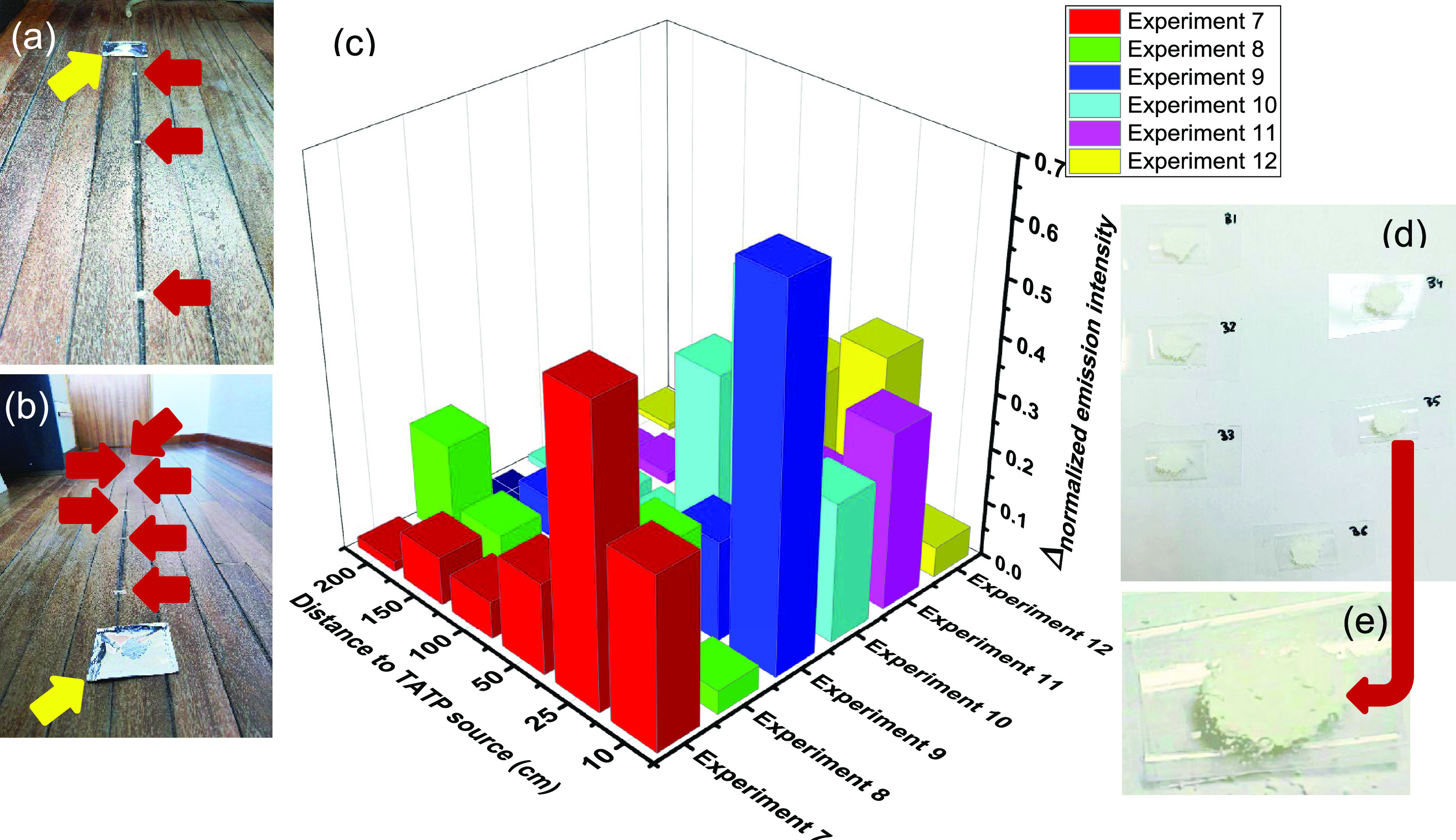
Summary of experiments 7–12. (a) Arrangement of the TATP in front of the airflow outlet. (b) Distribution of sensing nanoparticles in the room. (c) 3D representation of the variation of the normalized emission intensity as a function of the distance to the TATP source. (d) Assembly of the nanoparticles for measurements. (e) Details of one sample of AR82@SiO_2_.

From the results of the previous series of experiments, we observed that the best temperature to perform the experiment was around 26 °C. Therefore, a series of 6 additional experiments were performed at an average temperature of 26 °C for all experiments. The experiments were carried out in an office of dimensions 3.07 × 3.54 × 2.15 m^3^ (height × length × width) and had a duration of 30 min. Figures S76 and S77 illustrate both the dimensions and the arrangement of the different elements in the room as the experiments were performed. Inside the office, an air compressor was placed, which generated a continuous stream of clean air that passed through the TATP in the direction of the points where the particles were arranged. Initially, the temperature inside the room was regulated with a fan-forced convection air heater. When the temperature reached near 30 °C, the heater was turned off and the temperature was left to reach an average temperature of 26 °C for the performance of the experiments. Then, 0.75 mg of AR82s@SiO_2_ nanoparticles was placed on the surface of a cover glass and adhered to it with the help of a small amount of adhesive tape, as in previous experiments. This process was repeated six times, and the cover glasses were placed at increasing distances from the point where the TATP should be located (10, 25, 50, 100, 150, and 200 cm). To conduct every experiment, blank measurements of pristine nanoparticles left for 30 min in the room, with no presence of TATP, were performed and used as reference in every case. Then, 250 mg of TATP was placed at a 30 cm distance from the source of the airflow. Once TATP and nanoparticles were placed, the system was left in the presence of TATP for a period of 30 min so the measurements were made for each group of nanoparticles before and after the exposure to TATP vapors. After each experiment, the remaining amount of TATP was weighed and the fluorescence of nanoparticles was measured. To carry out the measurements in the fluorometer, each sample was covered with a quartz sheet to ensure that the nanoparticles remained in a fixed position (see Supporting Videos). This sheet was removed to leave the particles exposed to TATP vapors during the experiment. Pictures of the nanoparticles were taken under identical conditions in every case. The excitation wavelength was 370 nm. From the initial 250 mg samples of TATP, only a small amount evaporated in every case: 23, 23, 36, 36, 32, and 32 mg, an average of 30.3 mg of TATP involved in all of the experiments, 1.30 mg/m^3^ average, in a range of 0.98–1.54 mg/m^3^. Complete experimental data of all experiments are shown in Figures S78–S101. For easier visualization of the whole data, we graphically represented the normalized emission intensity variation before and after exposure to TATP vapors with respect to distance to the TATP source (Figure S102); this time the tendency of the data showed a clear decrease of the fluorescence intensity or the proportionality factor of fluorescence variation in each case from short to long distances from the TATP source, but again the number of fluctuations from the similarly performed experiments was high. For the representation of the data, the maximum emission intensity data from the experiments were organized according to the distance to the TATP source, representing in bars one next to the other, the emission values before and after contact with TATP vapors. These data were also normalized with respect to the maximum of emission from each experiment. After this, a common initial emission level was established using the lowest value measured for the nanoparticles prior to the exposure to TATP vapors. And finally, the subtraction was made between the final and initial emission values. Figure S104 shows the 2D representation, and Figure 4 shows the summary of experiments as a 3D representation of the variation of the normalized emission intensity as a function of the distance to the TATP source.

**Figure 5:**
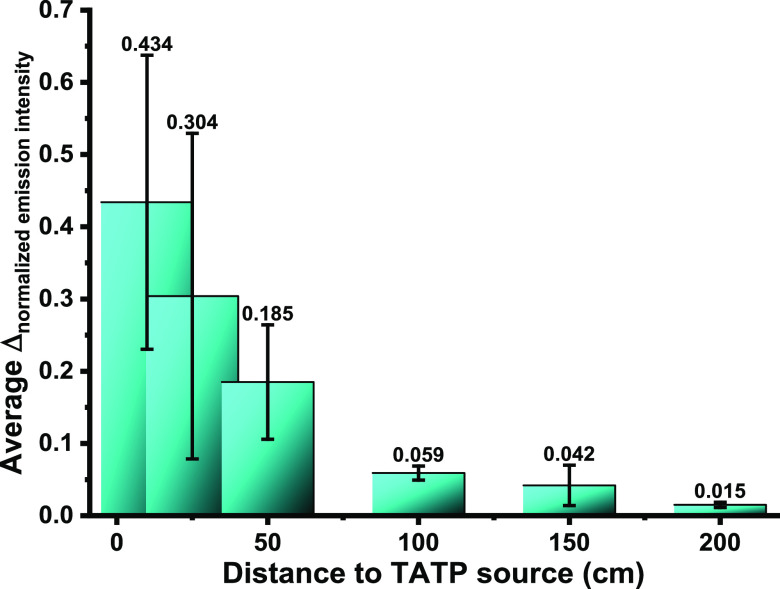
Average and standard deviation of normalized emission variation as a function of the distance to the TATP source.

With the aim to extract the tendency of the described experiments, a selection of the measurements was made, discarding outliers and selecting only 3 measurements for every one of the distances to the TATP source. Using these measurements, the average and standard deviation of the normalized variation of the emission intensity for each point were calculated and represented. Figure 5 shows the average and standard deviation of normalized emission variation as a function of the distance to the TATP source. Albeit the large fluctuations, represented by the large deviations from the normalized emission variations, it is clear that there is a marked tendency to stronger variations of the fluorescence as the detecting material is closer to the source of TATP vapor and the system is strong enough to work under close to real conditions appropriate to any environment where TATP vapor with a concentration of at least 1 mg/m^3^ is suspected to be found, in a nonsealed environment suitable for life scenarios that could be sensitive to terrorism attack.

### Mobile Phone Application

**Figure 6:**
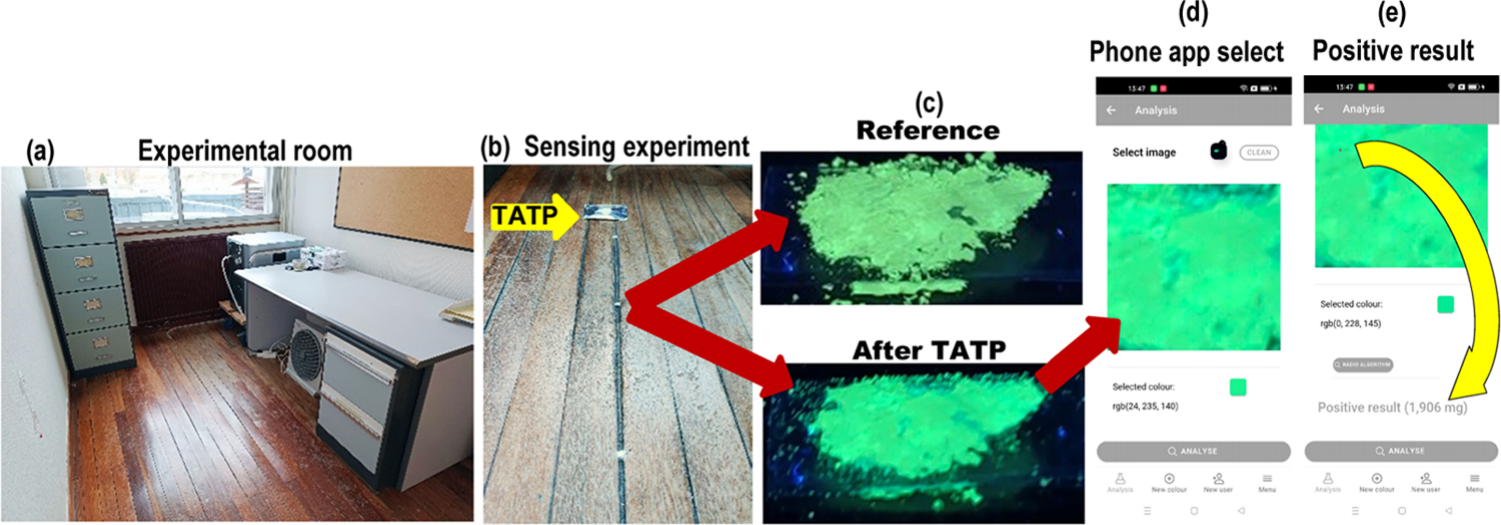
(a) View of the room for the experiment, (b) the TATP sample and the sensing material at different distances from the TATP, (c) picture of the sensing material placed at 25 cm from the TATP source, under a UV lamp, before (up) and after (down) being exposed to TATP, (d) picture (down) captured by the mobile phone app, and (e) the app assigned a positive value after comparing the RGB values in the selected point with the customized database.

A dedicated mobile phone to the application was used to detect the presence of TATP in the vapor flow. The purpose of the application was to detect the amount of TATP from an image of AR82s@SiO_2_ sample exposed to TATP vapors. The user had to configure the system correctly in order to obtain a suitable result. The application has two systems for determining the amount of TATP by RGB: (1) Interval search: each sample exposed to a known amount of TATP was configured with several colors, in which the target could be included. To detect the amount of TATP to which the nanoparticles were exposed, a color was selected and an interval search was carried out. Example: suppose we have a target color 50, 240, 70 and we have the following intervals [40, 130, 60], [10, 250, 60], and [55, 244, 71]. The comparison algorithm will detect that the target 50, 240, 70 is in the interval [40, 230, 60] 50, 240, 70 [55, 244, 71]. (2) Radio search: suppose we have a target color 50, 240, 70 and we work with a radius of 10. The intervals we have are [40, 230, 60], [10, 250, 60], and [85, 244, 41]. Then, the compare algorithm will detect that the target 50, 240, 70 is in the radius of the interval [40, 230, 60] since if we add the radius ±10, we would have this radius [40, 230, 60] or [60, 250, 80]. The system of the application was composed of the following components: (1) Mobile application: it was developed with IONIC technology with which we have the possibility of generating an APK for Android or IPA for IOS. (2) Laravel api: communication with the backend where queries were made from the mobile application to the database. (3) Database: system of tables designed to be able to store colors dynamically and to meet the needs of the application. System configuration: The application is made up of different configurable sections, (1) Login: to be able to enter the system, access credentials are required. (2) Menu: allows the user to view and access the different options of the application. (3) Users: the list of users authorized to access the application is displayed, as well as the option to delete them. (4) User settings: personal data (name, email, password, etc.) can be changed from this screen. (5) New user: it allows the user to create new users by filling in the required information. The data will be verified before granting access to the application. (6) Color list: list of the different nanoparticle colors for each amount of TATP in vapor phase, accessed from “menu” and then “my colors”. In this screen, colors can be deleted and the data updated at any time as well as to watch the rest of the stored colors. (7) New color: to add a new color to a TATP measurement, we click on “add color” and select the TATP quantity and add an RGB color, manually or by selecting it on an image. The latter is the fastest method as it allows the user to save and continue adding colors. (8) Color detection: once the system is configured with the different TATP measurements, in the “analysis” screen, the user can take a picture or select an image from the gallery and choose the point where the user wants to detect how much TATP it has been exposed to. The images are very important because the higher the quality, the higher the accuracy in detecting RGB color (see Figures S64–S66 for detailed images of the app). The application for the mobile phone was based on: *Ionic*https://ionicframework.com. *Laravel*, https://laravel.com/docs/8.x/eloquent-resources. *Database*, https://www.mysql.com. The application was part of an autonomous system for capturing and quantitative evaluation of fluorescent probe images based on mobile telephony composed of: (1) customized display box, (2) 360° metal bracket, (3) customized APP, and (4) OPPO Find X3 Pro 5G. The system allows taking photographs with a mobile phone camera from very short distances of a sensor material previously exposed to an explosive atmosphere. For this, the sensing material was placed in a dark box with a metal holder and a UV lamp (366 nm) to illuminate the material (Figure S68). The application to compare the photo with a database of images of the material exposed to different amounts of TATP in vapor phase is loaded on the mobile phone, consequently, from the comparison and the calculated calibration curve, the amount of the explosive that was in contact with the AR82s@SiO_2_ can be extracted. Initially, the system was trained and tested by using the experiments described in Figure 2. In this way, the system worked as a quick test for detecting TATP in air (see Figures S65 and S67 for detailed images of a selection of positive and negative detection cases) from samples with quantitative TATP treatment under controlled conditions, in those cases giving accurate results. Then, the system was validated under close to real conditions from samples obtained in experiments 7–12 (Figure 4). Details of experiments and the corresponding pictures are given in the Supporting Information (2-app measurements file). The selected images corresponded to nondiscarded measurements used for Figure 5, to allow the measurements to be compared with previous results obtained in those experiments. For maximal randomization, one sample from each experiment from the 7 to 12 series was measured. Positive results gave a value in mg, taken from experiments in Figure 2, that are converted to TATP concentrations by using the table of evaporation times from Figure S60 under the air stream flow (100 cm^3^/min). Therefore, exp. 8@10 cm and exp. 7@25 cm (Figure 6) distances, exposed to TATP vapor in the room, gave a value of 0.70 mg/m^3^ of TATP concentration in the air, exp. 10@50 cm, exp. 9@100 cm, and exp. 12@150 cm distances, exposed to TATP vapor in the room, gave a value of 0.24 mg/m^3^ of TATP concentration in the air, and exp. 11@200 cm distance, exposed to TATP vapor in the room, gave a value of 0.06 mg/m^3^ of TATP concentration in the air. On the other hand, nonexposed sample references from exp. 7@10 cm and exp. 10@100 cm gave in both cases a negative result, thus providing roughly approximate values of existence, or confirming inexistence, of TATP in the room, with the only assistance of the sensing material, a black box with a UV lamp, and a customized app installed in a mobile phone.

## Experimental Methods

**Figure 7:**
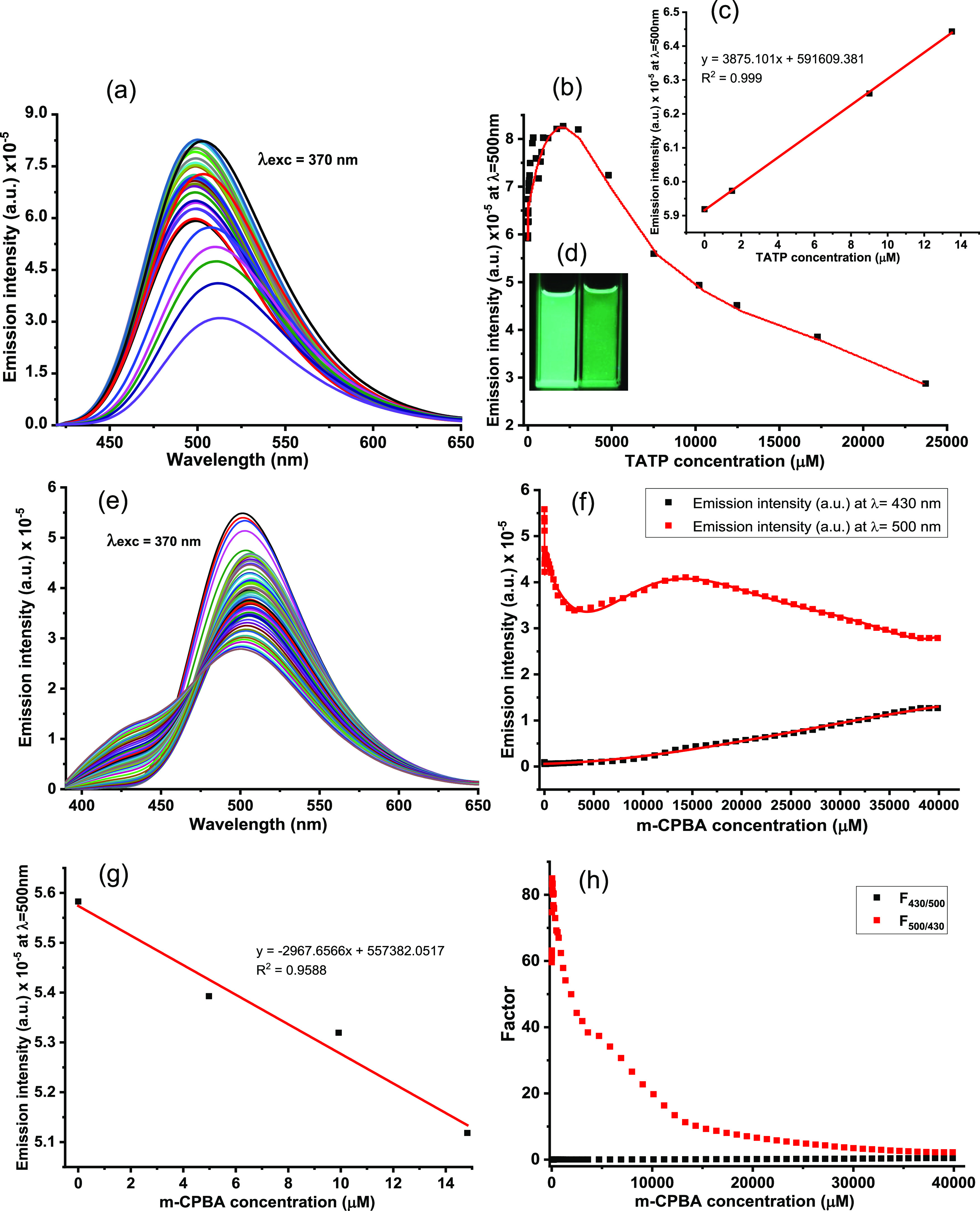
(a) Titration curves, TATP titration. (b) Fluorescence profile at 500 nm, TATP titration. (c) Calibration plot for the limit of detection of 2.5 μM GC2 solutions in DCM under increasing concentrations of TATP. (d) Image of solutions before and after TATP titration. (e) Titration curves, *m*-CPBA titration. (f) Fluorescence profile at 500 nm, *m*-CPBA titration. (g) Calibration plot for the limit of detection. (h) Fluorescence profile at the corresponding working curves of the ratiometric probe in the presence of *m*-CPBA.

The selection of the appropriate dye for TATP detection consisted of an iterative process. First, the synthesized compounds were fully characterized by physical and spectral methods, IR, ^1^H/^13^C NMR, HRMS (MALDI/ESI), UV–vis, FL, Ø, τ. UV–vis and fluorescence measurements were performed in a series of 14 solvents from high to low polarity [1. H_2_O, 2. MeOH, 3. DMSO, 4. DMF, 5. MeCN, 6. Acetone, 7. EtOAc, 8. THF, 9. CHCl_3_, 10. CH_2_Cl_2_, 11. Toluene, 12. Et_2_O, 13. Hexane, 14. Methylcyclohexane (MCH)]. Then, compounds are tested in solution for the sensitivity to low to high amounts of water (0 to 90% water, usually in THF), pH values (3.4–10.4), anions (11 representative anions: 1. F^–^, 2. Cl^–^, 3. Br^–^, 4. I^–^, 5. BzO^–^, 6. NO^3–^, 7. H_2_PO_4_^–^, 8. HSO_4_^–^, 9. AcO^–^, 10. CN^–^, 11. SCN^–^), cations (18 representative cations: 1. Ag^+^, 2. Ni^2+^, 3. Sn^2+^, 4. Cd^2+^, 5. Zn^2+^, 6. Pb^2+^, 7. Cu^2+^, 8. Fe^3+^, 9. Sc^3+^, 10. Al^3+^, 11. Hg^2+^, 12. Au^2+^, 13. Co^2+^, 14. Pd^2+^, 15. Ir^3+^, 16. Cu^+^, 17. Ru^3+^, 18. Pt^2+^), oxidizing and reducing agents in different solvents [(addition in water, series A: 1. HCl, 2. HNO_3_, 3. Oxone, 4. Hydrazine, 5. H_2_O_2_) (addition in MeOH, series B: 1. *Meta*-chloroperbenzoic acid (*m*-CPBA), 2. Trinitrobenzene (TNB), 3. Trinitrotoluene (TNT)) (addition as solid, series C: 1. *m*-CPBA, 2. TATP, 3. HMTD)] on suitable diluted solutions of the dye in an organic solvent, usually miscible with water, and the physical changes were followed by fluorescence measurements. Those compounds showing the utmost performance in terms of sensitivity and selectivity to the presence of peroxide-carried oxidants (except hydrogen peroxide) are then subjected to TATP sensitivity in different solvents, and the changes are monitored by fluorescence measurements. Then, kinetic behavior and titration experiments were performed, first in different solvents, then by accurate titration in the best solvent, selected from previous experiments. When the detection limit was sufficiently low, the study of detection of TATP in the gas phase was subsequently performed with the colorant adsorbed on the surface of silica, anatase, or hybrid nanoparticles. The experiments of sensitivity to TATP in the gas phase by solid-supported dyes were performed by comparing the increase of the quantum yield of the samples in the presence or absence of TATP under controlled conditions. Compound AR82s showed the best performance in all tests; therefore, it was selected for the experiments of TATP detection in the gas phase under real conditions in the open air. The mechanism of detection of TATP was carefully studied in solution by comparison of the action of TATP and *m*-CPBA, under the hypothesis that TATP acts in the experiments as a mild oxidant. We performed a point-to-point titration of AR82s (2.5 μM in CH_2_Cl_2_) in the presence of increasing amounts of TATP by preparing 33 solutions of AR82s (2.5 μM in CH_2_Cl_2_). TATP was added as a solid to each of them, resulting in 33 solutions of concentrations between 0 μM and 23.72 mM. The fluorescence changes were registered immediately after the addition of TATP to AR82s (2.5 μM in CH_2_Cl_2_), λ_exc_ = 370 nm and λ_em_ (CH_2_Cl_2_) = 500 nm, at 25 °C (Figure 7). The TATP titration plot showed an increase in fluorescence at low concentrations of TATP and a decrease at higher concentrations. We calculated the limit of detection, LOD, from the initial titration plot values by IUPAC-consistent methods.^65^ In this case, we calculated the LOD within the values measured (different than 0) by adjusting the initial values to a mean square linear regression and using the R program.^66,67^ In this way, linear regression at low concentrations of TATP led to a LOD = 0.87 μM or 0.19 μg·mL^–1^ of TATP, which was suitable for practical purposes. Analogously, we performed a point-to-point titration of a 2.5 μM solution of AR82s in CH_2_Cl_2_ and increasing amounts of *m*-CPBA (the oxidant concentration maintained between 0 and 0.02 M), and measuring the variation of the fluorescence after each addition with an Edinburg Instrument FLS-980 fluorometer. Measurements were carried out immediately after every addition of *m*-CPBA. λ_exc_ = 370 nm and λ_em_ = 430 and 500 nm, at 25 °C. The *m*-CPBA titration plot showed a decrease in fluorescence of the green emission at 500 nm at low concentrations of *m*-CPBA and an increase at higher concentrations, followed by a further decrease. We calculated the limit of detection, LOD, from the initial titration plot values by IUPAC-consistent methods.^65^ In this case, we calculated the LOD within the values measured (different than 0) by adjusting the initial values to a mean square linear regression and using the R program.^66,67^ In this way, linear regression at low concentrations of *m*-CPBA led to a LOD = 2.1 nM or 0.3 ng·mL^–1^ of *m*-CPBA, which was only interesting for comparison purposes (Figure 7). The blue emission at 430 nm showed instead a slow increase in intensity. The ratios of fluorescence intensities at 500 and 430 nm (*F*_500_/*F*_430_) exhibited good linearity with increasing *m*-CPBA concentrations in the range from 0 to 20 μM (Figure 7). The linear regression equation was *F*_500/430_ = 0.1744*x* + 59.8340.

### ^1^H NMR Titration of AR82s with TATP in the Presence of Amberlite

**Figure 8:**
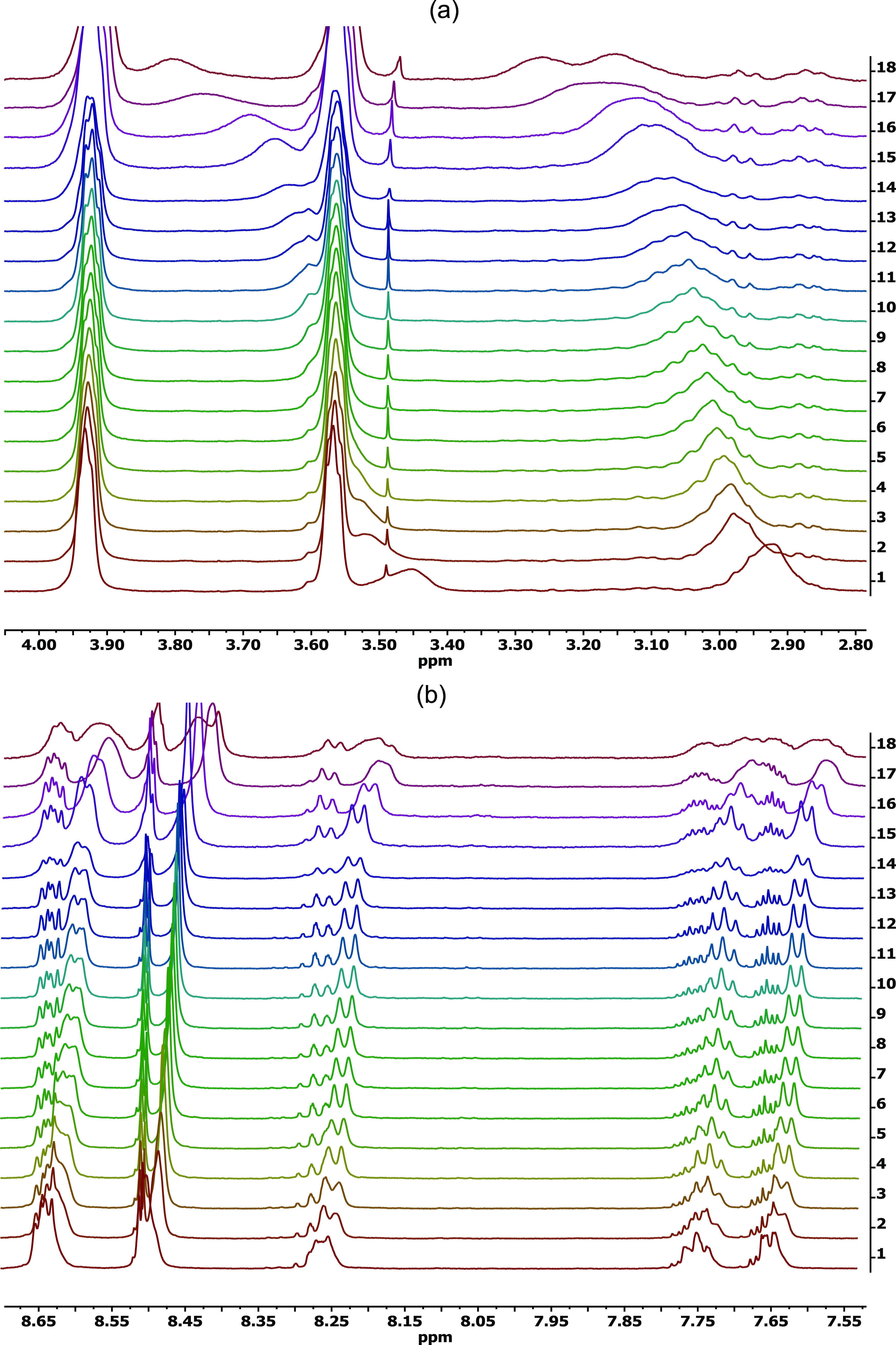
^1^H NMR (CDCl_3_, 500 MHz) titration of AR82s with increasing amounts of TATP in the presence of amberlite, 3 mg of AR82s in 0.5 mL of CDCl_3_, and TATP amounts: (1) 0 μg, (2) 5 μg, (3) 10 μg, (4) 15 μg, (5) 20 μg, (6) 30 μg, (7) 40 μg, (8) 60 μg, (9) 90 μg, (10) 150 μg, (11) 300 μg, (12) 500 μg, (13) 800 μg, (14) 1400 μg, (15) 3200 μg, (16) 6800 μg, (17) 10 400 μg, (18) 15 800 μg as total added TATP after each addition, (a) 2.8–4.0 ppm region, (b) 7.5–8.7 ppm region.

We performed a ^1^H NMR titration of AR82s (3 mg in 0.5 mL in CDCl_3_) with increasing amounts of TATP in the presence of Amberlite (1.5 mg) to prevent accumulation of TATP in the NMR tube. Two selected regions gave clear NMR modifications (Figure 8).

The titrations showed chemical shift of the signals of protons in the vicinity of the unprotected secondary amino group, signals at 2.93 and 3.46 ppm, accompanied by splitting of the signal at 2.93 at high amounts of TATP. There are no changes in the piperazine proton signals at 3.58 and 3.93 ppm. On the aromatic region, there is a clear splitting of all aromatic signals with changes in the coupling patterns between the signals, indicating a desymmetrization of the molecule. Apparently, oxidation of the unprotected secondary amino group will trigger the chemical shift of the methylene groups of the oxidized piperidine group as well as the association between molecules in solution, with a concomitant change in fluorescence either by suppressing the charge transfer from the amine group or by modifying the aggregation-induced effect when in solution because of the modification of the structure, in relation to the variation in the AIE effect we have observed for the rest of synthesized examples. We then studied the mechanism of oxidation of AR82s by NMR titration in the presence of increasing amounts of *m*-CPBA, and the titrations showed only oxidation of the unprotected secondary amino group with progressive disappearance of the proton signal of the amine group and chemical shift of the methylene groups of the oxidized piperidine group (the signals between 1.8 and 3.7 ppm in Figure S112), therefore confirming the oxidation mechanism. There is also splitting of the aromatic signals but, in this case, the changes in the aromatic region are obscured by the presence of the signals of the *m*-CPBA protons.

## Conclusions

We have described the proof of concept of a portable testing setup for the detection of triacetone triperoxide (TATP), a common component in improvised explosive devices. The system allows for field testing and generation of real-time results to test for TATP vapor traces in air by simply using circulation of the gas samples through the sensing material under the air conditioning system of an ordinary room. In this way, the controlled trapping of the analyte in the chemical sensor gives reliable results at extremely low concentrations of TATP in air under real-life conditions. The system may have immediate applications in everyday use. Air samples at airports, government buildings, sports arenas, or concert halls, where many people gather, should be checked for IED-containing peroxide explosives to prevent terrorist acts. The system provides for an extremely low limit of detection of traces of TATP in air as it is required to be of practical use. Vapor detection as we have shown here is a useful and noninvasive method suitable for explosive detection among current explosive detection technologies. TATP still goes largely unnoticed in many densely populated places, where permanent monitorization is needed. The results now reported are ready to convert the current knowledge in everyday useful technology to be applied whenever it is necessary for the detection of traces of IEDs in luggage storage, sports depots, headquarters, locker rooms, or restricted public access, before they can cause any harm.

## Supporting Information Available

Complete characterization for all compounds, additional experimental details, materials, and methods (PDF)
Fluorometer measurements with nanoparticles in a fixed position (MP4)
Fluorometer measurements with nanoparticles in a fixed position (MP4)
Photographs of experimental setup (PDF)

## Abbreviations

TATP: triacetone triperoxide
IED: improvised explosive devices
TNT: trinitrotoluene
HMTD: hexamethylene triperoxide diamine
AIE: aggregation-induced emission
PDMS: poly(dimethylsiloxane)
GC2@SiO_2_: C2-supported silica nanoparticles
TEM: transmission electron microscopy
LOD: limit of detection
MCH: methylcyclohexane
*m*-CPBA: *meta*-chloroperbenzoic acid
TNB: trinitrobenzene
IR: infrared
NMR: nuclear magnetic resonance
HRMS: high-resolution mass spectrometry
MALDI: matrix-assisted laser desorption/ionization
ESI: electrospray ionization
UV–Vis: ultraviolet–visible
FL: fluorescence
Ø: quantum yield
τ: lifetime
