# Supplementary material for: Silica Nanoparticle/Fluorescent Dye Assembly Capable of Ultrasensitively Detecting Airborne Triacetone Triperoxide: Proof-of-Concept Detection of Improvised Explosive Devices in the Workroom
Source: ACS Appl Mater Interfaces. 2023 Jun 21;15(26):32024–36. doi: 10.1021/acsami.3c05931 (PMC10326811; doi:10.1021/acsami.3c05931)
Supplement: Supplementary file 1 — am3c05931_si_001.pdf [file am3c05931_si_001.pdf]

Supporting Information

Silica Nanoparticle/Fluorescent Dye Assembly Capable of Ultrasensitively Detecting Airborne Triacetone Triperoxide: Proof-of-Concept Detection of Improvised Explosive Devices in the Workroom.

*Andrea Revilla-Cuesta,[a] Irene Abajo-Cuadrado,[a] María Medrano,[a] Mateo M. Salgado,[a] Manuel Avella,[a] María Teresa Rodríguez,[a] José García-Calvo,[#a] and Tomás Torroba[a]**

[a]Department of Chemistry, Faculty of Science, University of Burgos, 09001 Burgos, Spain.

[b]Electron Microscopy Lab, IMDEA Materials Institute, Eric Kandel, 2, Tecnogetafe, 28906 Getafe (Madrid), Spain.

*E-mail: ttorroba@ubu.es

Present Address: [#]J.G.C.: IMDEA Nanociencia Institute, Faraday 9, 28049 Madrid, Spain.

General methods …………………………………………..……………………………...S02

N-(*N'*-Boc-piperidin-4-yl)-4-bromonaphthalene-1,8-dicarboxylmonoimide (AR43p)..S03-S05

N-(Piperidin-4-yl)-4-bromonaphthalene-1,8-dicarboxylmonoimide (AR43d)………...S05-S07

N-(*N'*-Boc-piperidin-4-yl)-4-[2-(4-Boc-piperazin-1-yl)pyrimidin-5-yl]naphthalene-1,8-dicarboxylmonoimide (AR82p)……………………………………..……………....S08-S14

N-(Piperidin-4-yl)-4-[2-(4-Boc-piperazin-1-yl)pyrimidin-5-yl]naphthalene-1,8-dicarboxylmonoimide (AR82s)…………………………………………………………….……….S15-S66

N-(Piperidin-4-yl)-4-[2-(piperazin-1-yl)pyrimidin-5-yl]naphthalene-1,8-dicarboxylmonoimide (AR82d)…………………………………………………………………………S67-S96

N-(*N'*-Boc-piperidin-4-yl)-4-[2-(4-Boc-piperazin-1-yl)pyridin-5-yl]naphthalene-1,8-dicarboxylmonoimide (AR83p)…………………………………………………………S98-S105

N-(Piperidin-4-yl)-4-[2-(4-Boc-piperazin-1-yl)pyridin-5-yl]naphthalene-1,8-dicarboxylmonoimide (AR83s)…………………………………….……………………S106-S111

N-(Piperidin-4-yl)-4-[2-(piperazin-1-yl)pyridin-5-yl]naphthalene-1,8-dicarboxylmonoimide (AR83d)……………………………………………………………………S112-S126

N-(*N'*-Boc-piperidin-4-yl)-4-[(4-Boc-piperazin-1-yl)phenyl]naphthalene-1,8-dicarboxylmonoimide (AR90p)……………………………………………………S127-S133

N-(Piperidin-4-yl)-4-[(4-Boc-piperazin-1-yl)phenyl]naphthalene-1,8-dicarboxylmonoimide (AR90s)……………………………………………………………………S134-S148

N-(Piperidin-4-yl)-4-[(4-piperazin-1-yl)phenyl]naphthalene-1,8-dicarboxylmonoimide (AR90d)……………………………………………………………………S149-S160

Synthesis of chemical probes and additional experiments.

General methods: Melting points were determined on an electrothermal melting point Gallenkamp apparatus and are uncorrected. Infrared Spectra were recorded with the potassium bromide pellet method, with a JASCO FT/IR-4200 spectrometer. Nuclear magnetic resonance (NMR) spectra were recorded with Varian Mercury-300 and Varian Unity Inova-400 spectrometers at room temperature (25ºC), with $CDCl_3$, CD_3CN and CD_3OD as solvents. Chemical shifts (δ) were reported in parts per million (*ppm*) relative to the residual solvent peaks, rounded to the nearest 0.01 for ^{1}H-NMR and 0.1 for ^{13}C-NMR. Spin-spin coupling constants (J) in ^{1}H-NMR were given in Hz, rounded to the nearest 0.1 Hz. Peak multiplicity was indicated as follows: *s* (singlet), *d* (doublet), *t* (triplet), *q* (quartet), *m* (multiplet) and *br* (broad). Relative integrals were given in ^{1}H-NMR too. All ^{13}C NMR were recorded with complete proton decoupling. Carbon types, structure assignments and attribution of peaks were determined from ^{13}C-DEPT-NMR. NMR spectra were analyzed using MestReNova NMR data processing software. MALDI-TOF mass spectra were measured with a MALDI-TOF Bruker Autoflex Mass Spectrometry instrument, using DCTB (*trans*-2-[3-(4-tert-butylphenyl)-2-methyl-2-propenylidene]malononitrile) or DIT (dithranol) as matrixes, in modes positive or negative. The atomic mass of the molecular ion (and/or fragments) per elementary charge were reported in dimensionless quantities. Absorption spectra were acquired with a Hitachi U-3900 spectrometer, in one-centimeter quartz cells at 25ºC. Emission spectra were recorded with a Hitachi F-7000 FL or a modular Edinburgh Instruments FLS980 spectrofluorometers, in one-centimeter quartz cells at 25ºC. Solvatochromism Tests: All samples were freshly prepared in each different solvent. Photos were taken with a Canon (EOS M3) camera with a 22 mm lens. Absorption spectra were acquired with a Hitachi U-3900 spectrometer, in one-centimeter quartz cells at 25ºC. Emission spectra were recorded with Hitachi F-7000 FL spectrofluorometer, in one-centimeter quartz cells at 25ºC. The solvents were, if it is not said otherwise: 1: H_2O, 2: MeOH (methanol), 3: DMSO (dimethyl sulfoxide), 4: DMF (*N,N'*-dimethylformamide), 5: MeCN (acetonitrile), 6: Acetone, 7: AcOEt (ethyl acetate), 8: THF (tetrahydrofuran), 9: $CHCl_3$, 10: CH_2Cl_2 (dichloromethane), 11: Toluene, 12: Et_2O (diethyl ether), 13: *n*-Hx (hexane), 14: *c*-Hx (cyclohexane). Fluorescence Lifetime Decays: Chromophore solutions were freshly prepared in a concentration of 1-10 μM in the corresponding solvent. The decay was fitted (black) with a sum of two exponentials by convolution with the instrumental response function (red). The quality of the fit was judged by $\chi 2$ values and the plot of the weighted residues. Measurements were made with a modular spectrometer Edinburgh Instruments FLS980, with a source excitation pulsed lasers of picosecond diode: 366-380 nm, 398-410 nm, 437-446 nm, 470-478 nm, 505-515 nm and 635 nm. One-centimeter quartz cells, at 25 ºC, were employed. Quantum Yields: Fresh solutions made from high-purity dyes (or mixtures of dye with an additive) and solvent were used. Integrating sphere was used as the method and three measurements were performed for each sample in order to calculate the average. Solutions were prepared in a concentration of 1-10 μM in the corresponding solvent. The excitation wavelength is indicated in each case. Measurements were made with a modular spectrometer Edinburgh Instruments FLS980. One-centimeter quartz cells, at 25ºC, were employed.

Synthesis of *N*-(*N'*-Boc-piperidin-4-yl)-4-bromonaphthalene-1,8-dicarboxylmonoimide (AR43p)

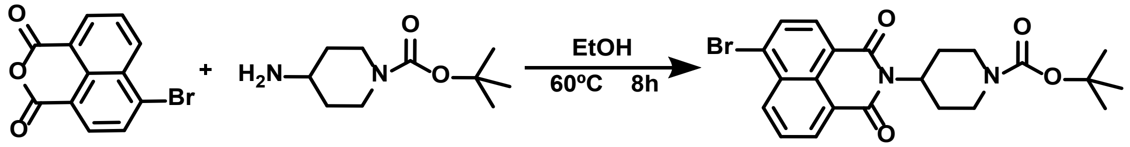

In a 100 ml flask, provided with a magnetic stirrer, 2 g of 4-bromo-1,8-naphtalic anhydride (7.22 mmol) and 2.9 g of 4-amino-1-Boc-piperidine (14.44 mmol) were dissolved in 40 ml of ethanol and the mixture was stirred 8 hours at 60ºC. After that, the mixture was filtered, cooling to room temperature and the solvent was evaporated under reduced pressure. The organic solid was purified by column chromatography (SiO_2, CH_2Cl_2), getting 3.15 g of AR43p, white solid, 95% yield. m.p.: 148 – 150ºC. IR (ATR, cm^{-1}): 2977 – 2849, 1689 (C=O), 1649 (C=O), 1419 (C-O), 1359, 1344, 1244, 1173 (C-N), 1143 (-CH_3), 1101, 1032 (C-Br), 981, 894, 774, 747, 424. ^{1}H-NMR (300 MHz, $CDCl_3$) δ (ppm): 8.48 (d, J = 7.1 Hz, 1H, H_{Ar}), 8.35 (d, J = 8.4 Hz, 1H, H_{Ar}), 8.23 (d, J = 7.9 Hz, 1H, H_{Ar}), 7.86 (d, J = 7.9 Hz, 1H, H_{Ar}), 7.70 (t, J = 7.9 Hz, 1H, H_{Ar}), 5.17 – 5.02 (m, 1H, CH), 4.26 (s, 2H, CH_2), 2.69 (m, 4H, 2×CH_2), 1.64 (m, 2H, CH_2), 1.45 (s, 9H, 3×CH_3). ^{13}C-NMR (75MHz, $CDCl_3$) δ (ppm): 163.3 (C=O), 163.2 (C=O), 154.3 (C=O), 132.4 (C_{Ar}H), 131.5 (C_{Ar}H), 130.7 (C_{Ar}H), 130.6 (C_{Ar}H), 129.8 (C_{Ar}), 129.5 (C_{Ar}), 128.3 (C_{Ar}), 127.6 (C_{Ar}H), 122.9 (C_{Ar}), 122.0 (C_{Ar}), 79.2 (Cq), 51.6 (CH), 43.8 (CH_2), 28.2 (CH_3), 27.9 (CH_2). HRMS (MALDI, DCTB-) m/z: calculated for $C_{22}H_{23}BrN_2O_4$: 458.0836 (M^-); found 458.0802.

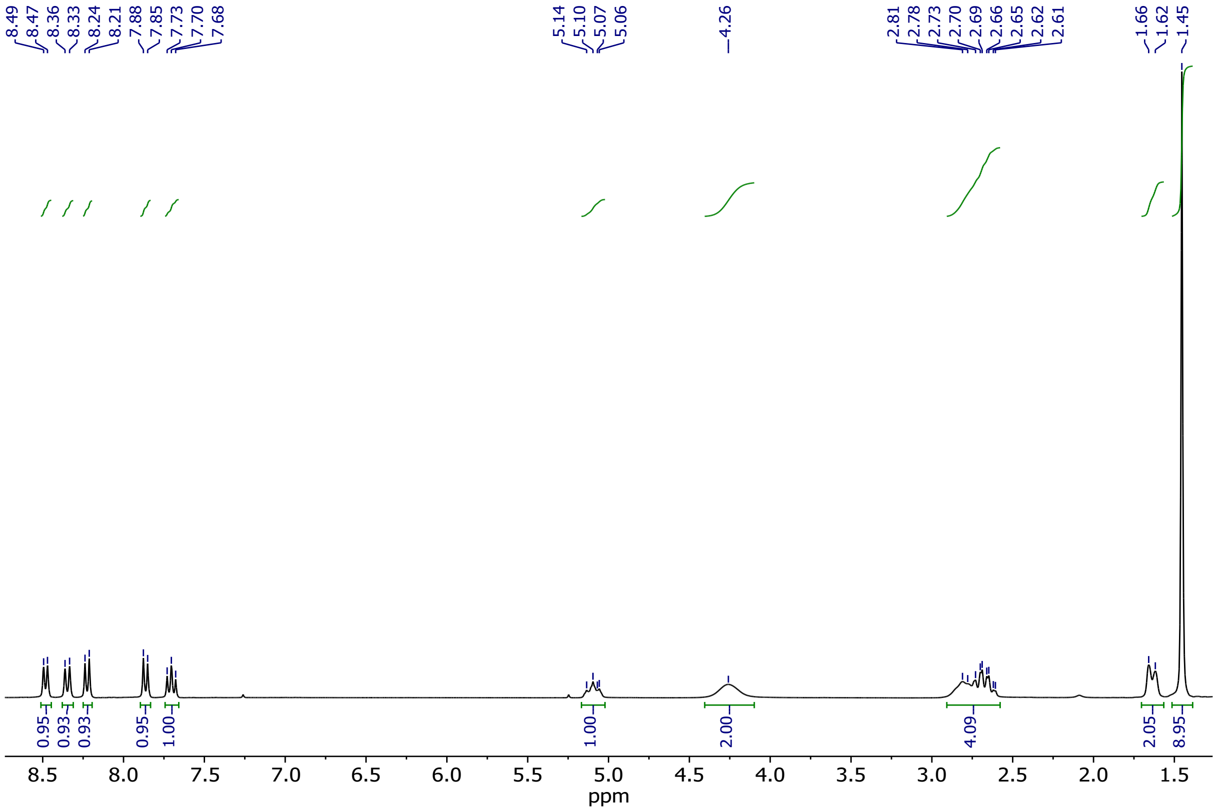

Figure S1. ^{1}H-NMR ($CDCl_3$, 300 MHz) of AR43p.

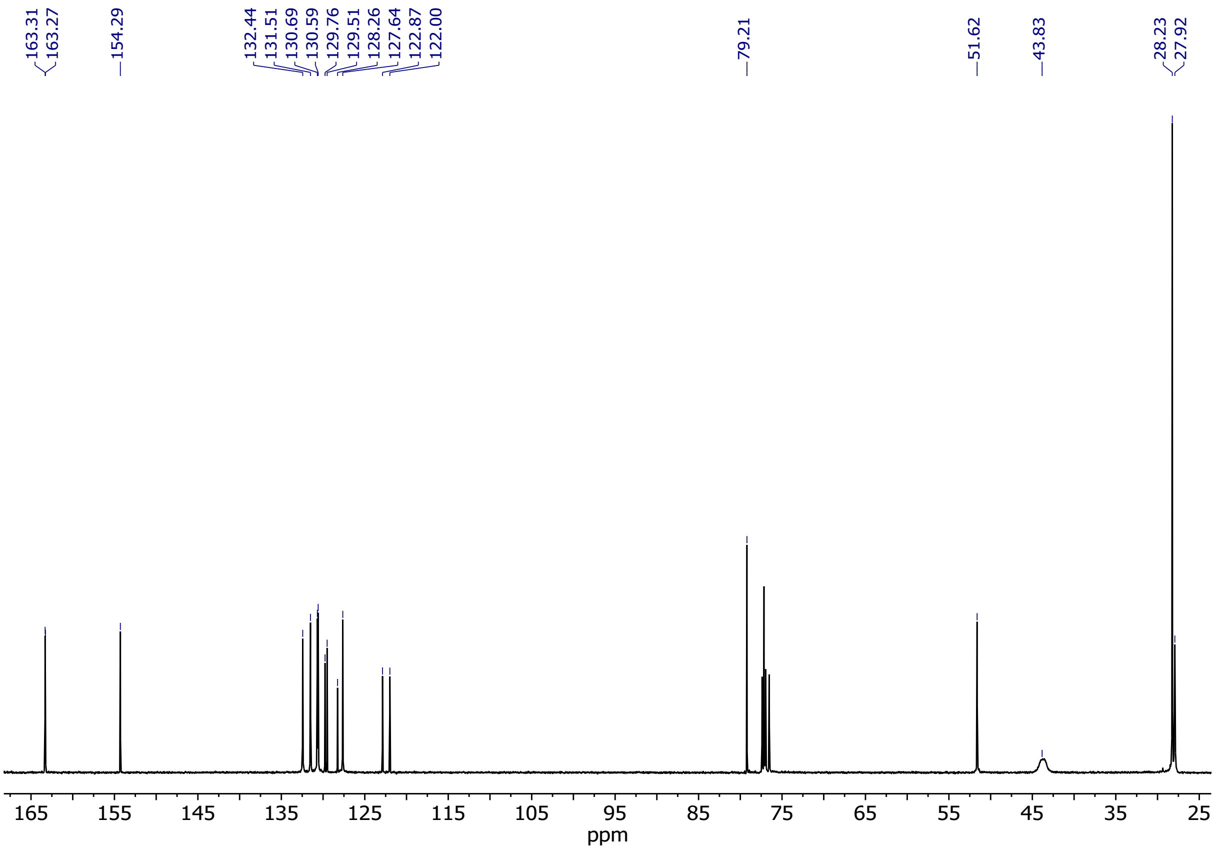

Figure S2. ^{13}C-NMR (CDCl$_3$, 75 MHz) of AR43p.

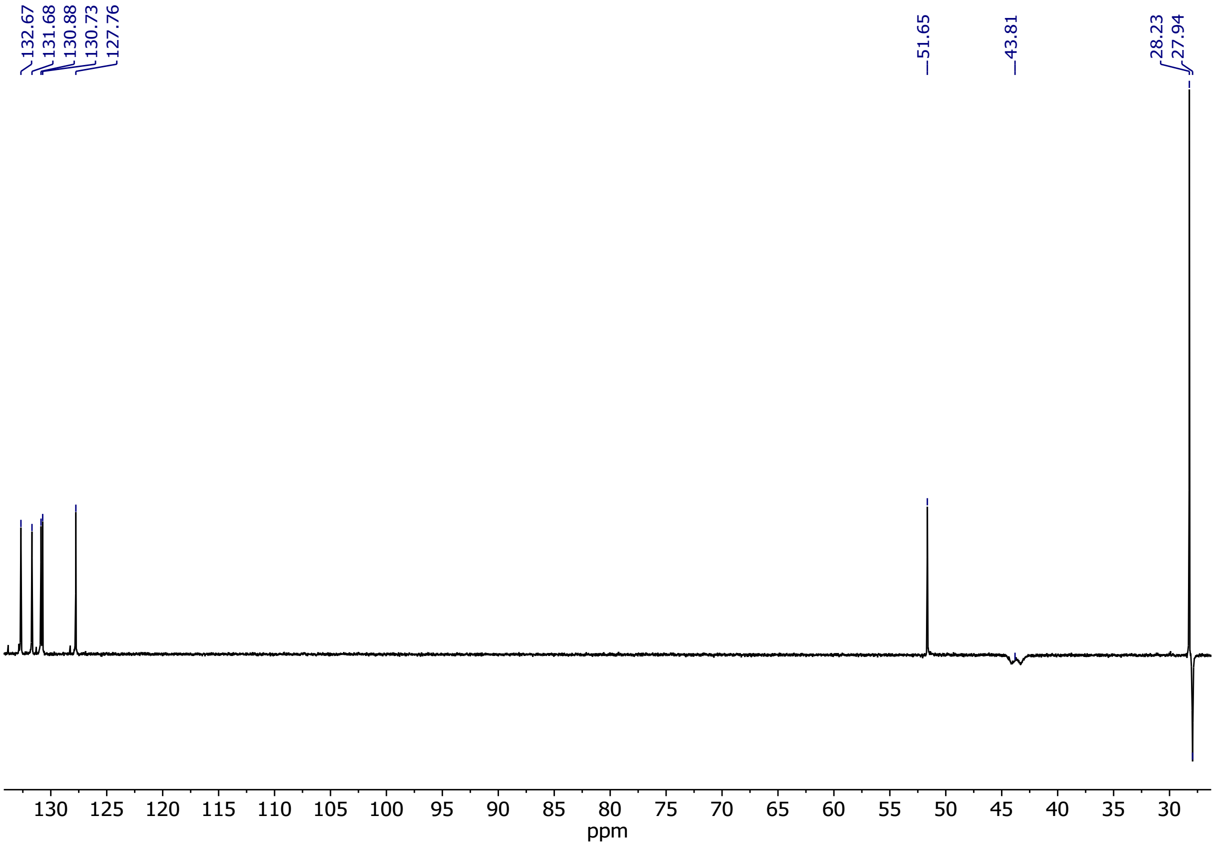

Figure S3. DEPT NMR (CDCl$_3$, 75 MHz) of AR43p.

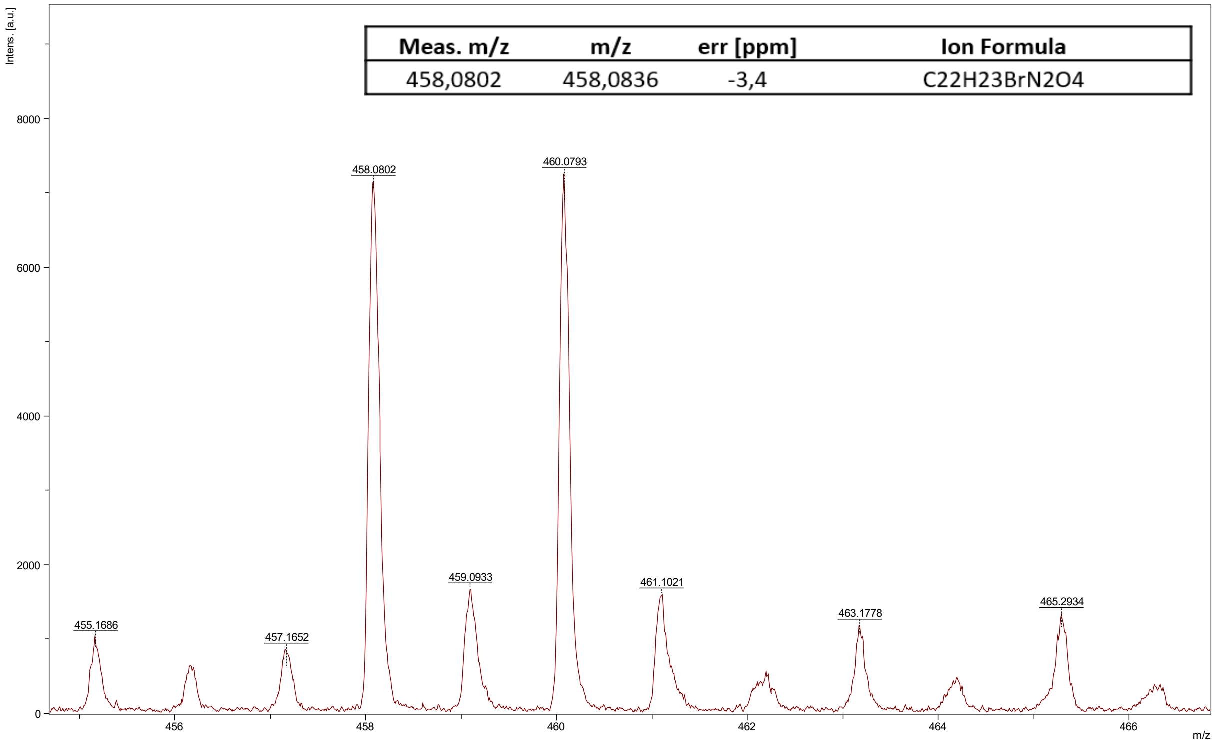

Figure S4. HRMS (MALDI, DCTB-) of AR43p.

Synthesis of *N*-(piperidin-4-yl)-4-bromonaphthalene-1,8-dicarboxylmonoimide (AR43d)

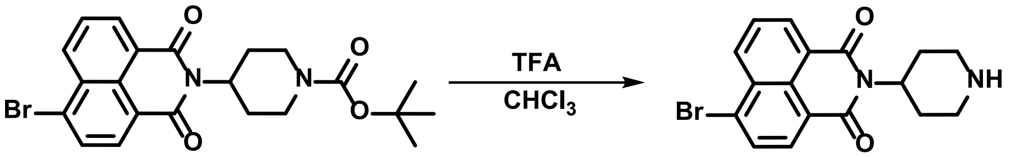

In a 50 ml flask with a magnetic stirrer, 150 mg of *N*-(*N'*-Boc-piperidin-4-yl)-4-bromonaphthalene-1,8-dicarboxylmonoimide (AR43, 0.33 mmol) were dissolved in 15 ml of chloroform and 2 ml of trifluoroacetic acid (26 mmol) were added, and the mixture was stirred for 4 hours at room temperature. Afterwards, 10 ml of water were poured into the flask and the mixture was neutralized employing a 40% sodium hydroxide solution until it reached a neutral pH. Then, 20 ml of water were added and the mixture was extracted with chloroform (3×30 ml). The organic extracts were combined, dried over anhydrous Na_2SO_4 and the solvent was evaporated under reduced pressure. The product AR43d was obtained quantitatively as a white solid, 100% yield (117 mg). m.p.: 88 – 89ºC. IR (ATR, cm^{-1}): 3359 (N-H), 2973 – 2830, 1686 (C=O), 1579, 1477, 1417, 1390, 1357, 1238, 1162 (C-N), 1121, 1097, 1076, 1049, 998 (C-Br), 932, 861, 807, 761, 642, 543, 511, 462, 408. ^{1}H-NMR (300 MHz, CD_3OD) δ (ppm): 8.61 – 8.55 (m, 2H, 2×H_{Ar}), 8.36 (d, *J* = 7.9 Hz, 1H, H_{Ar}), 8.10 (d, *J* = 7.9 Hz, 1H, H_{Ar}), 7.90 (dd, *J* = 8.5, 7.3 Hz, 1H, H_{Ar}), 5.31 (m, 1H, CH), 3.60 – 3.52 (m, 2H, NH y ½CH_2), 3.27 – 2.93 (m, 5H, 2×CH_2 y ½CH_2), 2.04 – 1.95 (m, 2H, CH_2). ^{13}C-NMR (75MHz, $CDCl_3$) δ (ppm): 163.7 (C=O), 133.1 (C_{Ar}H), 132.1 (C_{Ar}H), 131.3 (C_{Ar}H), 131.1 (C_{Ar}H), 128.9 (C_{Ar}), 128.1 (C_{Ar}H), 123.3 (C_{Ar}), 122.4 (C_{Ar}), 53.4 (Cq), 50.6 (CH), 45.5 (CH_2), 27.7 (CH_2). HRMS (MALDI, DCTB+) m/z: calculated for $C_{17}H_{15}BrN_2O_2$: 359.0390 (M^+ + H); found 359.0391.

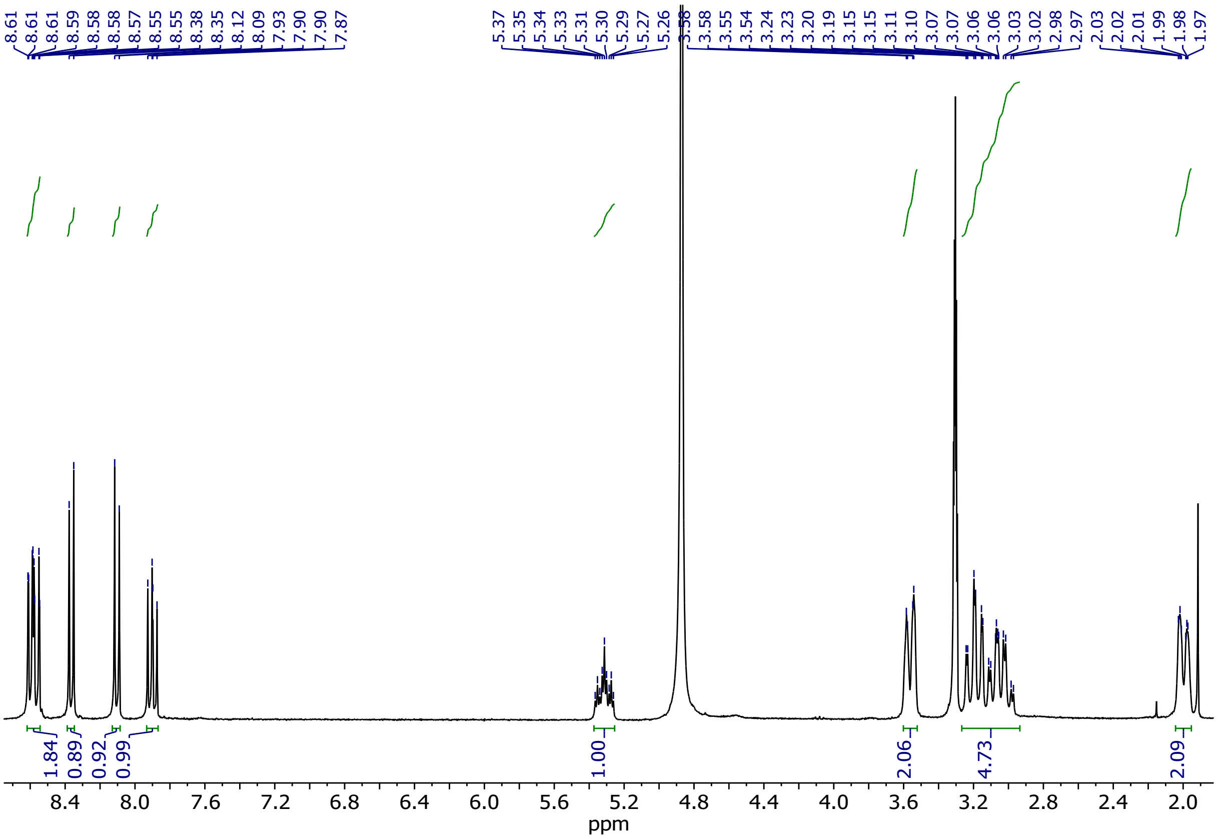

Figure S5. ^{1}H-NMR (CD_3OD, 300 MHz) of AR43d.

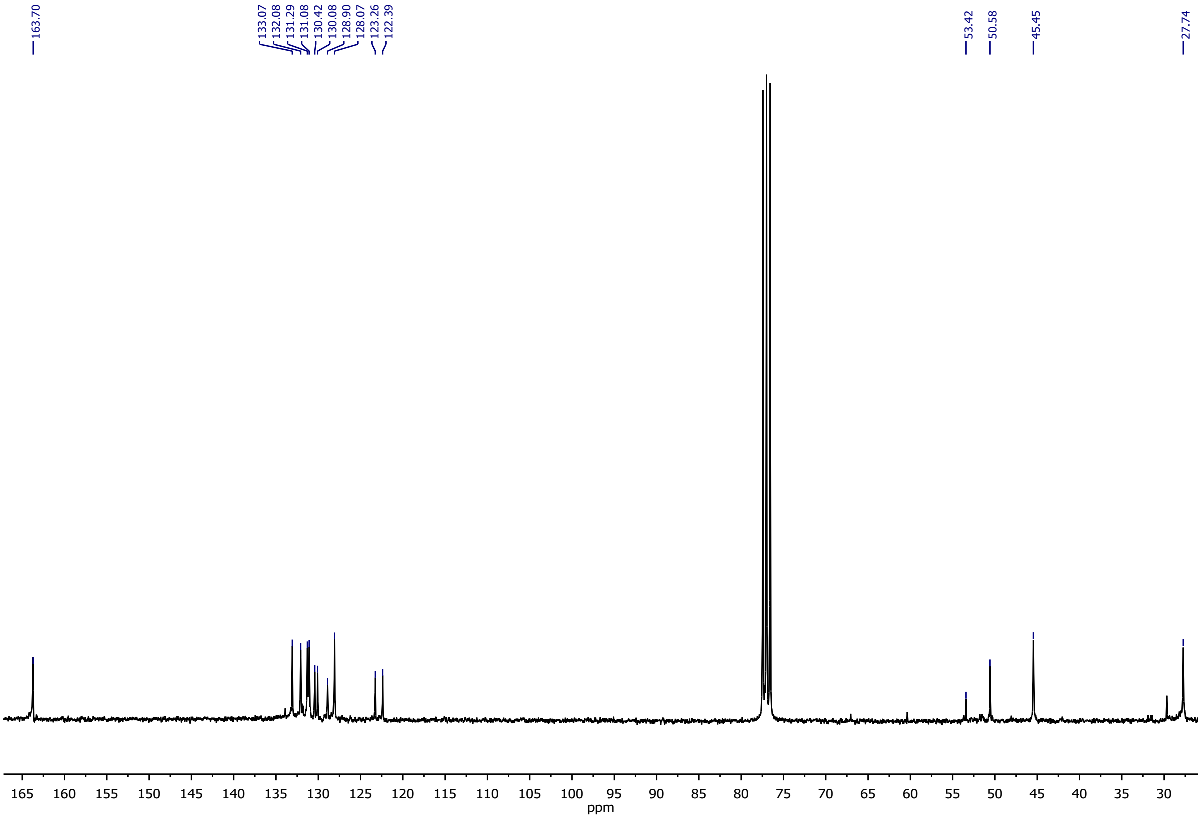

Figure S6. ^{13}C-NMR ($CDCl_3$, 75 MHz) of AR43d.

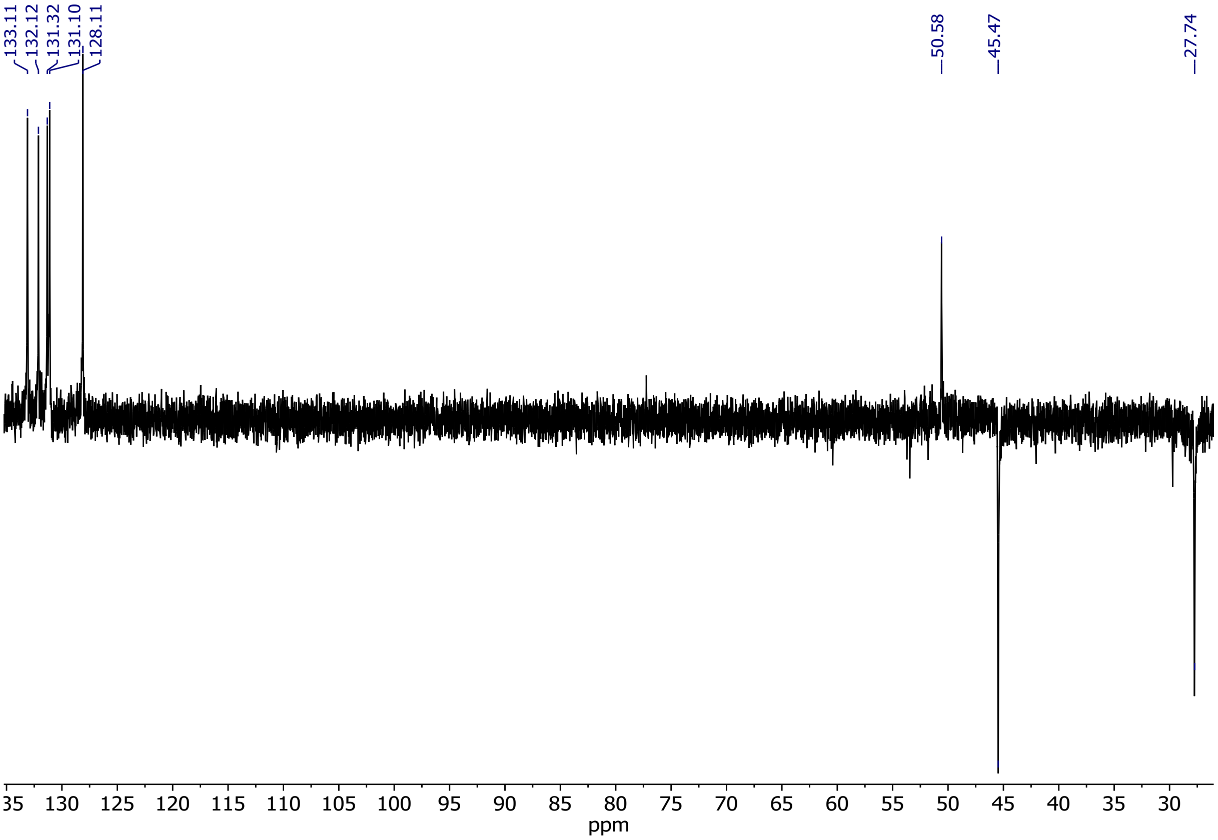

Figure S7. DEPT NMR ($CDCl_3$, 75 MHz) of AR43d.

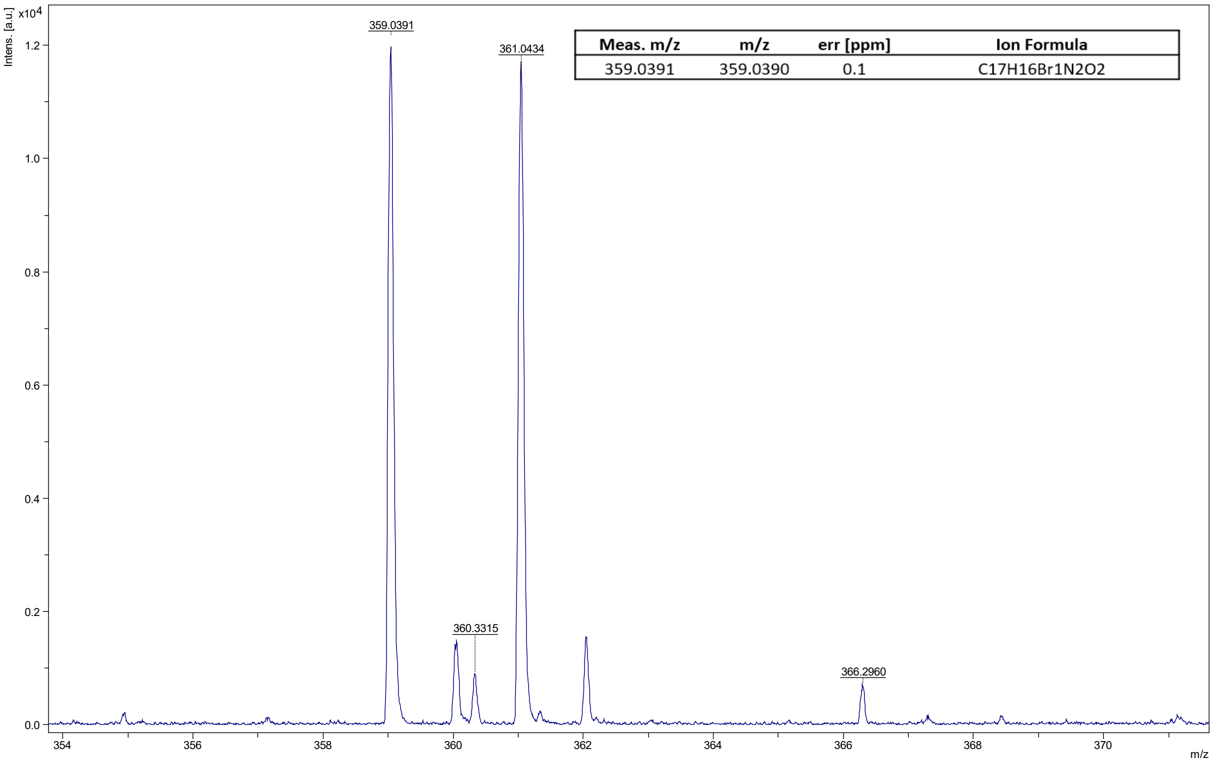

Meas. m/z	m/z	err [ppm]	Ion Formula
359.0391	359.0390	0.1	C17H16Br1N2O2

Figure S8. HRMS (MALDI, DCTB+) of AR43d.

Synthesis of *N*-(*N'*-Boc-piperidin-4-yl)-4-[2-(4-Boc-piperazin-1-yl)pyrimidin-5-yl]naphthalene-1,8-dicarboxylmonoimide (AR82p)

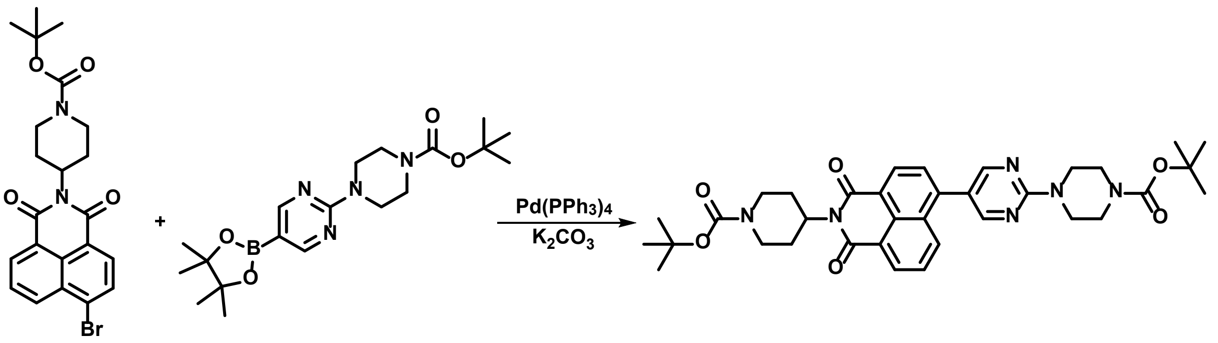

250 mg of *N*-(*N'*-Boc-piperidin-4-yl)-4-bromonaphthalene-1,8-dicarboxylmonoimide (AR43p, 0.54 mmol), 223 mg of 2-(4-Boc-piperazin-1-yl)pyrimidine-5-boronic acid pinacol ester (0.57 mmol) and 752 mg of potassium carbonate (5.44 mmol) were dissolved in a mixture of toluene:butanol:water (4:1:2 ml) under nitrogen atmosphere and the catalyst, $Pd(PPh_3)_4$ (5% mmol) was added. The resulting mixture was stirred overnight at 110ºC. After that, the solvent was evaporated under reduce pressure, the solid was dissolved in CH_2Cl_2 and washed with water (3×100 ml). The organic extracts were combined, dried over anhydrous Na_2SO_4 and the solvent was evaporated under reduce pressure. The organic solid was purified by column chromatography (SiO_2, DCM:MeOH 50:1) to obtain 280 mg of AR82p, yellow solid, 80% yield. m.p.: 226 – 229ºC (decomposition). IR (ATR, cm^{-1}): 3437 (N-H), 2977 – 2860 (C-H), 1699 (C=O), 1657 (C=O), 1585, 1513, 1450,1420, 1352, 1244, 1169 (C-N), 1106, 999, 948, 780, 759. ^{1}H-NMR (300 MHz, $CDCl_3$) δ (ppm): 8.57 (d, *J* = 7.6 Hz, 2H, 2×H_{Ar}), 8.44 (m, 2H, 2×H_{Ar}), 8.21 (d, *J* = 8.4 Hz, 1H, H_{Ar}), 7.70 (dd, *J* = 8.4, 7.3 Hz, 1H, H_{Ar}), 7.60 (d, *J* = 7.6 Hz, 1H, H_{Ar}), 5.16 (m, 1H, CH), 4.26 (s, 2H, CH_2), 3.89 (m, 4H, 2×CH_2), 3.53 (m, 4H, 2×CH_2), 2.72 (m, 4H, 2×CH_2), 1.65 (m, 2H, CH_2), 1.47 (m, 18H, 6×CH_3). ^{13}C-NMR (75MHz, $CDCl_3$) δ (ppm): 164.5 (C=O), 164.3 (C=O), 161.2 (C=O), 158.2 (C_{Ar}H), 140.7 (C_{Ar}), 132.3 (C_{Ar}), 132.2 (C_{Ar}), 132.1 (C_{Ar}), 131.6 (C_{Ar}H), 131.1 (C_{Ar}H), 130.1 (C_{Ar}), 128.7 (C_{Ar}H), 128.5 (C_{Ar}H), 127.7 (C_{Ar}H), 127.3 (C_{Ar}H), 123.6 (C_{Ar}), 121.0 (C_{Ar}), 80.3 (Cq), 79.6 (Cq), 52.0 (CH), 43.9 (CH_2), 28.6 (CH_3), 28.5 (CH_3), 28.4 (CH_2). HRMS (MALDI, DIT-) m/z: calculated for $C_{35}H_{42}N_6O_6$: 642.3160 (M^-); found 642.3181. UV-Vis ($CHCl_3$), λ_{max} nm (log ε): 380 (4.3). Ø (MCH, %): 92.87. τ (375 nm, MCH, ns): 2.853 (χ^2: 1.080).

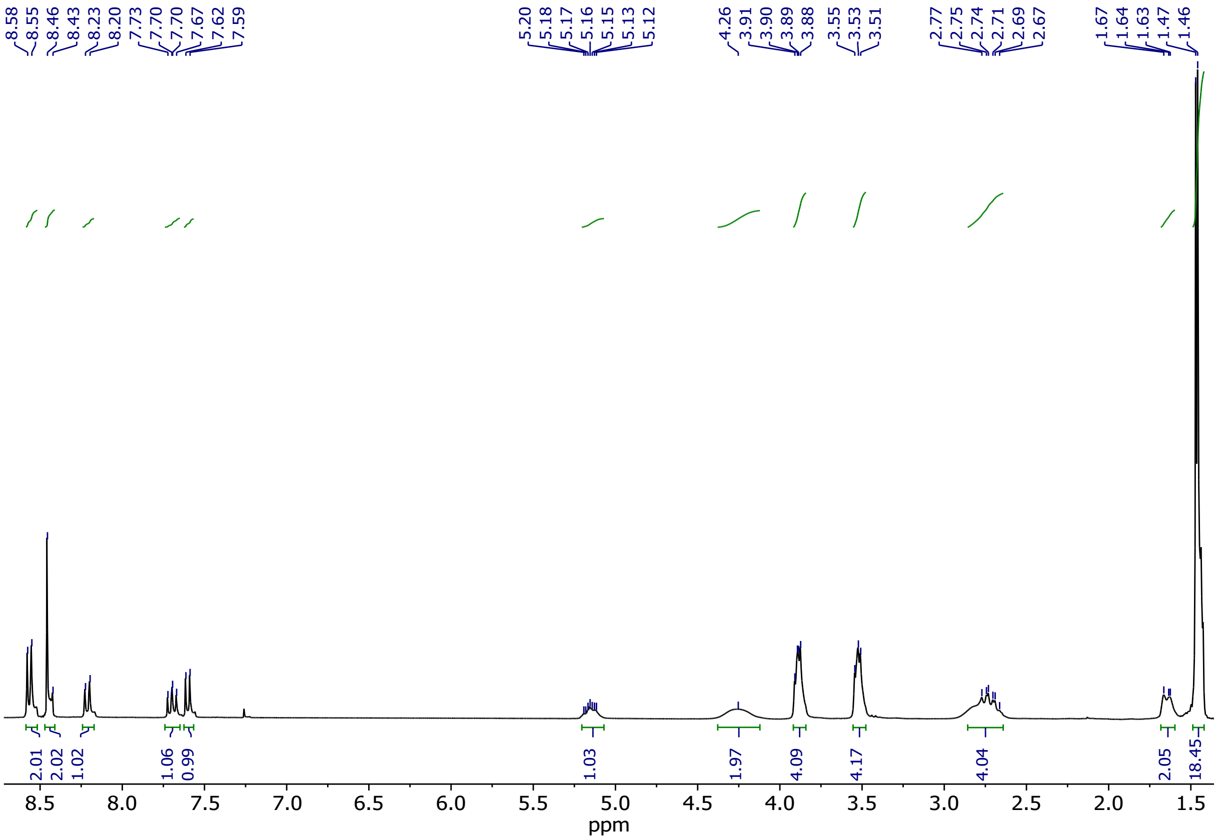

Figure S9. ^{1}H-NMR ($CDCl_3$, 300 MHz) of AR82p.

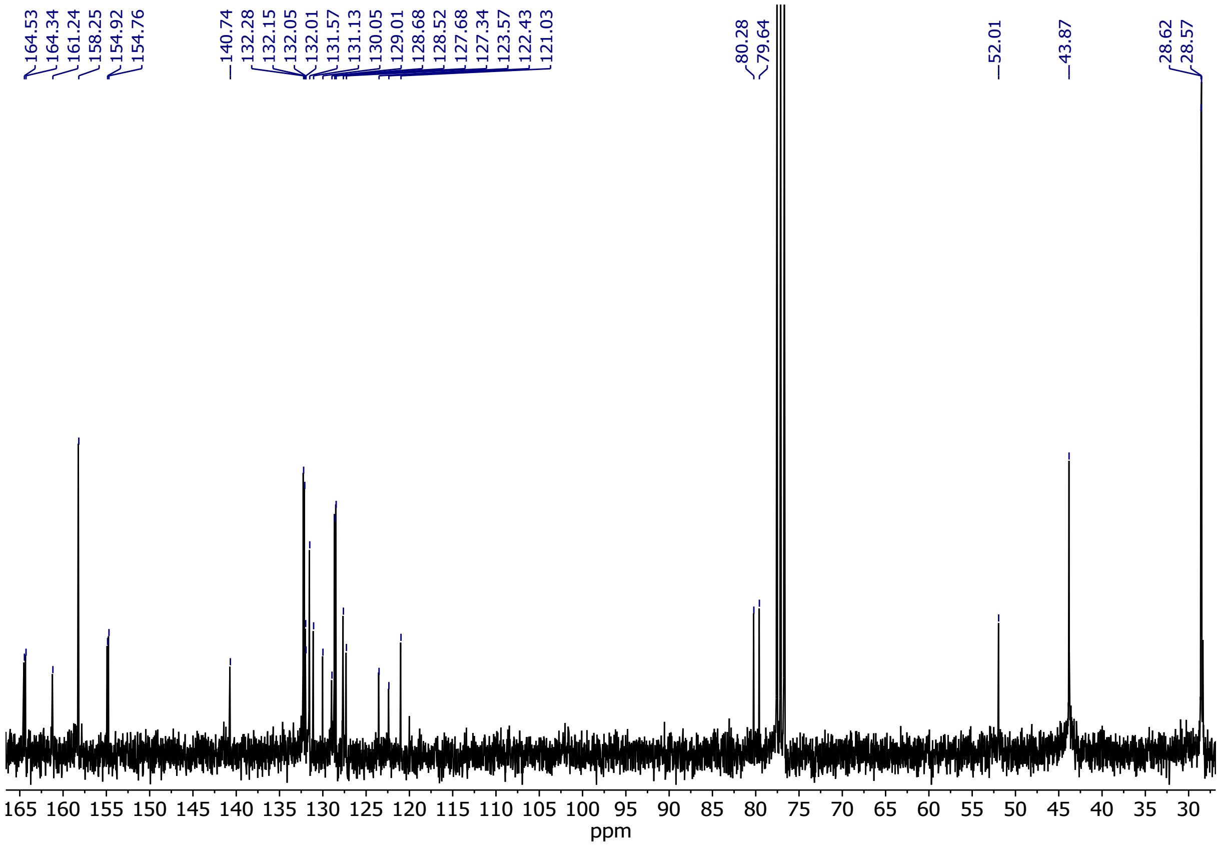

Figure 1. ^{13}C-NMR ($CDCl_3$, 75 MHz) of AR82p.

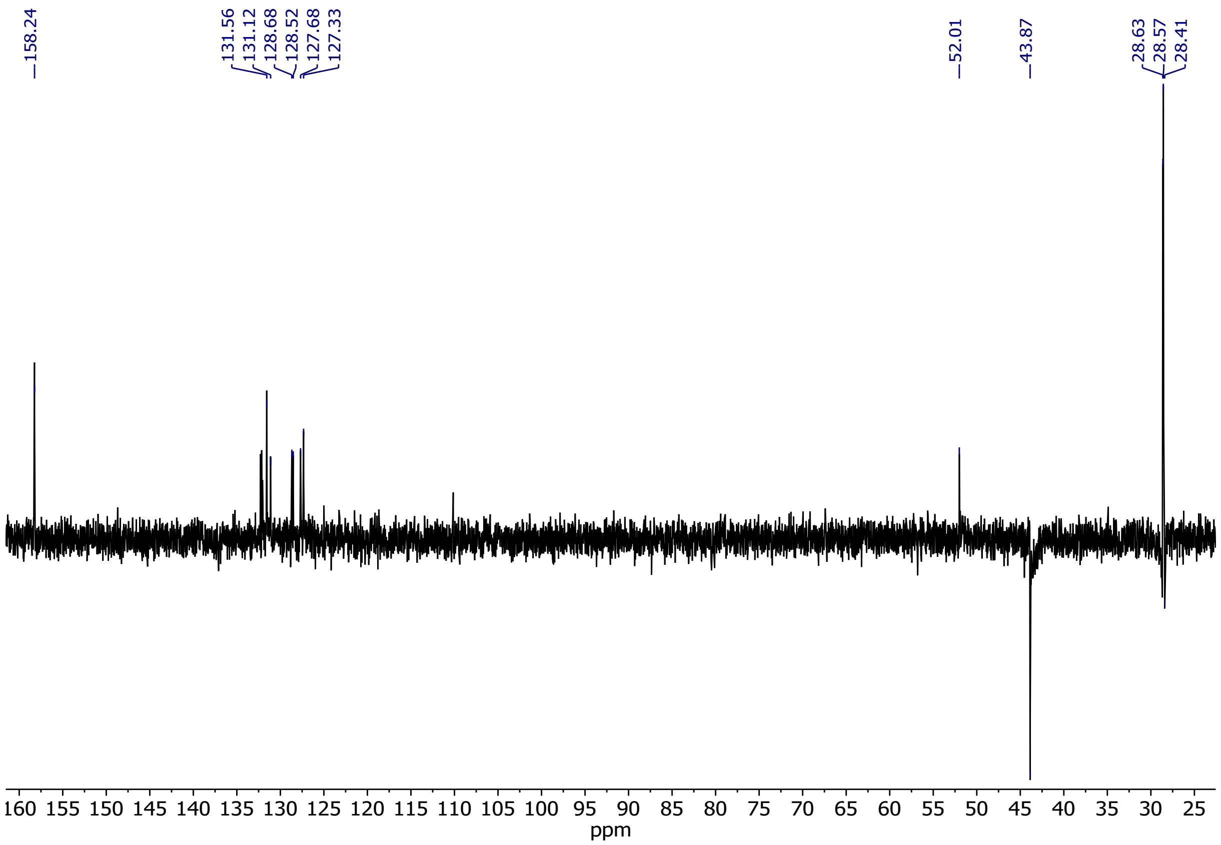

Figure S11. DEPT NMR ($CDCl_3$, 75 MHz) of AR82p.

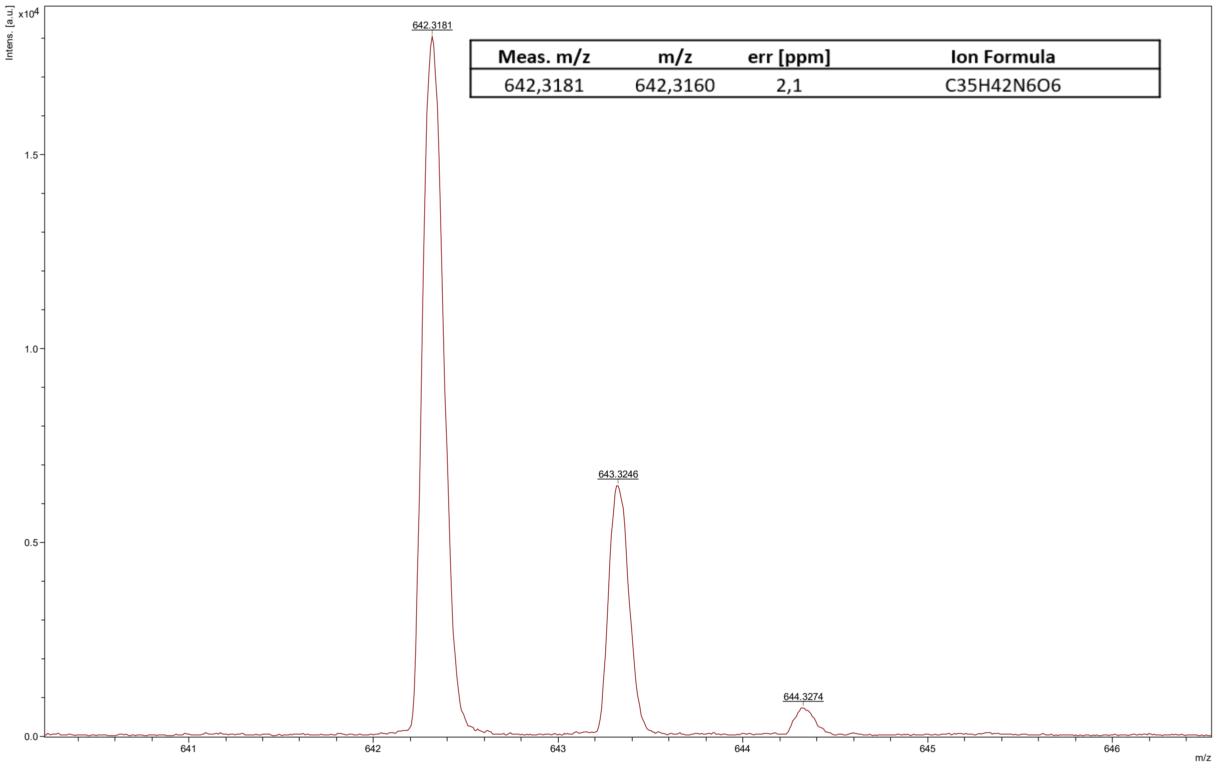

Figure S12. HRMS (MALDI, DIT-) of AR82p.

Solvatochromism:

The concentration of the compound was 20 µM and the excitation wavelength was 380 nm.

Figure S13. Solvatochromism of AR82p.

Absorbance

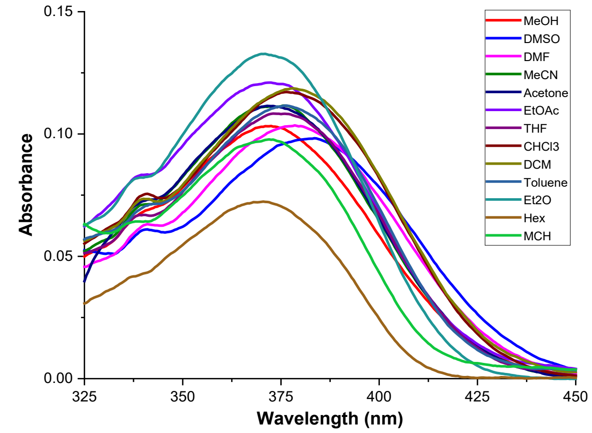

Normalized absorbance

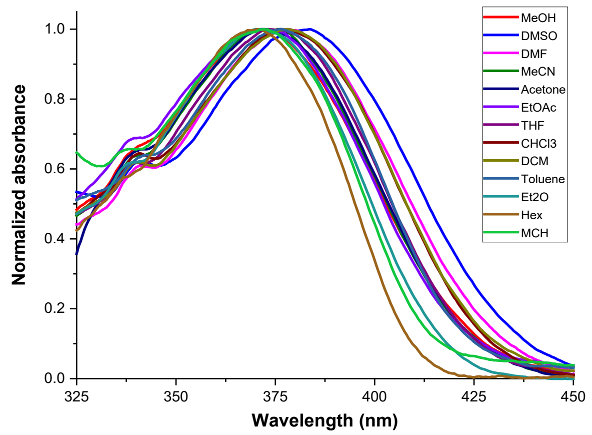

Emission

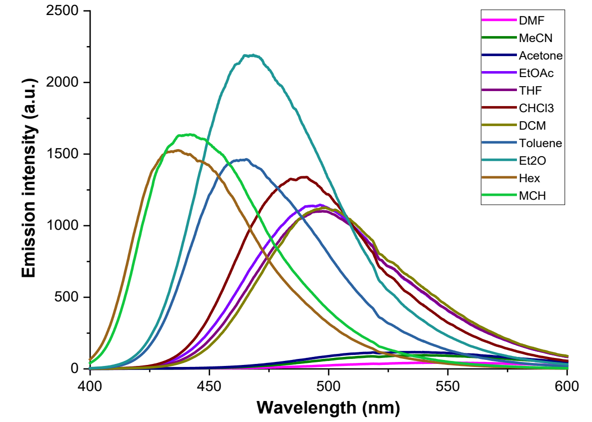

Normalized emission

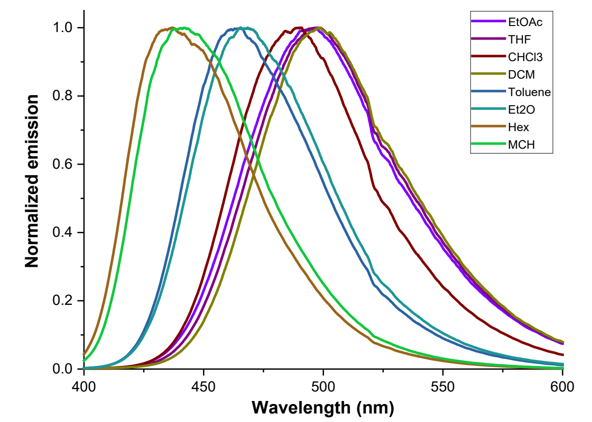

Figure S14. Solvatochromism of AR82p under visible and 366 nm light.

H₂O MeOH DMSO DMF MeCN Acet. EtOAc THF CHCl₃ DCM Tol. Et₂O Hex MCH

Visible light

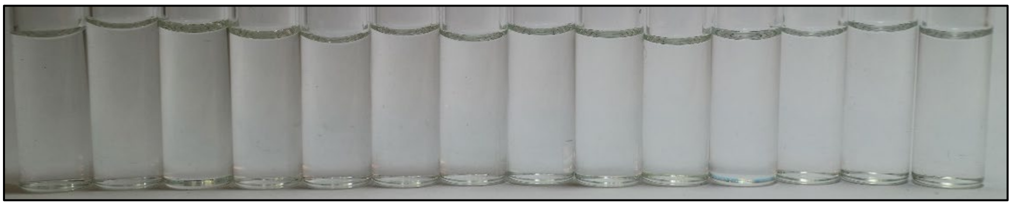

UV light (366 nm)

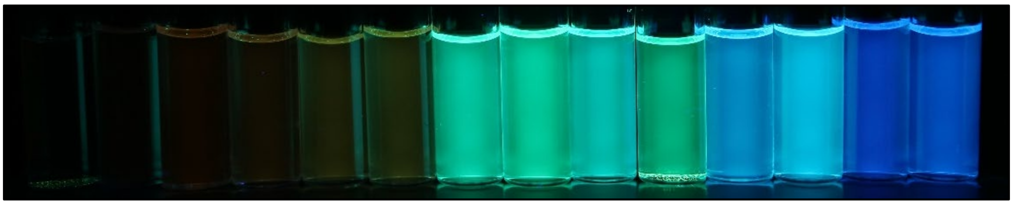

Fluorescence decay lifetime (τ):

The solvent used was MCH and the concentration of the compound was 20 μM.

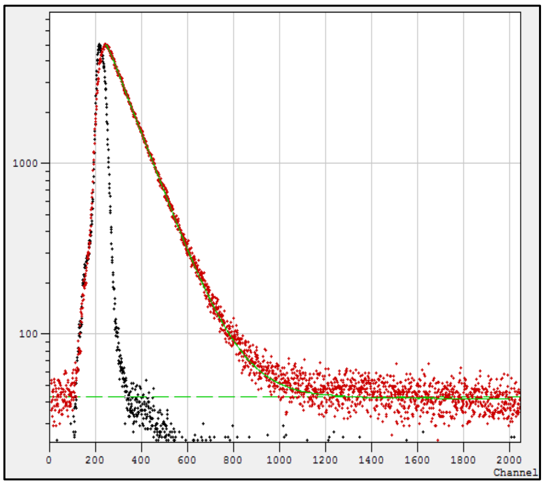

Figure S15. Fluorescence decay lifetime of AR82p.

Water acceptance test:

The solvent used was MeCN and THF and the concentration of the compound was 20 μM.

Figure 2. Water acceptance test of AR82p in MeCN under visible (up) and UV (down) light.

	R 5% 10% 20% 30% 40% 50% 60% 70% 80% 90%
**Visible light**	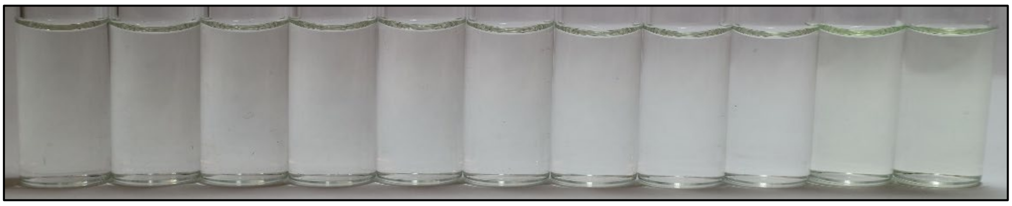
**UV light (366 nm)**	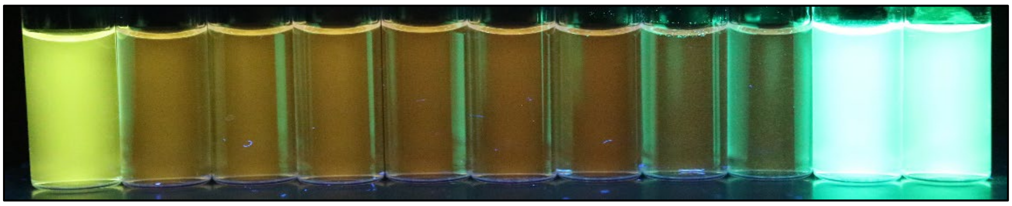

Figure S16. Water acceptance test of AR82p in THF under visible (up) and UV (down) light.

	R 5% 10% 20% 30% 40% 50% 60% 70% 80% 90%
**Visible light**	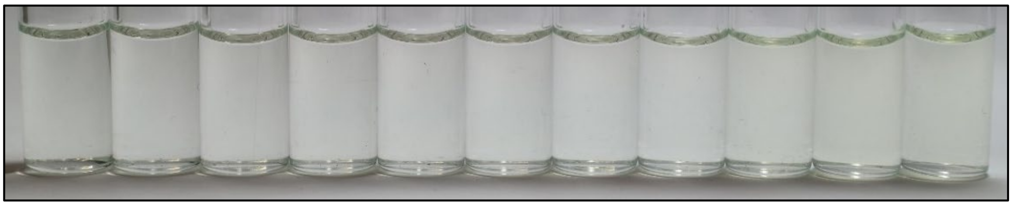
**UV light (366 nm)**	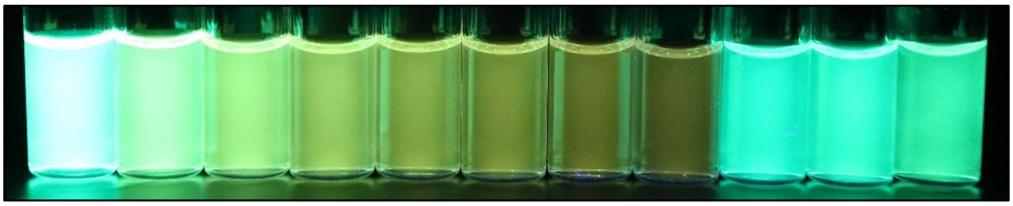

There was an increase in emission intensity from 80% water (AIE). This phenomenon was also studied using THF as solvent and the same amounts of water. In THF, an increase in the intensity of fluorescent emission was observed from 70% water. In both cases, there was a shift of the emission maximum from yellow-brown (535–545 nm) to green (about 475 nm).

Figure S17. Emission intensity graph (left) and graph of relationship between the emission intensity and the wavelength of the maximum of emission (right) as a function of the amount of water added for AR82p in THF.

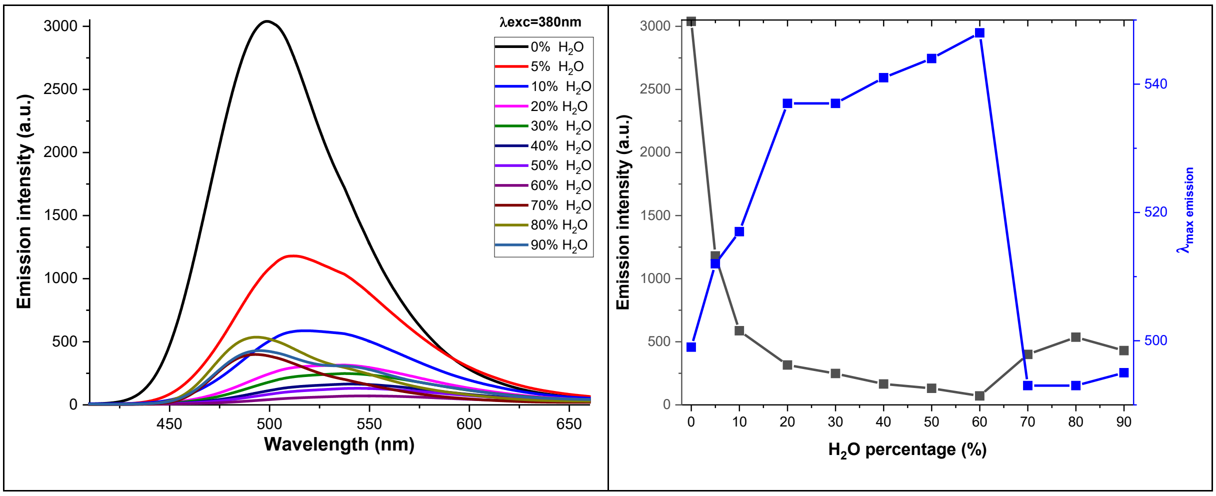

pH test:

The proportion of water that the compound could accept was 40%. The concentration of the compound was 20 μM and the solvent used was MeCN.

Figure S18. pH test of AR82p under visible (up) and UV (down) light.

	R HCl 3.4 4.8 6.4 7.4 7.9 10.5 KOH
**Visible light**	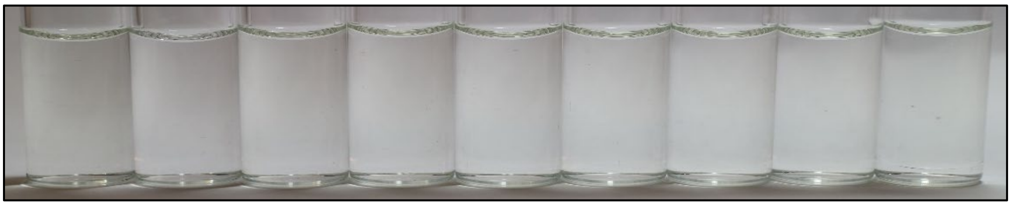
**UV light (366 nm)**	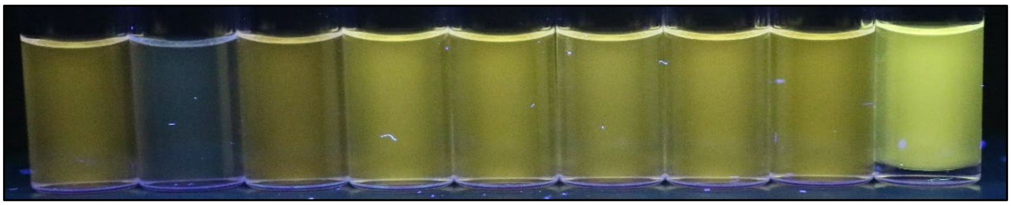

Quenching of emission at very acidic pH and yellow fluorescence at very basic pH were seen.

Cations and anions test:

The concentration of the compound was 20 μM and the solvent used was MeCN.

Figure S19. Cations test of AR82p under visible (up) and UV (down) light.

	R Ag^+ Ni^{2+} Sn^{2+} Cd^{2+} Zn^{2+} Pb^{2+} Cu^{2+} Fe^{3+} Sc^{3+} Al^{3+} Hg^{2+} Au^{3+} Co^{2+} Pd^{2+} Ir^{3+} Cu^+ Ru^{3+} Pt^{2+}
**Visible light**	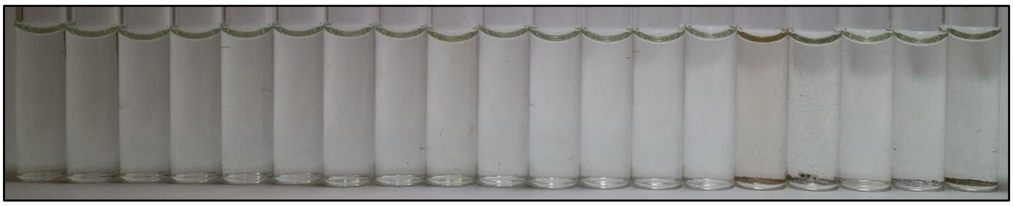

**UV light (366 nm)**	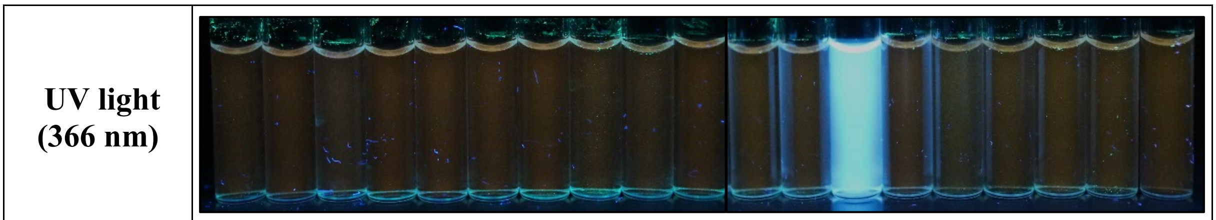

Under visible light, a response to Pd^{2+} was observed, while under UV light, a fluorescence change to blue was taken place to Au^{3+}.

The concentration of the compound was 20 µM and the solvent used was MeCN.

Figure S20. Anions test of AR82p under visible (up) and UV (down) light.

	R F$^-$ Cl$^-$ Br$^-$ I$^-$ BzO$^-$ NO_3^- $H_2PO_4^-$ HSO_4^- AcO$^-$ CN$^-$ SCN$^-$
**Visible light**	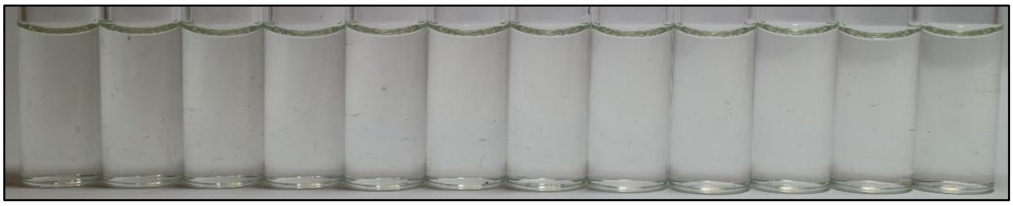
**UV light (366 nm)**	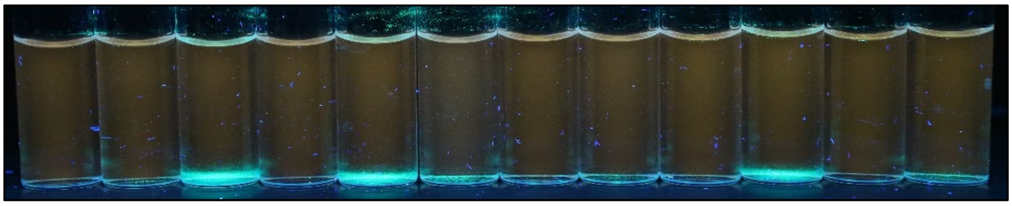

No significant changes were observed.

Oxidants and reductants test:

The solvent used was MeCN and concentration of the compound was 20 µM.

Figure S21. Oxidants and reductants test of AR82p under visible (up) and UV (down) light.

	R HCl* HNO_3 m-CPBA Oxn Hz H_2O_2 TNB TNT TATP
**Visible light**	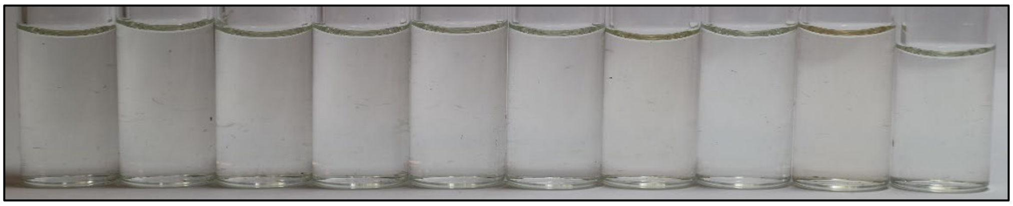
**UV light (366 nm)**	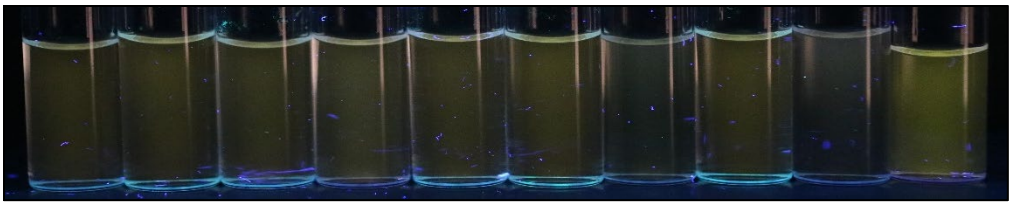

*HCl is included in all experiments in a close position to HNO_3 to distinguish between the redox action of HNO_3 and a possible effect due only to the concomitant acidity of nitric acid.

No significant changes were observed.

Synthesis of *N*-(piperidin-4-yl)-4-[2-(4-Boc-piperazin-1-yl)pyrimidin-5-yl]naphthalene-1,8-dicarboxylmonoimide (AR82s)

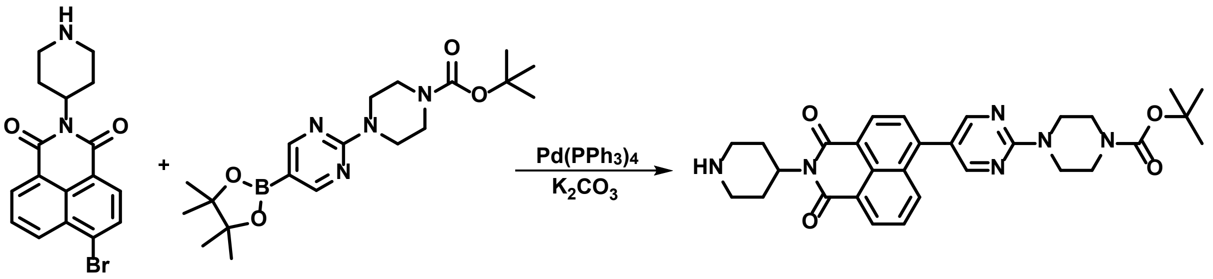

150 mg of *N*-(piperidin-4-yl)-4-bromonaphthalene-1,8-dicarboxylmonoimide (AR43d, 0.42 mmol), 172 mg of 2-(4-Boc-piperazin-1-yl)pyrimidin-5-yl boronic acid pinacol ester (0.44 mmol) and 451 mg of potassium carbonate (3.26 mmol) were dissolved in a mixture of toluene:butanol:water (4:1:2 ml) under nitrogen atmosphere and, then, the $Pd(PPh_3)_4$ (5% mmol) was added. The resulting mixture was stirred overnight at 110ºC, the solvent was evaporated under reduce pressure. The solid was dissolved in CH_2Cl_2 and washed with water (3×100 ml). The organic extracts were combined, dried over anhydrous Na_2SO_4 and the solvent was evaporated under reduce pressure. The organic solid was purified by column chromatography (SiO_2, CH_2Cl_2:MeOH 50:1) getting 87 mg of AR82s, yellow solid, 38% yield. m.p.: 302 – 304ºC (decomposition). IR (ATR, cm^{-1}): 3440 (N-H), 2922 – 2480, 1693 (C=O), 1650 (C=O), 1585, 1509, 1450 (C-O), 1402, 1351, 1238, 1162 (C-N), 1121, 998, 947, 780, 753, 687, 642, 508. ^1H-NMR (300 MHz, $CDCl_3$) δ (ppm): 8.62 (dd, *J* = 7.4, 1.7 Hz, 2H, 2×H_{Ar}), 8.50 (s, 2H, 2×H_{Ar}), 8.25 (dd, *J* = 8.5, 1.1 Hz, 1H, H_{Ar}), 7.73 (dd, *J* = 8.5, 7.4 Hz, 1H, H_{Ar}), 7.64 (d, *J* = 7.4 Hz, 1H, H_{Ar}), 5.26 – 5.13 (m, 1H, CH), 3.93 (m, 4H, 2×CH_2), 3.56 (m, 4H, 2×CH_2), 3.29 (m, 2H, CH_2), 2.89 – 2.65 (m, 4H, 2×CH_2), 2.13 (s, 1H, NH), 1.73 (m, 2H, CH_2), 1.51 (s, 9H, 3×CH_3). ^{13}C-NMR (75 MHz, $CDCl_3$) δ (ppm): 164.5 (C=O), 164.3 (C=O), 164.1 (C=O), 158.2 ($C_{Ar}H$), 154.9 (C_{Ar}), 140.7 (C_{Ar}), 132.1 (C_{Ar}), 131.9 ($C_{Ar}H$), 131.8 (C_{Ar}), 131.7 (C_{Ar}), 131.5 (C_{Ar}), 131.4 (C_{Ar}), 130.1 (C_{Ar}), 129.0 (C_{Ar}), 127.8 ($C_{Ar}H$), 127.5 ($C_{Ar}H$), 123.3 (C_{Ar}), 122.2 (C_{Ar}), 121.0 (C_{Ar}), 80.4 (Cq), 44.1 (CH_2), 28.6 (CH_3), 25.8 (CH_2). HRMS (MALDI, DCTB+) m/z: calculated for $C_{30}H_{34}N_6O_4$: 543.2714 (M^+ + H); found 543.2689. UV-Vis ($CHCl_3$), λ_{max} nm (log ε): 385 (4.9). Ø ($CHCl_3$, %): 73.00. τ (375 nm, $CHCl_3$, ns): 3.962 (χ^2: 1.072).

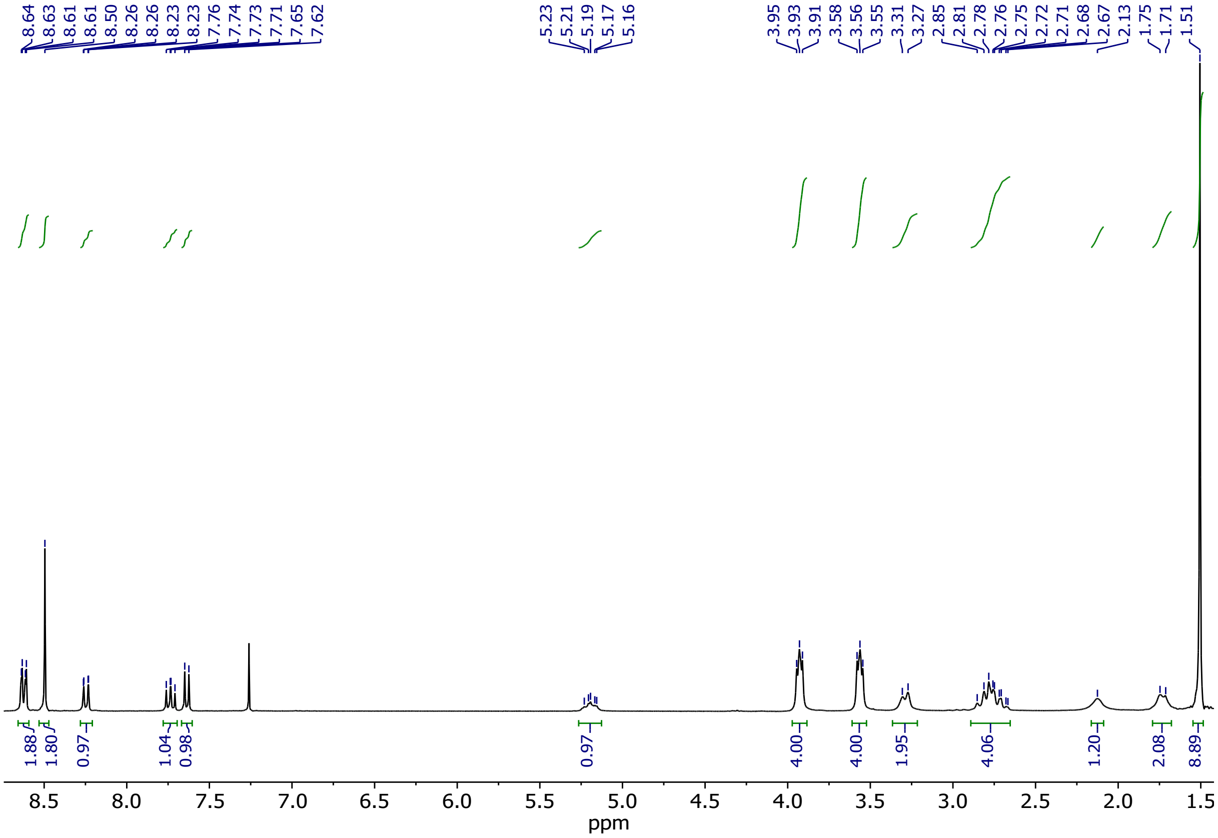

Figure S22. ^{1}H-NMR (CDCl$_3$, 300 MHz) of AR82s.

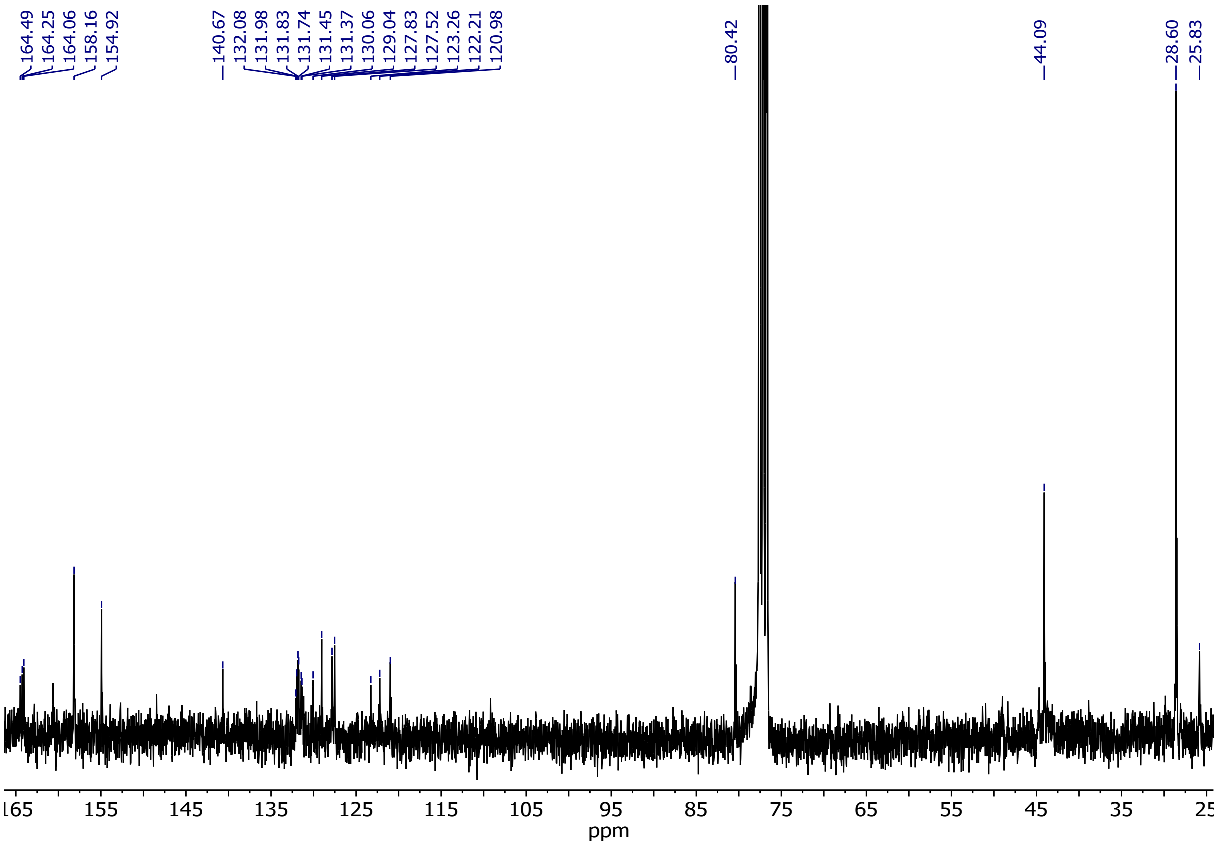

Figure S23. ^{13}C-NMR (CDCl$_3$, 75 MHz) of AR82s.

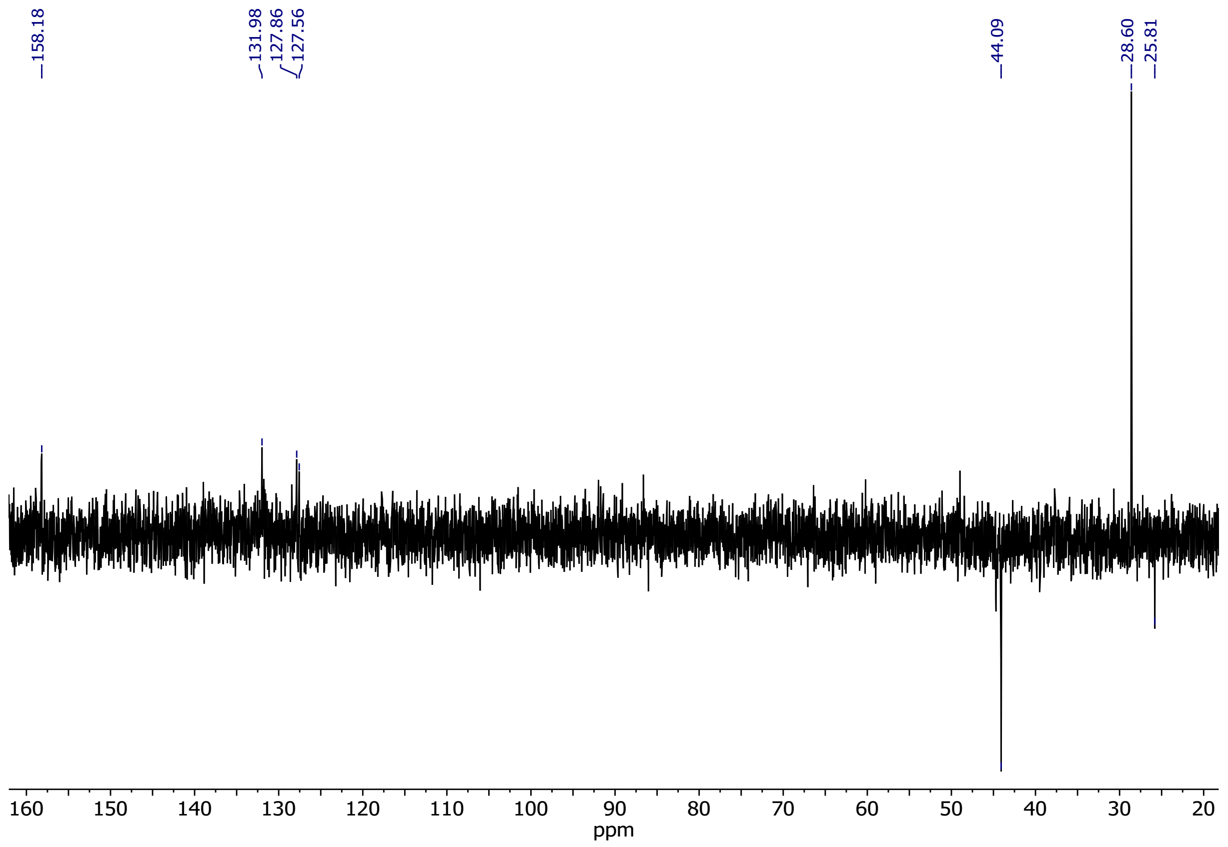

Figure S24. DEPT NMR ($CDCl_3$, 75 MHz) of AR82s.

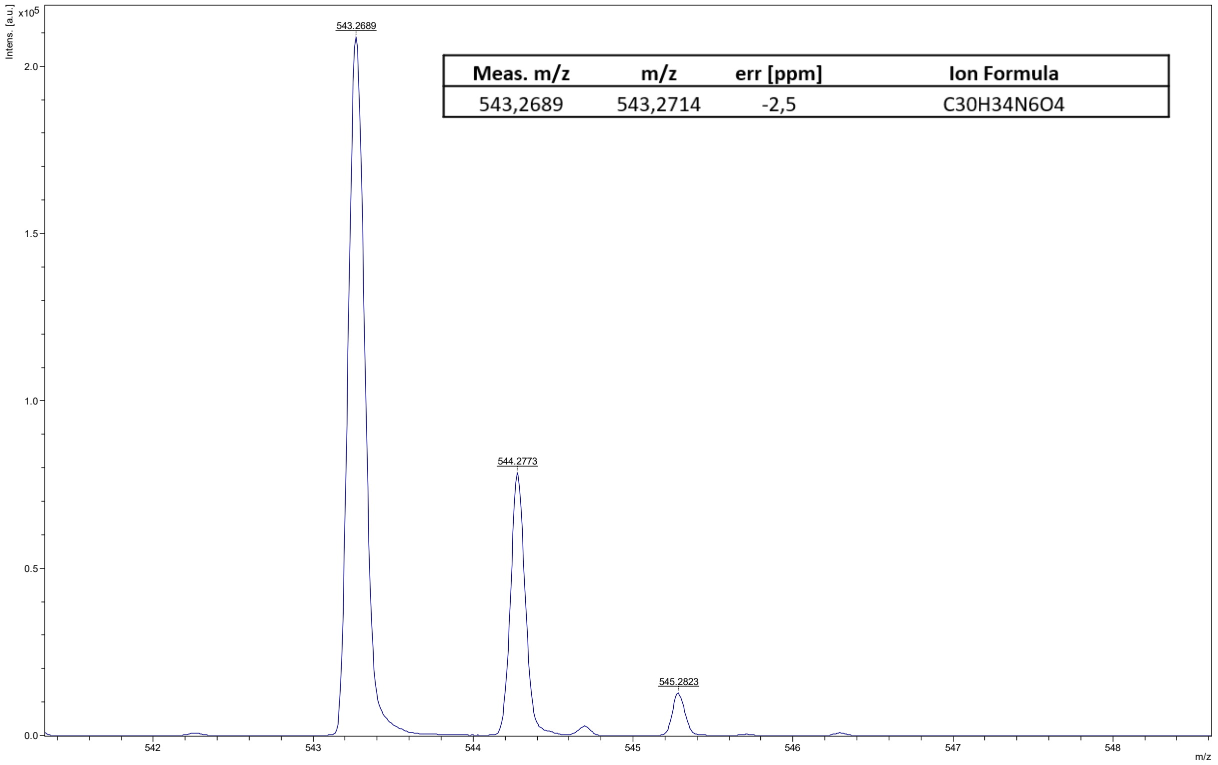

Meas. m/z	m/z	err [ppm]	Ion Formula
543,2689	543,2714	-2,5	C30H34N6O4

Figure S25. HRMS (MALDI, DCTB+) of AR82s.

Solvatochromism:

The concentration of the compound was 20 μM and the excitation wavelength was 370 nm.

Figure S26. Solvatochromism of AR82s.

Absorbance

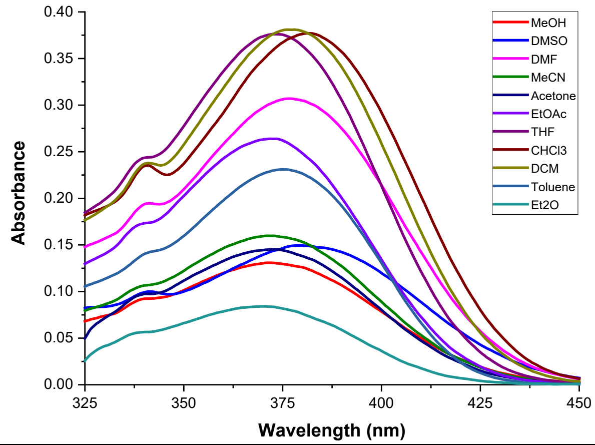

Normalized absorbance

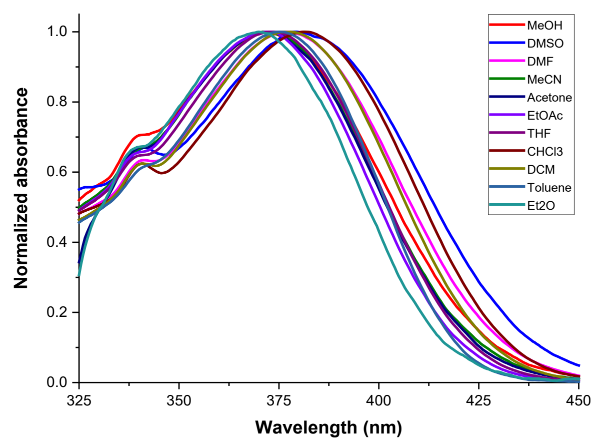

Emission

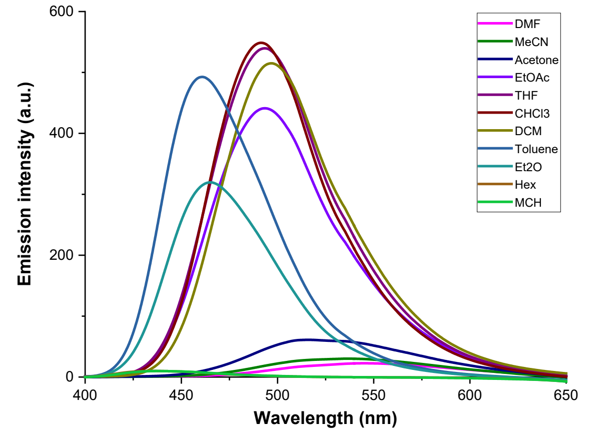

Normalized emission

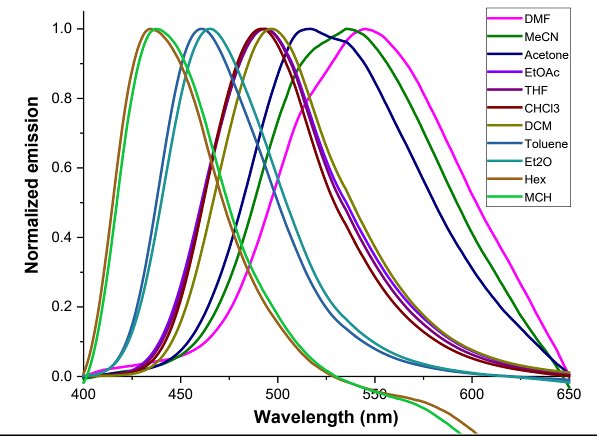

Figure S27. Solvatochromism of AR82s under visible and 366 nm light.

	H_2O MeOH DMSO DMF MeCN Acet. EtOAc THF $CHCl_3$ DCM Tol. Et_2O Hex MCH
**Visible light**	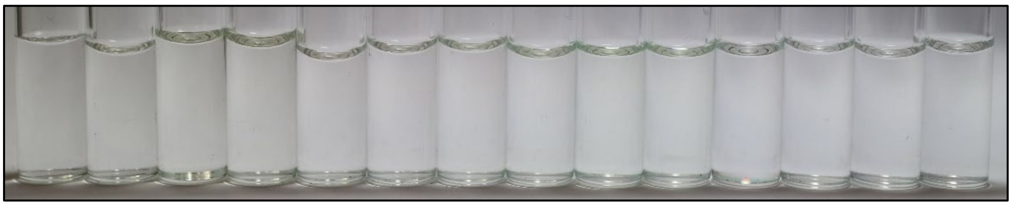
**UV light (366 nm)**	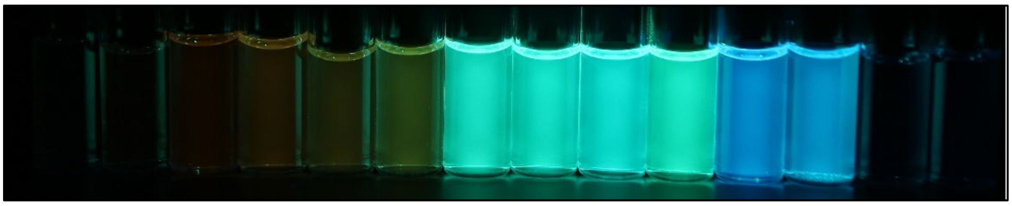

Fluorescence decay lifetime (τ):

The solvent used was $CHCl_3$. The concentration of the compound was 20 µM.

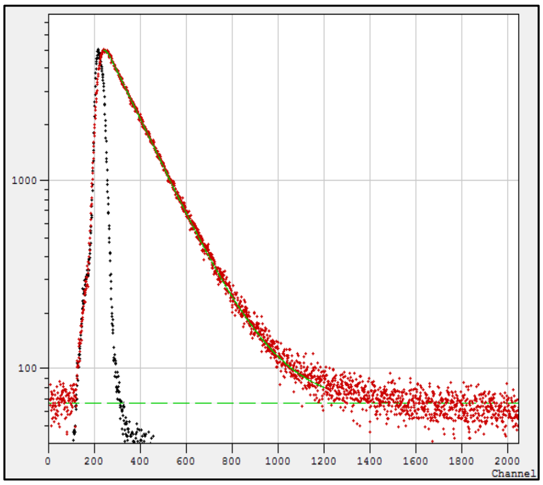

Figure S28. Fluorescence decay lifetime of AR82s.

Water acceptance test:

The solvent used was acetone and the concentration of the compound was 20 µM.

Figure S29. Water acceptance test of AR82s, acetone under visible (up) and UV (down) light.

	R 5% 10% 20% 30% 40% 50% 60% 70% 80% 90%
**Visible light**	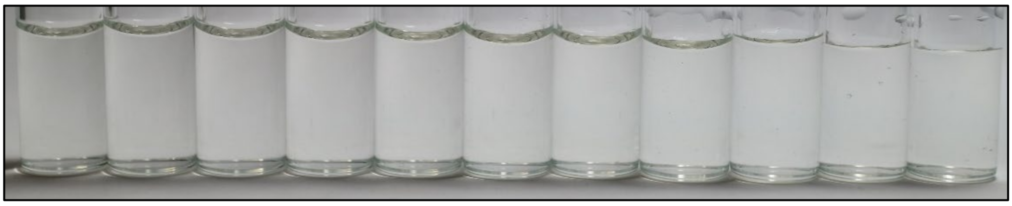
**UV light (366 nm)**	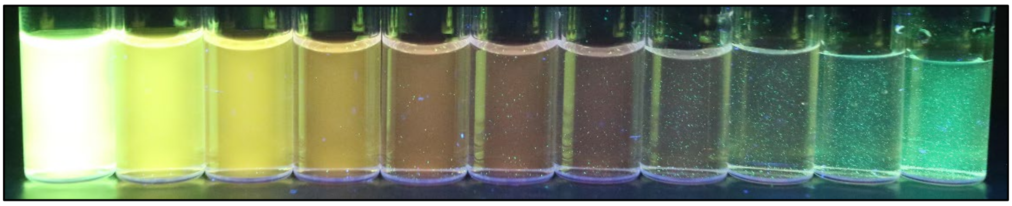

Figure S30. Water aceptance test of AR82s in THF under visible (up) and UV (down) light.

	R 5% 10% 20% 30% 40% 50% 60% 70% 80% 90%
**Visible light**	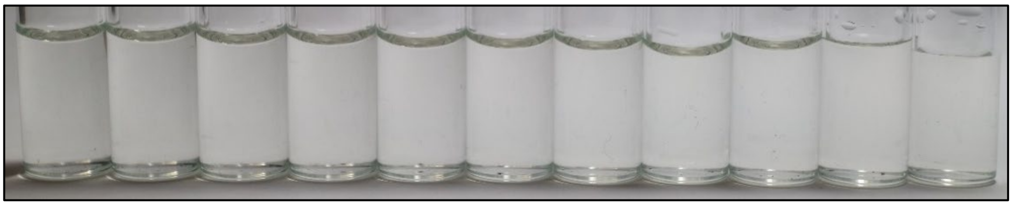
**UV light (366 nm)**	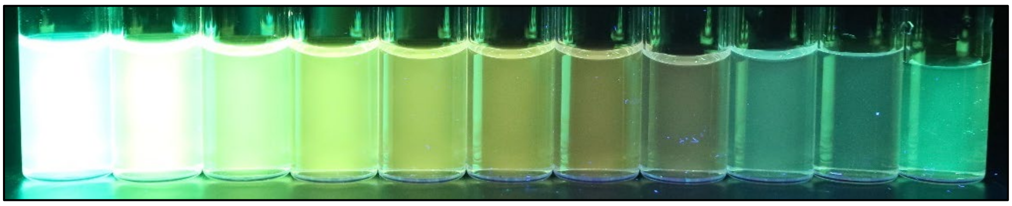

There was a very slight increase in emission intensity at 80% and 90% of water (AIE) and the result using THF as a solvent was very similar. The emission maximum was shifted from brown – yellow region (530 – 555 nm), with intermediate amounts of water, to the green region (490 – 505 nm), with high water content.

Figure S31. Emission intensity graph (left) and graph of relationship between the emission intensity and the wavelength of the maximum of emission (right) as a function of the amount of water added for AR82s using THF as a solvent.

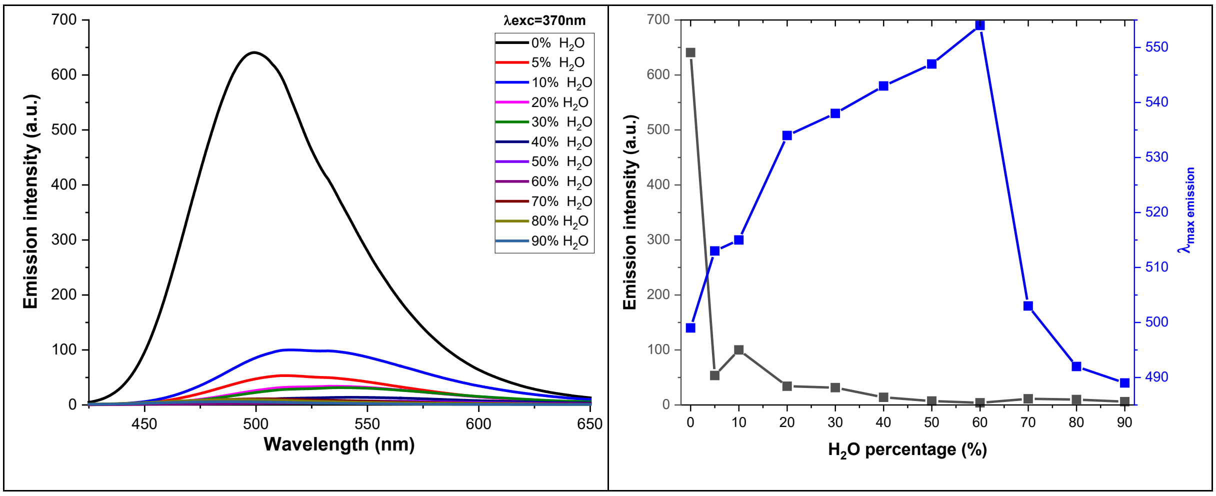

pH test:

The amount of water that the compound could accept was 20%. The concentration of the compound was 20 µM and the solvent used was acetone.

Figure S32. pH test of AR82s under visible (up) and UV (down) light.

	R HCl 3.4 4.8 6.4 7.4 7.9 10.5 KOH
**Visible light**	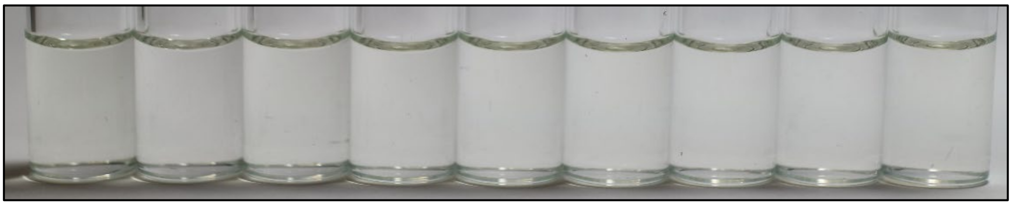
**UV light (366 nm)**	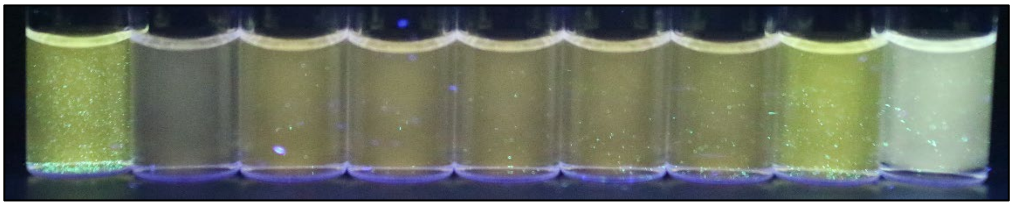

The most significative change was the quenching of the emission at very acidic pH and the fluorescence color change to white at very basic pH. For intermediate pH, the fluorescence was turned brownish.

Cations and anions test:

The concentration of the compound was 20 µM and the solvent used was acetone.

Figure S33. Cations test of AR82s under visible (up) and UV (down) light.

	R Ag^{+} Ni^{2+} Sn^{2+} Cd^{2+} Zn^{2+} Pb^{2+} Cu^{2+} Fe^{3+} Sc^{3+} Al^{3+} Hg^{2+} Au^{3+} Co^{2+} Pd^{2+} Ir^{3+} Cu^{+} Ru^{3+} Pt^{2+}
**Visible light**	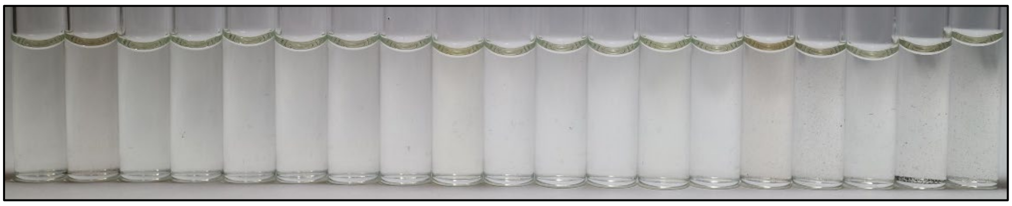
**UV light (366 nm)**	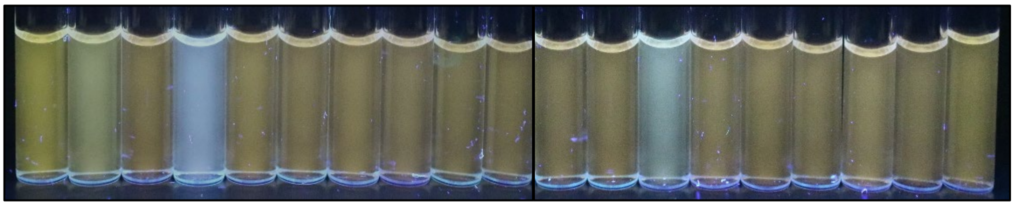

The most significant changes under UV light were the turn of the fluorescence to white against Sn^{2+} and Au^{3+}.

The concentration of the compound was 20 µM and the solvent used was acetone.

Figure S34. Anions test of AR82s under visible (up) and UV (down) light.

	R F^{-} Cl^{-} Br^{-} I^{-} BzO^{-} NO_3^{-} $H_2PO_4^{-}$ HSO_4^{-} AcO^{-} CN^{-} SCN^{-}
**Visible light**	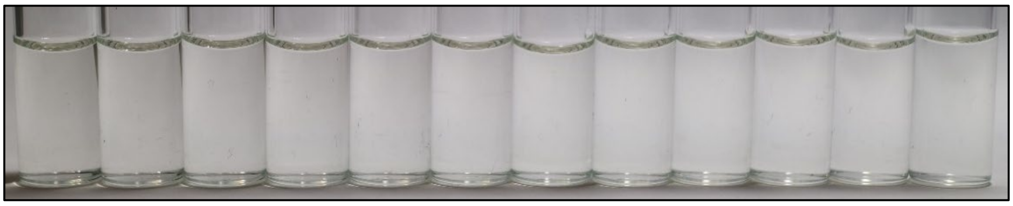
**UV light (366 nm)**	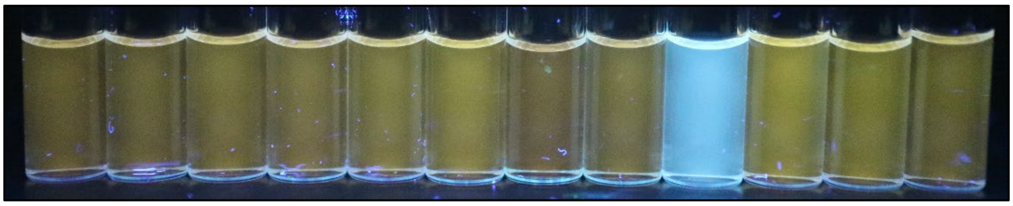

The only response of the probe to the different anionic analytes was against HSO_4^{-}.

Oxidants and reductants test:

The solvent used was acetone and concentration of the compound was 20 µM.

Figure S35. Oxidants and reductants test of AR82s under visible (up) and UV (down) light.

	R HCl* HNO_3 Oxn Hz H_2O_2 R m-CPBA TNB TNT R m-CPBA TATP HMTD
**Visible light**	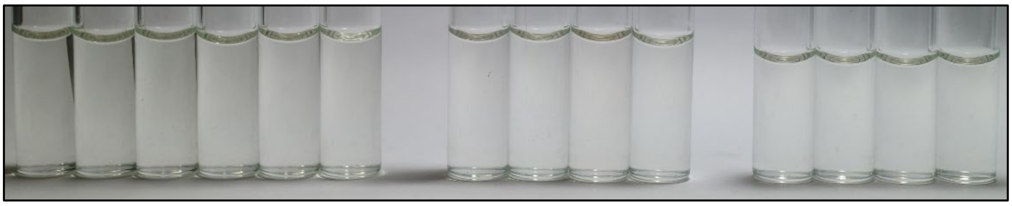

**UV light (366 nm)**	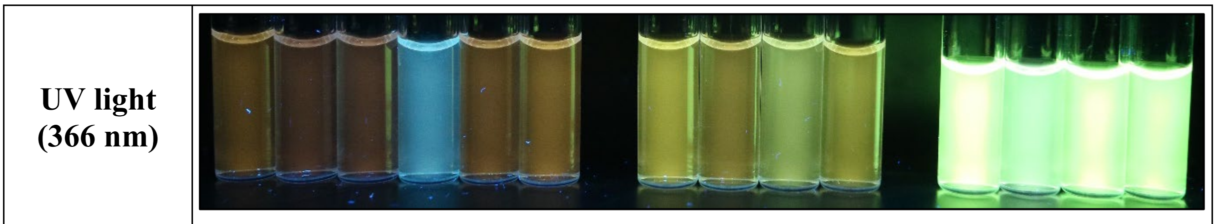

*HCl is included in all experiments in a close position to HNO_3 to distinguish between the redox action of HNO_3 and a possible effect due only to the concomitant acidity of nitric acid.

The most significant change was the turn of the fluorescence to blue against oxone.

Preliminary solvents test:

In order to determine the fluorescence variation of the compound under study in the presence of TATP, some tests were carried out for the probe in 9 different solvents. The AR82s concentration was 20 µM and the TATP was added in excess (7 mg in each vial). All the tests were performed at room temperature and the photographs were taken immediately after the addition of TATP and again after 24 hours.

Figure S36. Pairs of AR82s (left) and AR82s with excess of TATP (right) in different solvents at time 0 h (up) and 24 h (down).

	DMF MeCN EtOAc THF $CHCl_3$ DCM Tol $CHCl_3$:MeOH 9:1 DCM:MeOH 9:1
**t = 0 h**	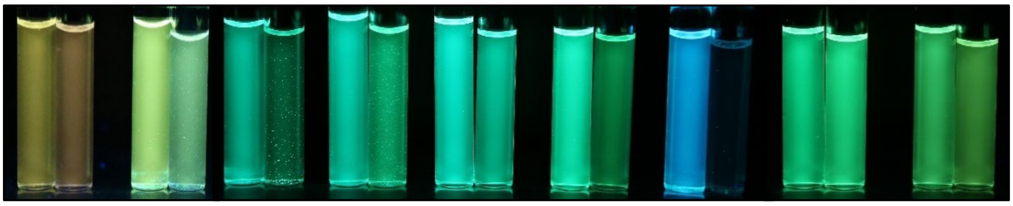
**t = 24 h**	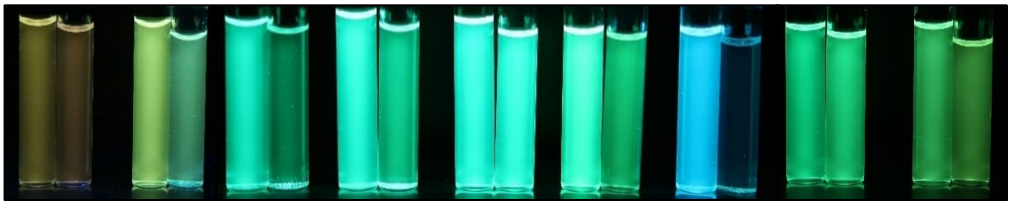

The biggest changes were seen in DMF and MeCN with a fluorescence color change and in toluene and DCM with a decrease in the emission intensity.

Work concentration test:

In order to choose an optimum concentration for the next quantitative studio, the absorbance and fluorescence of the probe was checked to be linear while concentration changes was small, even when working with an excess of m-CPBA (7000 µM). The solvent used for this test was DCM and the excitation wavelength was 370 nm for all measurements.

Figure S37. Absorbance spectrum (up left), absorbance profile at 385 nm (up right), fluorescence spectrum (down left) and fluorescence profile at 497 nm (down right) of increasing concentrations of AR82s solution in DCM.

DCM

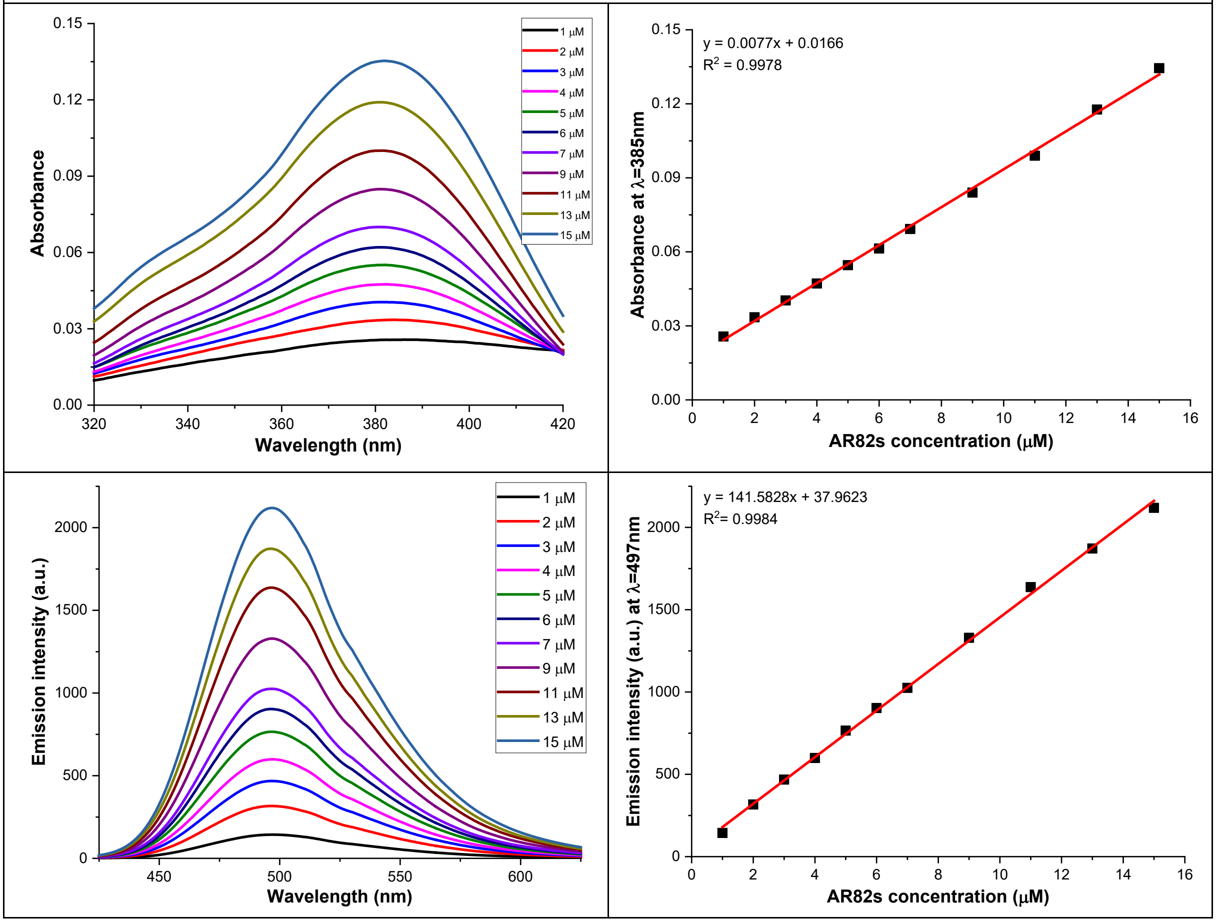

Figure S38. Absorbance spectrum (up left), absorbance profile at 385 nm (up right), fluorescence spectrum (down left) and fluorescence profile at 507 nm (down right) of increasing concentrations of AR82s with an excess of m-CPBA solution in DCM.

DCM with m-CPBA

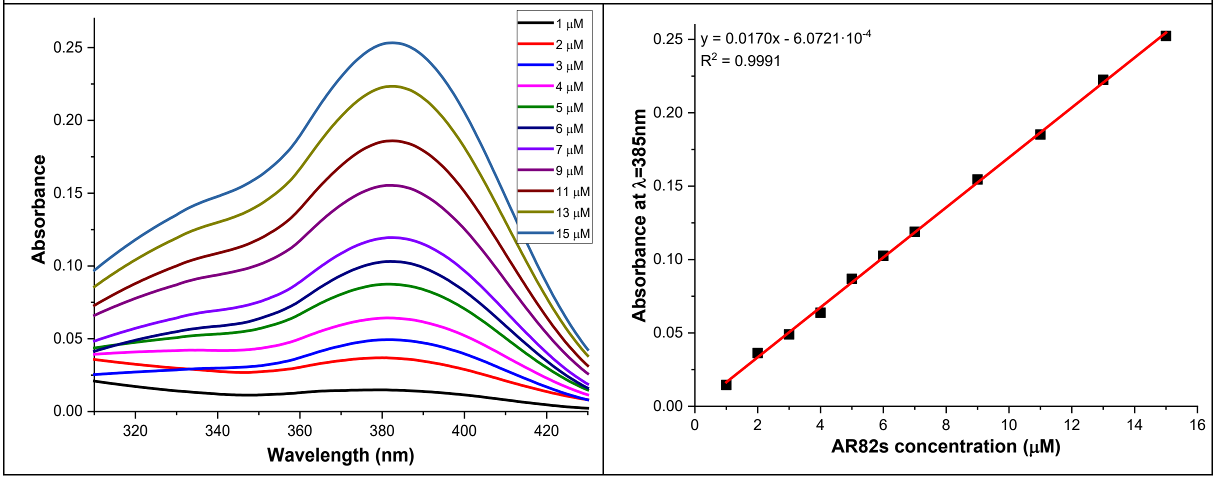

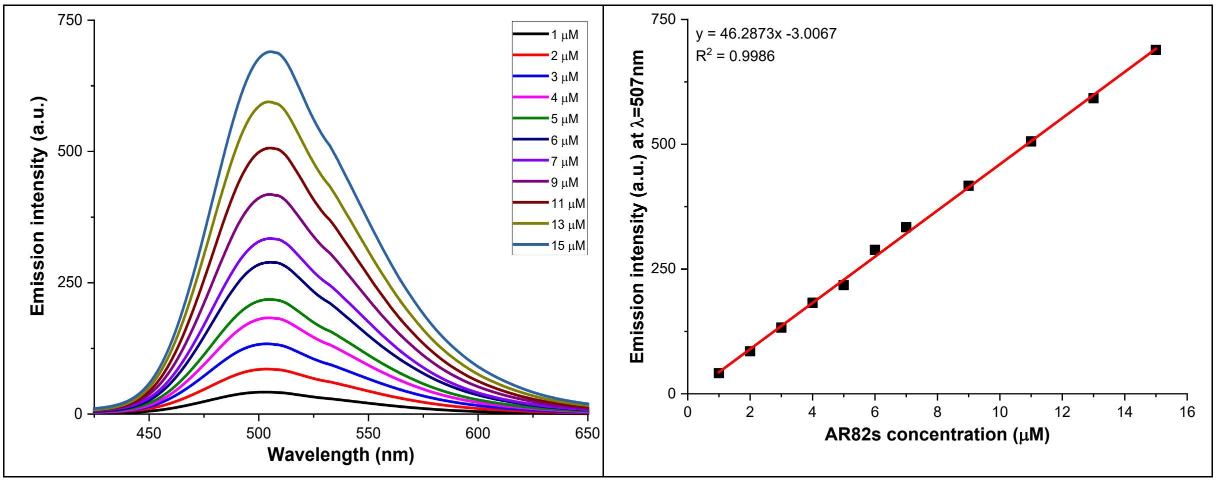

The ideal working concentration should stay at 0.1 of absorbance or less, to avoid inner filter effects, possible dynamic quenching or stacking processes. It was implied a concentration below 11 μM. Taking the results into account, the chosen concentration was 2.5 μM, value around which the Lambert – Beer Law (or pseudo – Lambert – Beer linear behavior for fluorescence) was fulfilled.

Solvents test with TATP:

For all solvents, the working concentration of AR82s was 2.5 μM, the excitation wavelength was 370 nm and the temperature for all test was 25ºC.

In the kinetic studies, TATP was in excess (20 mM) and the fluorescence emission measurements were made during 25 minutes in the case of DMF and 60 minutes in MeCN, toluene and DCM at 5 minutes time intervals. In the titration, the TATP concentration was between 0 and 35000 μM and it was added directly as solid. The measurements were carried out immediately after the addition of the probe.

Figure S39. Study of AR82s with TATP in DMF. Kinetic study (up left) and profile as function as time at 415 nm and 550 nm simultaneously (up right) in presence of TATP excess. Titration (down left) and fluorescence profile at 550 nm (down right) under increasing concentrations of TATP.

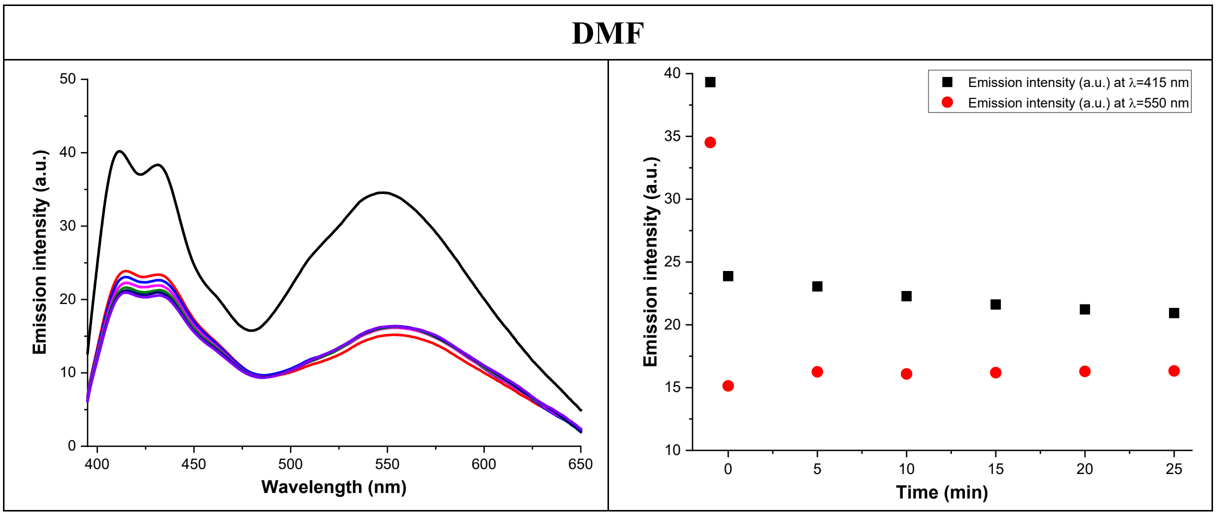

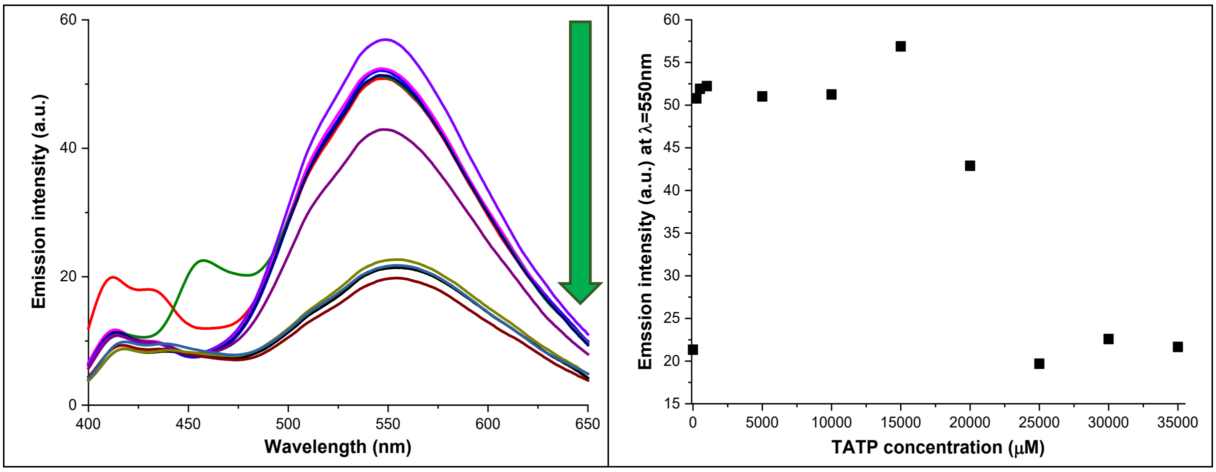

There was a drop in the emission intensity band immediately after adding TATP. According to the fluorescence spectra, the LOD of TATP was somewhere between 15000 and 20000 μM, too high for a good probe.

Figure S40. Kinetic study at different times under visible (up) and UV (down) light in DMF. In each photo, left vial was contained the probe and right vial was contained the probe and an excess of TATP.

	t=0	t=30 min	t=24 h
**Visible light**	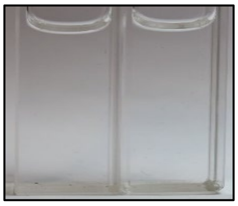	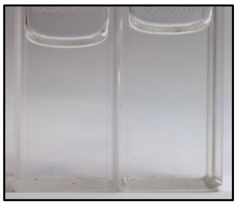	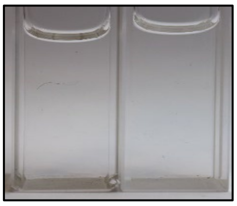
**UV light (366 nm)**	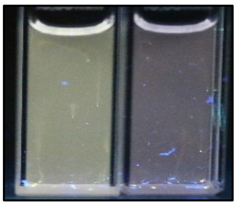	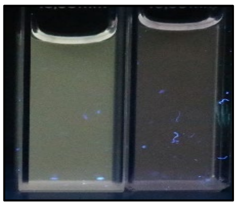	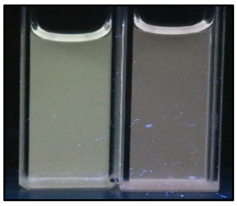

Figure S41. Titration of AR82s with an excess of TATP under visible (up) and UV (down) light in DMF.

μM	**0 250 500 1000 5000 10000 15000 20000 25000 30000 35000**
**Visible light**	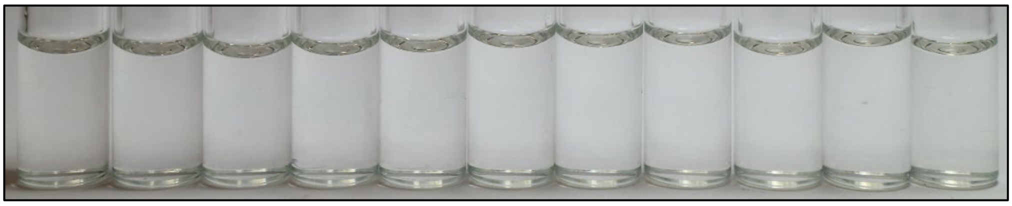
**UV light (366 nm)**	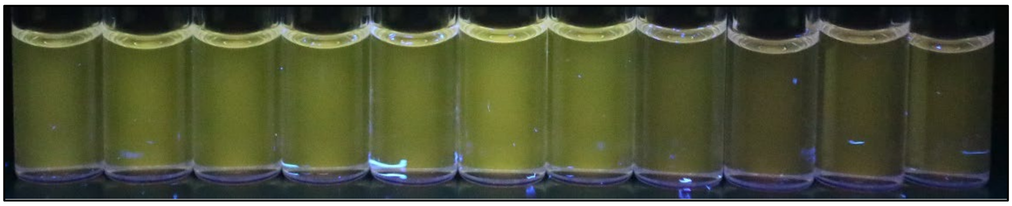

Figure S42. Study of AR82s with TATP in MeCN. Kinetic study (up left) and profile as function as time at 535 nm (up right) in presence of TATP excess. Titration (down left) and fluorescence profile at 530 nm (down right) under increasing concentrations of TATP.

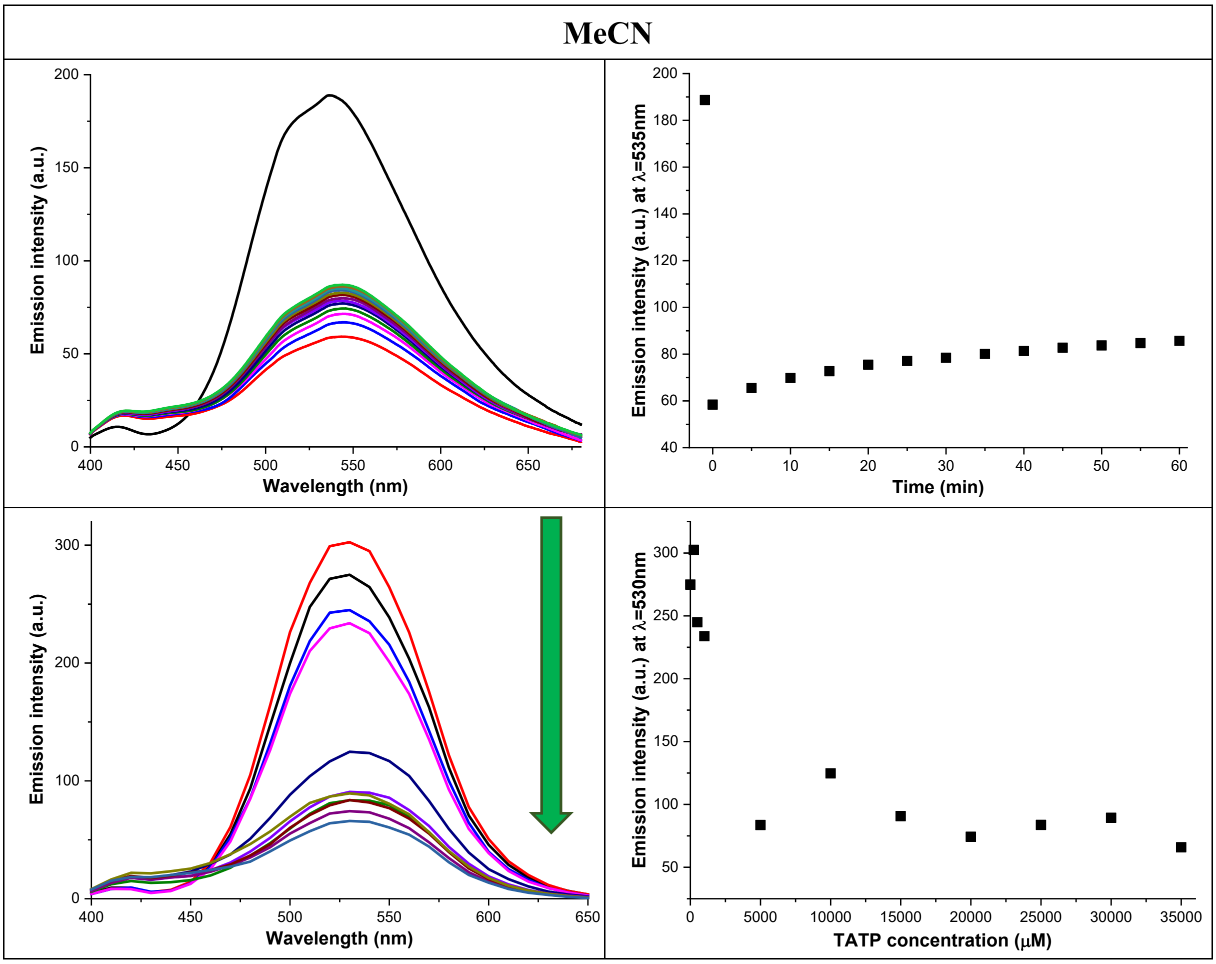

There was a drop in the emission intensity band immediately after adding TATP which was stabilized after 40 minutes. According to the fluorescence spectra, the LOD of TATP was somewhere between 1000 and 5000 μM, a little high for a good probe.

Figure S43. Kinetic study at different times under visible (up) and UV (down) light in MeCN. In each photo, left vial was contained the probe and right vial was contained the probe and an excess of TATP.

	**t=0**	**t=60 min**	**t=24 h**
**Visible light**	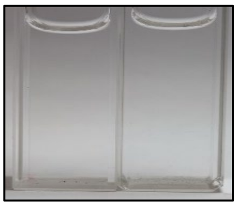	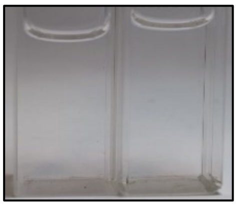	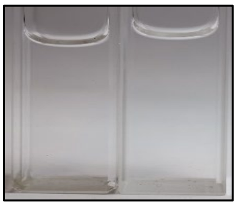
**UV light (366 nm)**	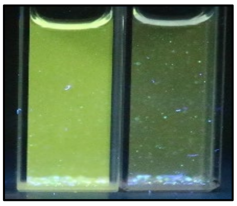	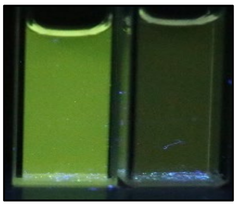	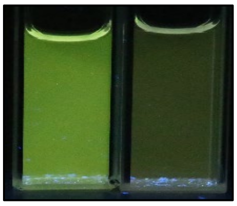

Figure S44. Titration of AR82s with an excess of TATP under visible (up) and UV (down) light in MeCN.

µM	0 250 500 1000 5000 10000 15000 20000 25000 30000 35000
**Visible light**	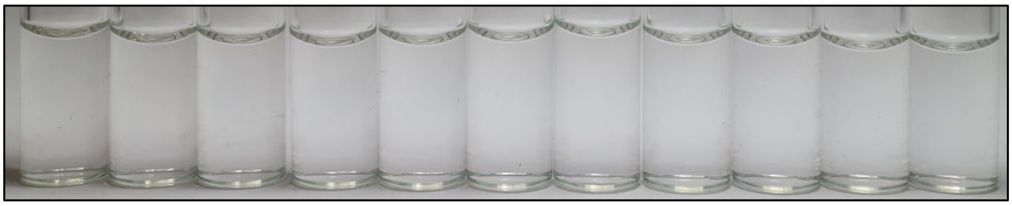
**UV light (366 nm)**	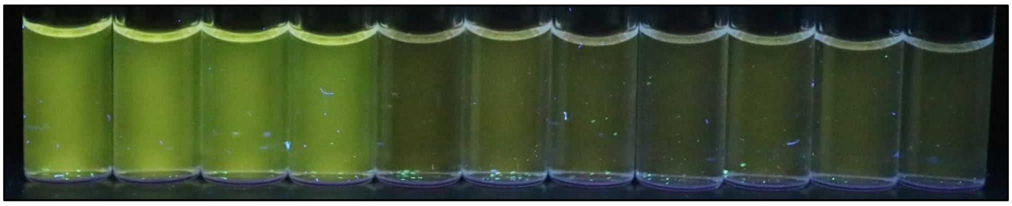

Figure S45. Study of AR82s with TATP in toluene. Kinetic study (up left) and profile as function as time at 460 nm (up right) in presence of TATP excess. Titration (down left) and fluorescence profile at 460 nm (down right) under increasing concentrations of TATP.

Toluene

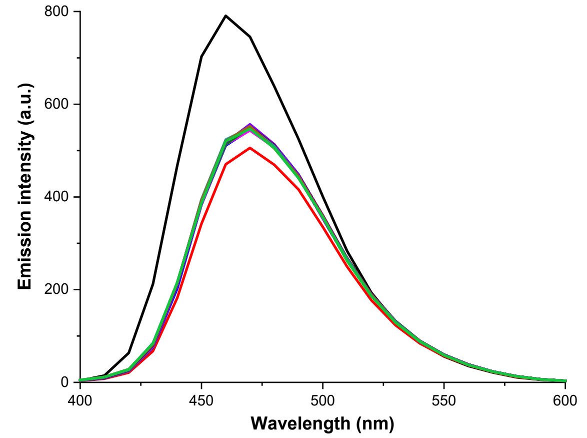

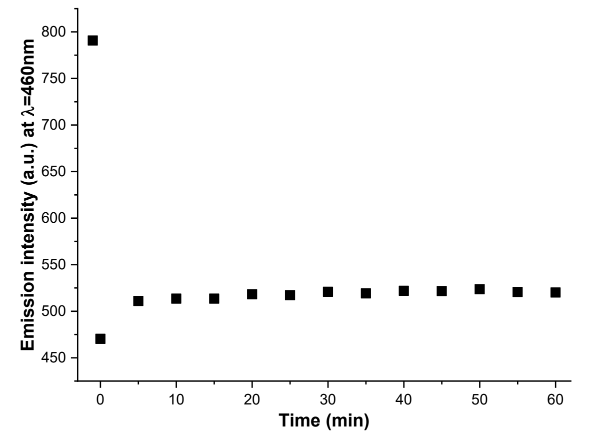

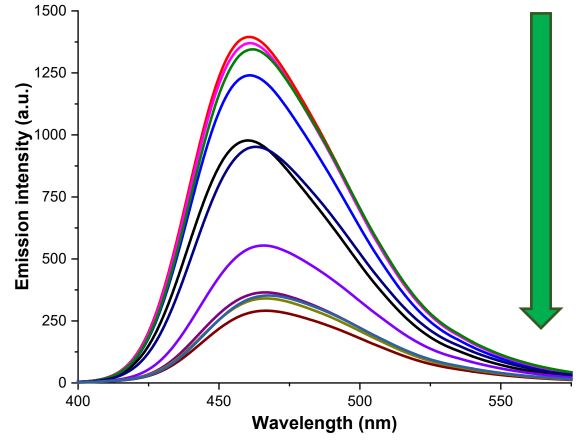

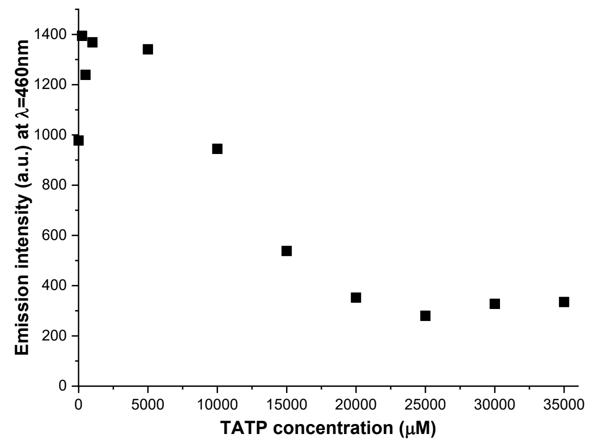

There was a decrease in the emission intensity band immediately after adding TATP which was stabilized after 5 minutes. According to the fluorescence spectra, the LOD of TATP was

somewhere between 5000 and 10000 µM with a small initial increase and then a considerable decrease in emission intensity.

Figure S46. Kinetic study at different times under visible (up) and UV (down) light in toluene. In each photo, left vial was contained the probe and right vial was contained the probe and an excess of TATP.

	**t=0**	**t=60 min**	**t=24 h**
**Visible light**	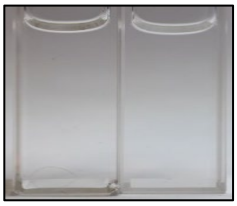	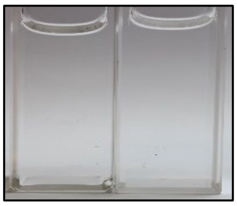	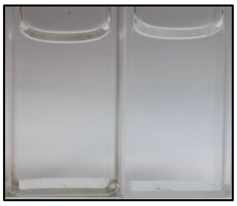
**UV light (366 nm)**	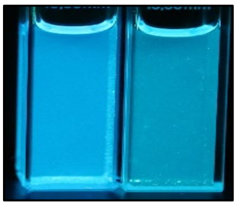	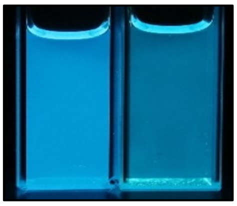	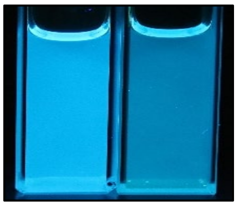

Figure S47. Titration of AR82s with an excess of TATP under visible (up) and UV (down) light in toluene.

µM	**0 250 500 1000 5000 10000 15000 20000 25000 30000 35000**
**Visible light**	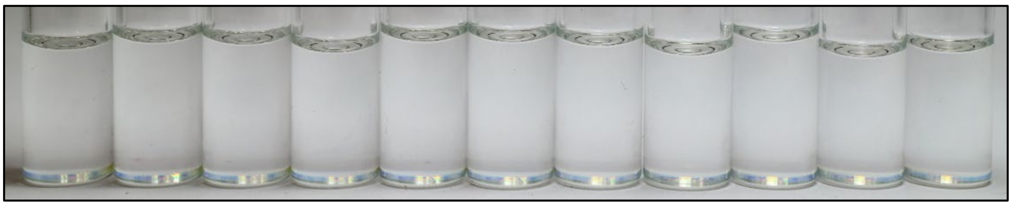
**UV light (366 nm)**	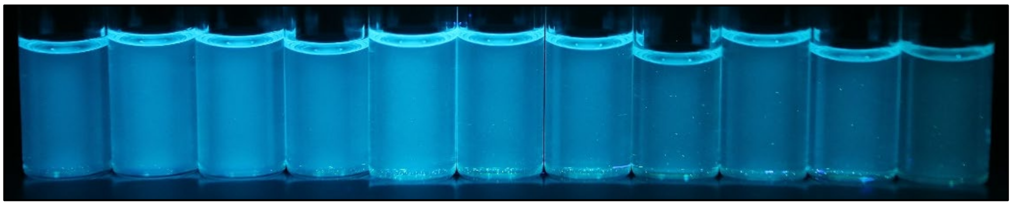

Figure S48. Study of AR82s with TATP in DCM. Kinetic study (up left) and profile as function as time at 500 nm (up right) in presence of TATP excess. Titration (down left) and fluorescence profile at 500 nm (down right) under increasing concentrations of TATP.

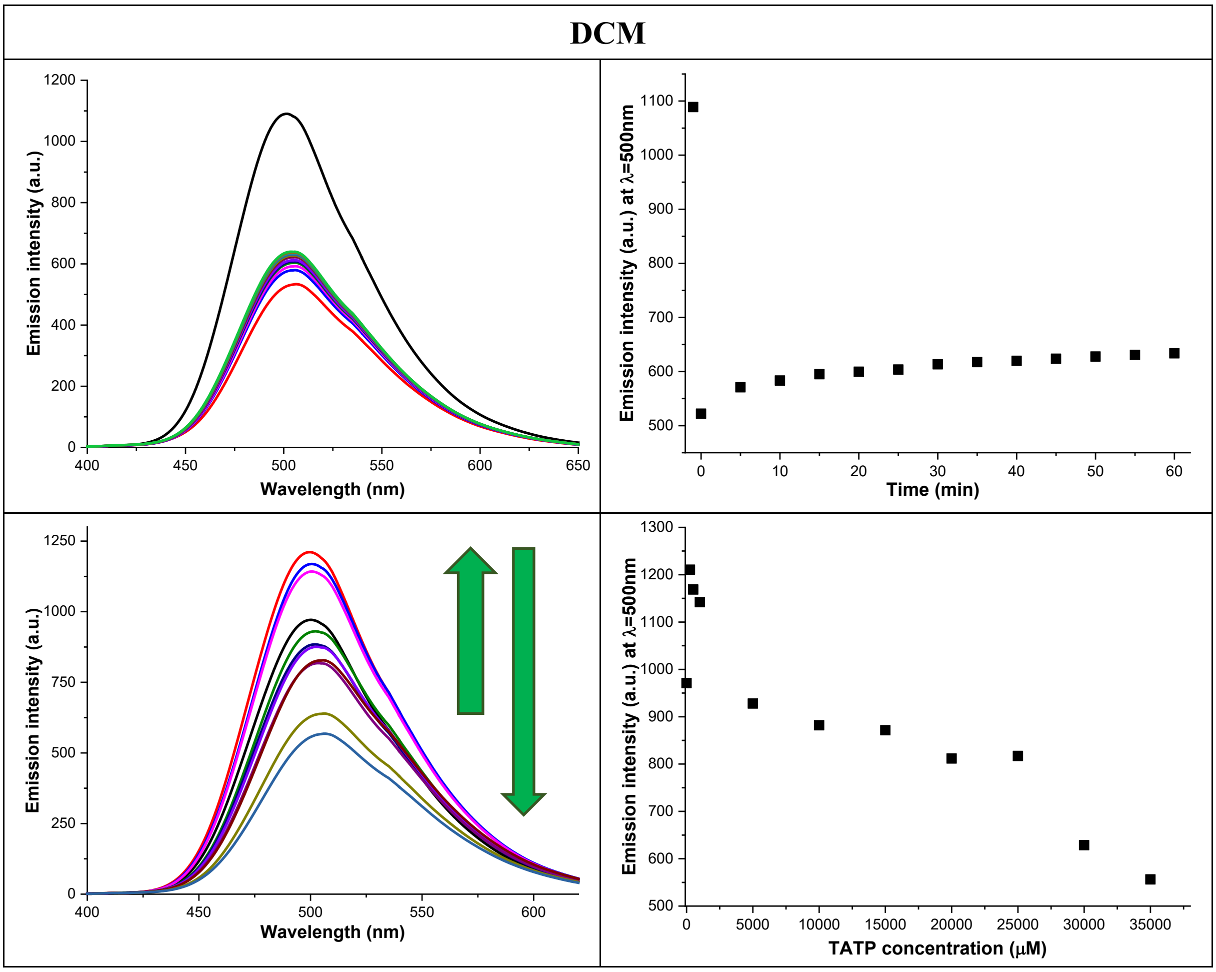

There was a decrease in the emission intensity band immediately after adding TATP which was stabilized after 30 minutes. According to the fluorescence spectra, the LOD of TATP was somewhere between 0 and 250 μM, it was the solvent that gave the lowest detection limit.

Figure S49. Kinetic study at different times under visible (up) and UV (down) light in DCM. In each photo, left vial was contained the probe and right vial was contained the probe and an excess of TATP.

	**t=0**	**t=60 min**	**t=24 h**
**Visible light**	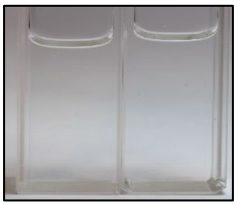	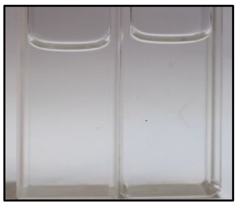	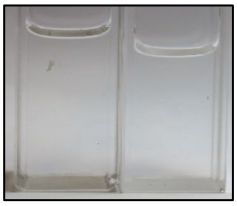
**UV light (366 nm)**	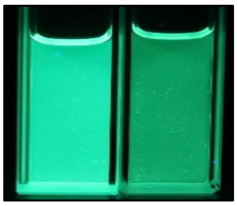	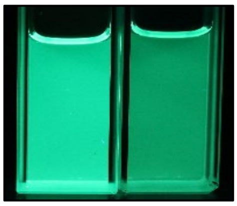	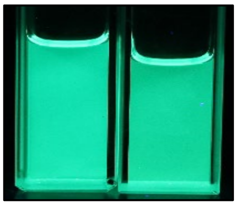

Figure S50. Titration of AR82s with an excess of TATP under visible (up) and UV (down) light in DCM.

µM	**0 250 500 1000 5000 10000 15000 20000 25000 30000 35000**
**Visible light**	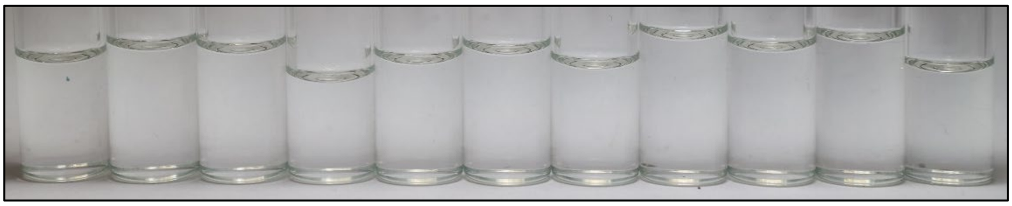
**UV light (366 nm)**	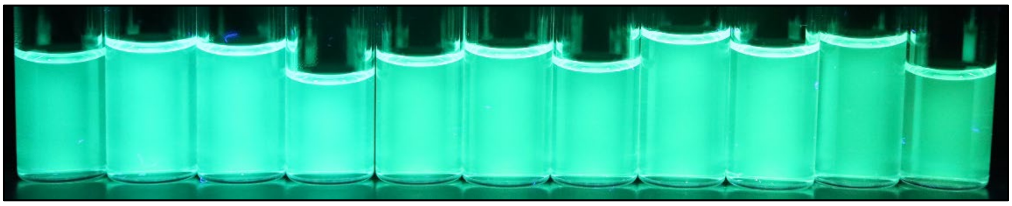

Titration with TATP in DCM and LOD calculations:

The working concentration of AR82s was 2.5 µM, the TATP was directly as a solid and its concentration was between 0 and 25 mM. The measurements were carried out immediately after the addition of TATP. The excitation wavelength was 370 nm and the fluorescence changes were registered at 25ºC.

Figure S51. Titration (up left), fluorescence profile at 500 nm (up right) and calibration for the LOD calculation using emission values at 500 nm (down) of a 2.5 µM of AR82s solution in DCM under increasing concentrations of TATP.

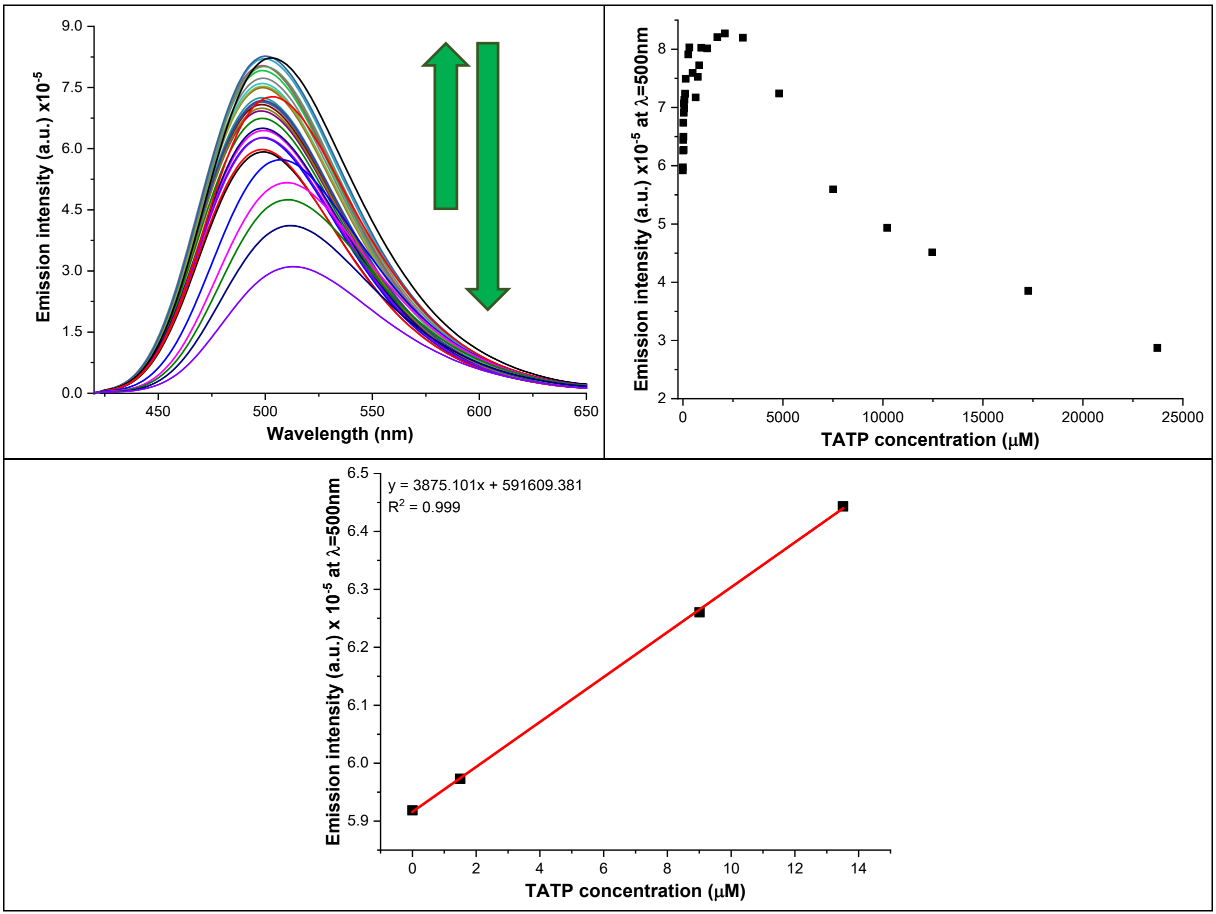

An initial increase in the emission intensity was observed, followed by a significant decrease in emission. In addition, the blank (sample only containing probe) was measured three times obtaining an intensity value of 596722.725 a.u. and a standard deviation of 5682.909 a.u. The LOD of AR82s with TATP was calculated.

Calculation of the LOD by linear regression + false positive and negative at 500 nm.

For this method, the R software was used and the value of the LOD was 0.87 µM.

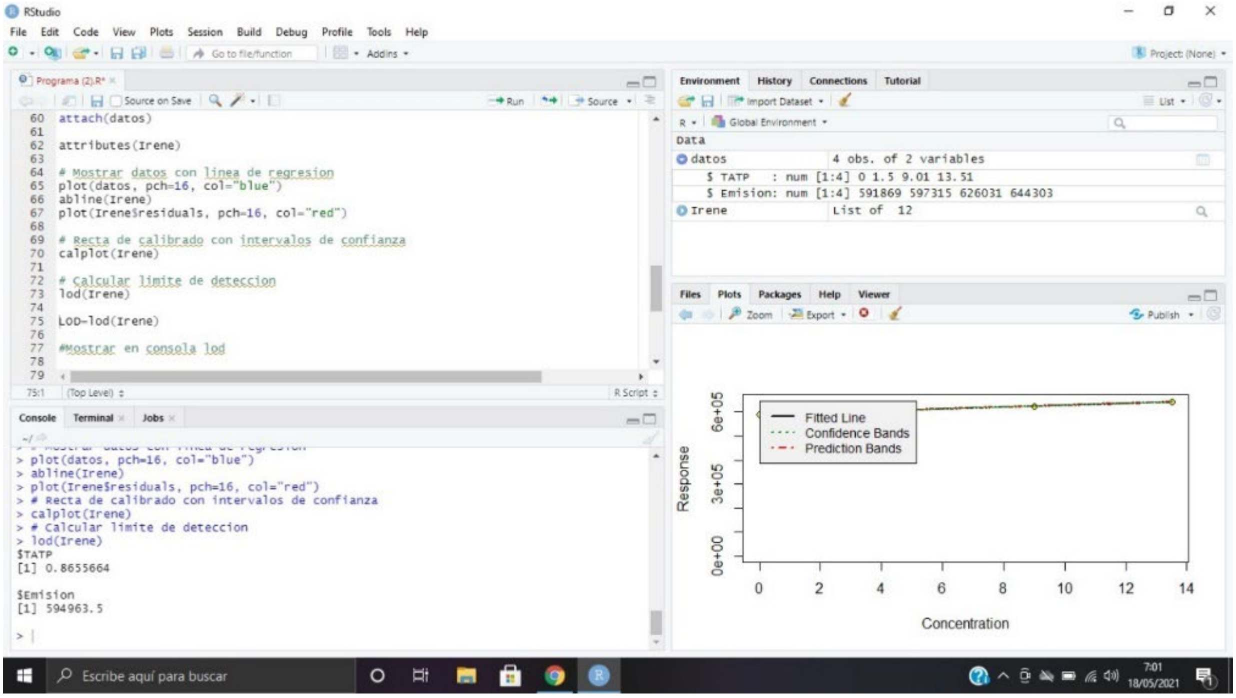

Figure S52. Calculation of LOD by the R software.

Titration with m-CPBA in DCM and LOD calculations:

To compare the results and the possibility of having different processes, not only the response to TATP was evaluated. The changes with m-CPBA, other strong oxidant, were also measured, determining their optical changes and the LOD.

The working concentration of AR82s was 2.5 µM, the excitation wavelength was 370 nm and the fluorescence changes were registered at 25ºC. In the kinetic study, the m-CPBA was in excess (7 mM) and the fluorescence emission measurements were made during the subsequent 2 hours, at the increasing time intervals. In the titration, the concentration of the m-CPBA was between 0 and 20 mM, measuring the variation of the fluorescence after each addition.

Figure S53. Kinetic study (left) and fluorescence profile at 430 nm and 500 nm simultaneously (right) of a 2.5 µM of AR82s solution in DCM under increasing concentrations of m-CPBA.

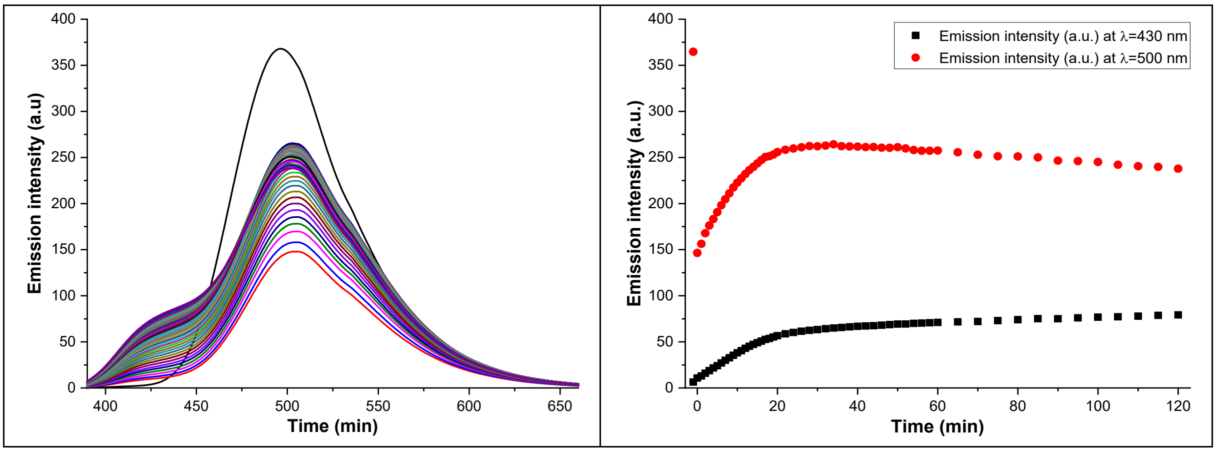

The fluorescence profile showed that the emission intensity in the band with a maximum of 500 nm fell drastically just after the addition of m-CPBA, at which point it begun to increase again, reaching a certain stability after 30 minutes. Initially, there was no band in the area of 430 nm but it begun to appear after the addition of m-CPBA and it increased gradually. It reached some stability after 30 minutes.

Figure S54. Titration (up left), fluorescence profile at 430 nm and 500 nm simultaneously (up right) and calibration for the LOD calculation using emission values at 430 nm (down right) and 500 nm (down left) of a 2.5 µM of AR82s solution in DCM under increasing concentrations of m-CPBA.

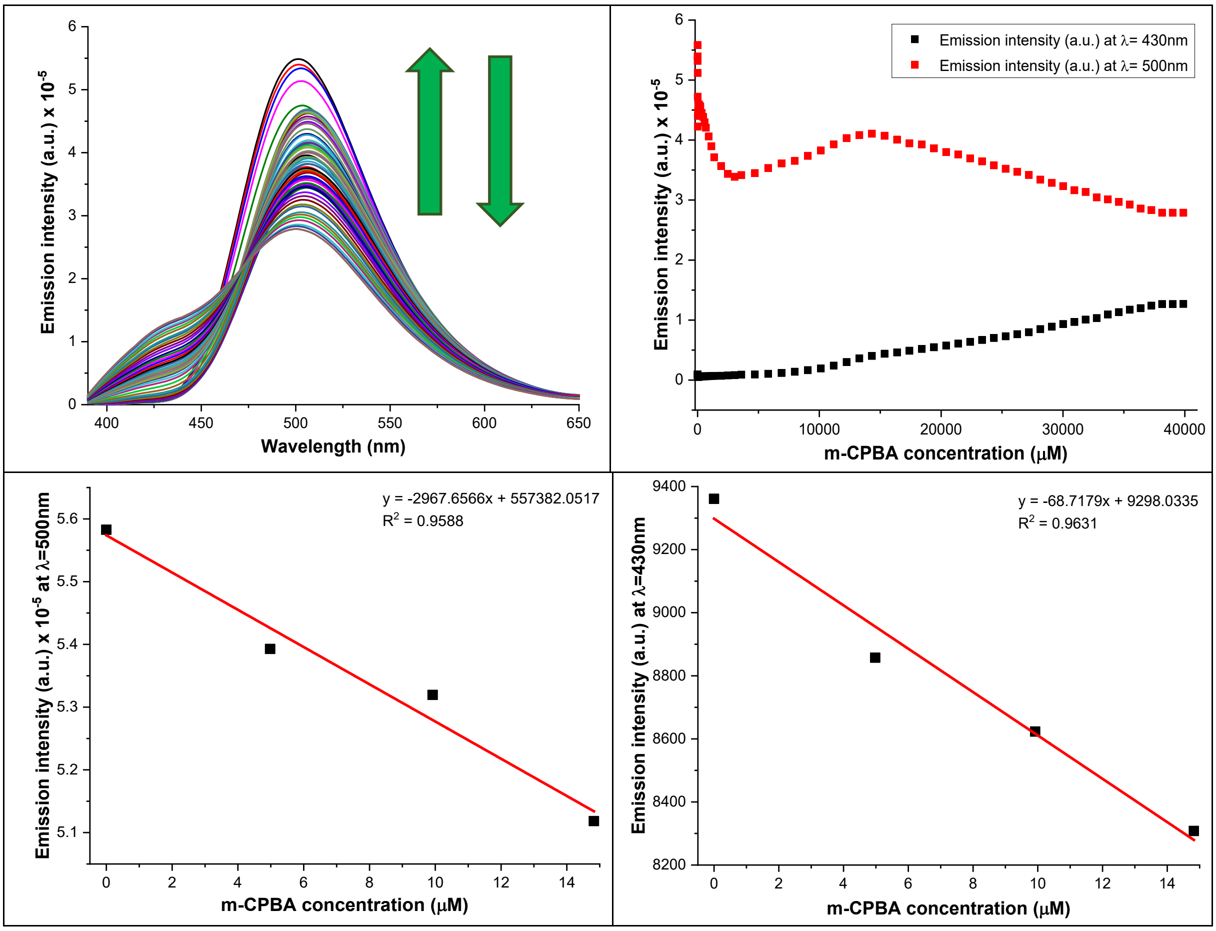

In addition, the blank (sample only containing probe) was measured three times obtaining an intensity value of 553148.256 a.u. and a standard deviation of 5055.503 a.u. The LOD of AR82s with TATP was calculated.

Calculation of the LOD by linear regression + false positive and negative at 500 nm.

For this method, the R software was used and the value of the LOD was 2.1 nM.

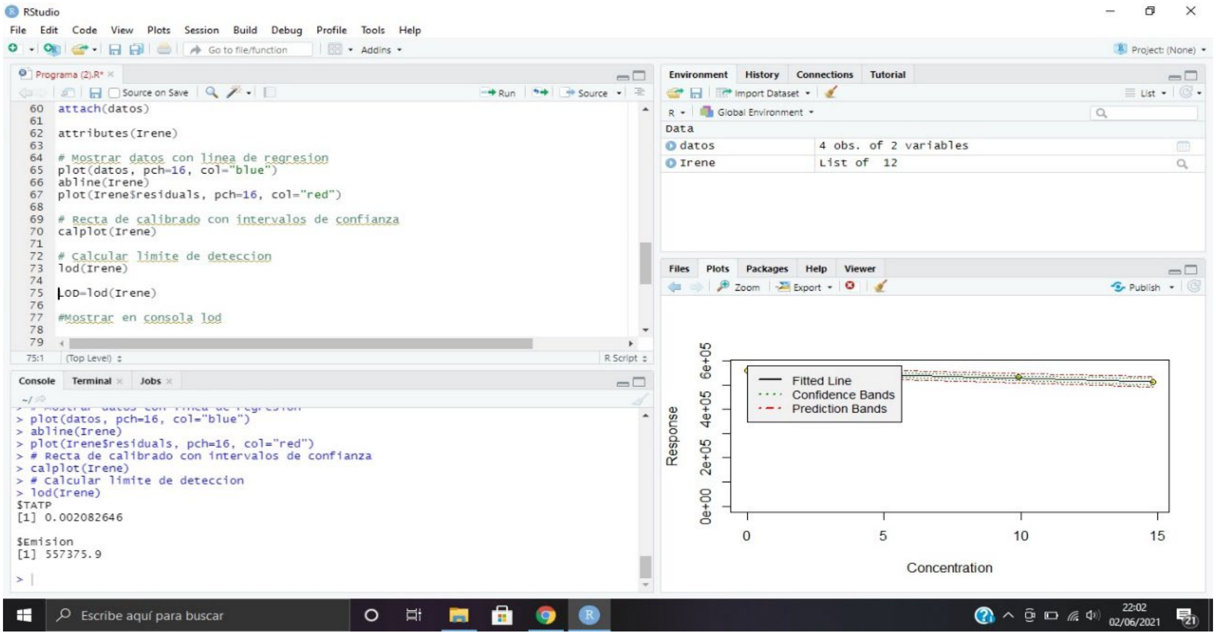

Figure S55. Calculation of LOD by the R software.

Calculation of the LOD by linear regression + false positive and negative at 430 nm.

For this method, the R software was used and the value of the LOD was 2.1 nM.

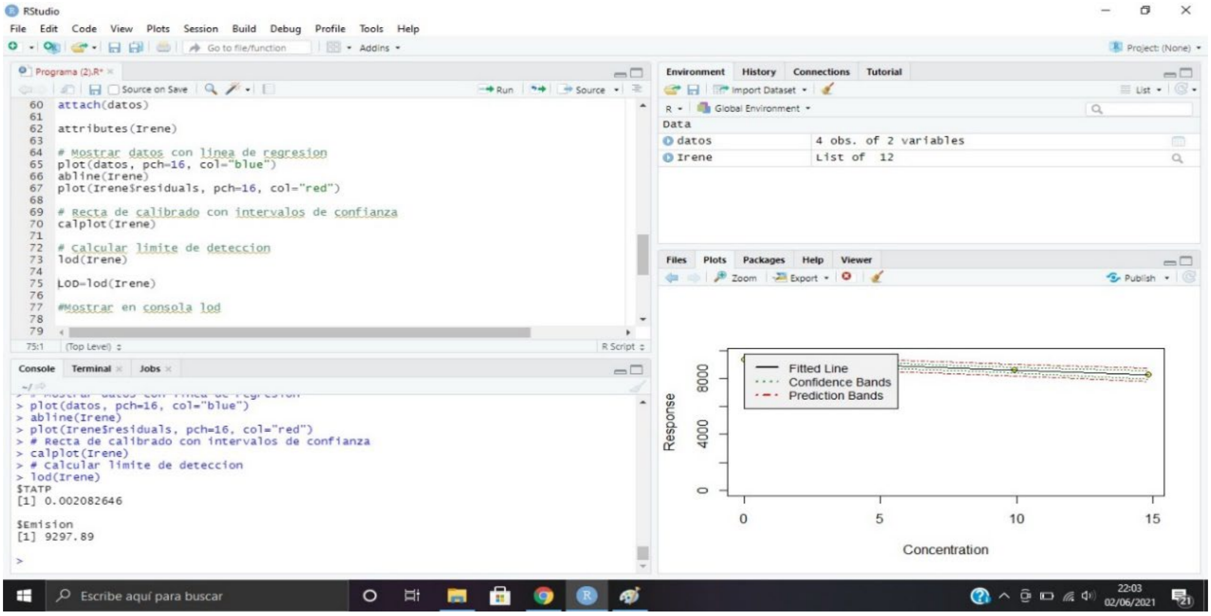

Figure S56. Calculation of LOD by the R software.

TATP gas detection by modified nanoparticles:

Several nanoparticles samples were modified by adsorption of the probe and tested as potential TATP sensors. In all three cases, 100 mg of the SiO_2 nanoparticles (5 – 15 μm particle size, 99.5% trace metal basis, Aldrich Chemistry) and 1 mg of the probe were added to a 5 ml of $CHCl_3$ and thin foil was used to cover the vial to avoid degradation of the nanoparticles by the action of light. The resulting suspension was stirred at 60 ºC under a stream of nitrogen until complete evaporation of solvent (approximately 30 minutes). After that, nanoparticles with the adsorbed compound (AR82s·SiO_2) were washed with hexane (3×3 ml) and the solvent residues were dried with a stream of nitrogen, working in the dark throughout the process.

The supported probe was evaluated by several methods that was given complementary information about the influence of different analytes. In a first instance, the color variation of the nanoparticles before and after exposure to TATP was tested. Afterwards, the detection capacity was evaluated by a more quantitative approach by registering changes in fluorescence intensity (by comparing the fluorescence quantum yield for higher precision and in the position of the maximum of emission. Finally, a quantitative study for the specific detection of TATP vapors was also performed.

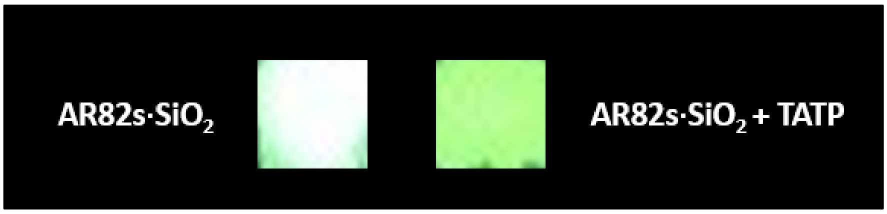

Figure S57. Color comparation between the nanoparticles with the probe over glass plates before and after the exposure to TATP.

The increase in fluorescence of the solid was recorded by measuring the fluorescence of the solids and determining the increase in fluorescence quantum yield (ØF). The most straightforward way to detect the presence of the analyte was the increase in fluorescence of the modified material. However, the measurements were sometimes reliant on the position of the solid in the fluorometer (especially in powder samples) and these factors were difficult to control without designing a specific device for doing so. As a consequence, fluorescence quantum yields were the most trustworthy way to measure it quantitatively.

The initial evaluation of the AR82s adsorbed on SiO_2 nanoparticles against TATP in vapor was carried out by placing, at the bottom of an Eppendorf, 30 mg of the corresponding nanoparticles and 4 mg of TATP in the upper part of the Eppendorff, which was placed horizontally. The Eppendorf was covered with tin foil to prevent the degradation of the nanoparticles by action of light and was heated, at a temperature not higher than 60ºC, until the complete evaporation of TATP. Once evaporated, the probe and the TATP were kept in contact for ten minutes more before proceeding to measure the emission and the excitation spectrums and the quantum yield of the nanoparticles, both exposed to the TATP and before the exposure, with an Edinburg Instrument FLS – 980 fluorometer at an excitation wavelength of 370 nm an at 25ºC.

Figure S58. Response of fluorescence (down) and excitation (up) of AR82s adsorbed in SiO_2 nanoparticles.

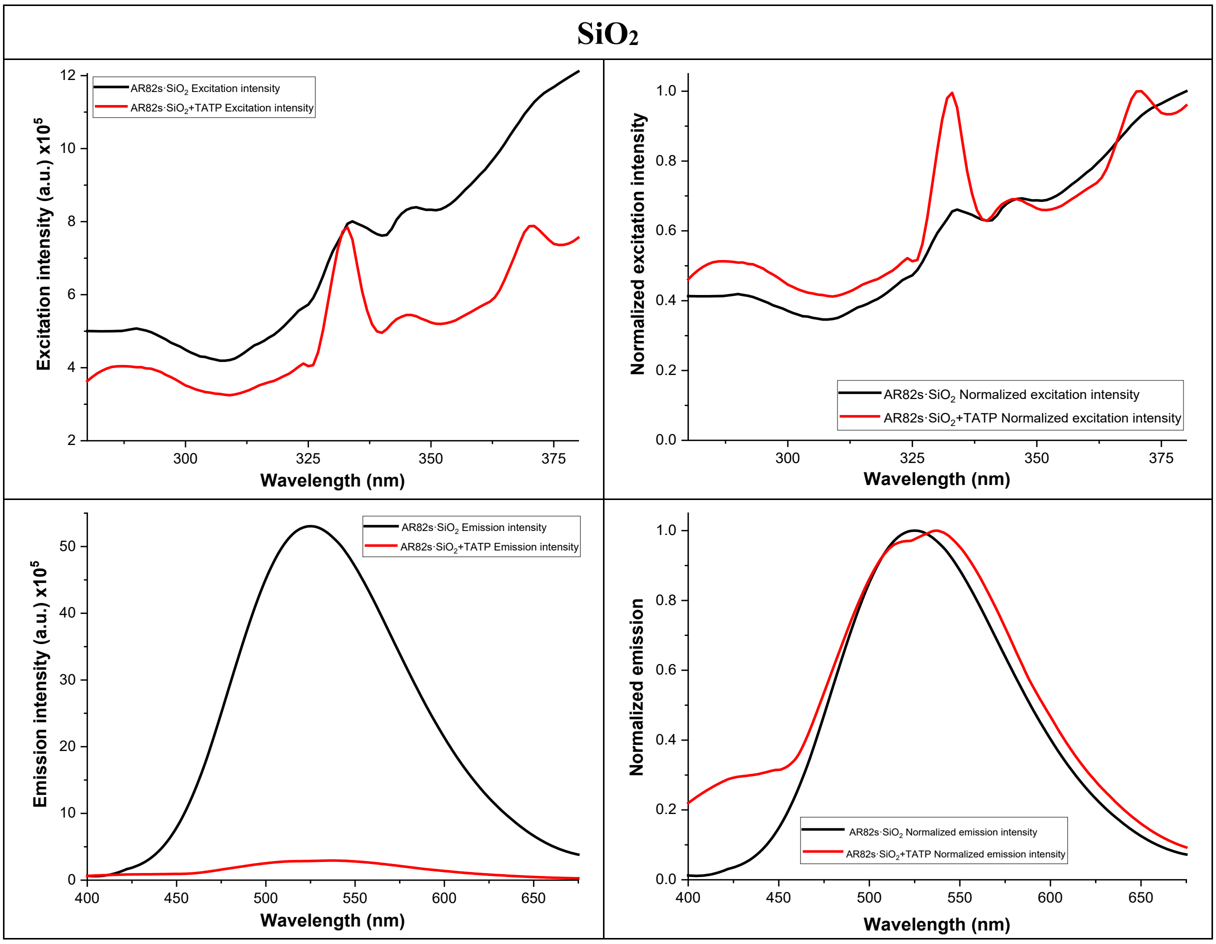

The variation of quantum yields between the probe and the probe after the exposure to TATP was a parameter that provides lower errors, less than 2%.

$$\frac{\emptyset_{probe+TATP}}{\emptyset_{probe}} = 0.081$$

Fluorescence intensity with the probe was around 12 times lower after the exposure to TATP vapor.

Titration with TATP in vapor phase:

The titration of AR82s adsorbed onto SiO_2 nanoparticles was carried out by placing 15 mg of nanoparticles in an Eppendorf, covered with tin foil to avoid degradation of the nanoparticles by the action of light, and increasing amounts of TATP (from 0.025 to 2 mg) in another Eppendorf. Both were connected to each other and to an air stream, air flow, 100 cm^3/min, provided by a compressor. Working in the absence of light, TATP was heated, to a temperature below 50ºC, until its complete evaporation, keeping probe and TATP under recirculation in the system for 10 additional minutes after the complete TATP evaporation. After this time, the emission measurements of the nanoparticles exposed to TATP were carried out in an Edinburg Instrument FLS-980 fluorometer at an excitation wavelength of 370 nm at 25ºC.

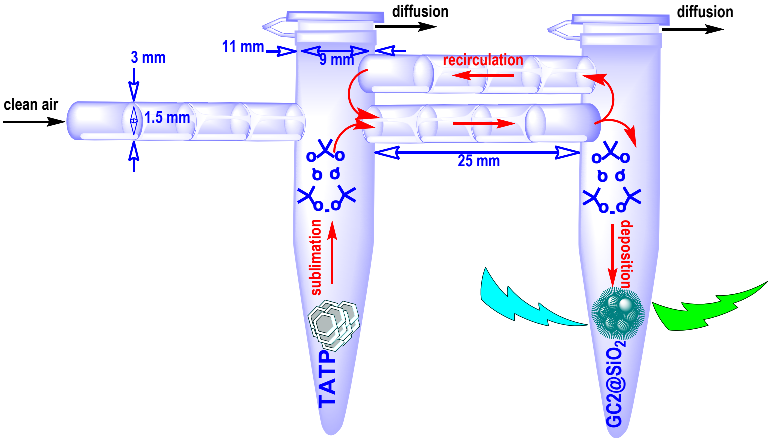

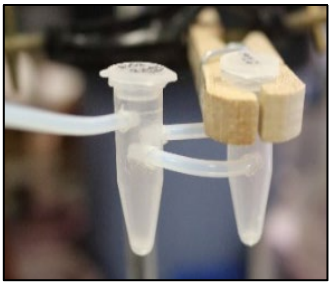

Figure S59. Microfluidic device used for titrations in vapor phase (left) and the actual aspect of the system (right).

The microfluidic device dimensions were: Eppendorf: outer top diameter 11mm, inner top diameter 9 mm, outer bottom diameter 5 mm, inner bottom diameter 3 mm, volume 1.5 ml, height (with lid) 40 mm, height (without lid) 38 mm. Tubing: outer diameter 3 mm, inner diameter 1.5 mm, length (flow tube) 25 mm, length (return tube) 35 mm.

Figure S60. Time required for total evaporation of the different amounts of TATP used in the experiment.

TATP (mg)	Time required for complete evaporation
0.032	1’41”
0.048	1’45”
0.083	2’14”
0.114	2’42”
0.158	3’10”
0.203	3’21”
0.247	3’57”
0.333	4’26”
0.483	5’03”
0.800	7’45”
1.000	11’23”
1.400	13’13”
2.100	19’37”

The calibration was performed in steady state, fixed quantities of TATP were vaporized in the presence of a sample containing 15 mg of AR82s·SiO_2 nanoparticles and the fluorescence increase was measured 10 minutes after the complete vaporization of each TATP sample.

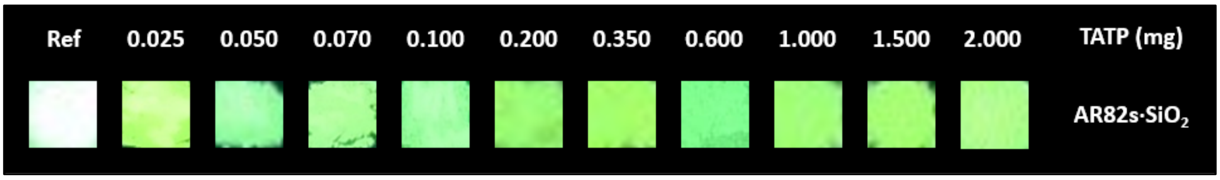

Figure S61. Response of AR82s·SiO$_2$ exposed to different quantities of TATP.

Figure S62. Titration (up left), fluorescence profile at 535 nm (up right) and calibration for the LOD calculation using emission values at 535 nm (down) of AR82s·SiO$_2$ under increasing concentrations of TATP in vapor phase.

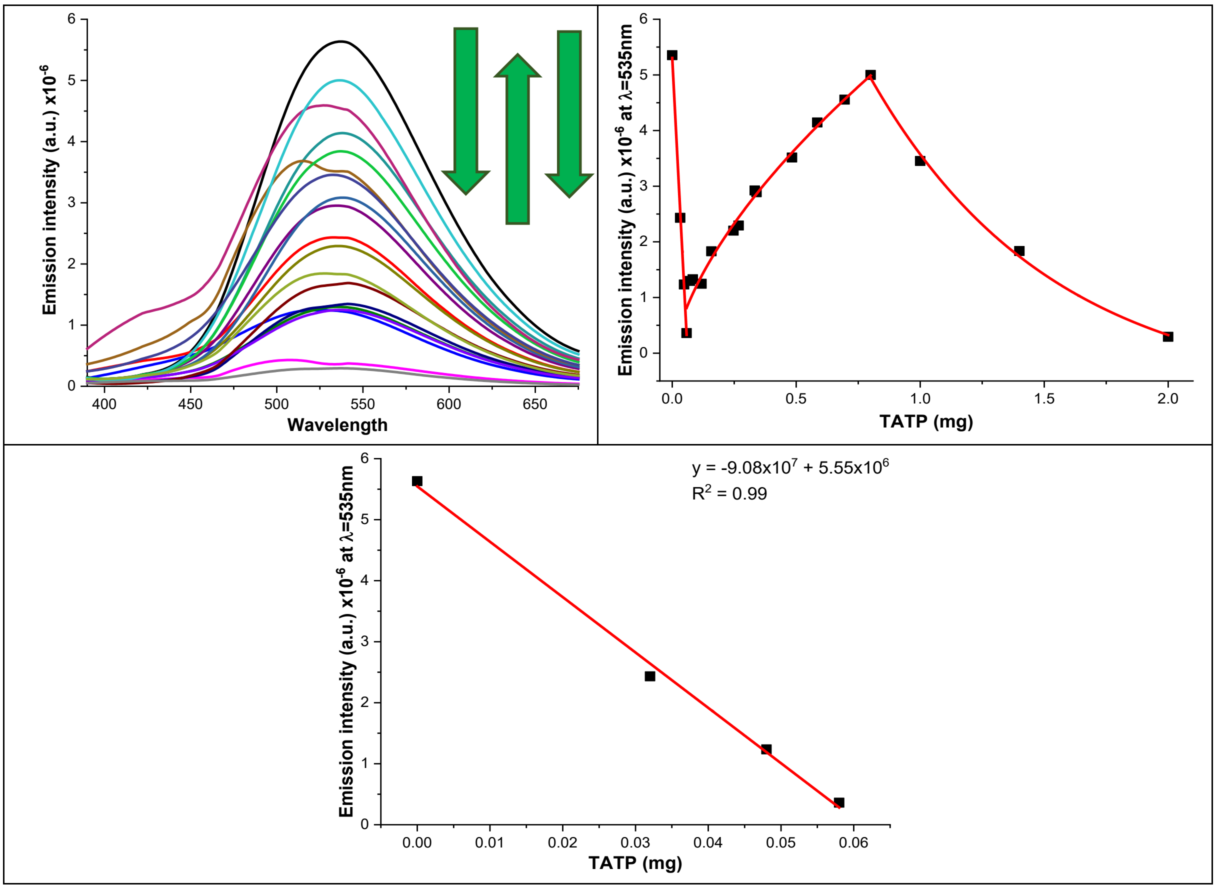

The blank (sample only containing probe) was measured three times obtaining an intensity value of 5454206.667 a.u. and a standard deviation of 154231.172 a.u. The LOD was calculated by linear regression + false positive and negative at 535 nm.

For this method, the R software was used and the value of the LOD was $1.303 \cdot 10^{-5}$ mg or 0.01303 µg.

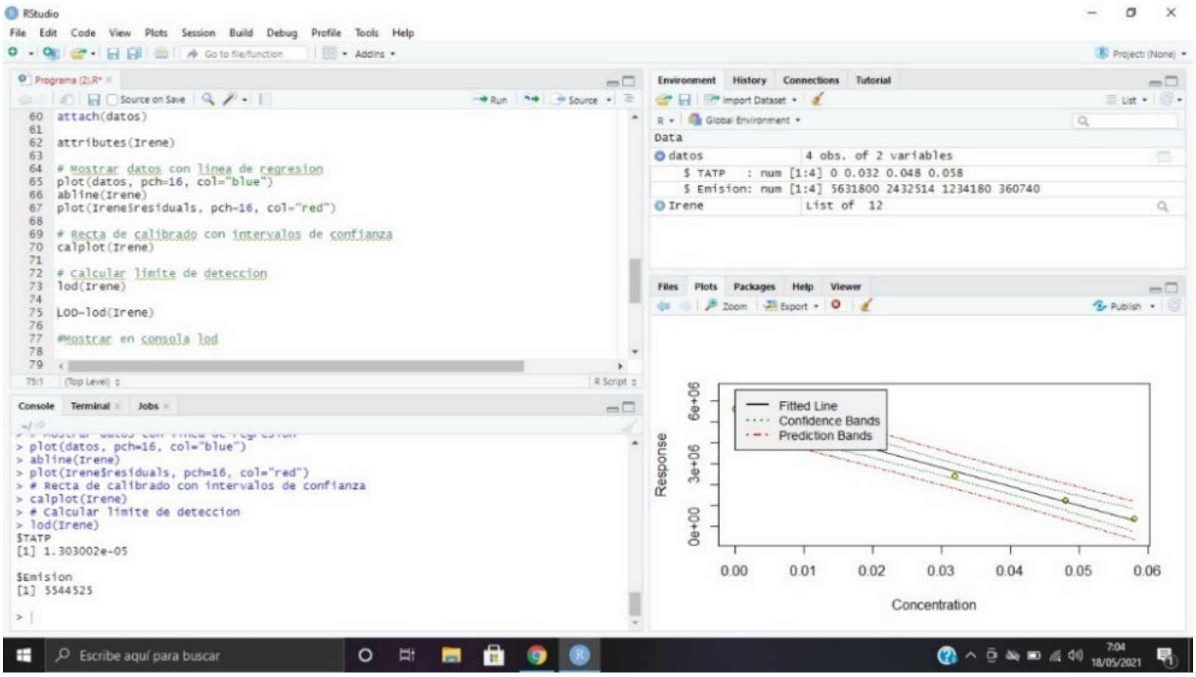

Figure S63. Calculation of LOD by the R software.

Mobile phone application:

In this case an OPPO Find X3 Pro 5G (dedicated to the application) was used to detect the presence of TATP in a vapor flow. The application was developed in collaboration with J. Rafael Santana Tejada (Movilmatica, www.movilmatica.com).

The purpose of the application was to detect the amount of TATP from an imagen of AR82s·SiO_2 sample exposed to TATP vapors. The user had to configure the system correctly in order to obtain a suitable result. The application has two systems for determining the amount of TATP by RGB:

- Interval search: each sample exposed to a known amount of TATP was configured with several colors, in which the target could be included. To detect the amount of TATP to which the nanoparticles were exposed, a color was selected and an interval search was carried out. Example: suppose we have a target color 50, 240, 70 and we have the following intervals [40, 130, 60], [10,250, 60] and [55, 244, 71]. The comparison algorithm will detect that the target 50, 240, 70 is in the interval [40, 230, 60] 50, 240, 70 [55, 244, 71].
- Radio search: suppose we have a target color 50, 240, 70 and we work with a radius of 10. The intervals we have are [40, 230, 60], [10, 250, 60] and [85, 244, 41]. When the compare algorithm will detect that the target 50, 240, 70 is in the radius of the interval [40, 230, 60] since if we add the radius ±10 we would have this radius [40, 230, 60] or [60, 250, 80].

The system of the application was composed of the following components:

- Mobile application: it was developed with IONIC technology with which we have the possibility of generating an APK for Android or IPA for IOS.
- *Laravel api*: communication with the backend where queries were made from the mobile application to the database.
- Database: system of tables designed to be able to store colors dynamically and to meet the needs of the application.

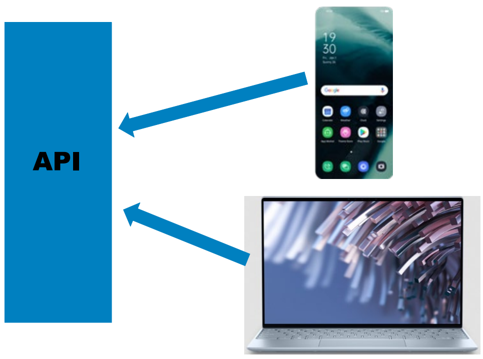

Figure S64. System structure of the mobile application

System configuration: The application is made up of different configurable sections:

- Login: to be able to enter the system, access credentials are required.
- Menu: allows us to view and access to the different options of the application.
- Users: the list of users authorized to access the application is displayed, as well as the option to delete them.
- User settings: personal data (name, email, password…) can be changed from this screen.
- New user: allows you to create new users by filling in the required information. The data will be verified before granting access to the application.
- Color list: list of the different nanoparticle colors for each amount of TATP in vapor phase, accessed from "menu" and then "my colors". In this screen, colors can be deleted and the data updated at any time as well as watch the rest of the stored colors.
- New color: to add a new color to a TATP measurement, we click on "add color" and select the TATP quantity and add an RGB color, manually or by selecting it on an image. The latter is the fastest method as it allows you to save and continue adding colors.
- Color detection: once the system is configured with the different TATP measurements, in the "analysis" screen you can take a picture or select an image from the gallery and choose the point where you want to detect how much TATP it has been exposed to. The images are very important because the higher the quality, the higher the accuracy in detecting RGB color.

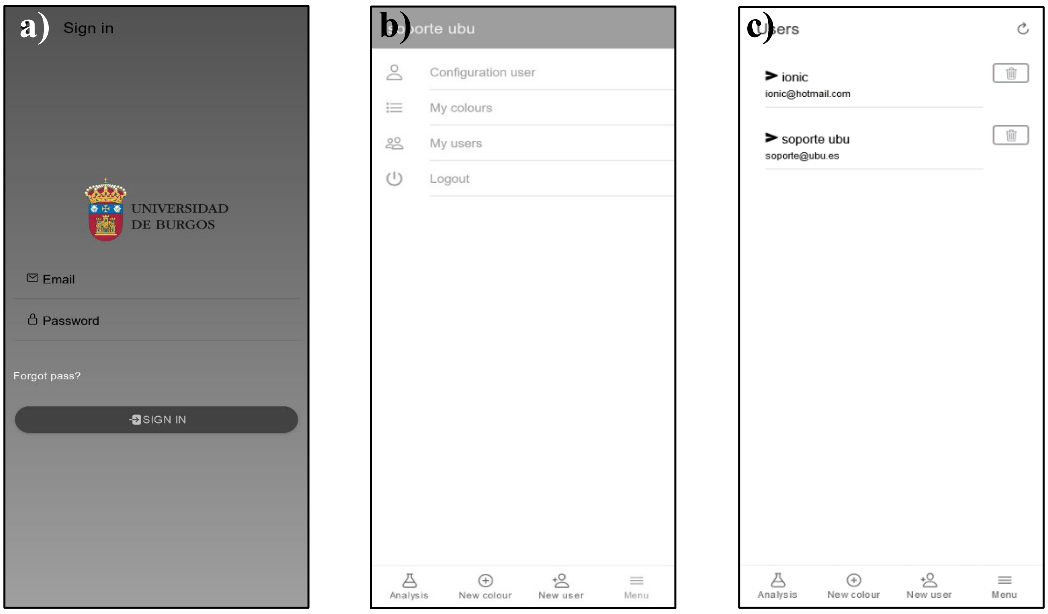

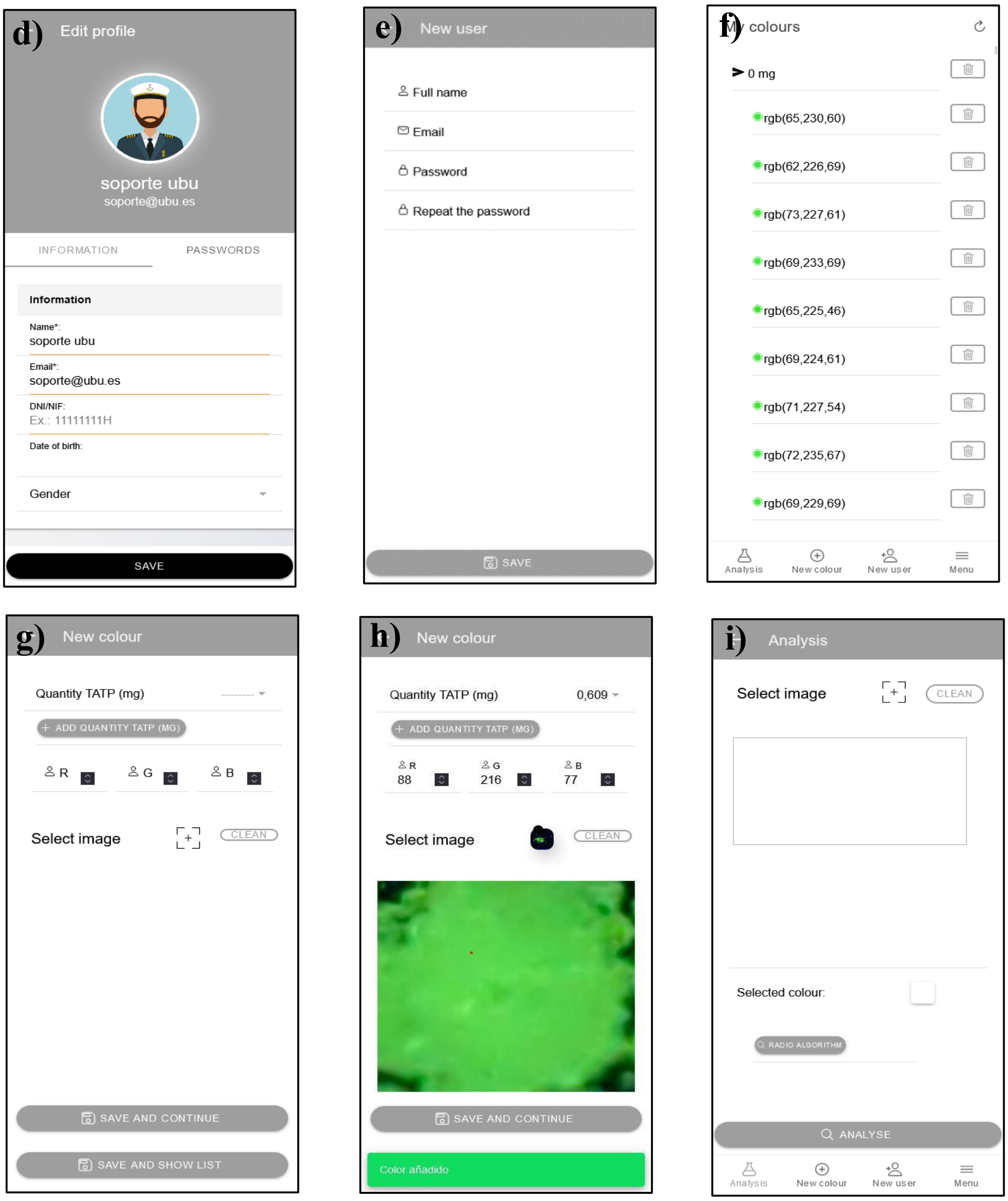

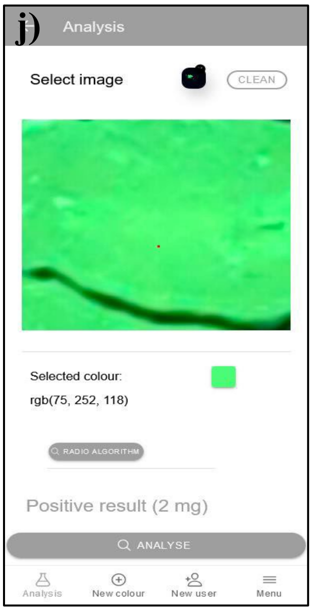

Figure S65. Screenshots of the mobile application.
a) login.
b) menu.
c) list of users.
d) edit profile of an user.
e) new user.
f) list of colours.
g) and h) new colour.
i) and j) colour detection

The figures showed an example for a 2 mg image obtained by the device from previous section and the system with the color configuration should detect it. As we can see, the system is correct since the range of colors is in the correct range. In the lower part we have the option to change the radio search algorithm that is named in the introduction section.

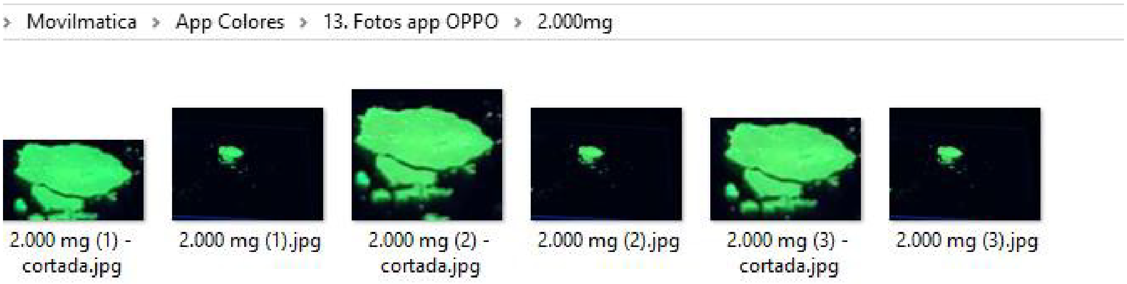

Figure S66. Images of the data base

The app was based on:

Ionic https://ionicframework.com, Laravel, https://laravel.com/docs/8.x/eloquent-resources, Data base, https://www.mysql.com

The application was part of an autonomous system for capturing and quantitative evaluation of fluorescent probe images based on mobile telephony composed of: (1) customized display box (2) 360º metal bracket (3) customized APP and (4) OPPO Find X3 Pro5G. The system allows photographs to be taken with a mobile camera from very short distances of a sensor material previously exposed to an explosive atmosphere. For this, the sensing material was placed in a dark box with a metal holder and a UV lamp (366 nm) to illuminate the material. The application to compare the photo with a database of images of the material exposed to different amounts of TATP in vapor phase is loaded on the mobile phone so, that from the comparison and the calculated calibration curve, the amount of the explosive that has come into contact with the AR82s·SiO_2 can be extracted.

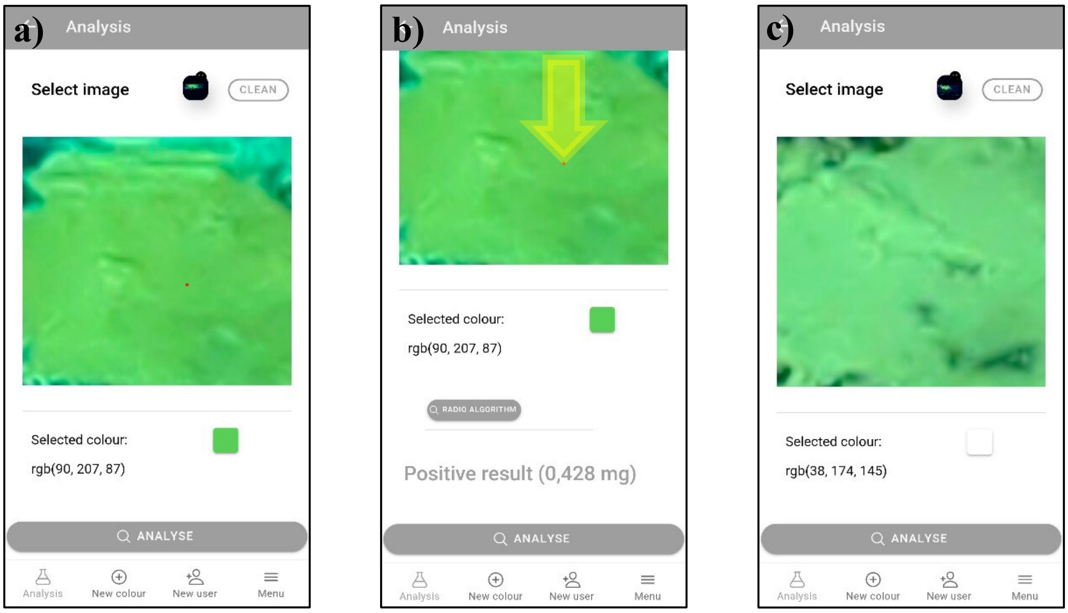

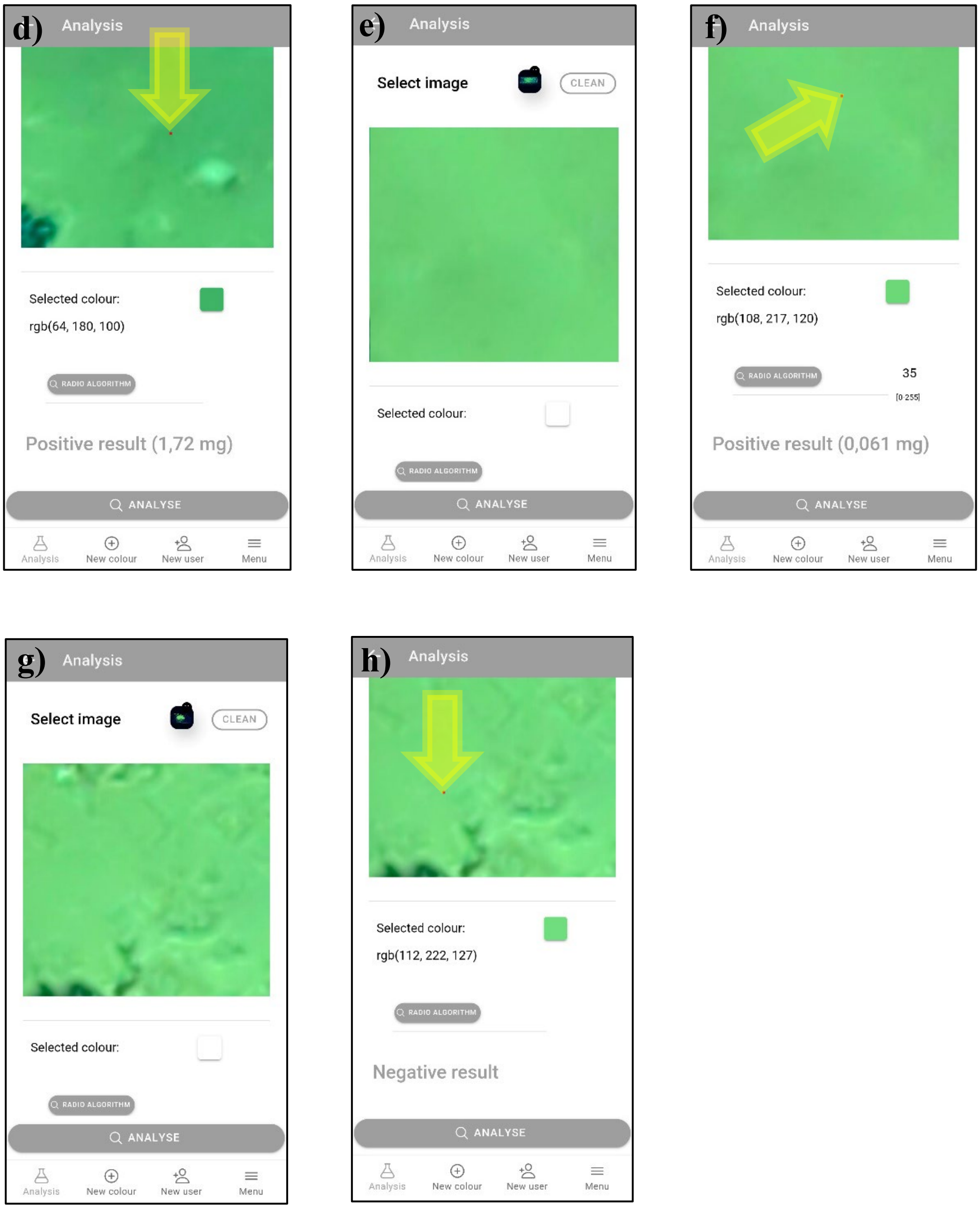

Figure S67. Images of the data base used for search and compare selected samples.
a) Selected image (0.428 mg of TATP in system Figure 2a).
b) A positive result.
c) Selected image (1.720 mg of TATP in system Figure 2a).
d) A positive result.
e) Selected image (0.061 mg of TATP in system Figure 2a).
f) A positive result.
g) Selected image (no TATP in system Figure 2a).
h) A negative result.
The yellow arrows indicate the point where the selected color is measured

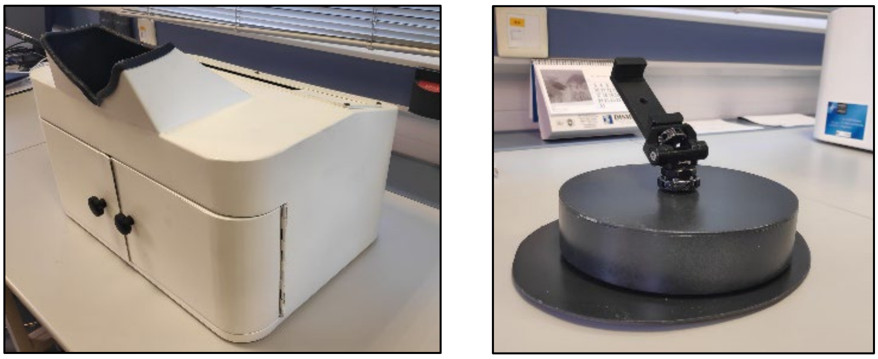

Figure S68. The dark box (left) with a metal support (right).

Validation of fluorescent device in real life scenarios:

The experiments were carried out in an office with dimensions 3.07×3.54×2.15 m (height × length × width). An air compressor was placed inside it, which was generated a continuous stream of clean air, that was passed through the TATP in the direction of the point where the particles were arranged, and three heaters that help regulate the temperature inside the room.

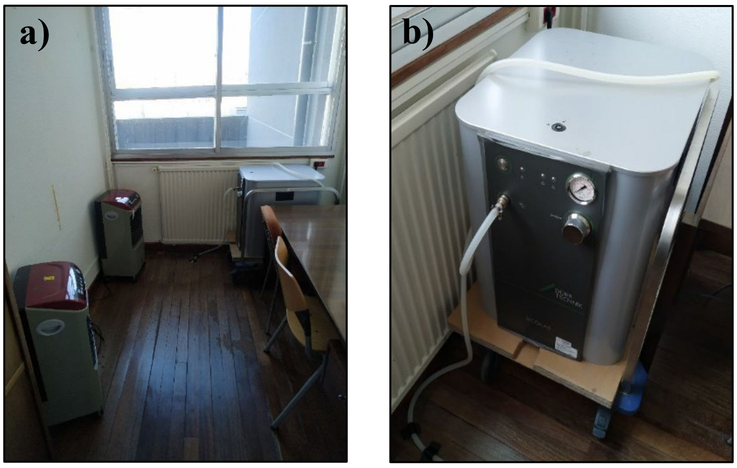

Figure S69. Office experiments with AR82s·SiO_2 with TATP in vapor phase. a) shows the dimensions and the arrangement of the elements inside the office. b) shows the compressor used in the experiments.

Varying amounts of TATP (specified in each experiment) were placed 30 cm from the source of the airflow. Approximately 0.75 mg of AR82s·SiO_2 were placed on the surface of a cover glass, adhered to it with the help of a small amount of adhesive tape. This process was repeated 6 times and the cover glasses were placed at increasing distances from the point where the TATP was located (10 – 25 – 50 – 100 – 150 – 200 cm).

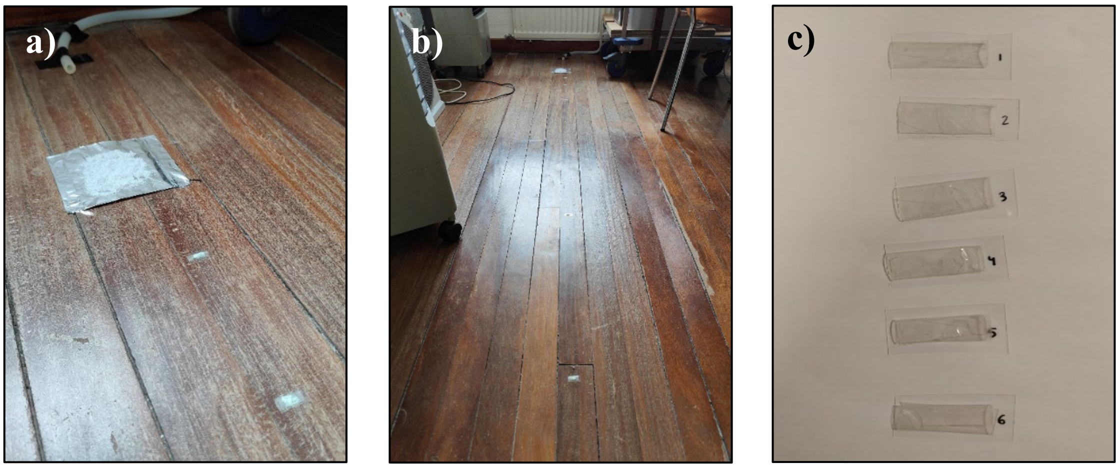

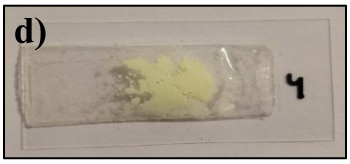

Figure S70. Office experiments with AR82s·SiO_2 with TATP in the vapour phase.
a) arrangement of the TATP in front of yhe airflow outlet.
b) distribution of the sensing nanoparticles in the room.
c) and d) assembly for measurement of the particles.

To carry out the measurements in the fluorometer, they were covered with a quartz sheet that ensured the nanoparticles remained in a fixed position. This sheet had then to be removed to leave the particles exposed to TATP vapors during the experiment. A measurement of the particles without being subjected to TATP was also made before and after remaining the same time as the particles that were subjected to TATP vapors to use as a reference.

Fluorescence measurements were made for each group of particles before and after the exposure to TATP vapors using a excitation wavelength of 370 nm. Photos of the particles were taken under identical conditions. Two representative experiments are detailed.

Experiment 4. 250 mg of TATP at 26ºC during 30 minutes.

Of the 252.3 mg of TATP initially weighed, only 38.6 mg were evaporated under the temperature and time conditions used in this experiment, remaining 216.7 mg of TATP as solid. The experimental procedure was similar to the previously explained.

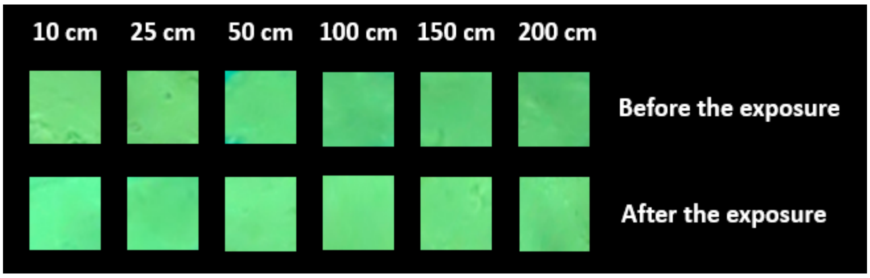

Figure S71. Comparative photograph taken under 366 nm light before and after exposure to TATP vapors at 26ºC during 30 minutes.

Experiment 5. 250 mg of TATP at 28ºC during 30 minutes.

Of the 252.3 mg of TATP initially weighed, only 97.3 mg were evaporated under the temperature and time conditions used in this experiment, remaining 155 mg of TATP as solid. The experimental procedure for these measurements was similar to the previous one.

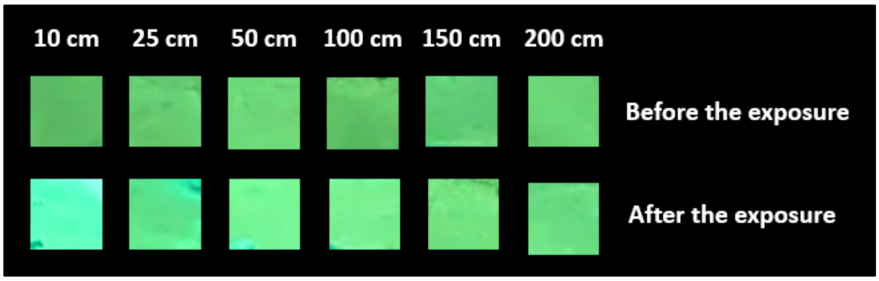

Figure S72. Comparative photograph taken under 366 nm light before and after exposure to TATP vapors at 28ºC during 30 minutes.

From the experiments, the following Figures have been made, representing the normalized emission intensity variation before and after exposure to TATP vapors versus distance to the TATP source.

Figure S73. Summary of experiments at different temperatures. Normalized emission intensity variation before and after exposure to TATP vapor versus distance to the TATP source in both cases.

<table>
<tr><th>20ºC</th><th>22ºC</th></tr>
<tr><td>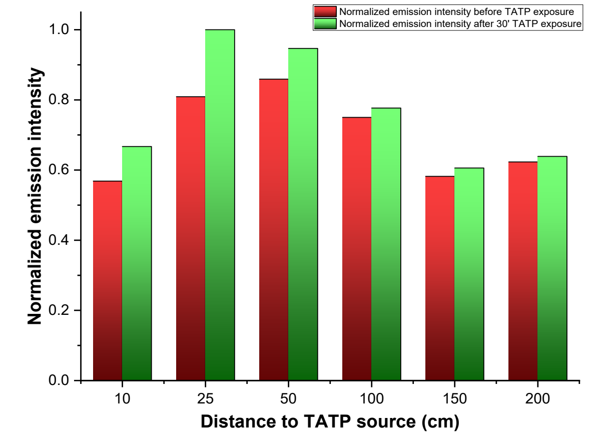
</td><td>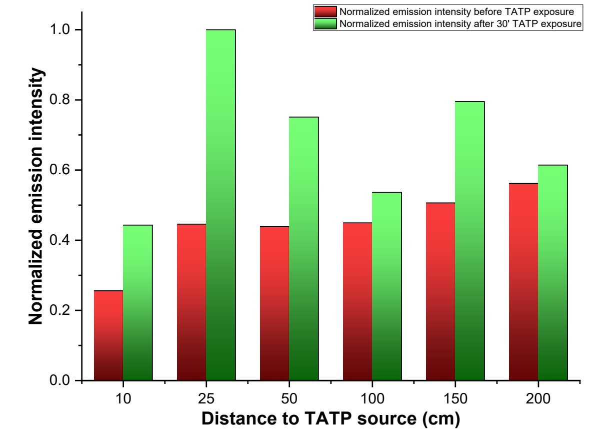
</td></tr>
<tr><th>24ºC</th><th>26ºC</th></tr>
<tr><td>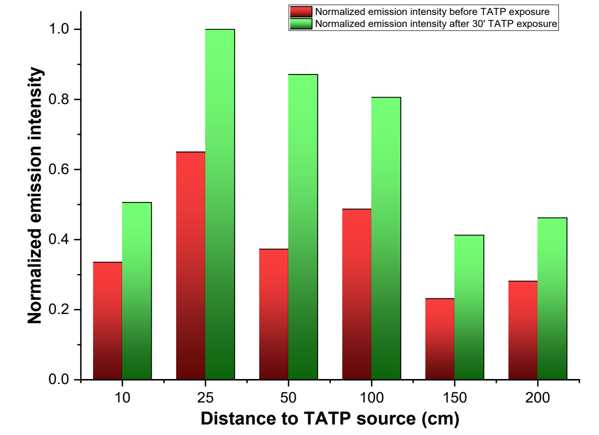
</td><td>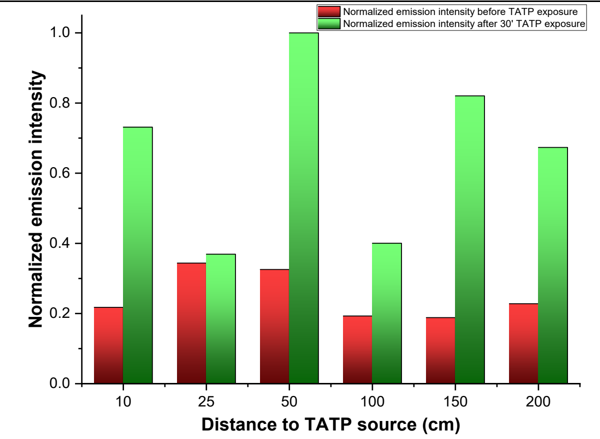
</td></tr>
<tr><th>28ºC</th><th>30ºC</th></tr>
</table>

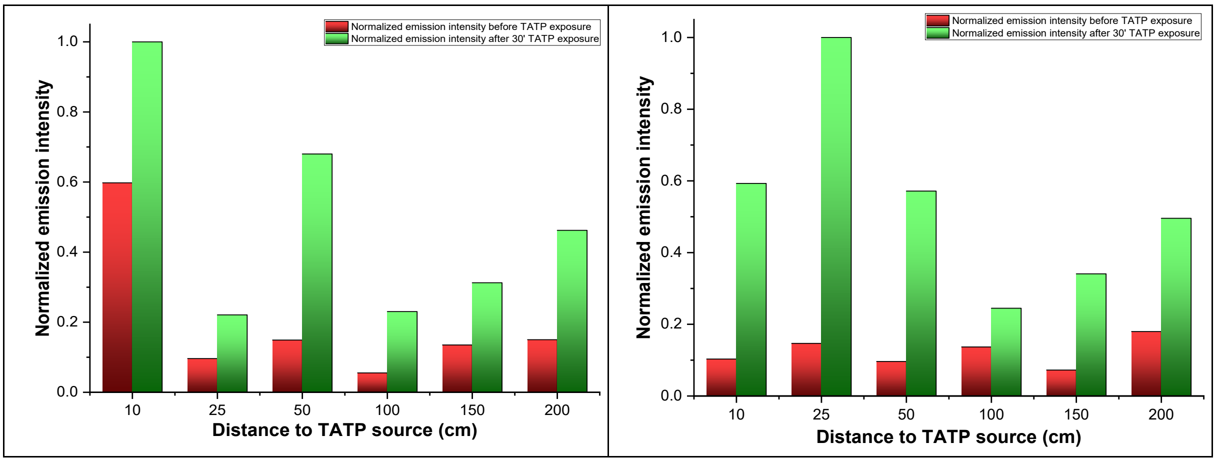

The following graph shows all the data together to facilitate comparison.

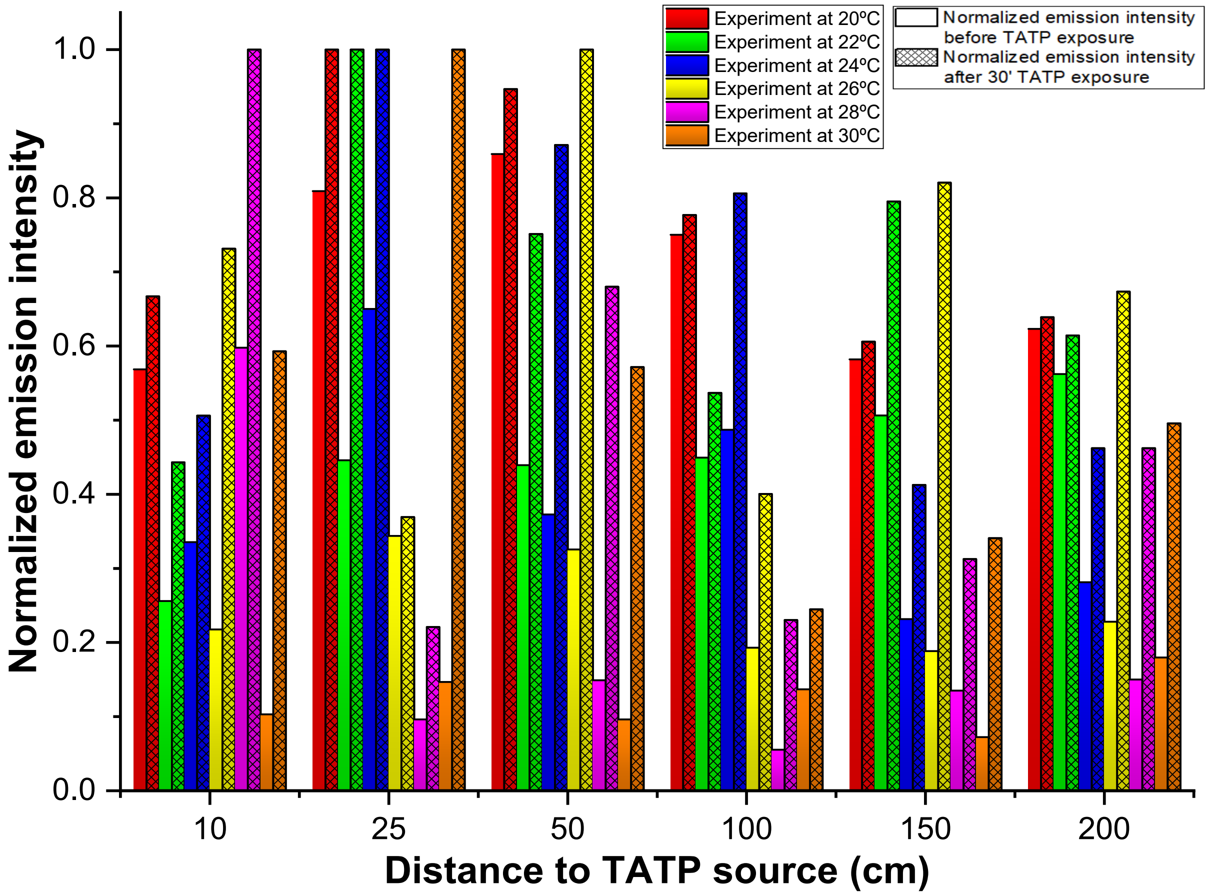

Figure S74. Summary of experiments at different temperatures. Normalized emission intensity variation before and after exposure to TATP versus distance to the TATP source.

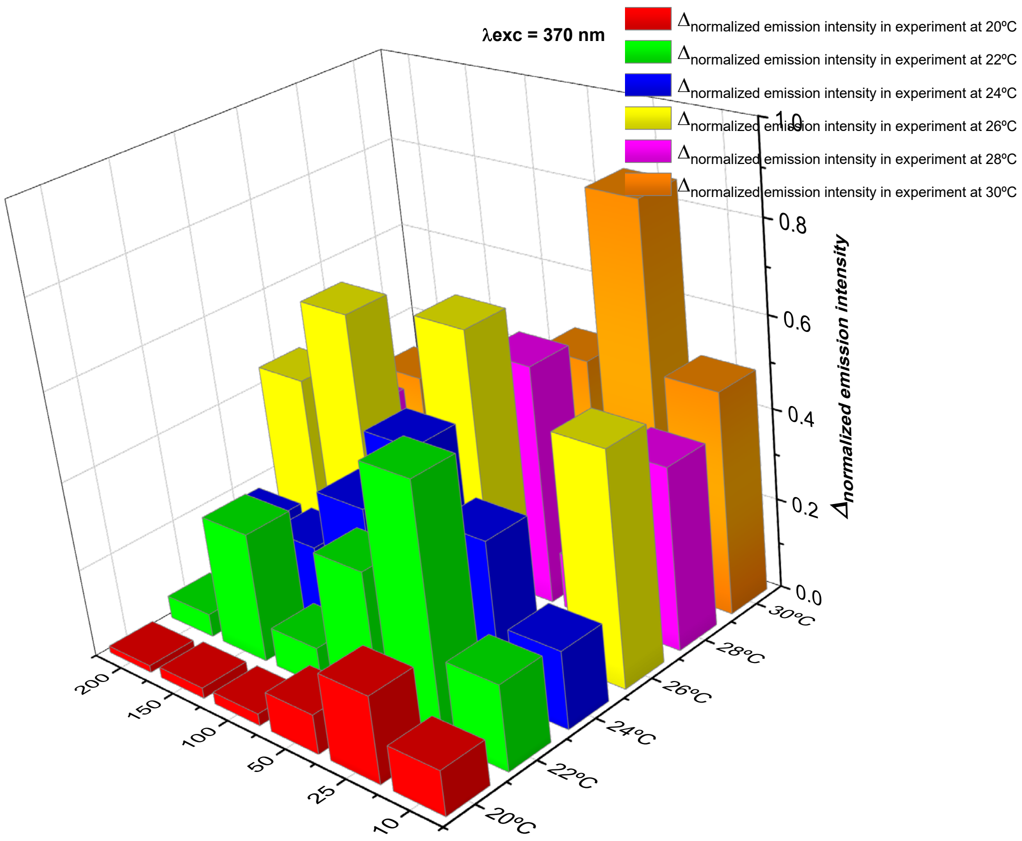

Figure S75. Summary 3D graph of experiments at different temperatures. Increase in normalized emission intensity variation before and after exposure to TATP versus distance to the TATP source.

Following the results of the previous series of experiments, it was observed that the best temperature conditions to perform the experiment was around 26ºC. Therefore, a series of 6 additional experiments were performed in which room temperature was raised up to 30ºC by the use of an air heater. Once this temperature was reached, the heater was turned off and the room temperature was left to decrease until an average temperature of 26ºC was obtained. The experiments had a duration of 30 minutes.

The experiments were carried out in an office with dimensions 3.07×3.54×2.15 m (height × length × width). The following figures illustrate both the dimensions and the arrangement of the different elements in the room when proceeding with the experiments.

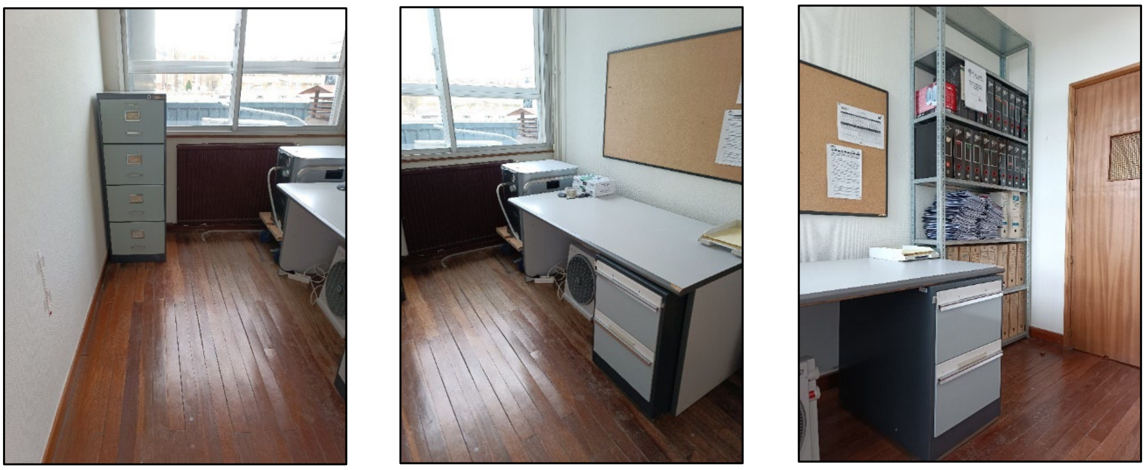

Figure S76. Images of the dimensions and arrangement of the elements inside the office.

Inside it, an air compressor was placed, which generated a continuous stream of clean air that passed through the TATP the direction of the points where the particles were arranged, and a heater that helped regulate the temperature inside the room. Once a temperature around 30ºC was reached, the heater was turned off and the temperature was left to reach an average temperature of 26ºC to perform the experiments.

For all experiments, 250 mg of TATP were placed 30 cm from the source of the airflow. Approximately, 0.75 mg of AR82s·SiO_2 nanoparticles were placed on the surface of a cover glass, adhered to it with the help of a small amount of adhesive tape, as the previous experiments. This process was repeated six times and the cover glasses were placed at increasing distances from the point where the TATP was located (10, 25, 50, 100, 150 and 200 cm).

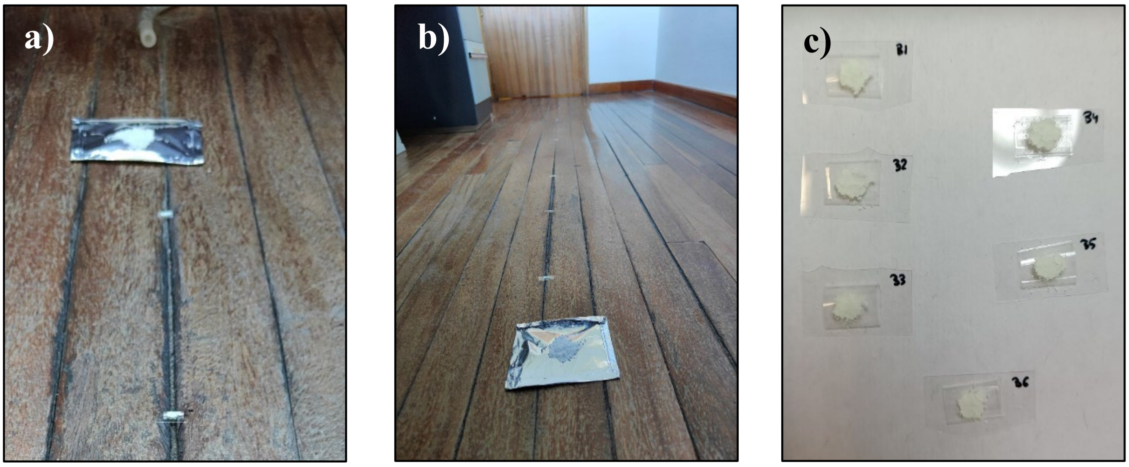

Figure S77. Arrangement the AR82·SiO_2 nanoparticles in the office.
a) Arrangement of the TATP in front of the airflow outlet.
b) distribution of sensing nanoparticles in the room.
c) assembly for measurement of the nanoparticles.

Once TATP and nanoparticles were placed, these were left in the presence of TATP vapors for a period of 30 minutes. To carry out the measurements in the fluorometer, they were covered with a quartz sheet that ensured the particles remained in a fixed position. This sheet was removed to leave the particles exposed to TATP vapors during the experiment. A measurement of the particles without being subjected to TATP was also made before and after remaining the same time as the particles that were subjected to TATP vapors to use as a reference.

Fluorescence measurements were made for each group of particles before and after the exposure to TATP vapors. Photos of the particles were taken under identical conditions and the excitation wavelength was 370 nm.

Experiment 7: at 26 ºC during 30 minutes.

Of the 256 mg of TATP initially weighed, only 23 mg were evaporated under the temperature and time conditions used in this experiment, remaining 233 mg of TATP as solid.

Figure S78. Emission spectra of the nanoparticles before and after exposure to TATP vapors at 26 - 30ºC during 30 minutes.

10 cm	25 cm
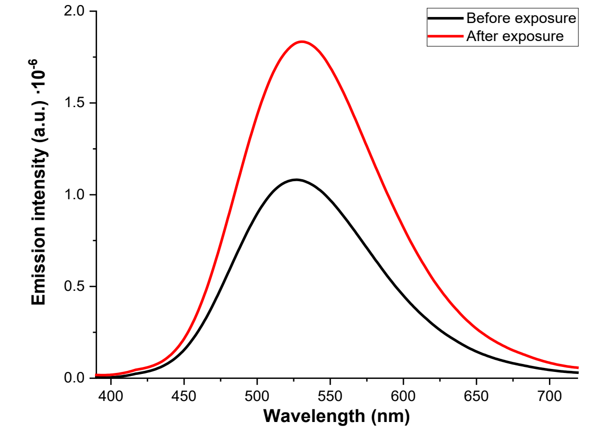	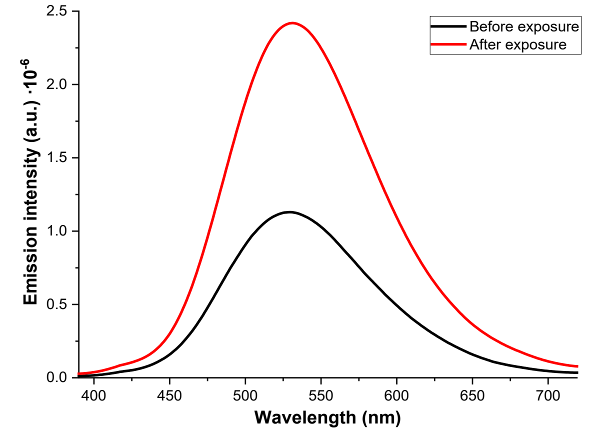

50 cm	100 cm
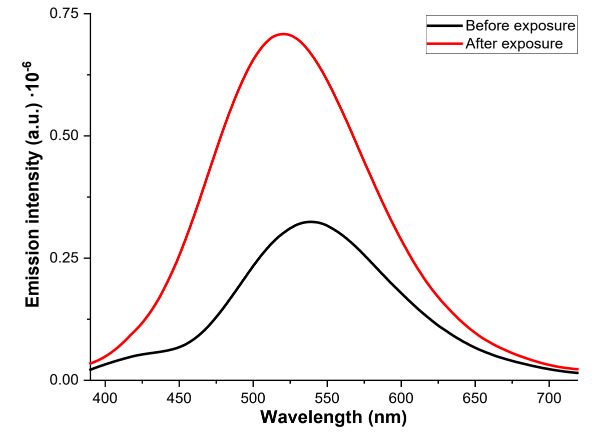	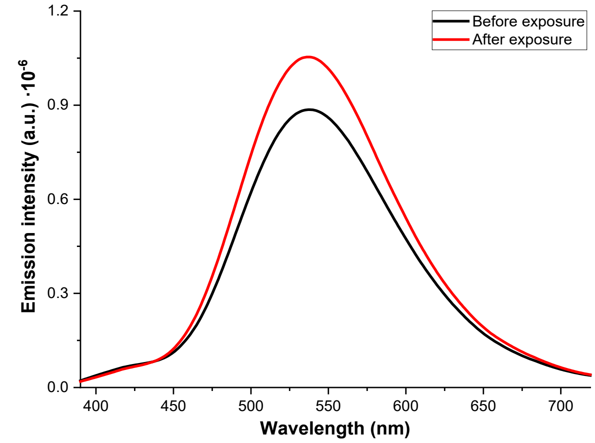

150 cm	200 cm
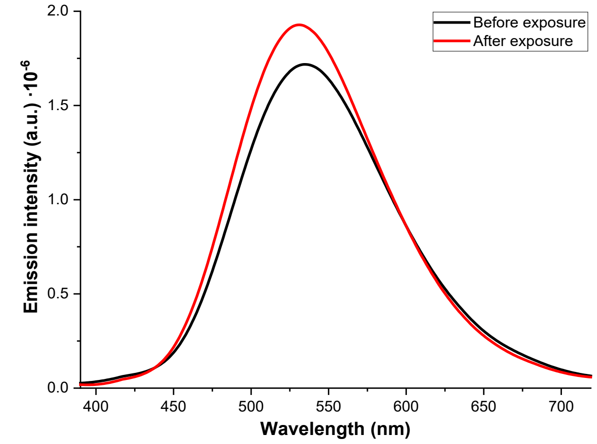	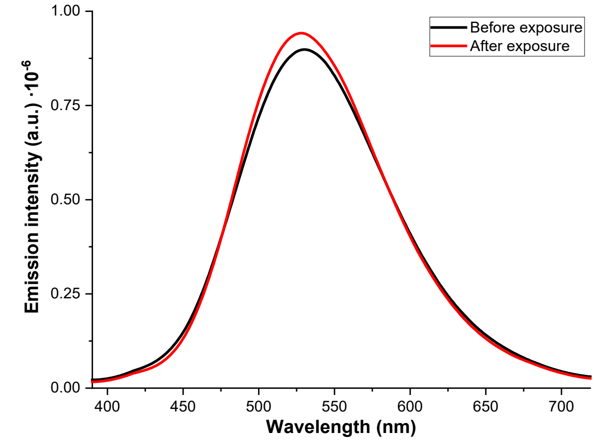

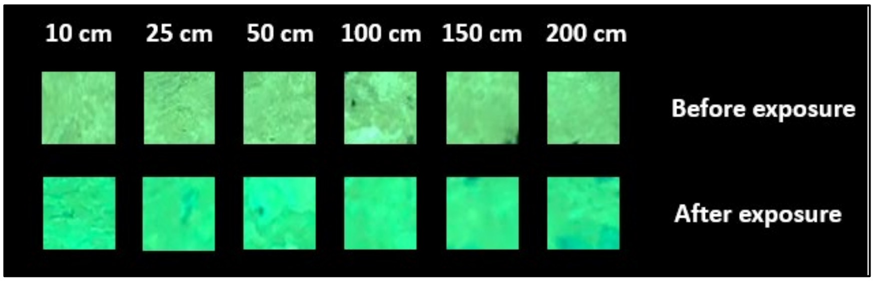

Figure S79. Comparative photograph taken under 366 nm light before and after exposure to TATP vapors at 26ºC during 30 minutes.

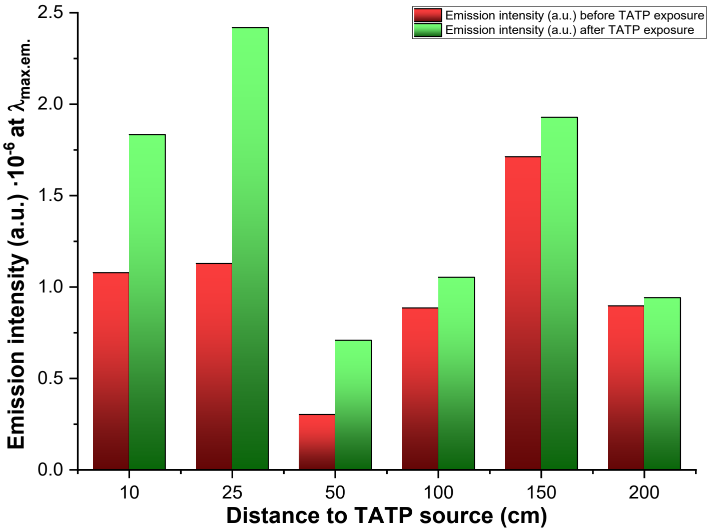

Figure S80. Histogram plotting the peak emission before and after exposure to TATP vapors at 26ºC during 30 minutes.

The average increase in the emission intensity for particles were located 50 cm or less from the TATP source was 206%, while the increase for the emission intensity for nanoparticles were located one meter or more was only 112%.

Figure S81. Increment in fluorescence emission intensity of sensing nanoparticles as a function of their distance from the TATP source.

**Sensing AR82s·SiO_2 particles position**	**Increase in emission intensity**
10 cm	170%
25 cm	214%
50 cm	234%
100 cm	119%
150 cm	113%
200 cm	105%

Experiment 8: at 26 ºC during 30 minutes.

Of the 256 mg of TATP initially weighed, only 23 mg were evaporated under the temperature and time conditions used in this experiment, remaining 233 mg of TATP as solid in this case too.

Figure S82. Emission spectra of the nanoparticles before and after exposure to TATP vapors at 26 ºC during 30 minutes.

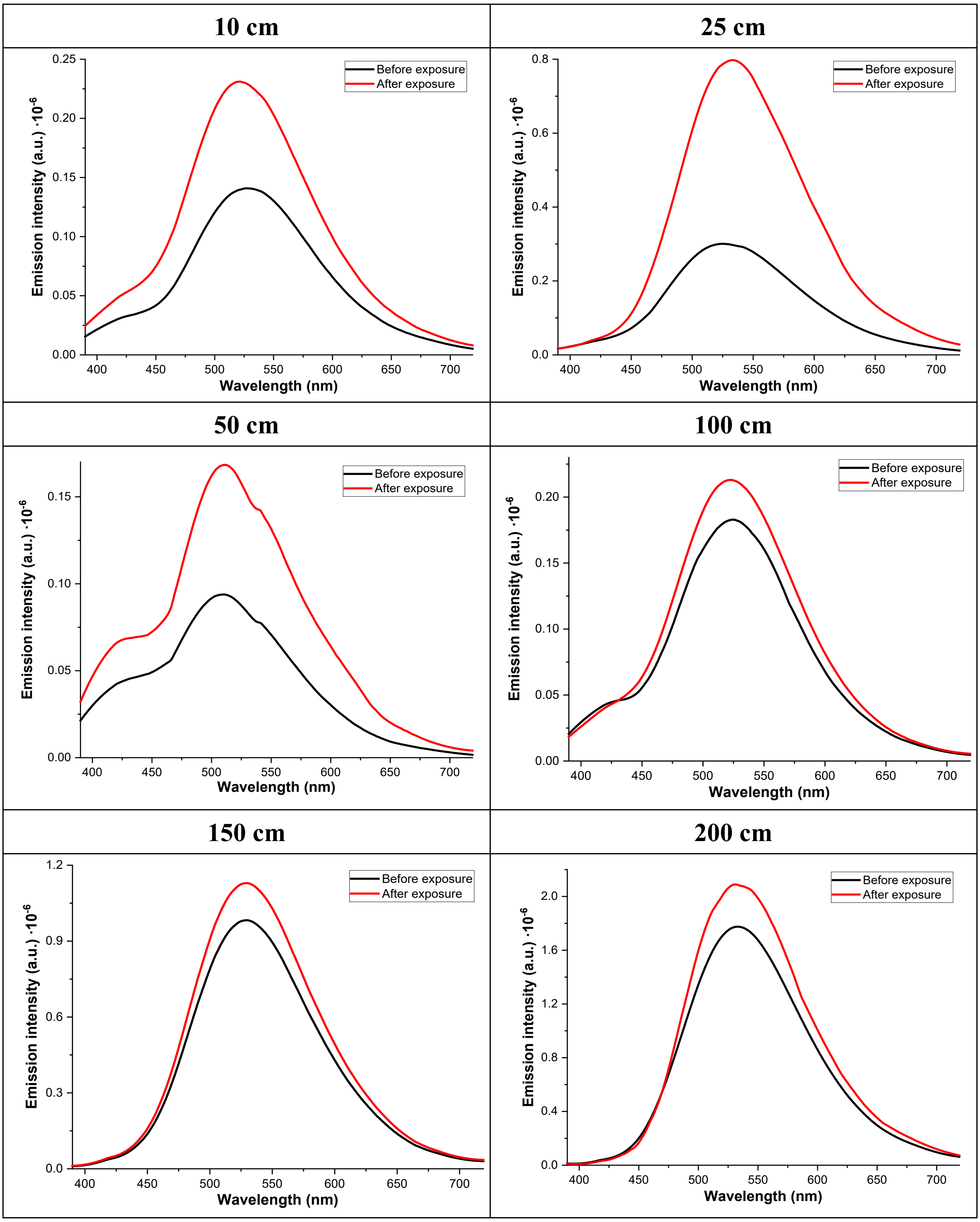

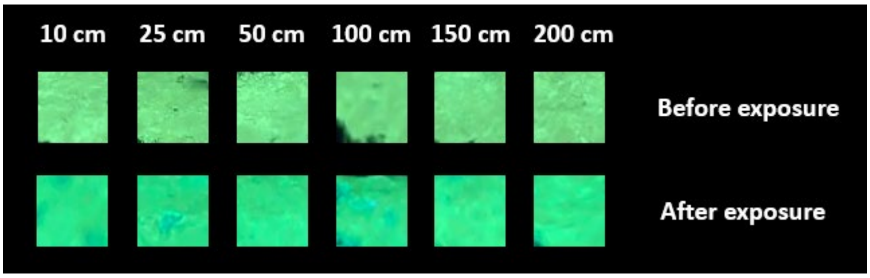

Figure S83. Comparative photograph taken under 366 nm light before and after exposure to TATP vapors at 26ºC during 30 minutes.

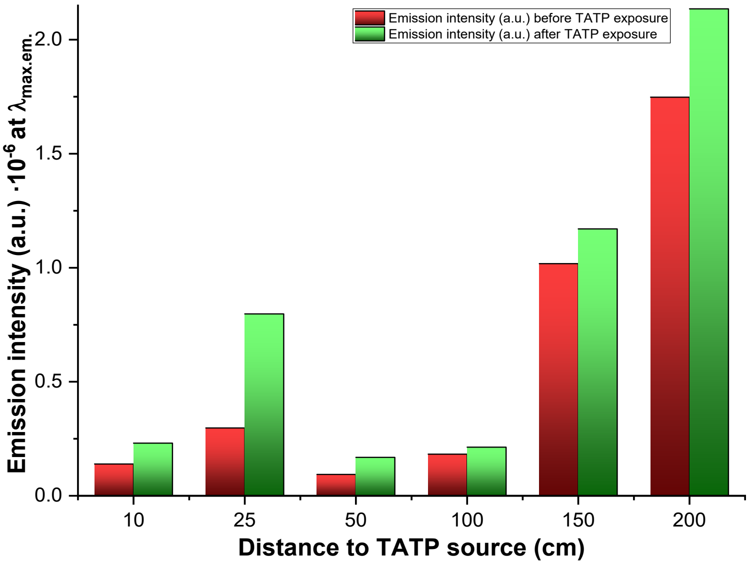

Figure S84. Histogram plotting the peak emission before and after exposure to TATP vapors at 26ºC during 30 minutes.

The average increase in the emission intensity for particles were located 50 cm or less from the TATP source was 205%, while the increase for the emission intensity for nanoparticles were located one meter or more was only 118%.

Figure S85. Increment in fluorescence emission intensity of sensing nanoparticles as a function of their distance from the TATP source.

**Sensing AR82s·SiO_2 particles position**	**Increase in emission intensity**
10 cm	166%
25 cm	268%
50 cm	180%
100 cm	117%
150 cm	115%
200 cm	122%

Experiment 9: at 26 ºC during 30 minutes.

Of the 251 mg of TATP initially weighed, only 36 mg were evaporated under the temperature and time conditions used in this experiment, remaining 215 mg of TATP as solid.

Figure S86. Emission spectra of the nanoparticles before and after exposure to TATP vapors at 26ºC during 30 minutes.

10 cm	25 cm
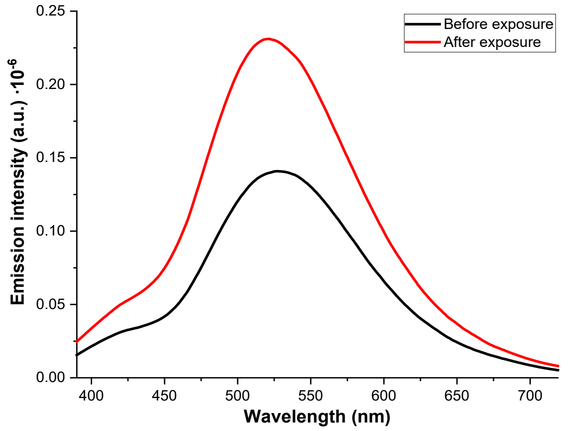	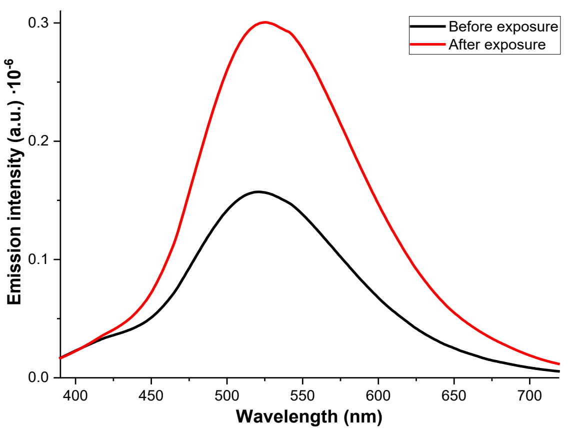
**50 cm**	**100 cm**
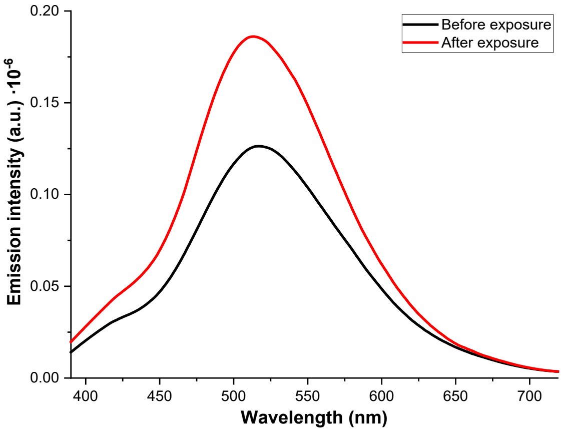	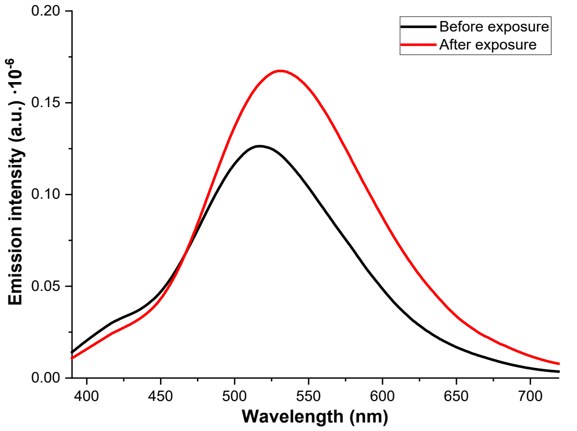
**150 cm**	**200 cm**
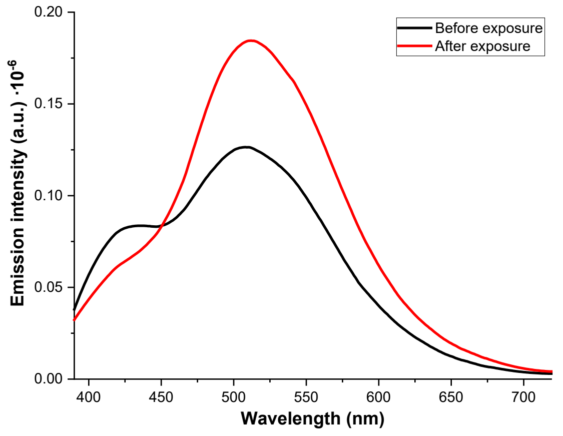	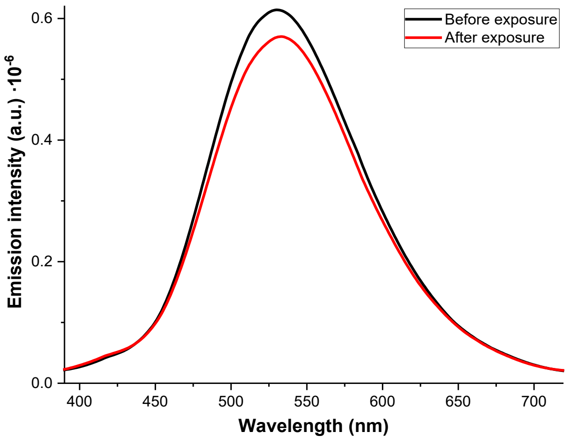

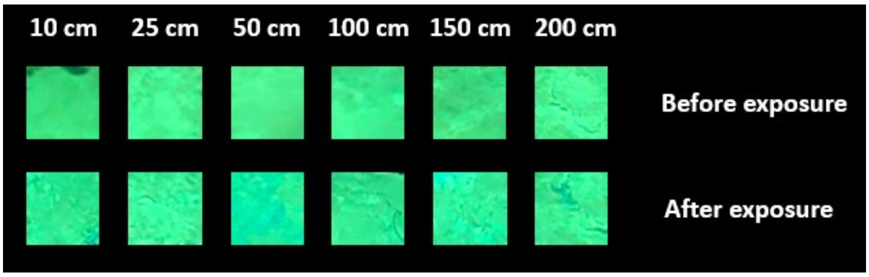

Figure S87. Comparative photograph taken under 366 nm light before and after exposure to TATP vapors at 26ºC during 30 minutes.

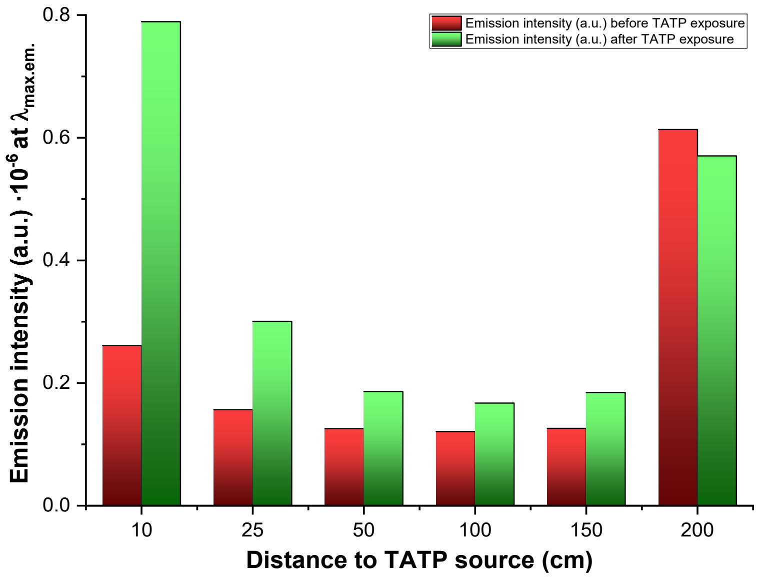

Figure S88. Histogram plotting the peak emission before and after exposure to TATP vapors at 26ºC during 30 minutes.

The average increase in the emission intensity for particles were located 50 cm or less from the TATP source was 214%, while the increase for the emission intensity for nanoparticles were located one meter or more was only 126%.

Figure S89. Increment in fluorescence emission intensity of sensing nanoparticles as a function of their distance from the TATP source.

Sensing AR82s·SiO_2 particles position	Increase in emission intensity
10 cm	302%
25 cm	192%
50 cm	148%
100 cm	138%
150 cm	146%
200 cm	93%

Experiment 10: at 26 ºC during 30 minutes.

Of the 250 mg of TATP initially weighed, only 36 mg were evaporated under the temperature and time conditions used in this experiment, remaining 214 mg of TATP as solid.

Figure S90. Emission spectra of the nanoparticles before and after exposure to TATP vapors at 26ºC during 30 minutes.

10 cm	25 cm
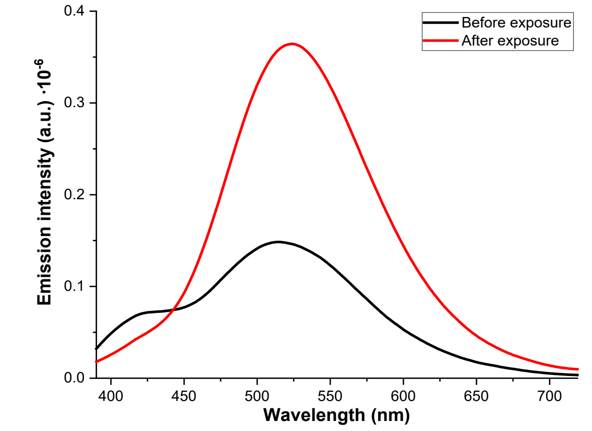	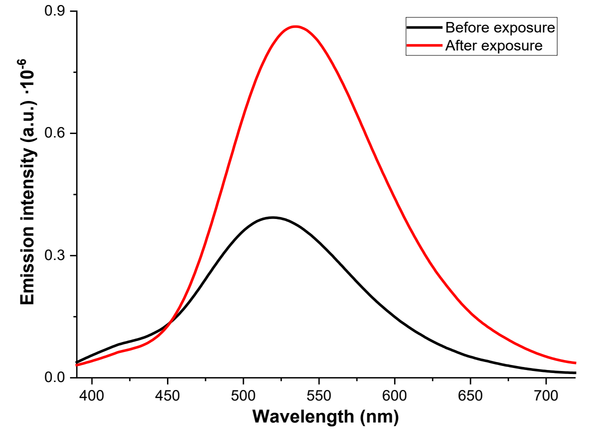
**50 cm**	**100 cm**
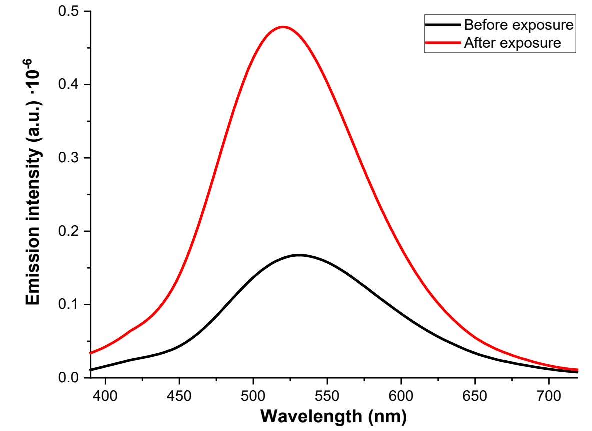	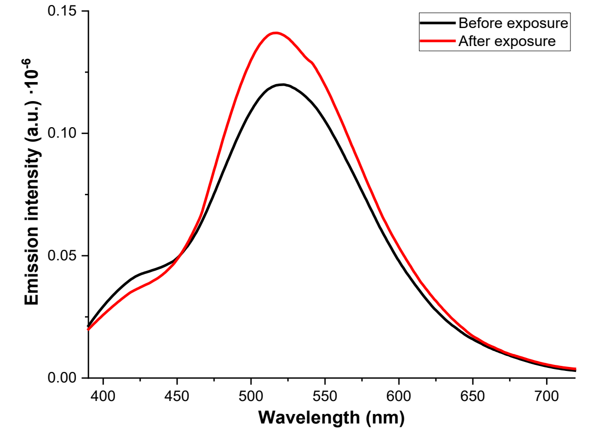
**150 cm**	**200 cm**
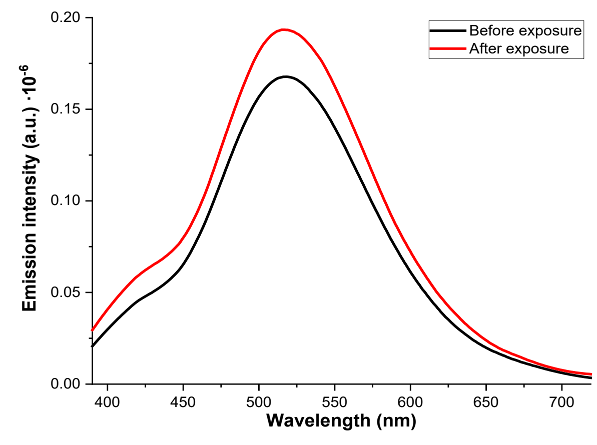	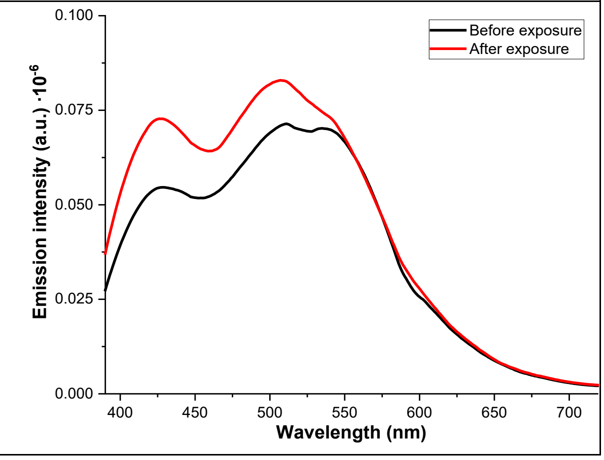

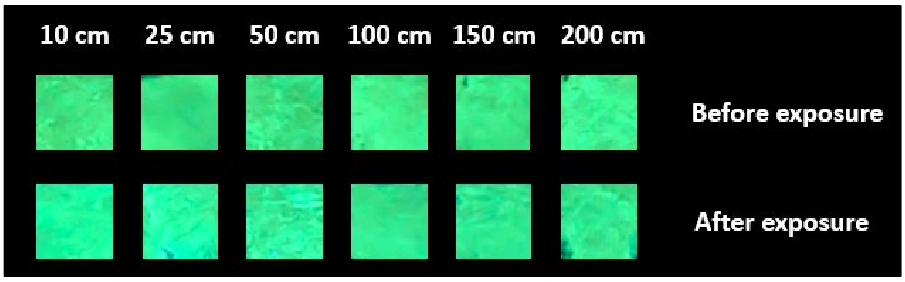

Figure S91. Comparative photograph taken under 366 nm light before and after exposure to TATP vapors at 26ºC during 30 minutes.

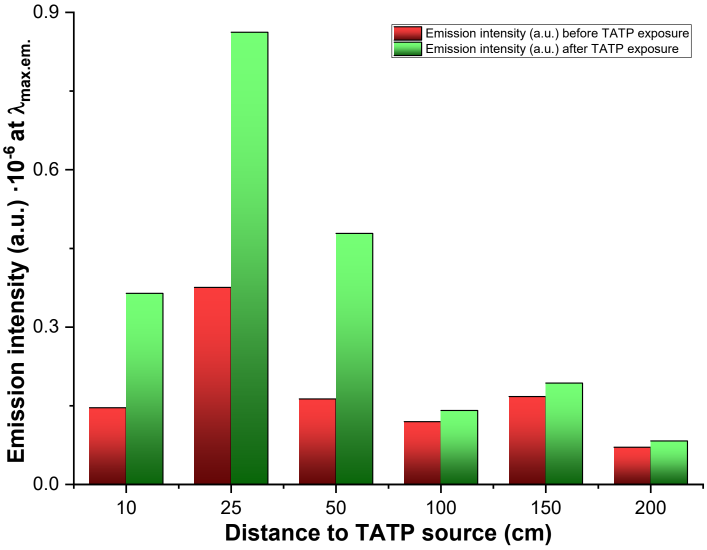

Figure S92. Histogram plotting the peak emission before and after exposure to TATP vapors at 26ºC during 30 minutes.

The average increase in the emission intensity for particles were located 50 cm or less from the TATP source was 257%, while the increase for the emission intensity for nanoparticles were located one meter or more was only 117%.

Figure S93. Increment in fluorescence emission intensity of sensing nanoparticles as a function of their distance from the TATP source.

**Sensing AR82s·SiO_2 particles position**	**Increase in emission intensity**
10 cm	249%
25 cm	229%
50 cm	293%
100 cm	118%
150 cm	115%
200 cm	117%

Experiment 11: at 26 ºC during 30 minutes.

Of the 254 mg of TATP initially weighed, only 32 mg were evaporated under the temperature and time conditions used in this experiment, remaining 222 mg of TATP as solid.

Figure S94. Emission spectra of the nanoparticles before and after exposure to TATP vapors at 26ºC during 30 minutes.

10 cm	25 cm
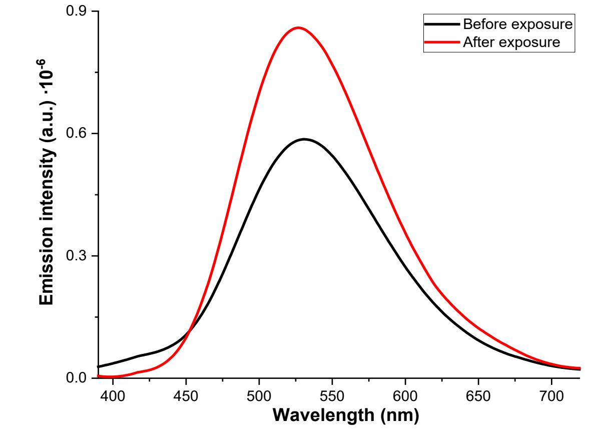	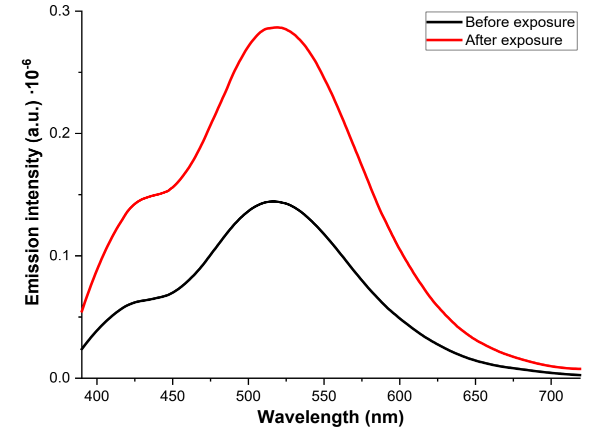
**50 cm**	**100 cm**
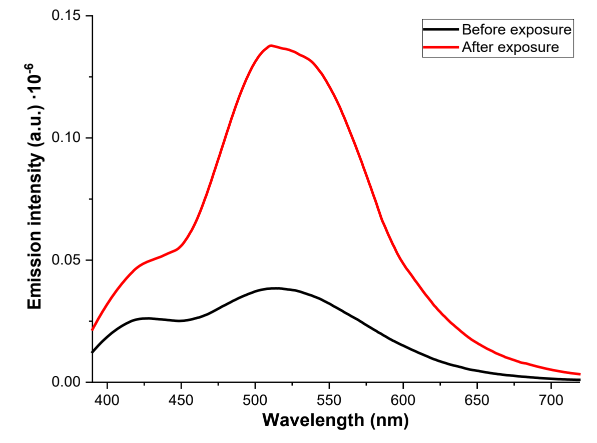	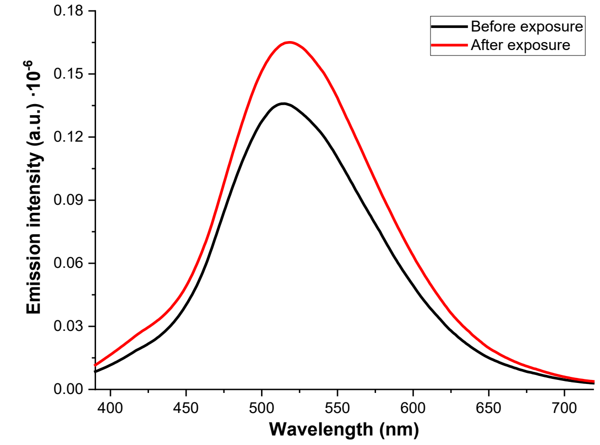
**150 cm**	**200 cm**
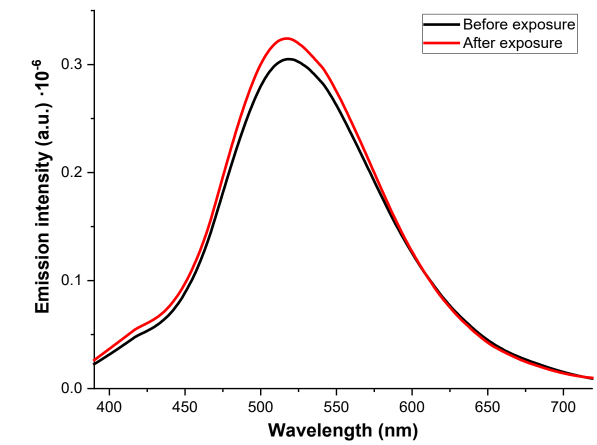	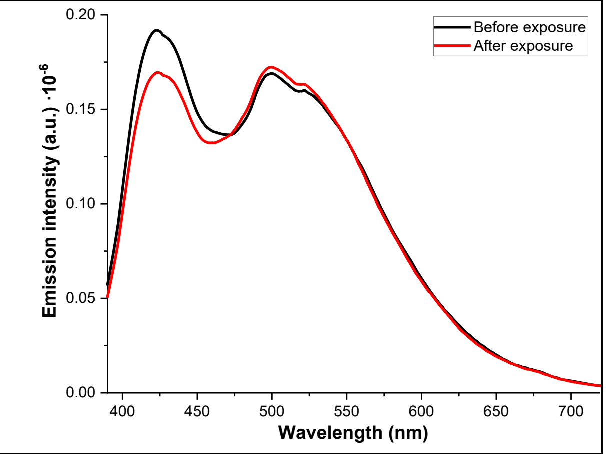

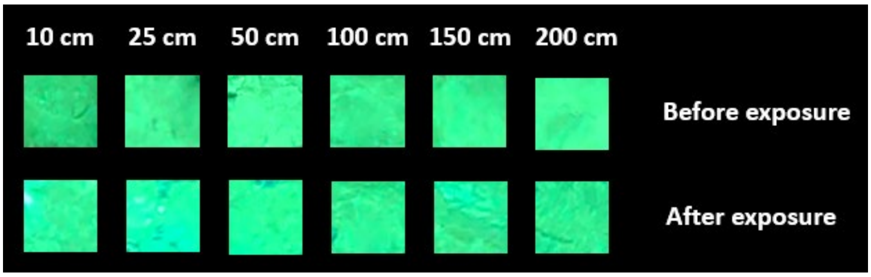

Figure S95. Comparative photograph taken under 366 nm light before and after exposure to TATP vapors at 26ºC during 30 minutes.

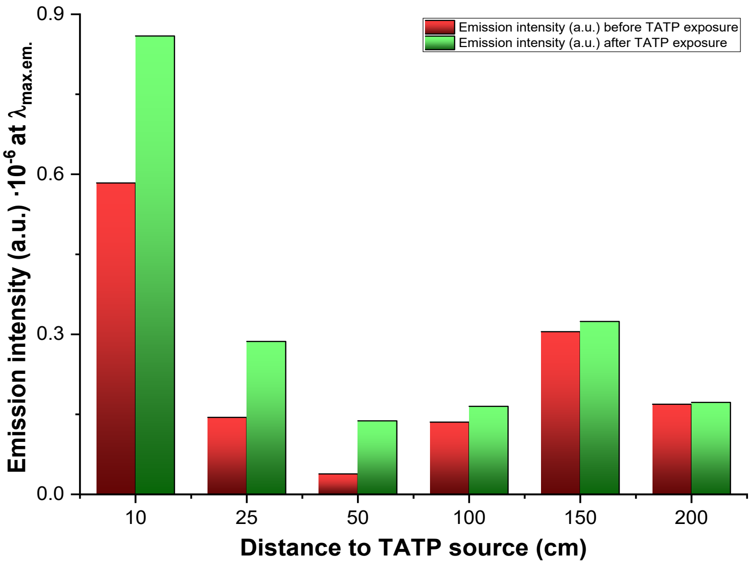

Figure S96. Histogram plotting the peak emission before and after exposure to TATP vapors at 26ºC during 30 minutes.

The average increase in the emission intensity for particles were located 50 cm or less from the TATP source was 235%, while the increase for the emission intensity for nanoparticles were located one meter or more was only 110%.

Figure S97. Increment in fluorescence emission intensity of sensing nanoparticles as a function of their distance from the TATP source.

**Sensing AR82s·SiO_2 particles position**	**Increase in emission intensity**
10 cm	147%
25 cm	199%
50 cm	359%
100 cm	122%
150 cm	106%
200 cm	102%

Experiment 12: at 26 ºC during 30 minutes.

Of the 254 mg of TATP initially weighed, only 32 mg were evaporated under the temperature and time conditions used in this experiment, remaining 222 mg of TATP as solid.

Figure S98. Emission spectra of the nanoparticles before and after exposure to TATP vapors at 26ºC during 30 minutes.

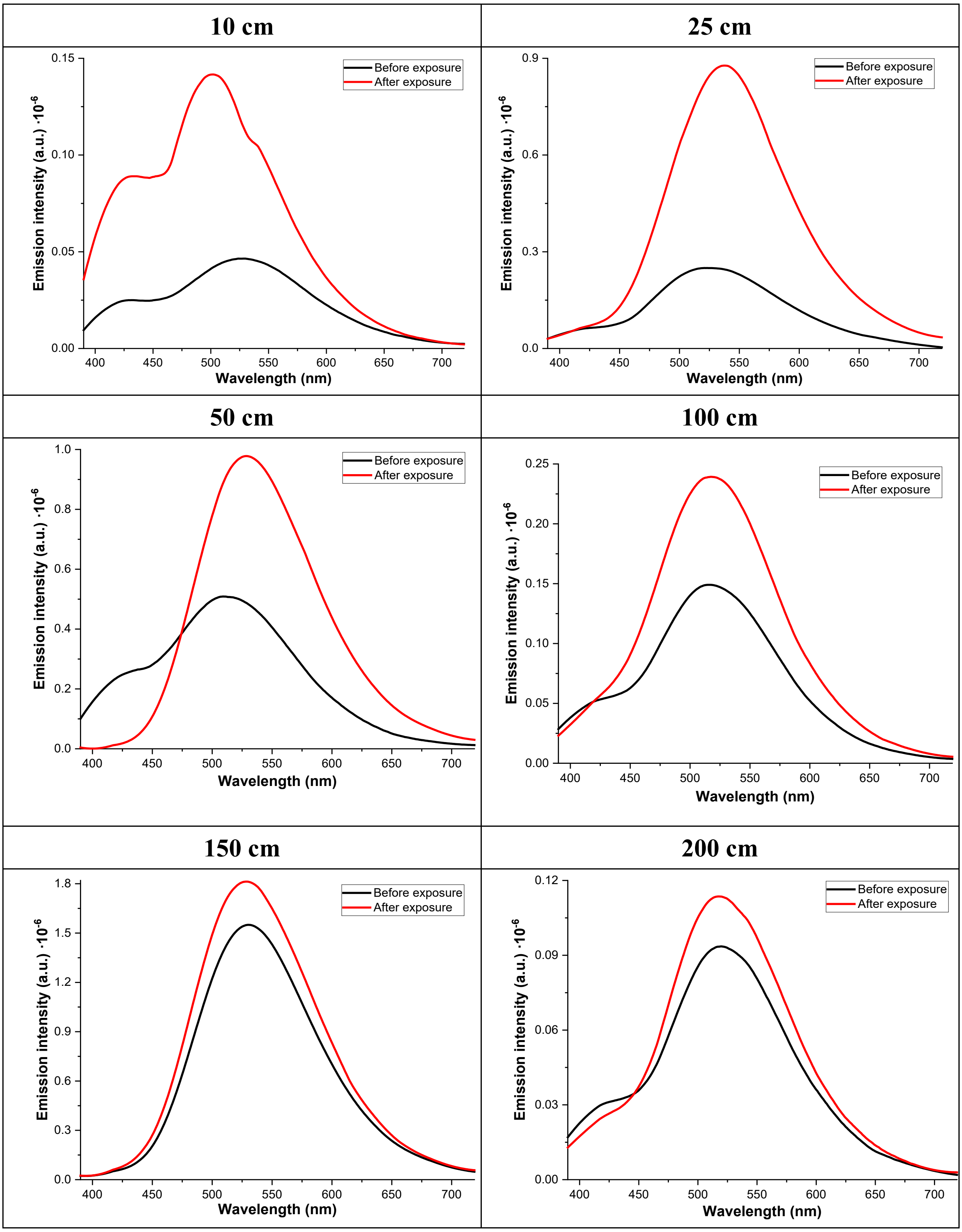

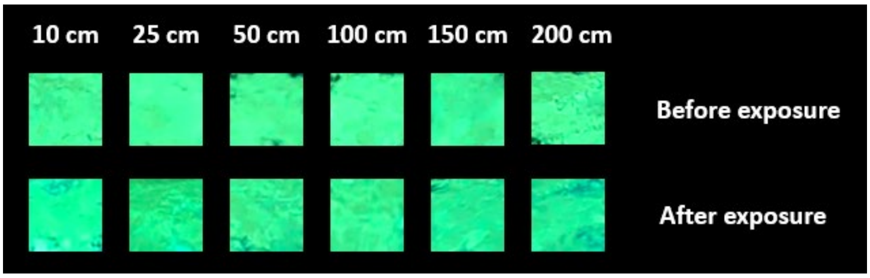

Figure S99. Comparative photograph taken under 366 nm light before and after exposure to TATP vapors at 26ºC during 30 minutes.

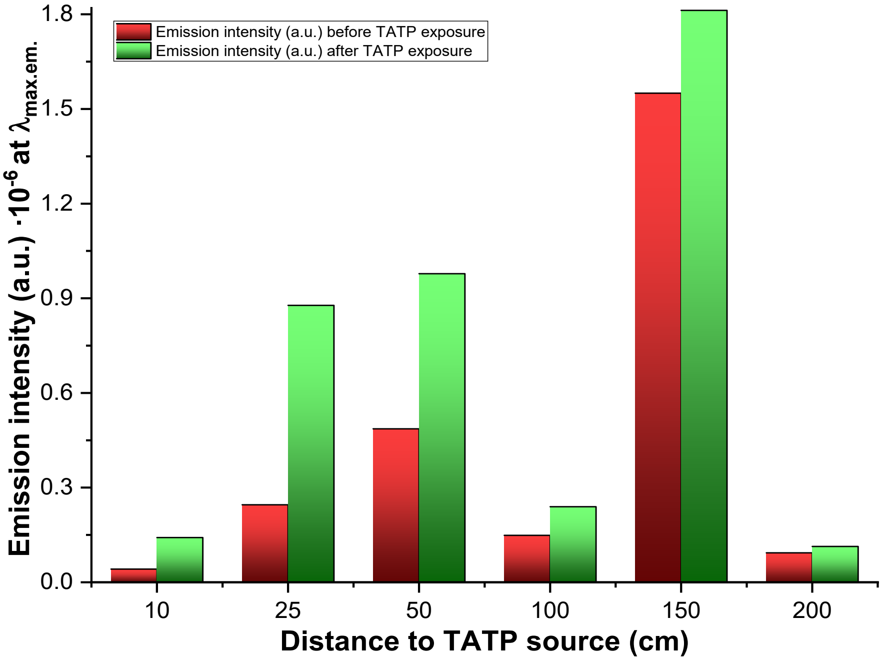

Figure S100. Histogram plotting the peak emission before and after exposure to TATP vapors at 26ºC during 30 minutes.

The average increase in the emission intensity for particles were located 50 cm or less from the TATP source was 299%, while the increase for the emission intensity for nanoparticles were located one meter or more was only 133%.

Figure S101. Increment in fluorescence emission intensity of sensing nanoparticles as a function of their distance from the TATP source.

Sensing AR82s·SiO_2 particles position	Increase in emission intensity
10 cm	338%
25 cm	357%
50 cm	201%
100 cm	161%
150 cm	117%
200 cm	122%

For an easier visualization of the whole data, the following graphs have been made, representing the normalized emission intensity variation before and after exposure to TATP vapors versus distance to the TATP source.

Figure S102. Summary of experiments 7 – 12 with TATP vapor at 26ºC. Emission intensity variation before and after exposure to TATP (left) and emission intensity proportionality factor (divided between the target, right) versus distance to the TATP source in both cases.

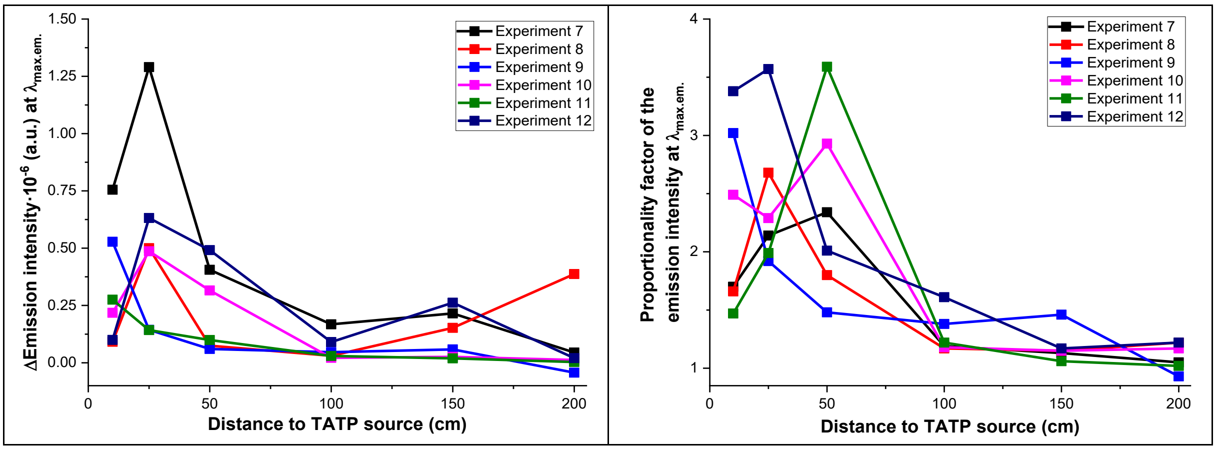

Maximum emission intensity data from previous experiments were organized according to the distance to the TATP source, representing, on the one hand, emission before contact with TATP vapors and, on the other hand, emission after contact with said vapors. These data were also normalized with respect to the maximum of emission from each experiment.

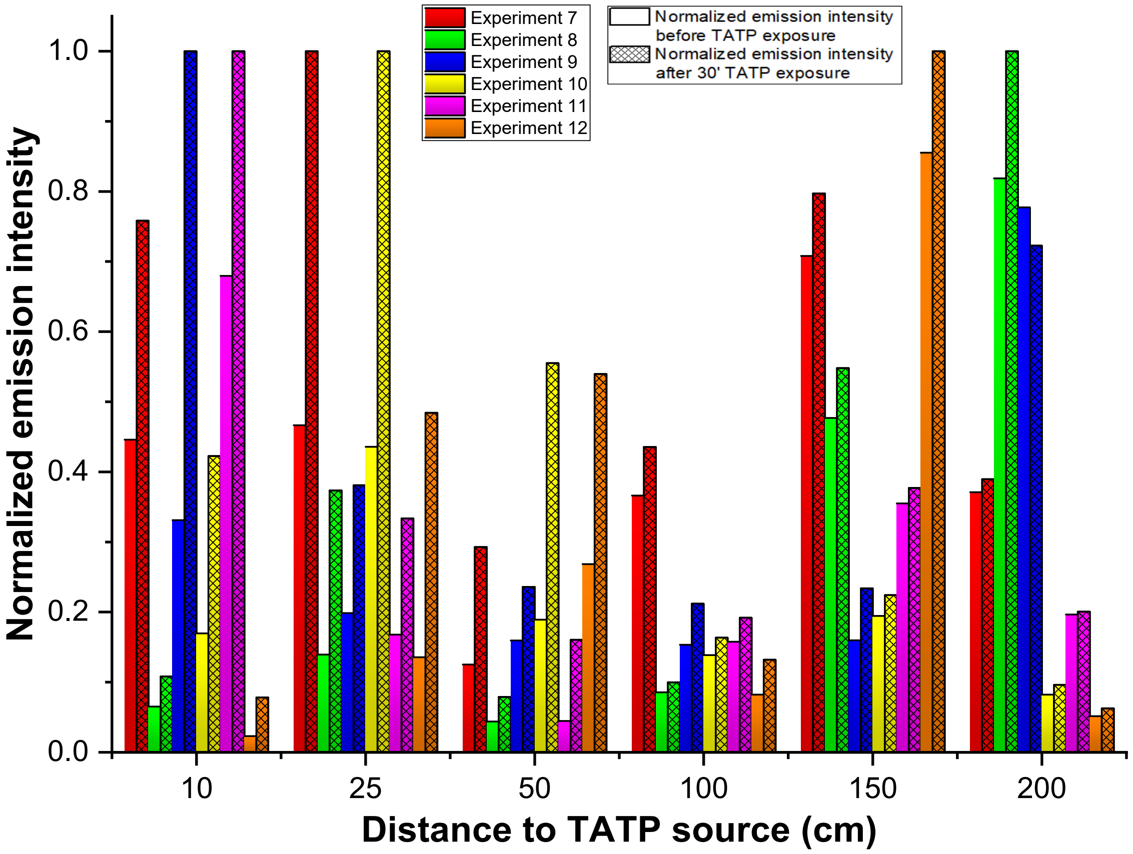

Figure S103. Summary of experiments 7 – 12. Normalized emission intensity variation before and after exposure to TATP versus distance to the TATP source.

After this, a common initial emission level was established using the lowest value measured for the nanoparticles prior to the exposure to TATP vapors. And finally, the subtraction was made between the final and initial emission.

Figure S104. Summary of experiments 7 – 12. Normalized emission intensity setting a common initial particulate emission level before and after exposure to TATP vapors (left) and variation of the normalized emission intensity (right) as a function of the distance to the TATP source in both cases.

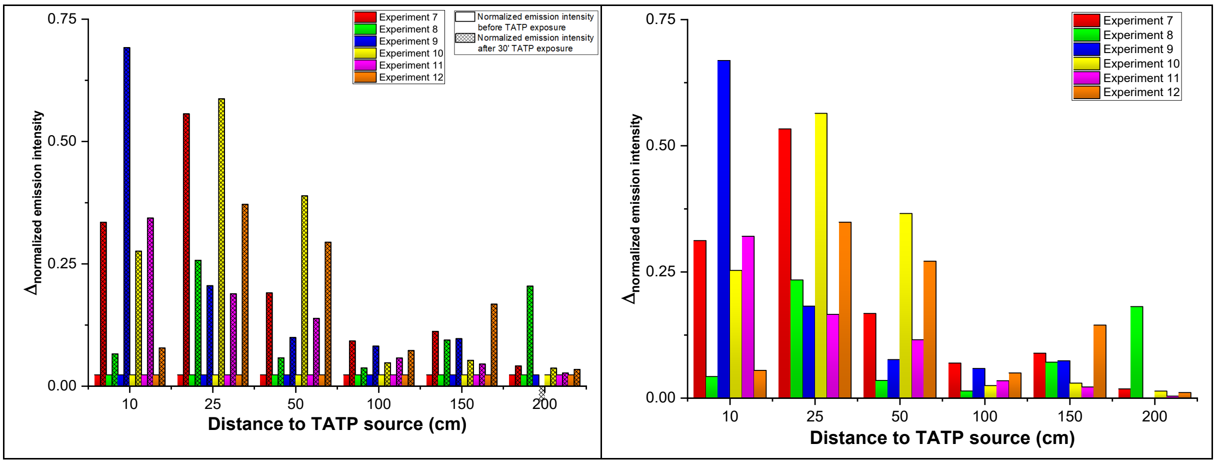

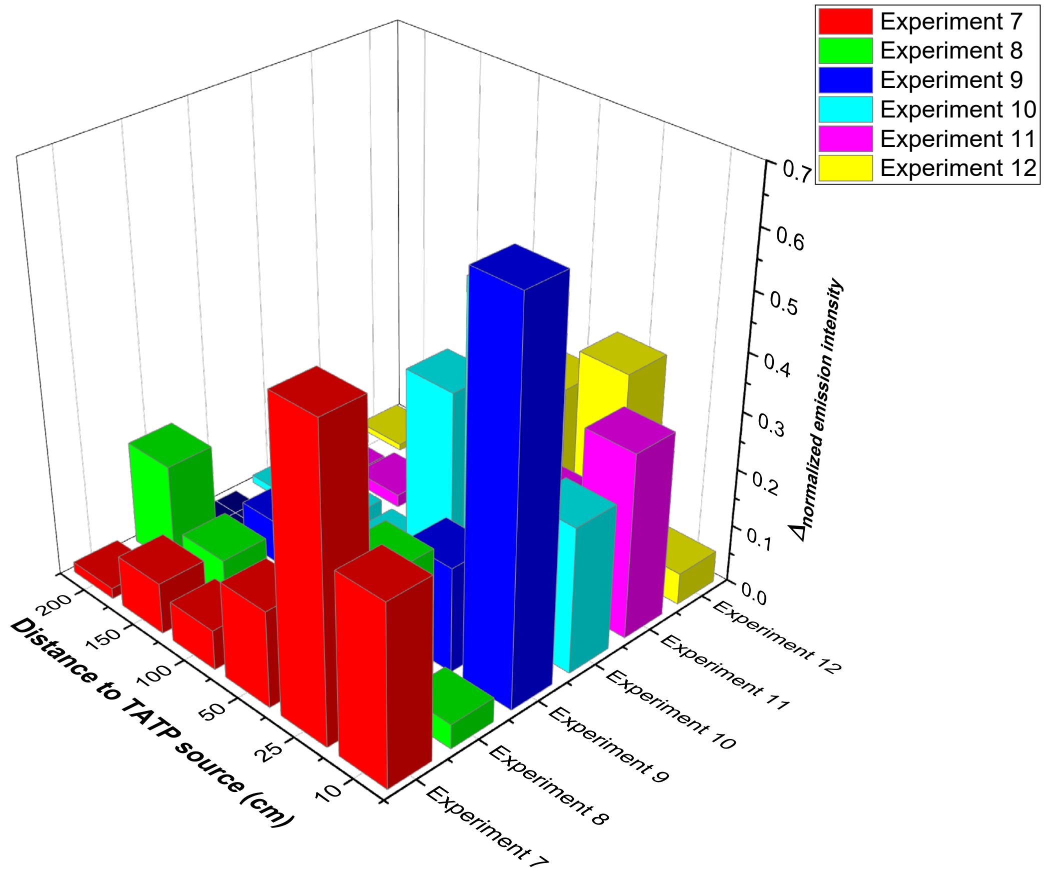

Figure S105. Summary of experiments 7 – 12. 3D representation of the variation of the normalized emission intensity as a function of the distance to the TATP source.

A selection of the measurements was made, discarding outliers and choosing only 3 measurements for each of the distances to the TATP source. Using these measurements, the average and standard deviation of the normalized variation of the emission intensity for each point were calculated and represented.

Figure S106. Values of variation of normalized the emission intensity as a function of the distance to the TATP source.

Distance (cm)	Experiment					
	7	8	9	10	11	12
10	**0.312**	0.043	**0.669**	0.252	**0.321**	0.055
25	0.533	0.234	**0.182**	**0.564**	**0.166**	0.349
50	**0.168**	0.035	0.076	0.366	**0.116**	**0.271**
100	**0.069**	0.014	**0.059**	0.025	0.035	**0.050**
150	0.089	0.071	**0.074**	**0.029**	**0.022**	0.145
200	**0.018**	0.181	-0.054	**0.014**	0.004	**0.011**

Figure S107. Values of the average and standard deviation of normalized emission variation as a function of the distance to the TATP source.

Distance (cm)	Average normalized emission variation	Standard deviation
10	0.434	0.203
25	0.304	0.225
50	0.185	0.079
100	0.059	0.011
150	0.042	0.028
200	0.014	0.004

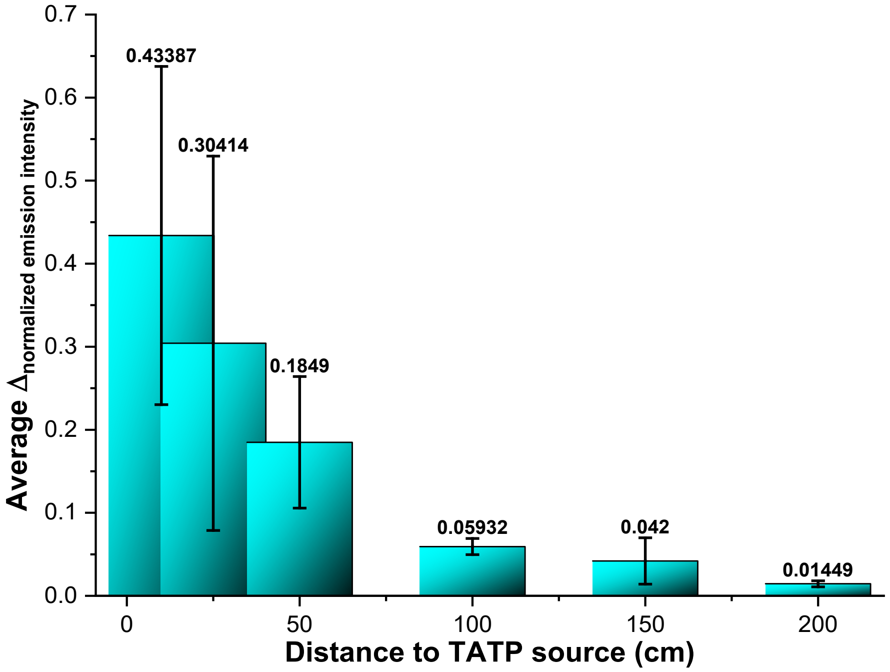

Figure S108. Average and standard deviation of normalized emission variation as a function of the distance to the TATP source.

^{1}H-NMR titration of AR82s with TATP in the presence of Amberlite:

An NMR titration was performed. Initially, ^{1}H-NMR of TATP (5 mg in 0.5 ml in $CDCl_3$), AR82s (3 mg in 0.5 ml in $CDCl_3$) and Amberlite (1.5 mg) were performed as a reference. After this, the following amounts of TATP were added to the NMR tube corresponding to AR82s to carry out the titration.

Figure S109. Additions of TATP to the ^{1}H-NMR titration of AR82s.

**Addition number**	**TATP mass added (µg)**	**Total mass of TATP added (µg)**	**Addition number**	**TATP mass added (µg)**	**Total mass of TATP added (µg)**
**1**	0	0	**10**	60	150
**2**	5	5	**11**	150	300
**3**	5	10	**12**	200	500
**4**	5	15	**13**	300	800
**5**	5	20	**14**	600	1400
**6**	10	30	**15**	1800	3200
**7**	10	40	**16**	3600	6800
**8**	20	60	**17**	3600	10400
**9**	30	90	**18**	5400	15800

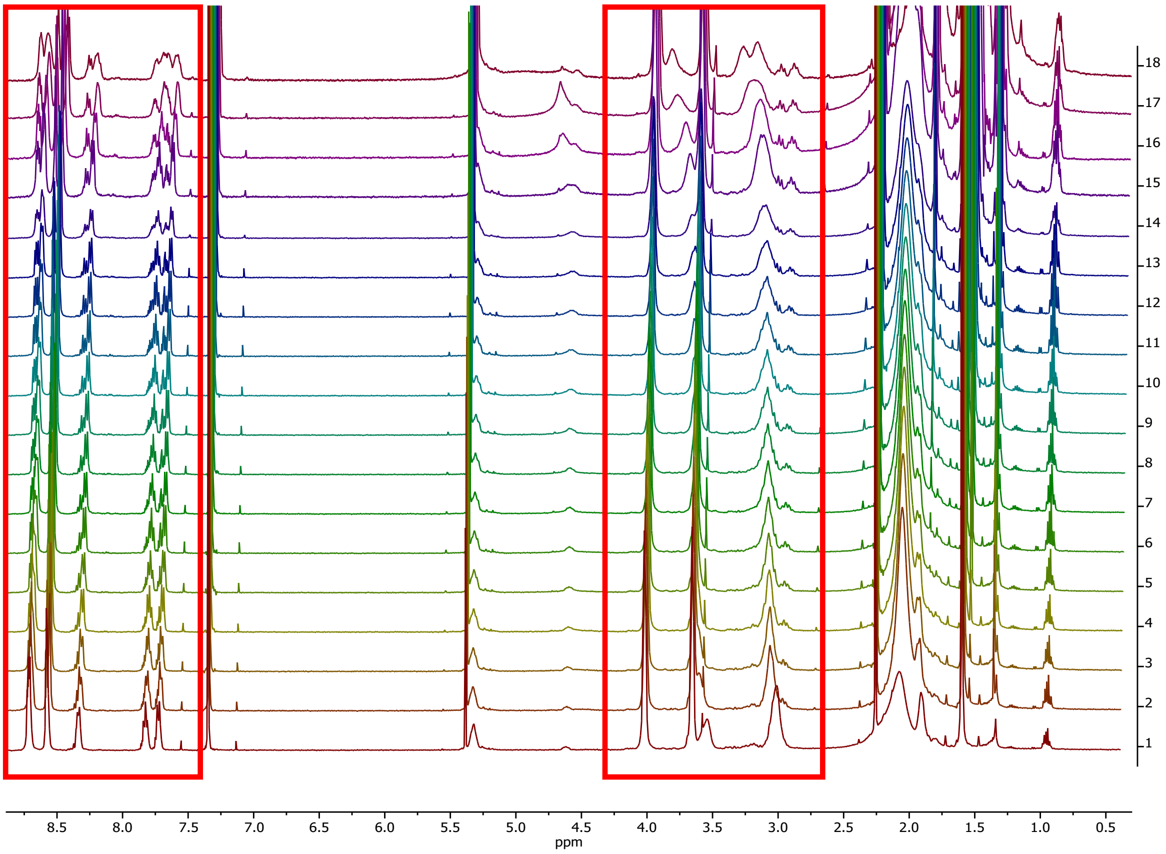

Figure S110. 1H-NMR ($CDCl_3$, 500 MHz) titration of AR82s with increasing amounts of TATP in the presence of amberlite.

pH of AR82s solution was 7 and the pH of AR82s solution with Amberlite was approximately 7, measured using litmus paper.

^{1}H-NMR titration of AR82s with m-CPBA

The mechanism of oxidation of AR82s in the presence of increasing amounts of peroxide was studied by NMR titration by using m-CPBA.

Addition number	m-CPBA mass added (µg)	Total mass of m-CPBA added (µg)	Addition number	m-CPBA mass added (µg)	Total mass of m-CPBA added (µg)
**1**	0	0	**9**	30	90
**2**	5	5	**10**	60	150
**3**	5	10	**11**	150	300
**4**	5	15	**12**	200	500
**5**	5	20	**13**	300	800
**6**	10	30	**14**	600	1400
**7**	10	40	**15**	1800	3200
**8**	20	60			

Figure S111. Amounts of m-CPBA used in NMR titration.

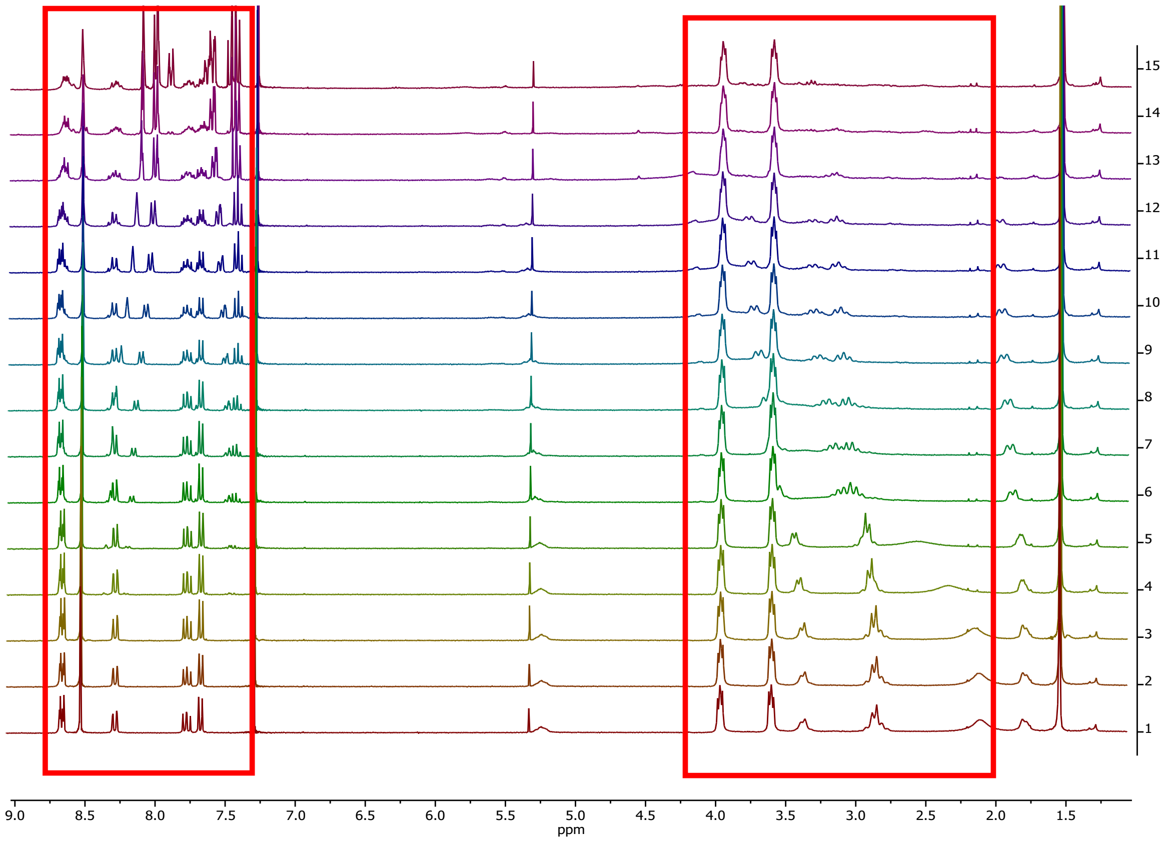

Figure S112. ^{1}H NMR titration. Spectra of 10 mg AR82s in 0.5 ml of $CDCl_3$ with increasing amounts of m-CPBA.

The titration showed oxidation of the unprotected secondary amino group with progressive disappearance of the proton signal of the amine group and chemical shift of the methylene groups of the oxidized piperidine group (the signals between 1.8 and 3.7 ppm).

Synthesis of *N*-(piperidin-4-yl)-4-[2-(piperazin-1-yl)pyrimidin-5-yl]naphthalene-1,8-dicarboxylmonoimide (AR82d)

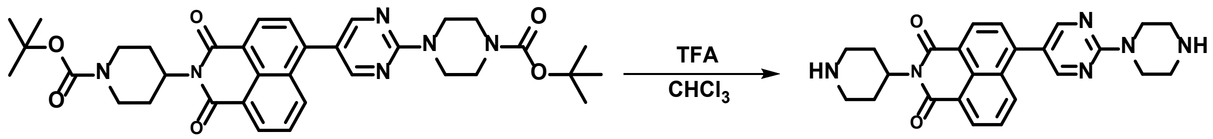

In a 50 ml flask, 150 mg of *N*-(Boc-piperidin-4-yl)-4-[2-(4-Boc-piperazin-1-yl)pyrimidin-5-yl]naphthalene-1,8-dicarboxylmonoimide (AR82p, 0.23 mmol) were dissolved in 15 ml of chloroform and, then, 2 ml of trifluoroacetic acid (26 mmol) were added. After 4 hours under stirring at room temperature, 10 ml of water were poured into the flask and the resulting mixture was neutralized employing a 40% sodium hydroxide solution until it reached a neutral pH. Then, 20 ml of water were added into the flask and the mixture was extracted three times with chloroform (3×30 ml). The organic extracts were combined, dried over anhydrous Na_2SO_4 and the solvent was evaporated under reduce pressure, to obtain 100 mg of AR82d, yellow solid, 98% yield. m.p.: 230 – 233ºC. IR (ATR, cm^{-1}): 3448 (N-H), 3007 – 2501, 1655 (C=O), 1584, 1506, 1449, 1349, 1271, 1236, 1197, 1173 (C-N), 1116, 1041, 948, 828, 781, 720, 535, 505, 432. ^{1}H-NMR (300 MHz, TFA) δ (ppm): 8.72 (m, 2H, 2×H_{Ar}), 8.63 (m, 2H, 2×H_{Ar}), 8.04 (m, 1H, H_{Ar}), 7.74 (m, 2H, 2×H_{Ar}), 5.57 – 5.39 (m, 1H, CH), 4.37 (s, 4H, 2×CH_2), 3.64 (s, 6H, 3×CH_2), 3.36 – 3.09 (m, 4H, 2×CH_2), 2.05 (m, 2H, CH_2). ^{13}C-NMR (75 MHz, TFA) δ (ppm): 169.9 (C=O), 169.5 (C=O), 169.2 (C=O), 168.9 (C=O), 160.0 (C_{Ar}H), 159.3 (C_{Ar}H), 155.6 (C_{Ar}), 154.9 (C_{Ar}), 139.8 (C_{Ar}), 139.2 (C_{Ar}), 137.2 (C_{Ar}H), 136.5 (C_{Ar}H), 135.7 (C_{Ar}H), 135.1 (C_{Ar}H), 134.5 (C_{Ar}H), 133.1 (C_{Ar}), 132.4 (C_{Ar}H), 132.3 (C_{Ar}H), 132.0 (C_{Ar}), 131.9 (C_{Ar}H), 131.6 (C_{Ar}H), 131.4 (C_{Ar}), 126.3 (C_{Ar}), 126.2 (C_{Ar}), 125.6 (C_{Ar}), 125.5 (C_{Ar}), 125.3 (C_{Ar}), 124.7 (C_{Ar}), 52.6 (CH), 51.9 (CH), 49.2 (CH_2), 48.6 (CH_2), 46.9 (CH_2), 46.3 (CH_2), 45.4 (CH_2), 44.7 (CH_2), 44.2 (Cq), 28.0 (CH_2), 27.3 (CH_2). HRMS (MALDI, DCTB+) m/z: calculated for $C_{25}H_{26}N_6O_2$: 443.2190 (M^+ + H); found 443.2193. UV-Vis ($CHCl_3$), λ_{max} nm (log ε): 380 (3.3). Ø (EtOAc, %): 67.62. τ (375 nm, EtOAc, ns): 4.270 (χ^2: 1.015).

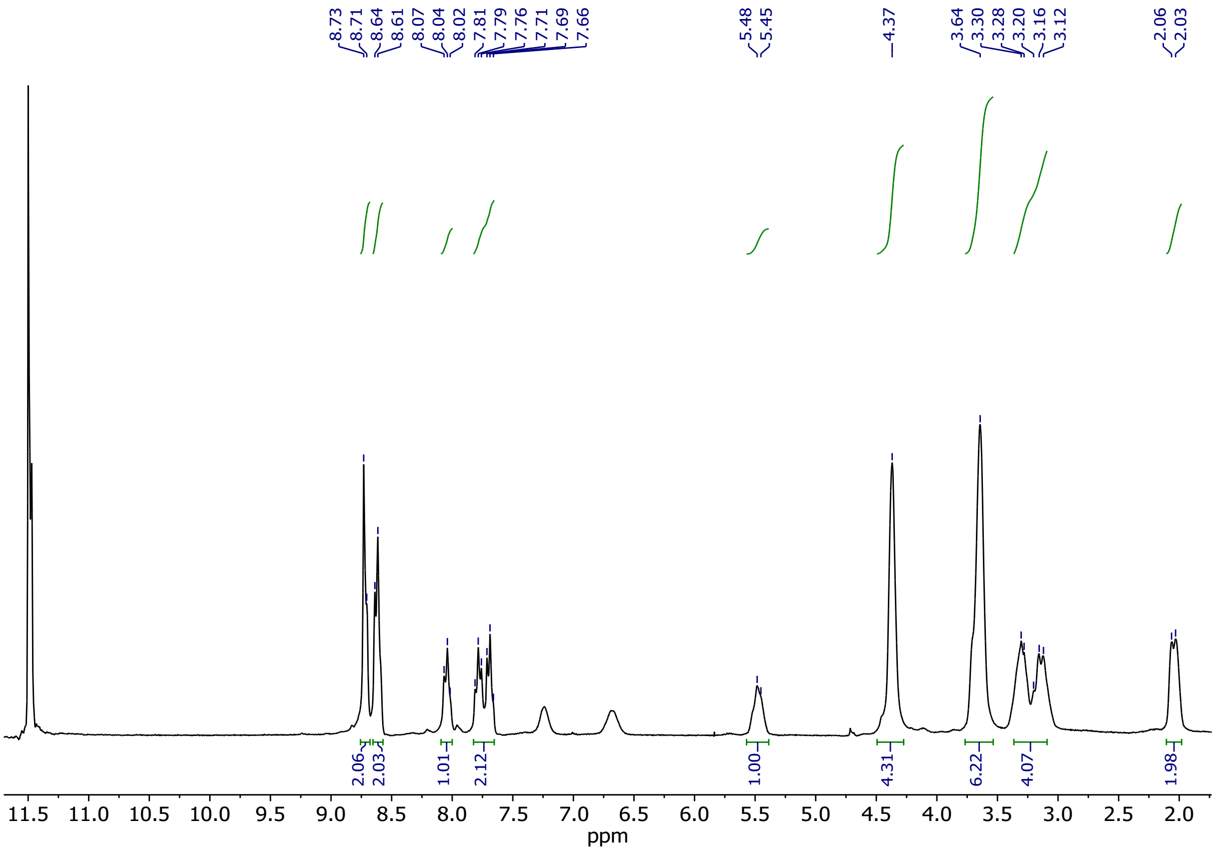

Figure S113. ¹H-NMR (TFA, 300 MHz) of AR82d.

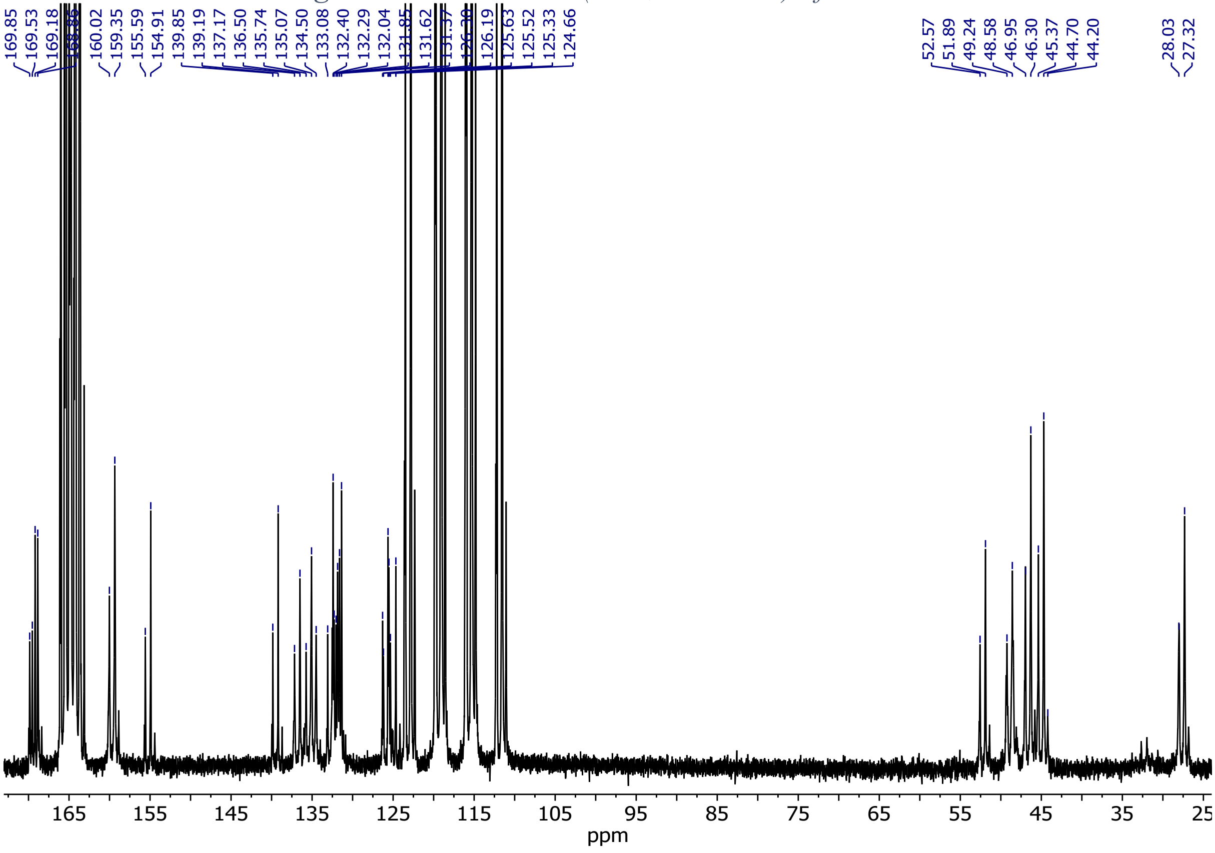

Figure S114. ¹³C-NMR (TFA, 75 MHz) of AR82d.

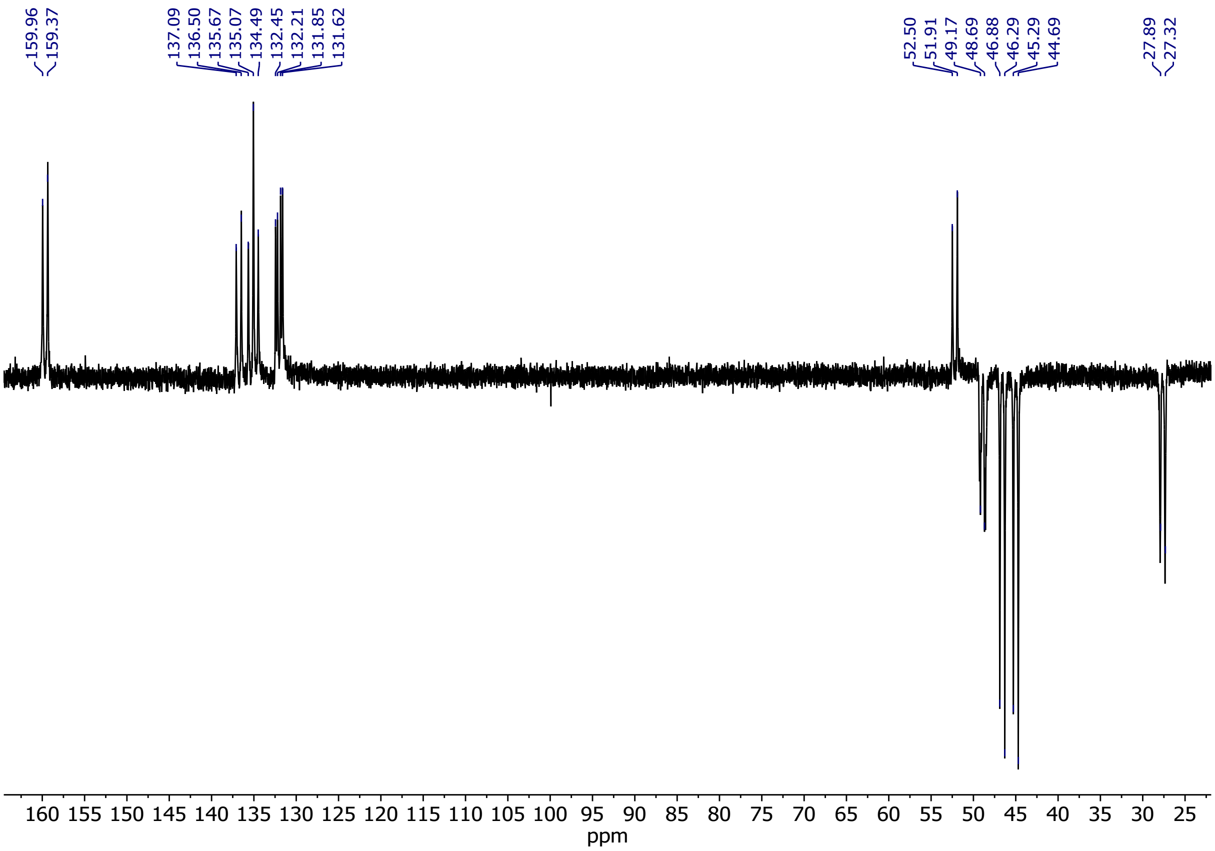

Figure S115. DEPT NMR (TFA, 75 MHz) of AR82d.

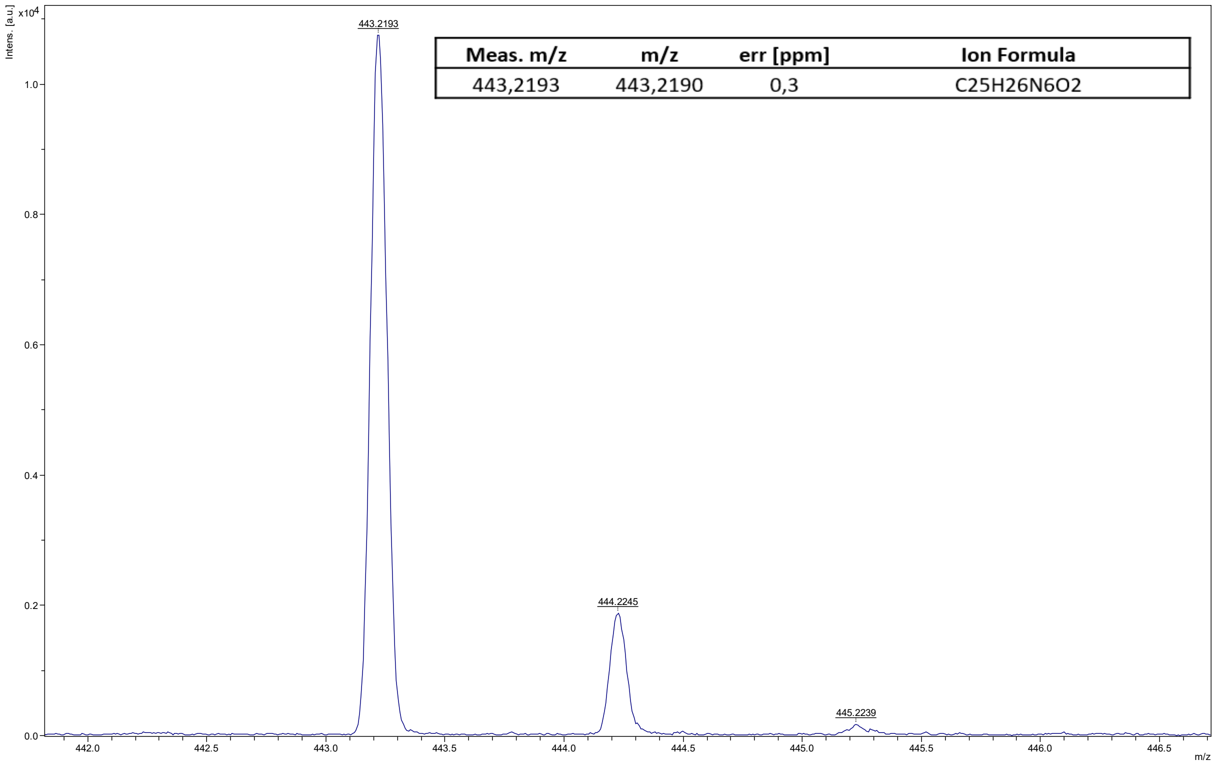

Figure S116. HRMS (MALDI, DCTB+) of AR82d.

Solvatochromism:

The concentration of the compound was 20 µM and the excitation wavelength was 360 nm.

Figure S117. Solvatochromism of AR82d.

Absorbance	Normalized absorbance
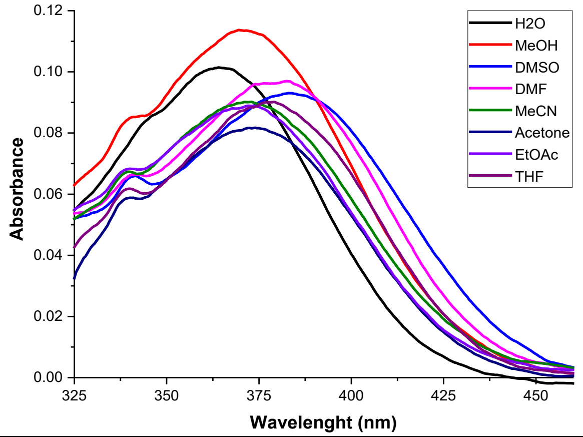	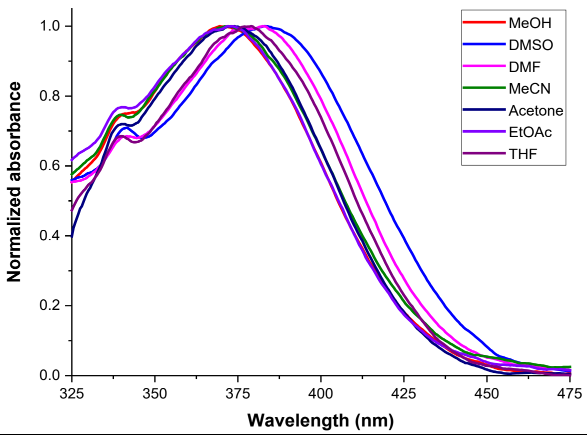
**Emission**	**Normalized emission**
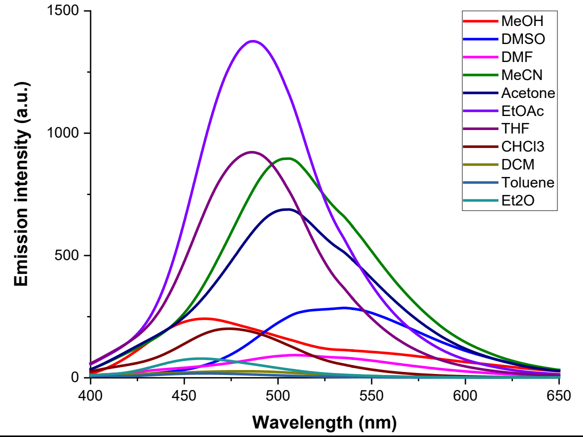	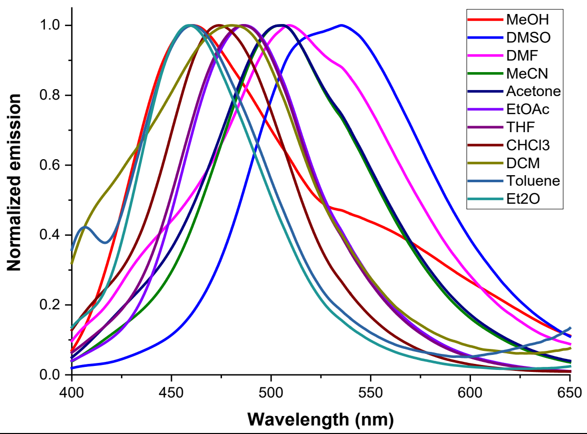

Figure S118. Solvatochromism of AR82d under visible and 366 nm light.

	H_2O MeOH DMSO DMF MeCN Acet. EtOAc THF $CHCl_3$ DCM Tol. Et_2O Hex MCH
**Visible light**	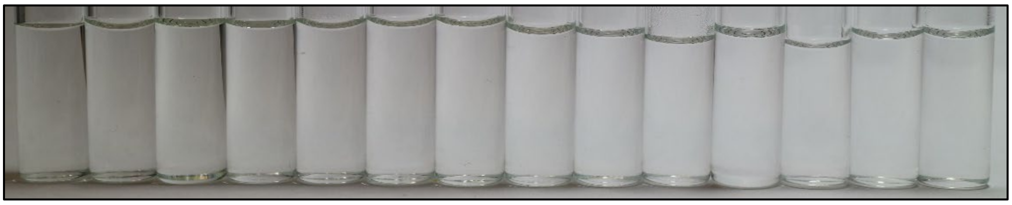
**UV light (366 nm)**	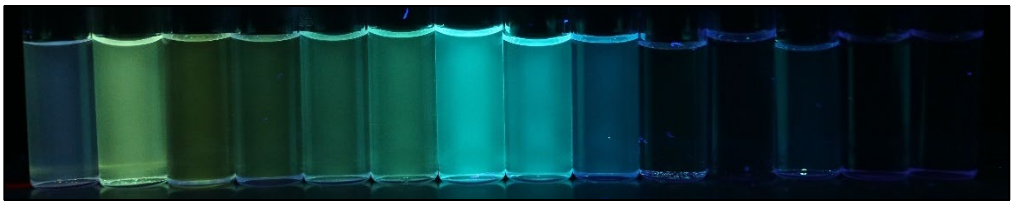

Fluorescence decay lifetime (τ):

The solvent used was ethyl acetate. The concentration of the compound was 20 µM.

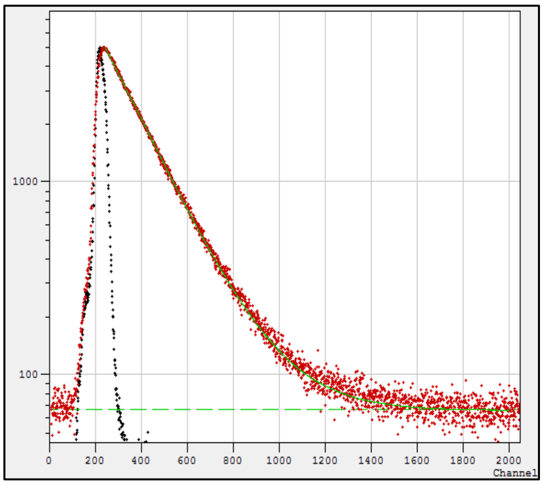

Figure S119. Fluorescence decay lifetime of AR82d.

Water aceptance test:

The solvent used was MeOH and the concentration of the compound was 20 µM.

Figure S120. Water acceptance test of AR82d under visible (up) and UV (down) light.

	**R 5% 10% 20% 30% 40% 50% 60% 70% 80% 90%**
**Visible light**	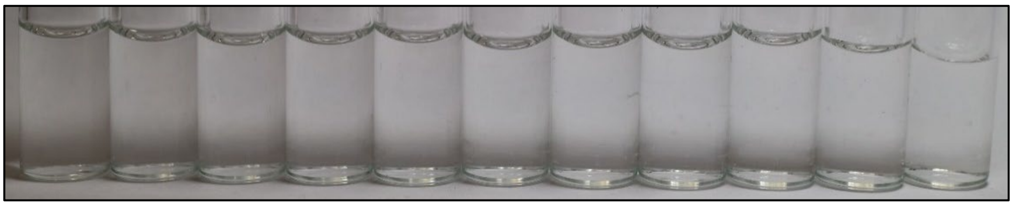
**UV light (366 nm)**	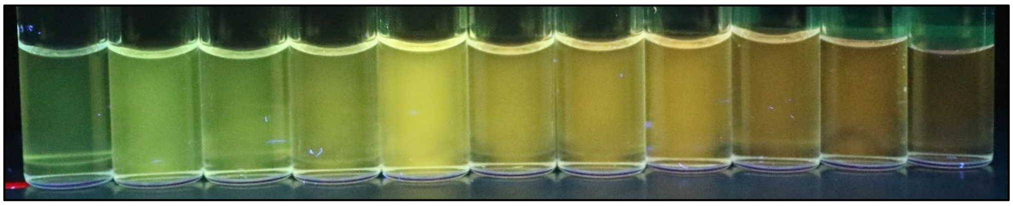

There was a quenching in the fluorescent emission as the amount of water was increased (ACQ).

pH test:

The amount of water that the compound could accept was 30%. The concentration of the compound was 20 µM and the solvent used was MeOH.

Figure S121. pH test of AR82d under visible (up) and UV (down) light.

	**R HCl 3.4 4.8 6.4 7.4 7.9 10.5 KOH**
**Visible light**	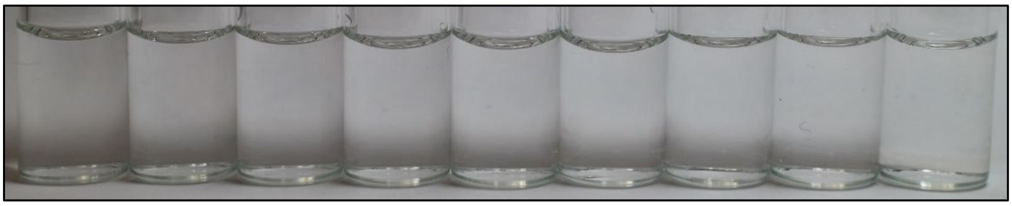

**UV light (366 nm)**	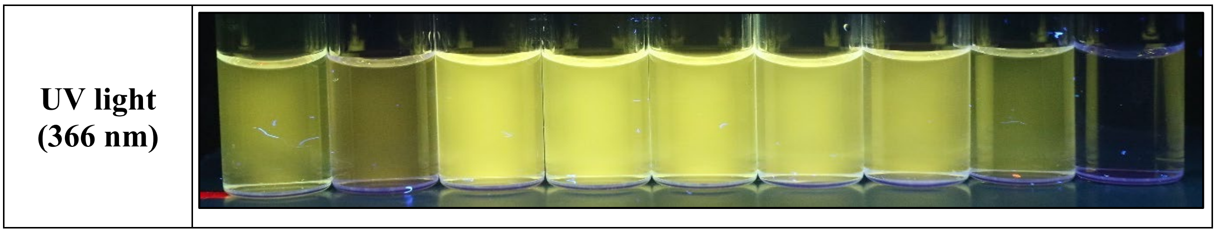

There was a quenching in the emission at very basic pH. At intermediate pH, the colour of the fluorescence was changed from green to yellow (3.4 – 7.9).

Cations and anions test:

The concentration of the compound was 20 μM and the solvent used was MeOH.

Figure S122. Cations test of AR82d under visible (up) and UV (down) light.

	**R Ag^{+} Ni^{2+} Sn^{2+} Cd^{2+} Zn^{2+} Pb^{2+} Cu^{2+} Fe^{3+} Sc^{3+} Al^{3+} Hg^{2+} Au^{3+} Co^{2+} Pd^{2+} Ir^{3+} Cu^{+} Ru^{3+} Pt^{2+}**
**Visible light**	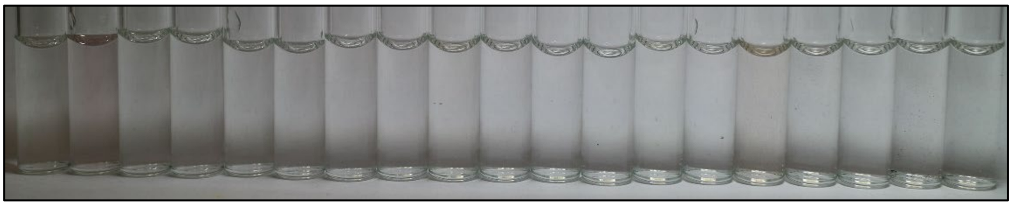
**UV light (366 nm)**	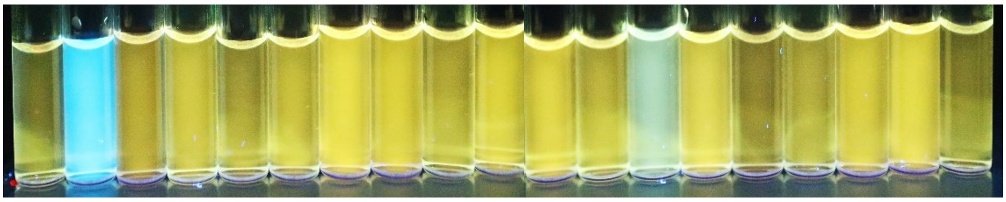

The most significant changes under UV light were the turn of the fluorescence to blue against Ag^{+} and Au^{3+}.

The concentration of the compound was 20 μM and the solvent used was MeOH.

Figure S123. Anions test of AR82d under visible (up) and UV (down) light.

	**R F^{-} Cl^{-} Br^{-} I^{-} BzO^{-} NO_3^{-} $H_2PO_4^{-}$ HSO_4^{-} AcO^{-} CN^{-} SCN^{-}**
**Visible light**	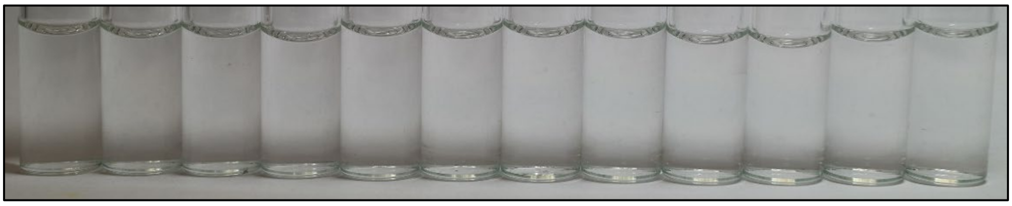
**UV light (366 nm)**	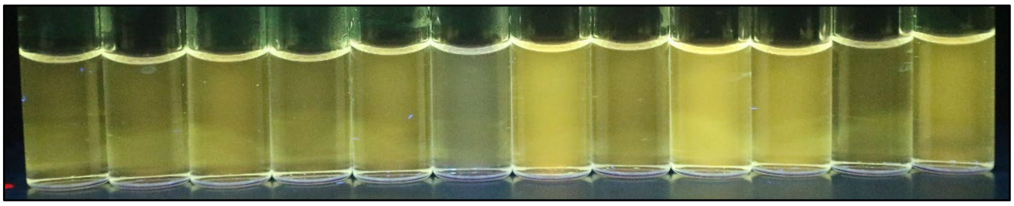

No significant changes were observed.

Oxidants and reductants test:

The solvent used was MeOH and concentration of the compound was 20 µM.

Figure S124. Oxidants and reductants test of AR82d under visible (up) and UV (down) light.

	R HCl HNO_3 m-CPBA Oxn Hz H_2O_2 TNB TNT TATP HMTD
**Visible light**	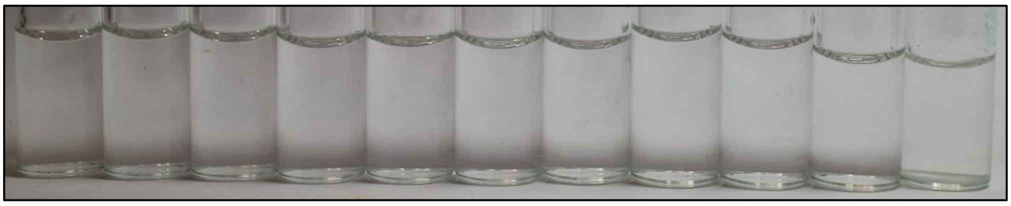
**UV light (366 nm)**	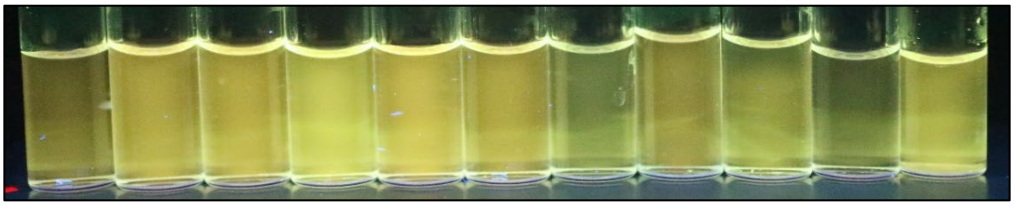

There was a slight change against the TATP, it was changed from yellow to green.

Preliminary solvents test:

In order to determine the fluorescence variation of the compound under study in the presence of TATP, some tests were carried out for the probe in 9 different solvents. The AR82d concentration was 20 µM and the TATP was added in excess (7 mg in each vial). All the tests were performed at room temperature and the photographs were taken immediately after the addition of TATP and again after 24 hours.

Figure S125. Pairs of AR82d (left) and AR82d with excess of TATP (right) in different solvents at time 0 h (up) and 24 h (down).

	DMF MeCN EtOAc THF $CHCl_3$ DCM Tol $CHCl_3$:MeOH DCM:MeOH
**t = 0 h**	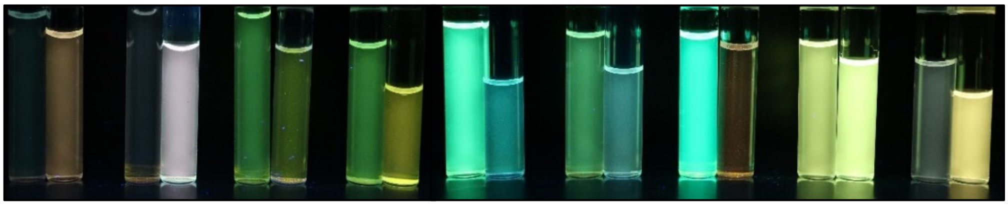
**t = 24 h**	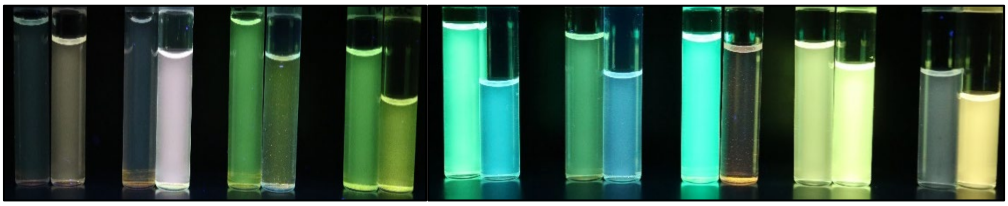

The biggest fluorescence changes were seen in DMF, MeCN and DCM:MeOH (9:1) with a emission intensity increase and in toluene a fluorescence color change was observed. The studies were also performed in $CHCl_3$, THF, EtOAc and DCM with a color change and in $CHCl_3$:MeOH (9:1) with a increase in the emission intensity, but less than the previous ones.

Work concentration test:

In order to choose an optimum concentration for the next quantitative studio, the absorbance and fluorescence of the probe was checked to be linear while concentration changes was small. The solvents used for this test were toluene, EtOAc and DCM. The excitation wavelength for all measurements was 360 nm.

Figure S126. Absorbance spectrum (up left), absorbance profile at 380 nm (up right), fluorescence spectrum (down left) and fluorescence profile at 470 nm (down right) of increasing concentrations of AR82d solution in toluene.

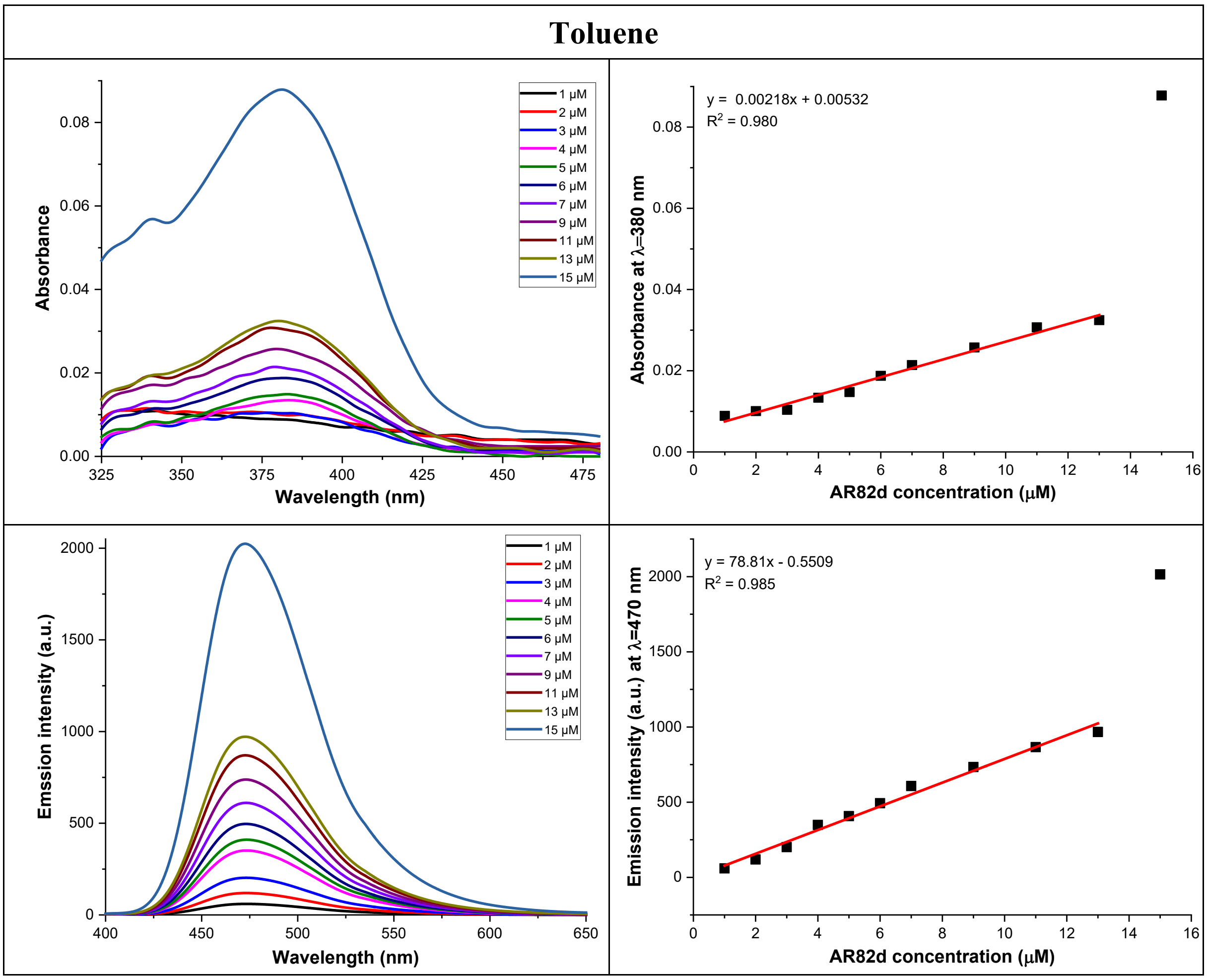

The ideal working concentration should stay at 0.1 of absorbance or less, to avoid inner filter effects, possible dynamic quenching or stacking processes. It was implied a concentration below 13 µM. Taking the results into account, the chosen concentration was 1 µM, value around which the Lambert – Beer Law (or pseudo – Lambert – Beer linear behavior for fluorescence) was fulfilled.

With m-CPBA the obtained result was the same: the concentration of the probe had to be less than 7 µM, so a concentration of 1 µM was adequate.

Figure S127. Absorbance spectrum (up left), absorbance profile at 360 nm (up right), fluorescence spectrum (down left) and fluorescence profile at 450 nm (down right) of increasing concentrations of AR82d solution with a m-CPBA excess (7000 µM) in toluene.

Toluene

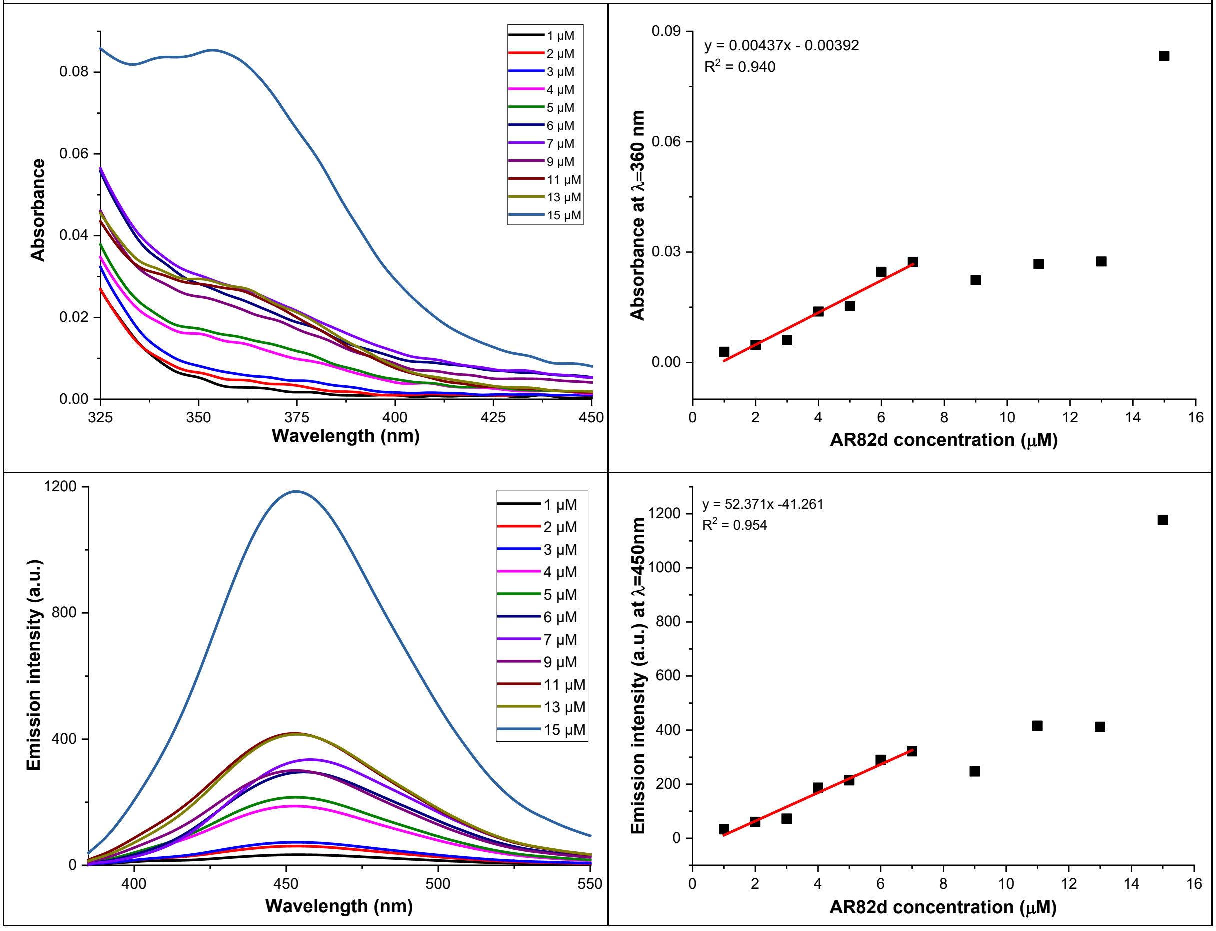

Figure S128. Absorbance spectrum (up left), absorbance profile at 375 nm (up right), fluorescence spectrum (down left) and fluorescence profile at 475 nm (down right) of increasing concentrations of AR82d solution in EtOAc.

EtOAc

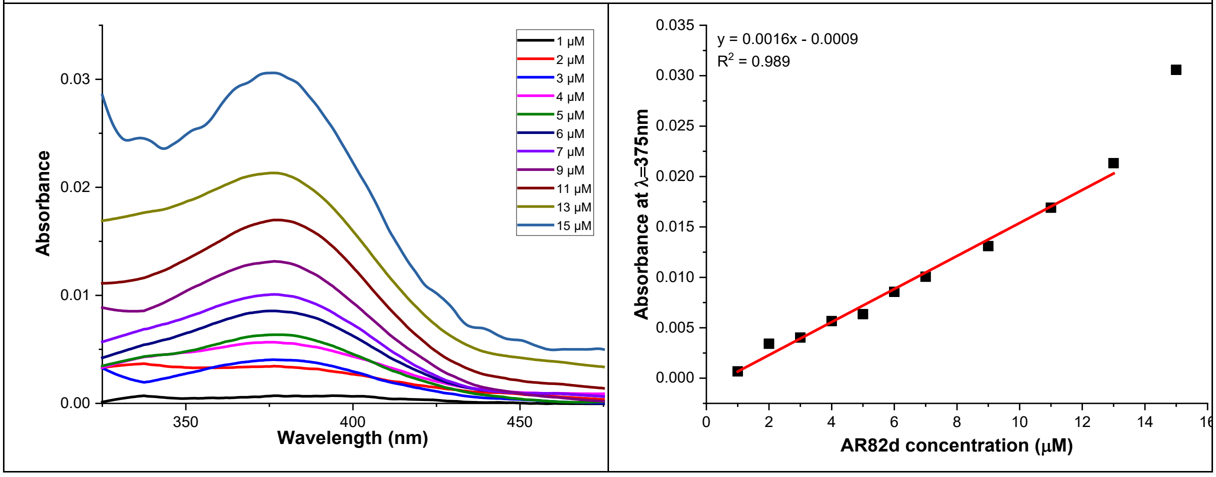

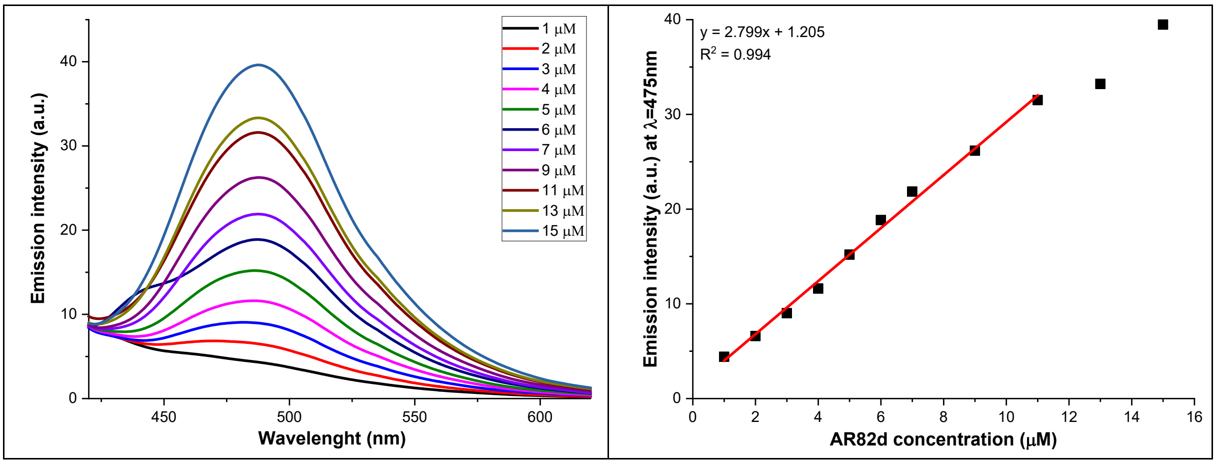

The obtained result was similar to the previous one. To work at an absorbance of less than 0.1, the probe concentration had to be below 12 μM, so a good working concentration was 2.5 μM, while with m-CPBA, the concentration should be below 6 μM so, 1 μM was also suitable. It was a concentration around which the Lambert – Beer Law (or pseudo – Lambert – Beer) was fulfilled.

Figure S129. Absorbance spectrum (up left), absorbance profile at 370 nm (up right), fluorescence spectrum (down left) and fluorescence profile at 485 nm (down right) of increasing concentrations of AR82d solution with a m-CPBA excess (7000 μM) in EtOAc.

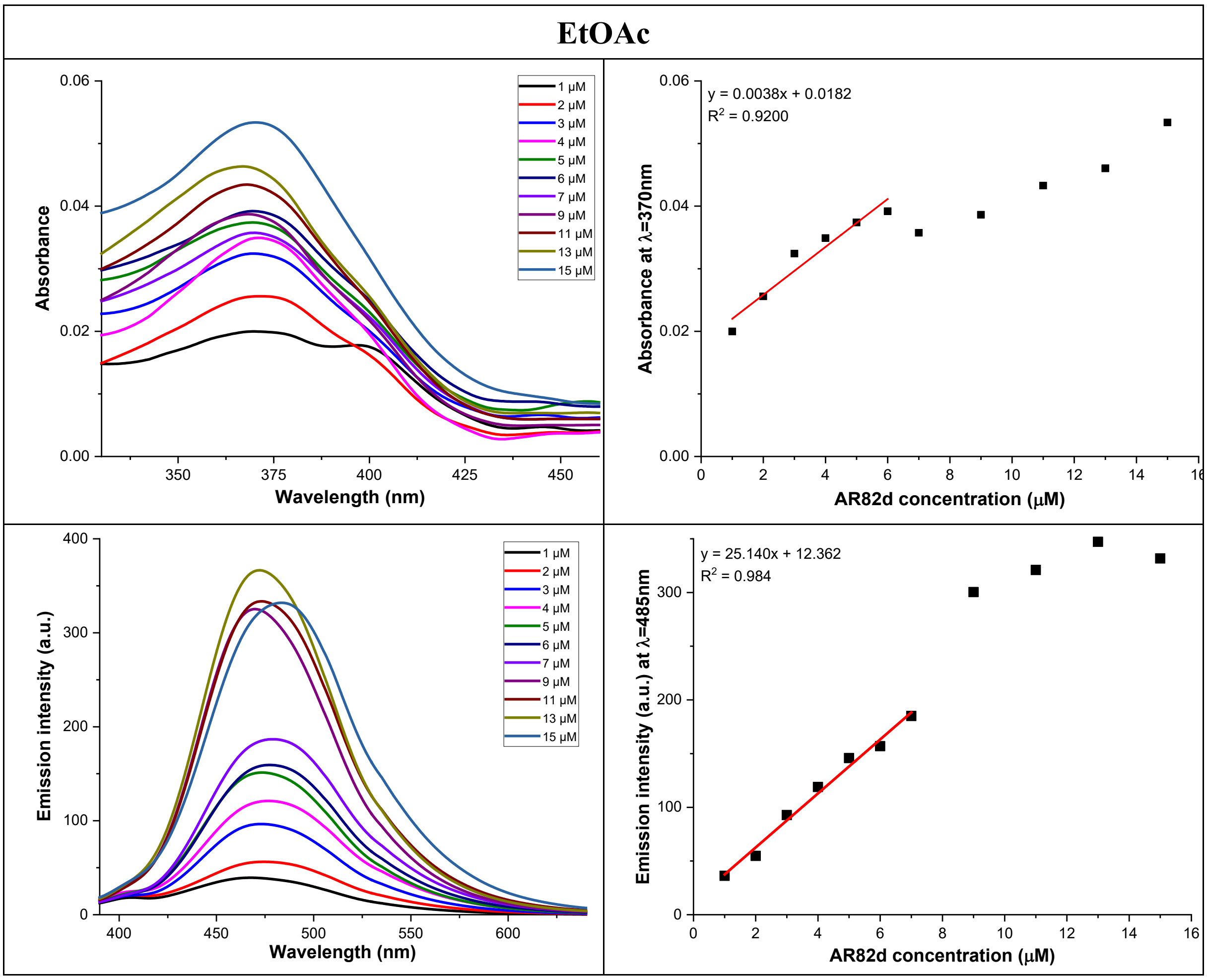

Figure S130. Absorbance spectrum (up left), absorbance profile at 380 nm (up right), fluorescence spectrum (down left) and fluorescence profile at 475 nm (down right) of increasing concentrations of AR82d solution in DCM.

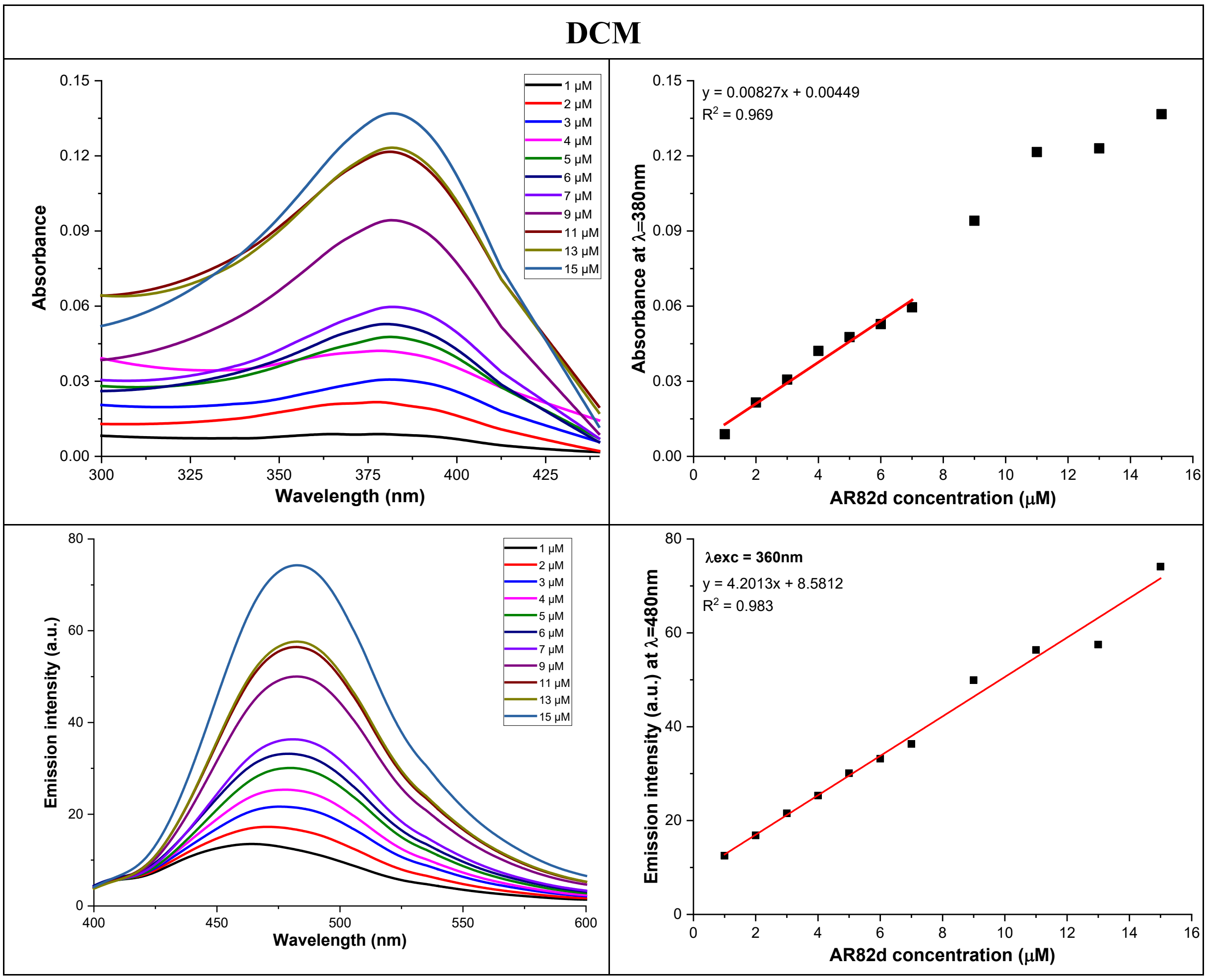

The result obtained with this solvent was similar to that obtained with the two previous ones.

Figure S131. Absorbance spectrum (up left), absorbance profile at 360 nm (up right), fluorescence spectrum (down left) and fluorescence profile at 465 nm (down right) of increasing concentrations of AR82d solution with a m-CPBA excess (7000 µM) in DCM.

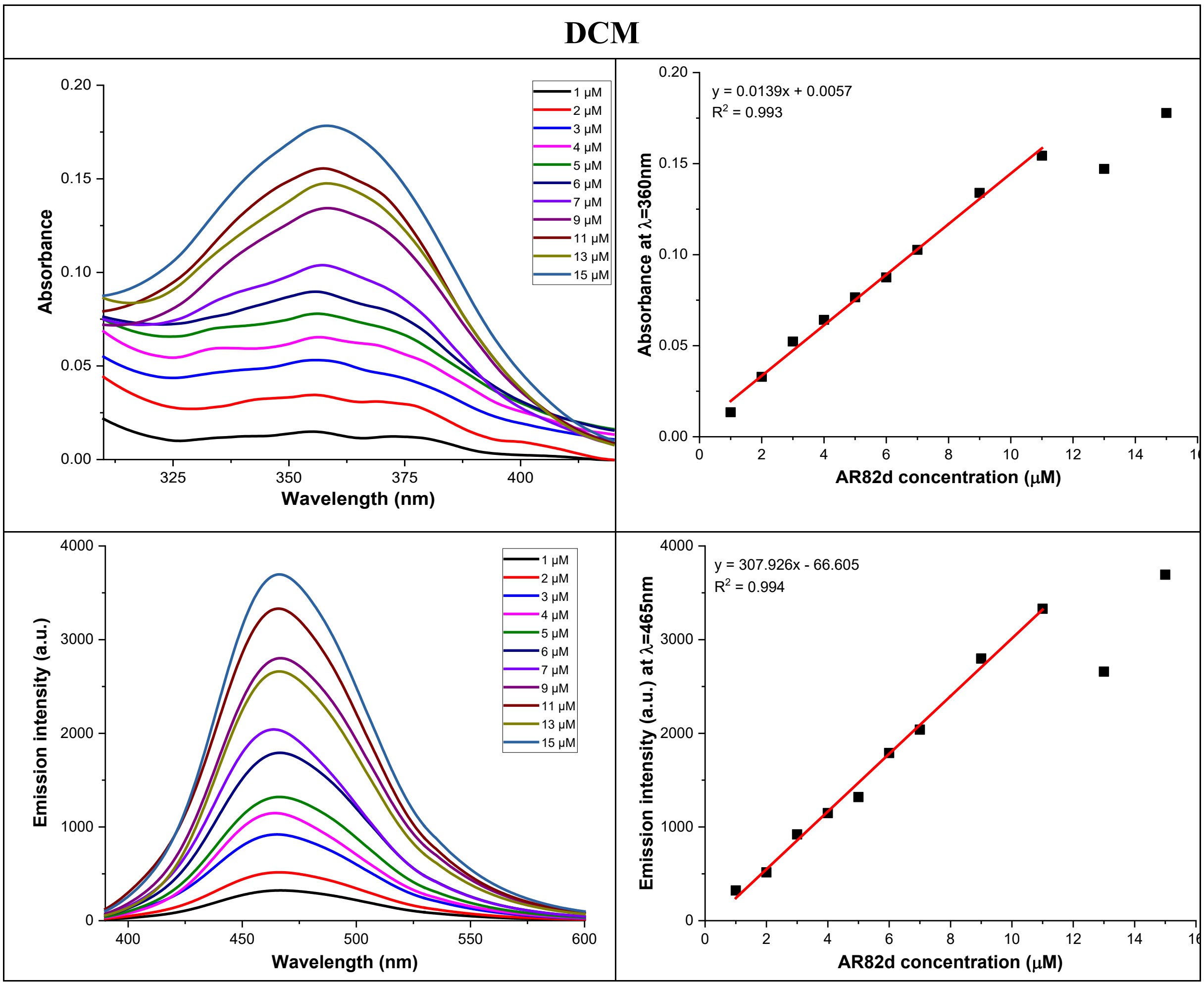

Solvents test with TATP:

For all solvents, the working concentration of AR82d was 2.5 µM, the excitation wavelenght was 360 nm and the temperature for all test was 25ºC.

In the kinetic studies, TATP was in excess (20 mM) and the fluorescence emission measurements were made during 30 minutes in the case of DMF, 150 minutes in MeCN, 120 minutes in DCM, 45 minutes in the mixture DCM:MeOH (9:1) and 60 minutes for all other solvents, at 5- or 10-minutes time intervals. In the titration, the TATP concentration was between 0 and 35000 µM and it was added directly as solid. The measurements were carried out immediately after the addition of the probe.

Figure S132. Study of AR82d with TATP in DMF. Kinetic study (up left) and profile as function as time at 390 nm and 475 nm simultaneously (up right) in presence of TATP excess. Titration (down left) and fluorescence profile at 470 nm and 595 nm simultaneously (down right) under increasing concentrations of TATP.

DMF

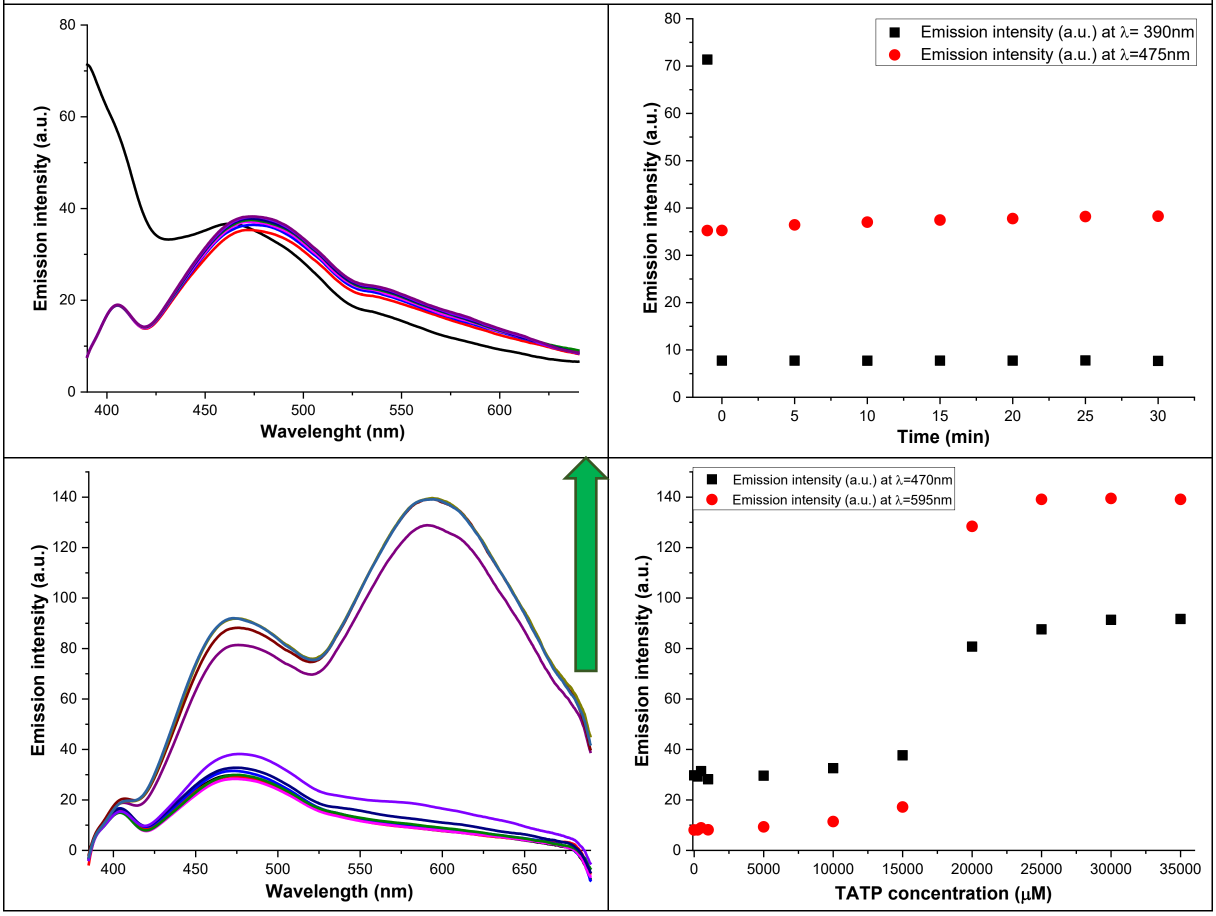

There was a change in the emission intensity band immediately after adding TATP. According to the fluorescence spectra, the LOD of TATP was somewhere between 15000 and 20000 μM with an increase of the emission intensity.

Figure S133. Kinetic study at different times under visible (up) and UV (down) light in DMF. In each photo, left vial contained probe and right vial contained probe and excess TATP.

	**t=0**	**t=30 min**	**t=24 h**
**Visible light**	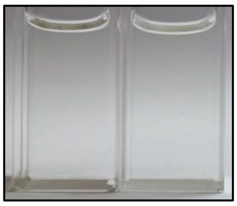	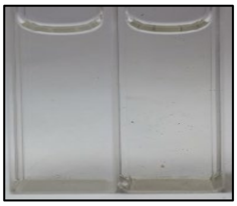	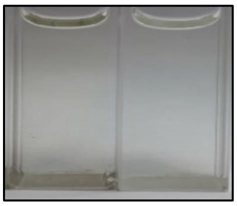
**UV light (366 nm)**	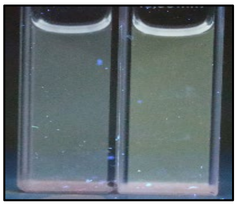	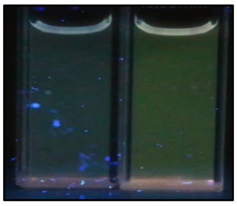	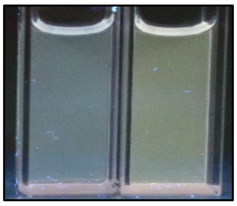

Figure S134. Titration of AR82d with excess TATP under visible (up) and UV (down) light in DMF.

µM	0 250 500 1000 5000 10000 15000 20000 25000 30000 35000
**Visible light**	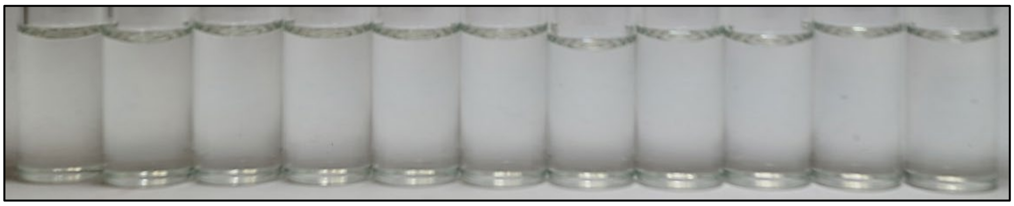
**UV light (366 nm)**	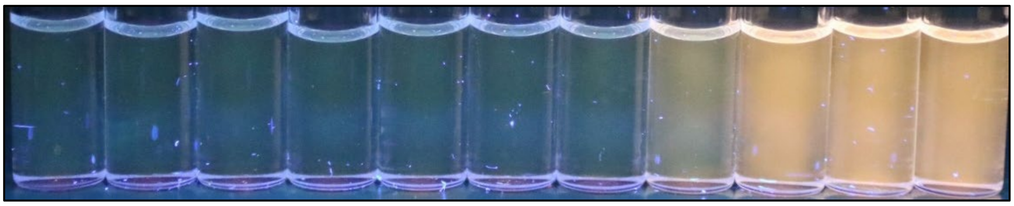

Figure S135. Study of AR82d with TATP in MeCN. Kinetic study (up left) and profile as function as time at 450 nm and 570 nm simultaneously (up right) in presence of TATP excess. Titration (down left) and fluorescence profile at 450 nm and 580 nm simultaneously (down right) under increasing concentrations of TATP.

MeCN

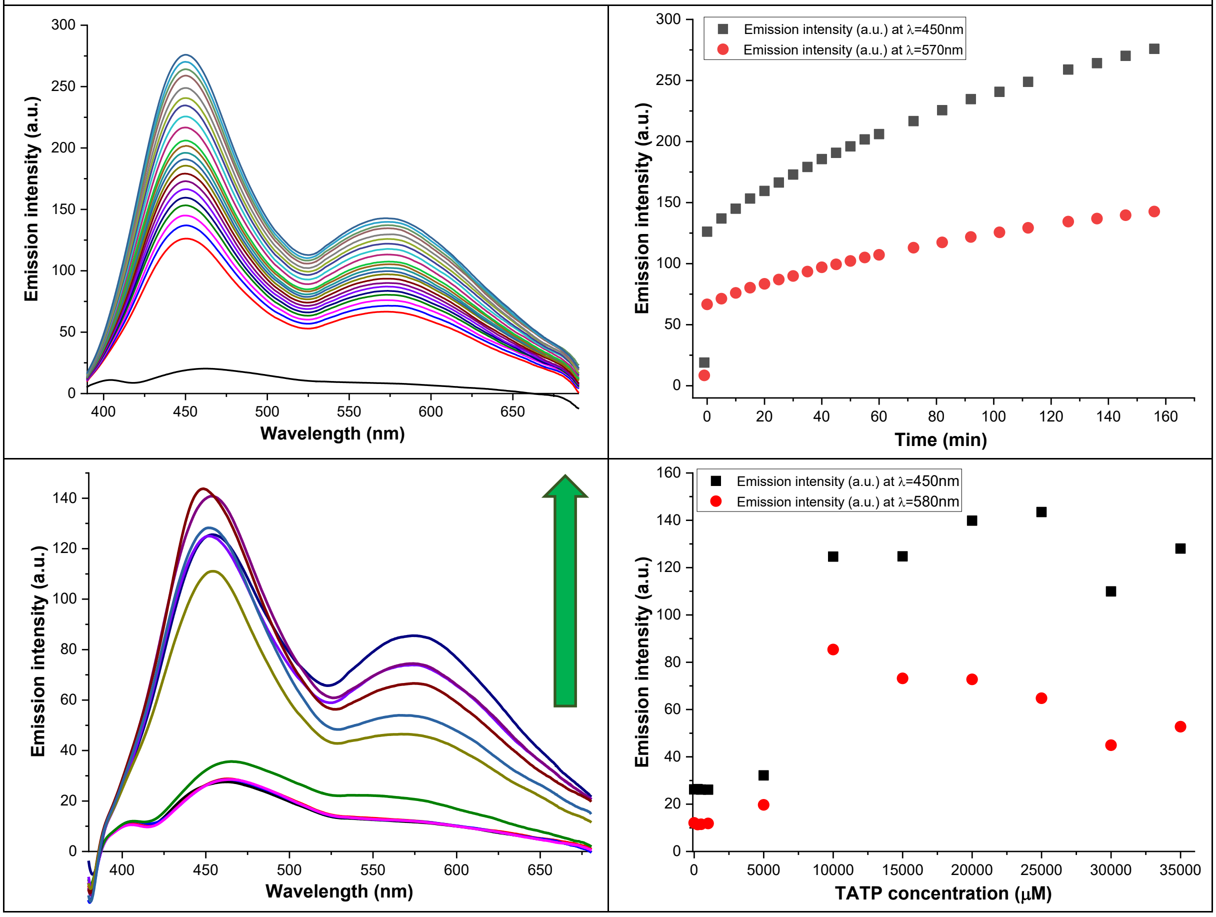

There was an increase in the emission intensity band immediately after adding TATP and it was stabilized after 140 – 160 minutes. According to the fluorescence spectra, the LOD was somewhere between 5000 and 10000 µM with a increase of the emission intensity.

Figure S136. Kinetic study at different times under visible (up) and UV (down) light in MeCN. In each photo, left vial was contained the probe and right vial was contained the probe and an excess of TATP.

	**t=0**	**t=60 min**	**t=24 h**
**Visible light**	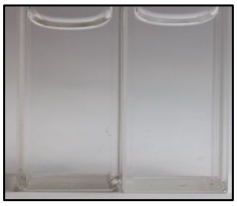	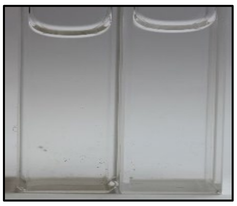	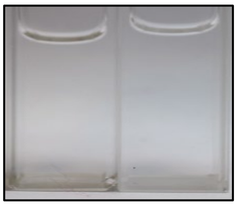
**UV light (366 nm)**	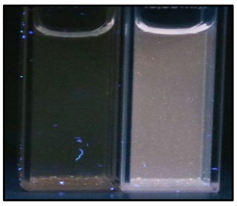	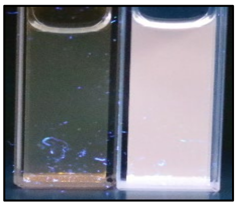	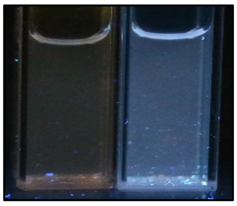

Figure S137. Titration of AR82d with an excess of TATP under visible (up) and UV (down) light in MeCN.

μM	**0 250 500 1000 5000 10000 15000 20000 25000 30000 35000**
**Visible light**	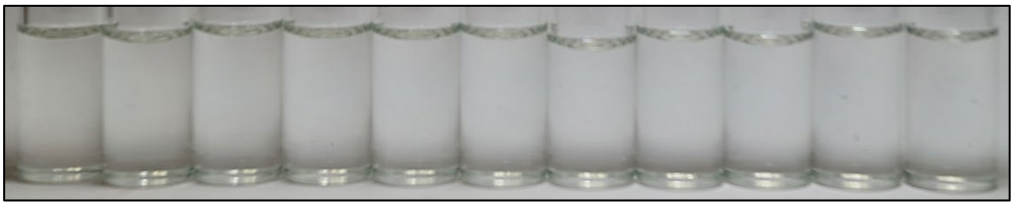
**UV light (366 nm)**	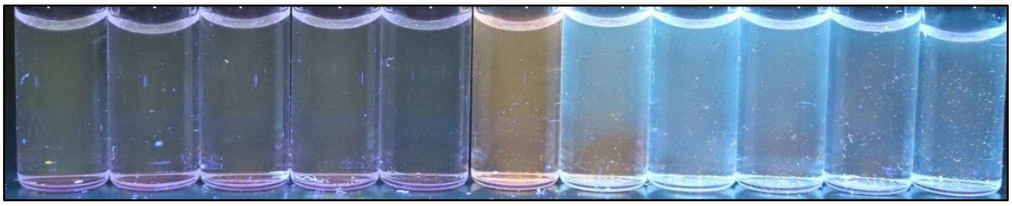

Figure S138. Study of AR82d with TATP in toluene. Kinetic study (up left) and profile as function as time at 450 nm and 570 nm simultaneously (up right) in presence of TATP excess. Titration (down left) and fluorescence profile at 505 nm and 570 nm simultaneusly (down right) under increasing concentrations of TATP.

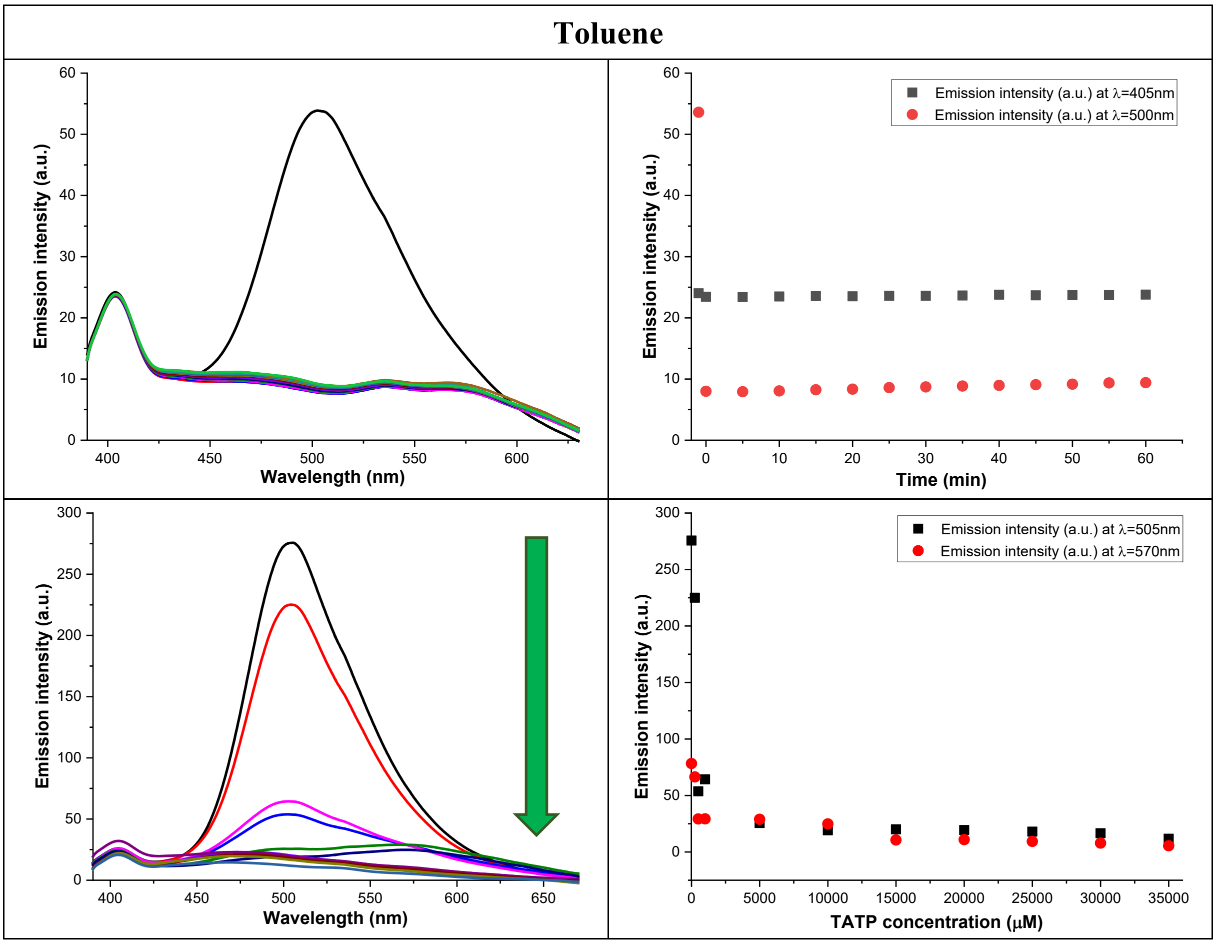

There was a drop in the emission intensity band immediately after adding TATP. According to the fluorescence spectra, the LOD was somewhere between 0 and 250 μM.

Figure S139. Kinetic study at different times under visible (up) and UV (down) light in toluene. In each photo, left vial was contained the probe and right vial was contained the probe and an excess of TATP.

	**t=0**	**t=60 min**	**t=24 h**
**Visible light**	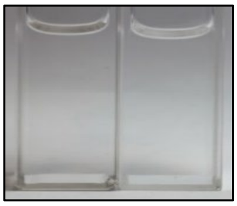	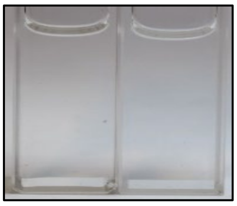	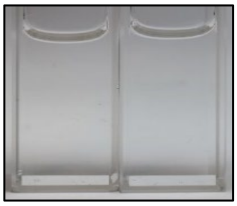
**UV light (366 nm)**	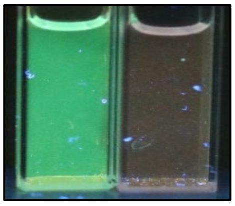	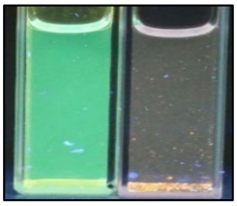	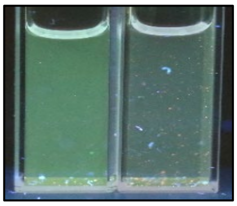

Figure S140. Titration of AR82d with an excess of TATP under visible (up) and UV (down) light in toluene.

µM	0 250 500 1000 5000 10000 15000 20000 25000 30000 35000
**Visible light**	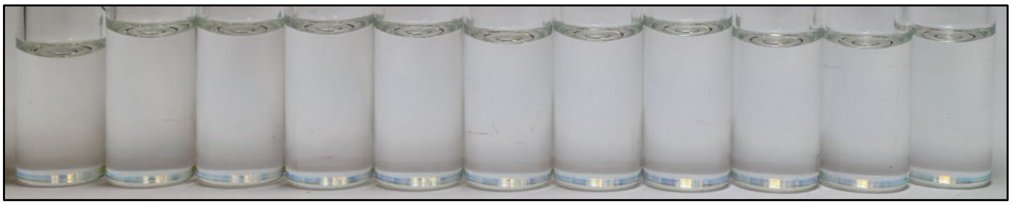
**UV light (366 nm)**	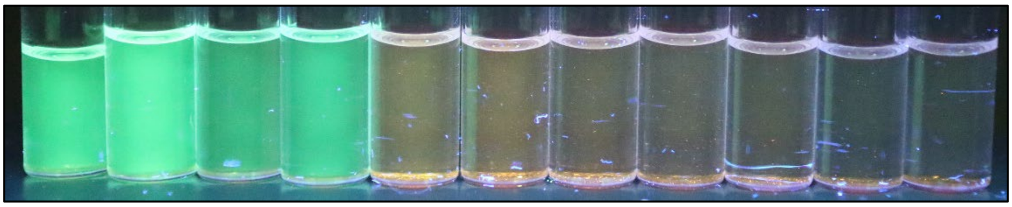

Figure S141. Study of AR82d with TATP in DCM:MeOH (9:1). Kinetic study (up left) and profile as function as time at 450 nm and 555 nm simultaneously (up right) in presence of TATP excess. Titration (down left) and fluorescence profile at 450 nm and 555 nm simultaneously (down right) under increasing concentrations of TATP.

DCM:MeOH (9:1)

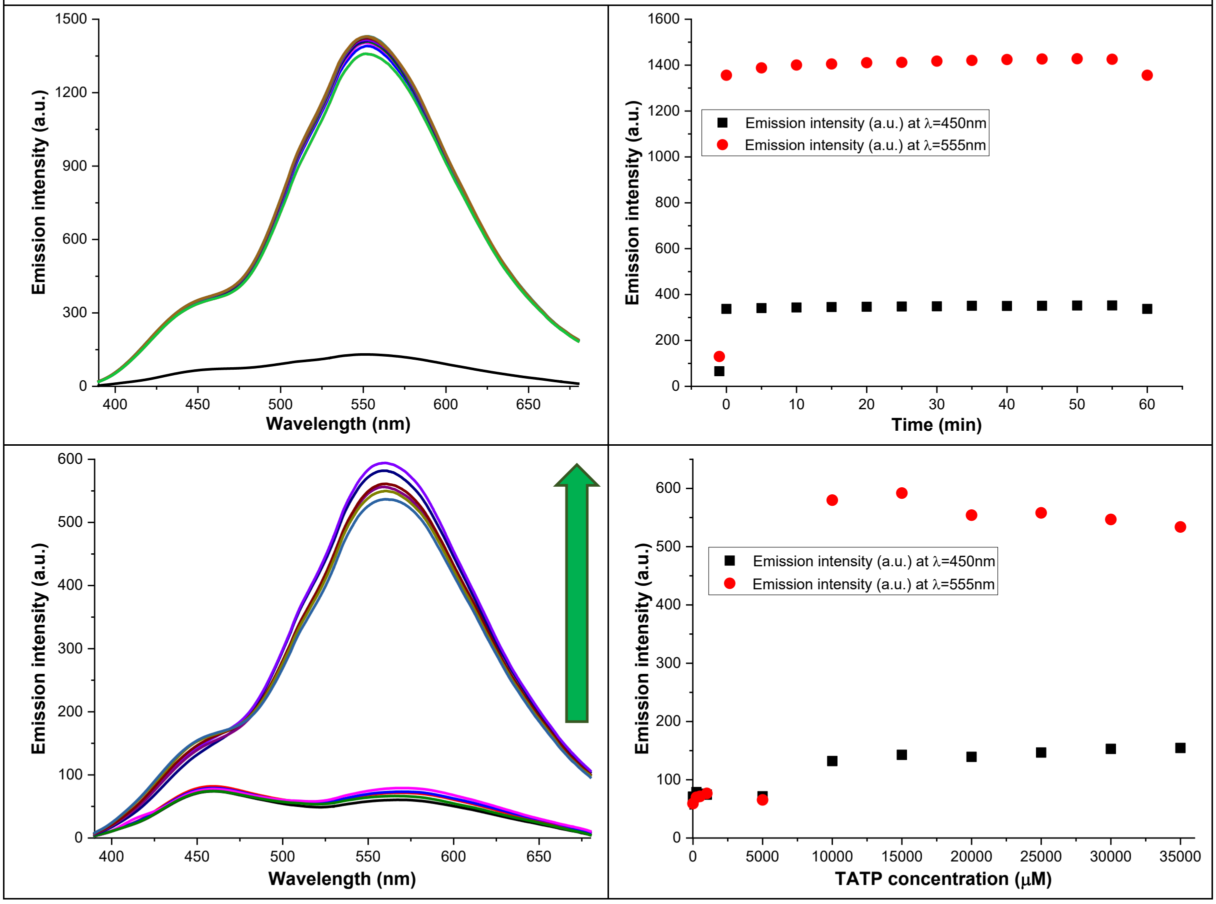

There was an increase in the emission intensity band immediately after adding TATP. According to the fluorescence spectra, the LOD was somewhere between 5000 and 10000 µM.

Figure S142. Kinetic study at different times under visible (up) and UV (down) light in DCM:MeOH (9:1). In each photo, left vial was contained the probe and right vial was contained the probe and an excess of TATP.

	**t=0**	**t=60 min**	**t=24 h**
**Visible light**	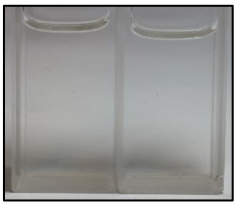	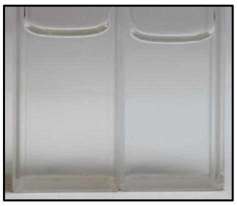	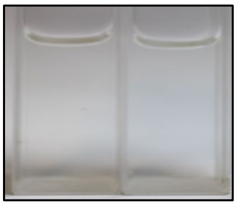
**UV light (366 nm)**	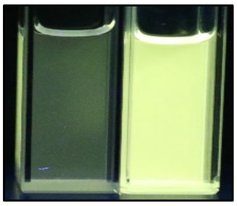	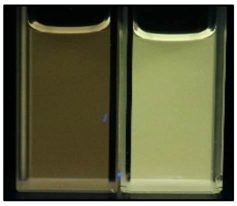	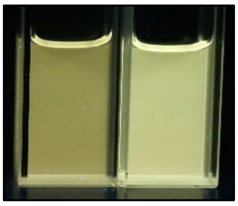

Figure S143. Titration of AR82d with an excess of TATP under visible (up) and UV (down) light in DCM:MeOH (9:1).

µM	**0 250 500 1000 5000 10000 15000 20000 25000 30000 35000**
**Visible light**	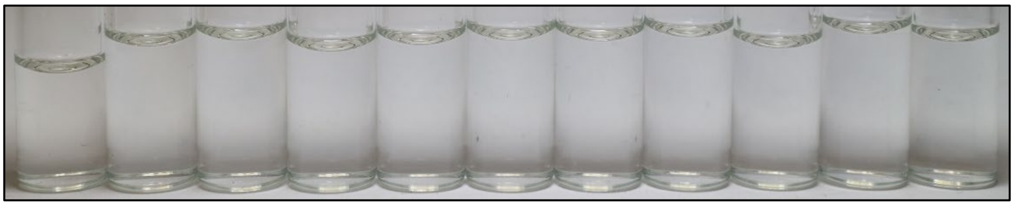
**UV light (366 nm)**	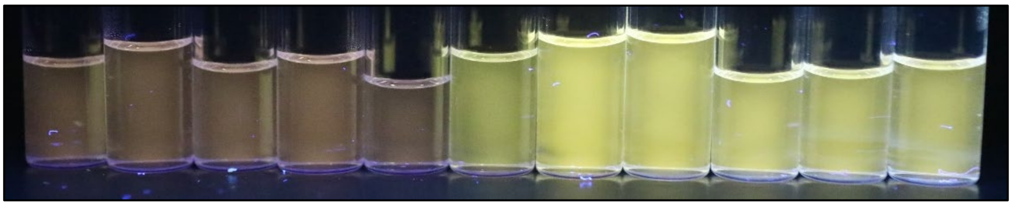

Figure S144. Study of AR82d with TATP in $CHCl_3$. Kinetic study (up left) and profile as function as time at 500 nm and 510 nm simultaneously (up right) in presence of TATP excess. Titration (down left) and fluorescence profile at 505 nm and 530 nm simultaneously (down right) under increasing concentrations of TATP.

$CHCl_3$

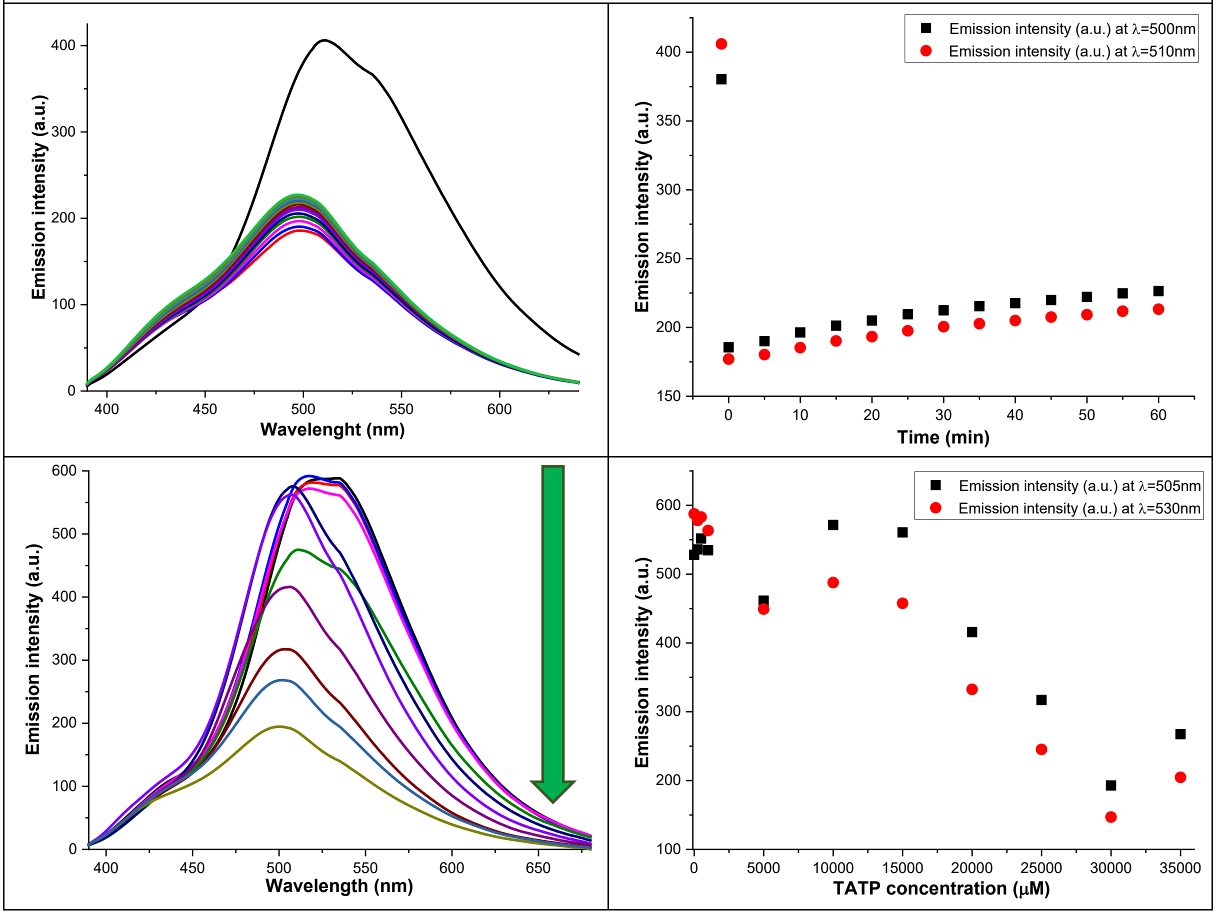

There was a decrease in the emission intensity band immediately after adding TATP. According to the fluorescence spectra, the LOD was somewhere between 1000 and 5000 μM, a high limit.

Figure S145. Kinetic study at different times under visible (up) and UV (down) light in $CHCl_3$. In each photo, left vial was contained the probe and right vial was contained the probe and an excess of TATP.

	t=0	t=60 min	t=24 h
**Visible light**	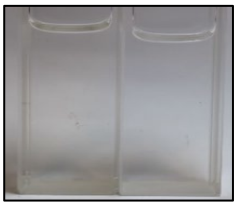	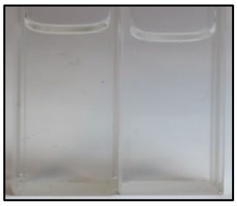	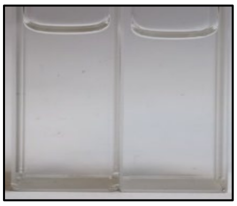
**UV light (366 nm)**	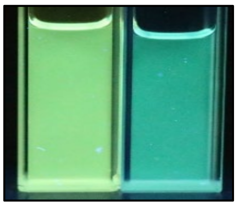	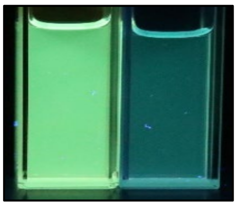	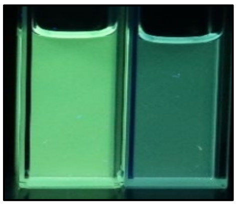

Figure S146. Titration of AR82d with an excess of TATP under visible (up) and UV (down) light in $CHCl_3$.

μM	0 250 500 1000 5000 10000 15000 20000 25000 30000 35000
**Visible light**	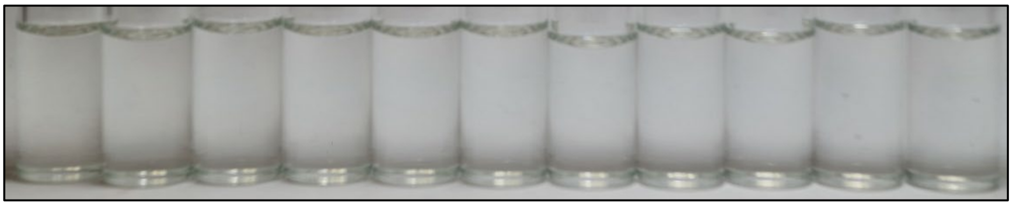
**UV light (366 nm)**	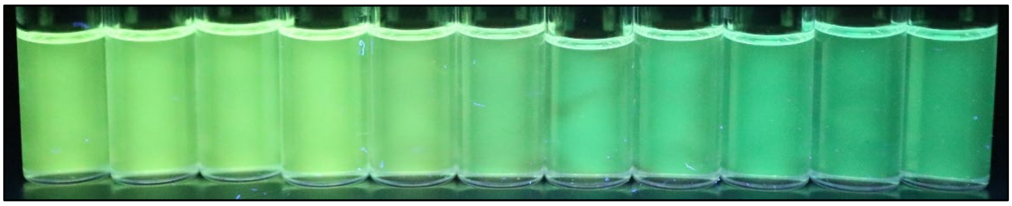

Figure S147. Study of AR82d with TATP in THF. Kinetic study (up left) and profile as function as time at 400 nm and 550 nm simultaneously (up right) in presence of TATP excess. Titration (down left) and fluorescence profile at 400 nm and 550 nm simultaneously (down right) under increasing concentrations of TATP.

THF

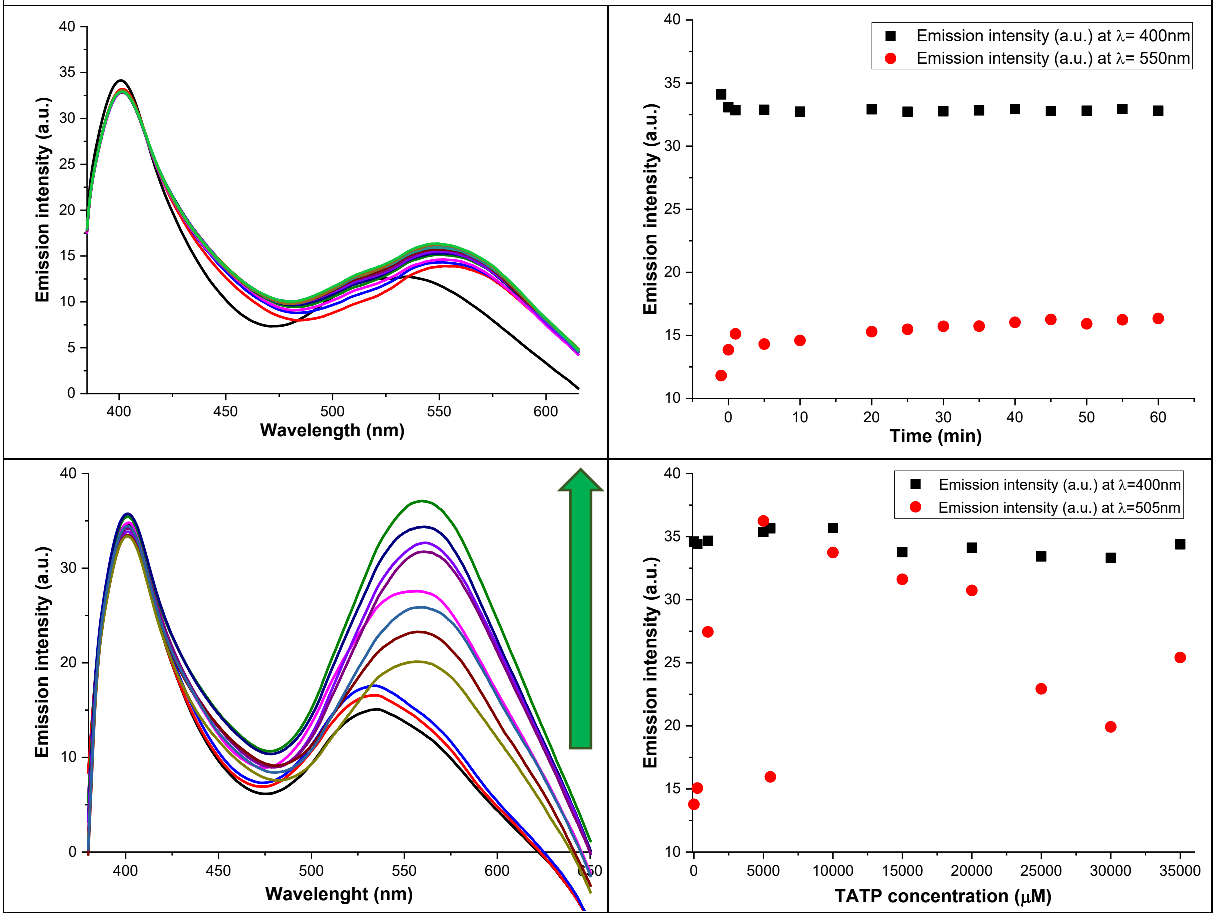

Related to the 550 nm band, there was a low increase in the emission intensity and a shifted to higher wavelengths immediately after adding TATP. According to the fluorescence spectra, the LOD was somewhere between 1000 and 5000 μM, a bit high limit.

Figure S148. Kinetic study at different times under visible (up) and UV (down) light in THF. In each photo, left vial was contained the probe and right vial was contained the probe and an excess of TATP.

	**t=0**	**t=60 min**	**t=24 h**
**Visible light**	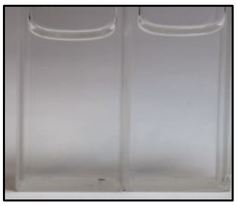	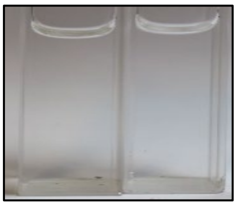	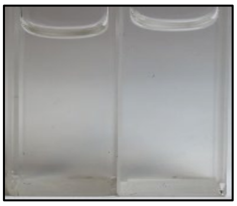
**UV light (366 nm)**	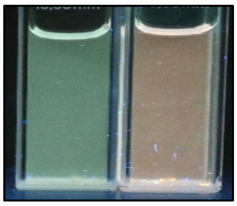	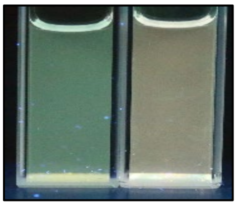	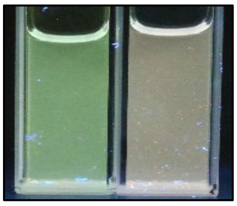

Figure S149. Titration of AR82d with an excess of TATP under visible (up) and UV (down) light in THF.

	**0 250 500 1000 5000 10000 15000 20000 25000 30000 35000**
**Visible light**	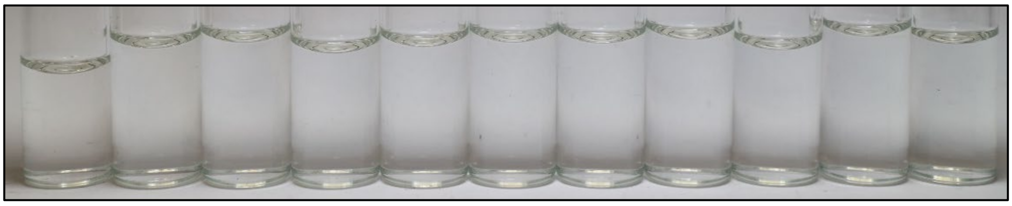
**UV light (366 nm)**	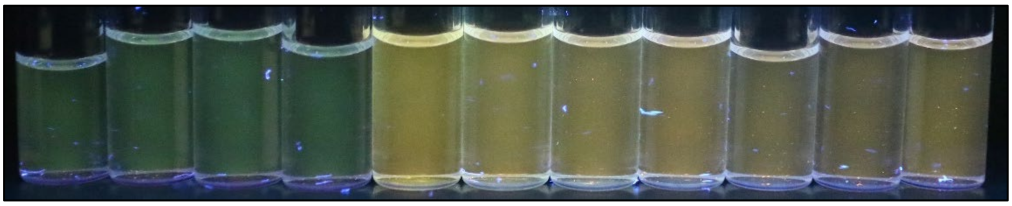

Figure S150. Study of AR82d with TATP in EtOAc. Kinetic study (up left) and profile as function as time at 380 nm, 400 nm and 520 nm simultaneously (up right) in presence of TATP excess. Titration (down left) and fluorescence profile at 500 nm and 560 nm simultaneously (down right) under increasing concentrations of TATP.

EtOAc

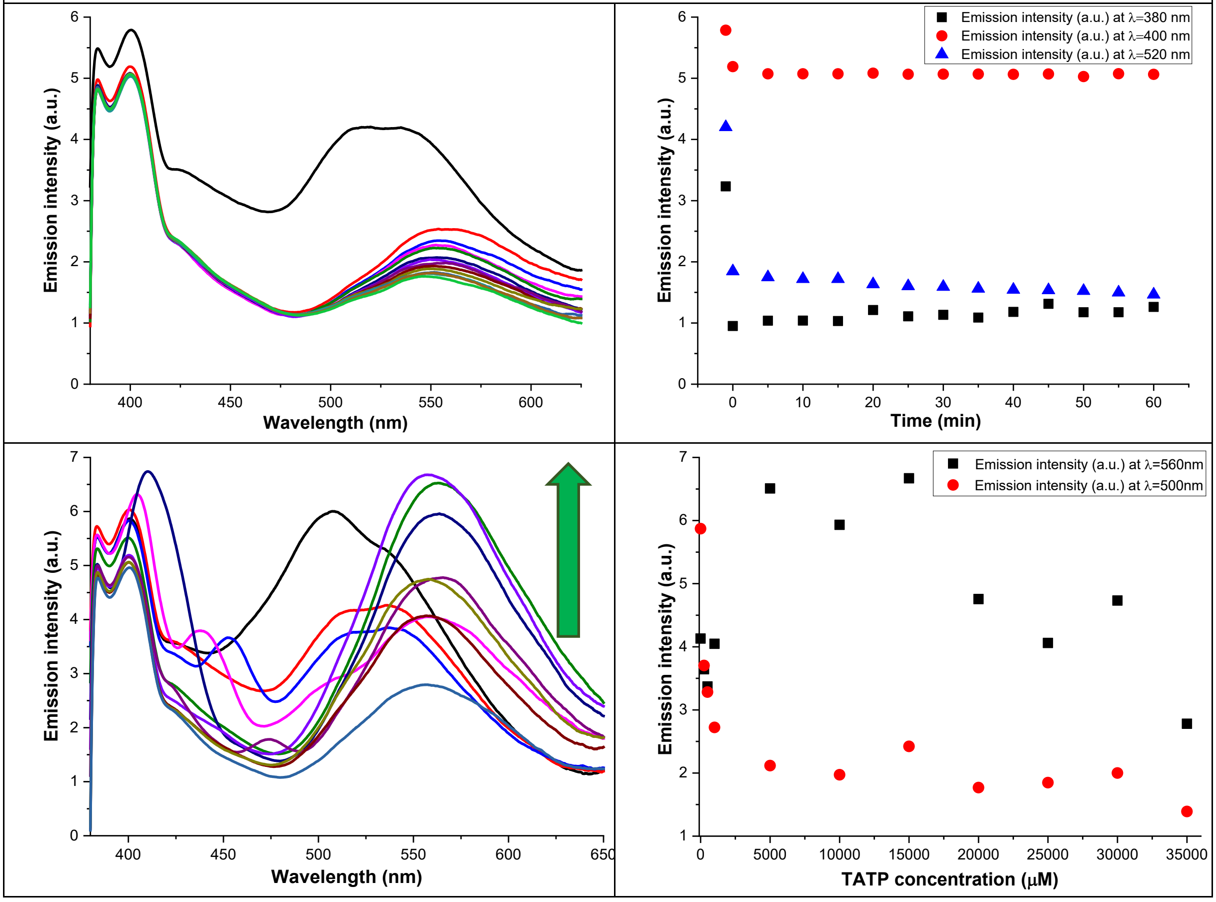

There was a drop in the emission intensity and a shifted to higher wavelengths immediately after adding TATP. According to the fluorescence spectra, the LOD was somewhere between 1000 and 5000 μM, with a displacement of the maximum emission.

Figure S151. Kinetic study at different times under visible (up) and UV (down) light in EtOAc. In each photo, left vial was contained the probe and right vial was contained the probe and an excess of TATP.

	**t=0**	**t=60 min**	**t=24 h**
**Visible light**	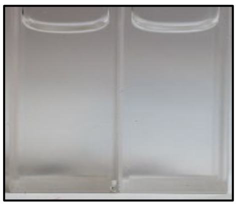	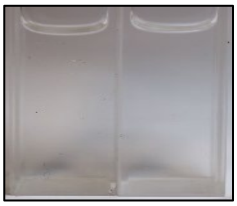	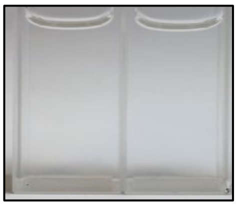
**UV light (366 nm)**	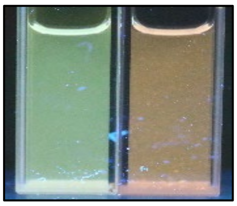	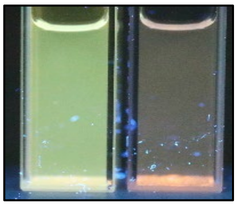	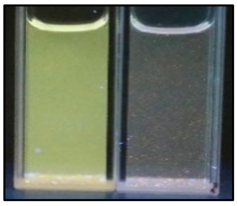

Figure S152. Titration of AR82d with an excess of TATP under visible (up) and UV (down) light in EtOAc.

μM	0 250 500 1000 5000 10000 15000 20000 25000 30000 35000
**Visible light**	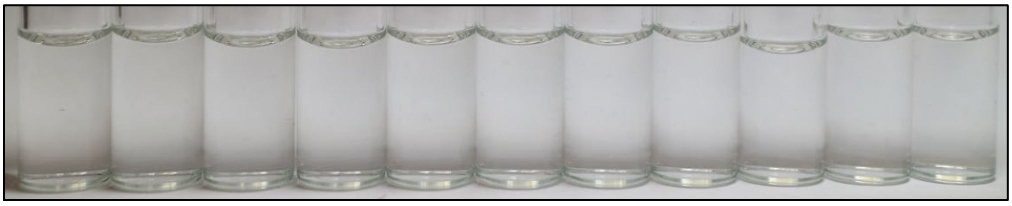
**UV light (366 nm)**	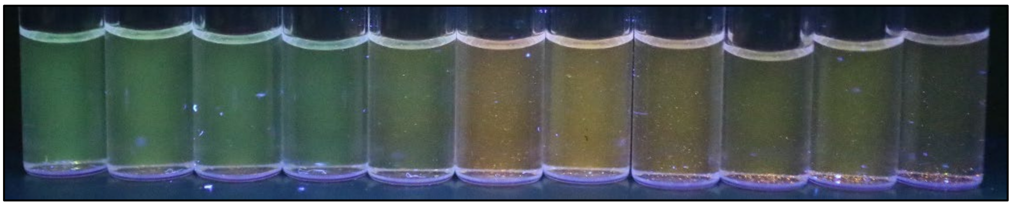

Figure S153. Study of AR82d with TATP in DCM. Kinetic study (up left) and profile as function as time at 430 nm and 530 nm simultaneously (up right) in presence of TATP excess. Titration (down left) and fluorescence profile at 430 nm and 525 nm simultaneously (down right) under increasing concentrations of TATP.

**DCM**	
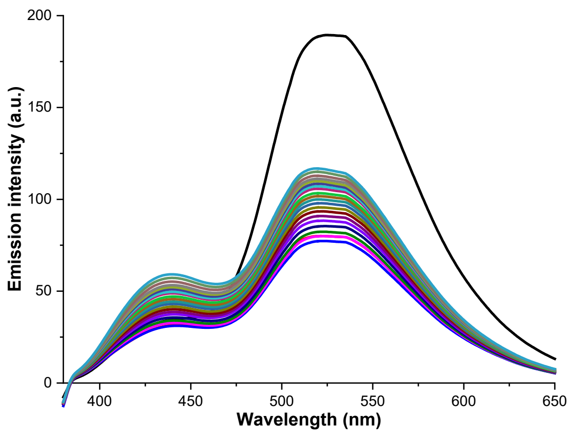	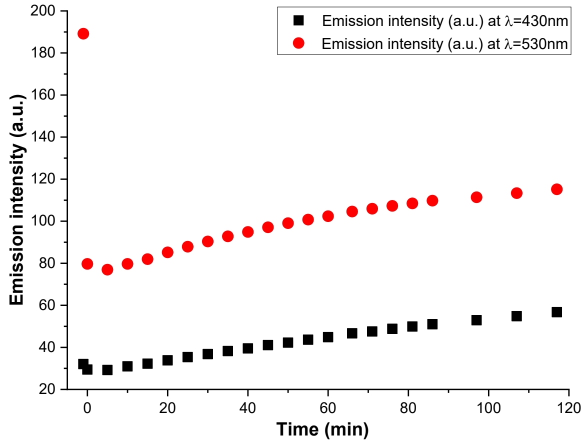
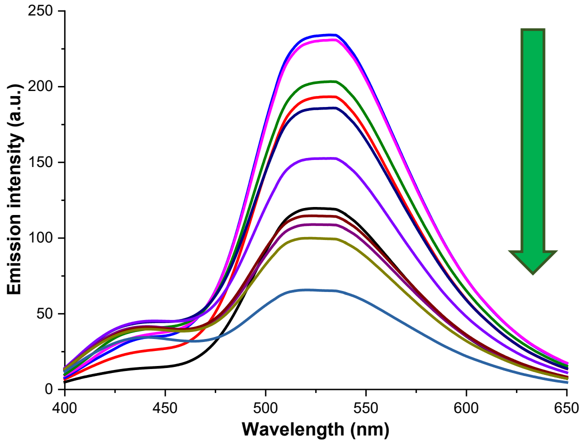	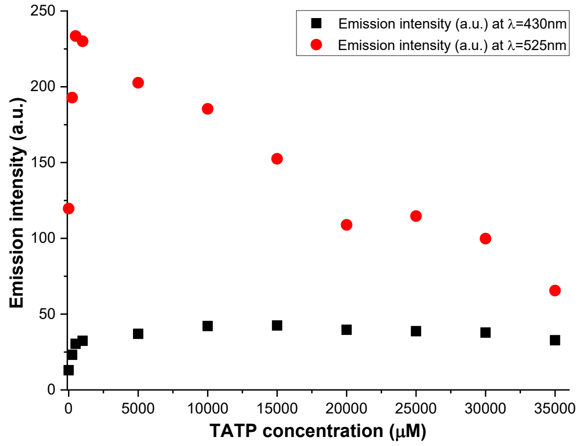

There was a change in the emission intensity immediately after adding TATP. The LOD was somewhere between 0 and 250 μM, with an increase of the intensity.

Figure S154. Kinetic study at different times under visible (up) and UV (down) light in DCM. In each photo, left vial was contained the probe and right vial was contained the probe and an excess of TATP.

	**t=0**	**t=140 min**	**t=24 h**
**Visible light**	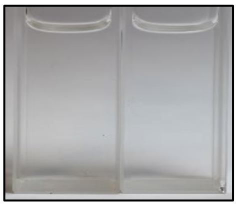	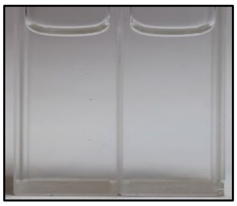	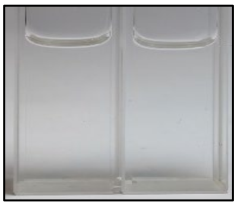
**UV light (366 nm)**	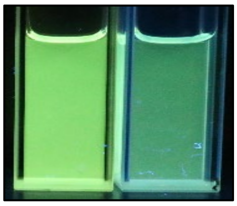	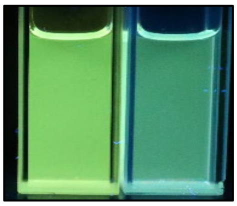	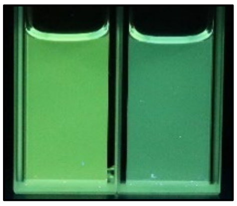

Figure S155. Titration of AR82d with an excess of TATP under visible (up) and UV (down) light in DCM.

	**0 250 500 1000 5000 10000 15000 20000 25000 30000 35000**
**Visible light**	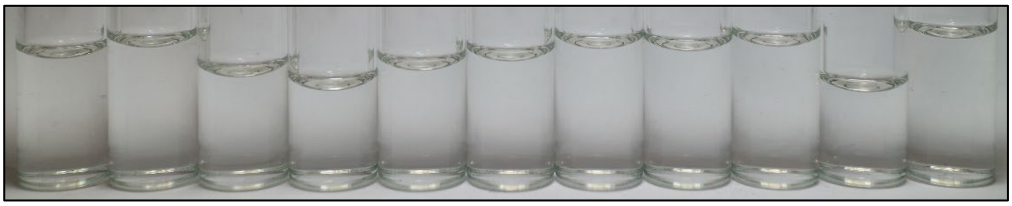
**UV light (366 nm)**	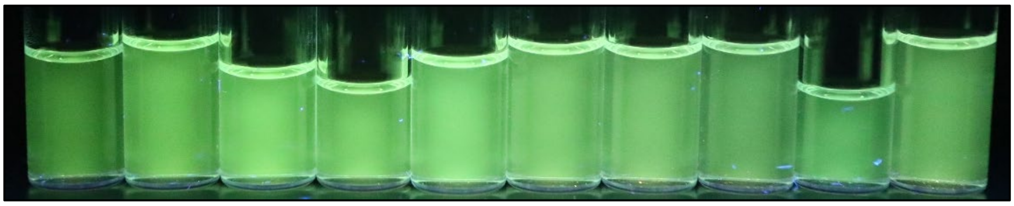

Figure S156. Kinetic study (up left) and profile as function as time at 445 nm and 545 nm simultaneously (up right) in presence of TATP excess in $CHCl_3$:MeOH (9:1).

$CHCl_3$:MeOH (9:1)

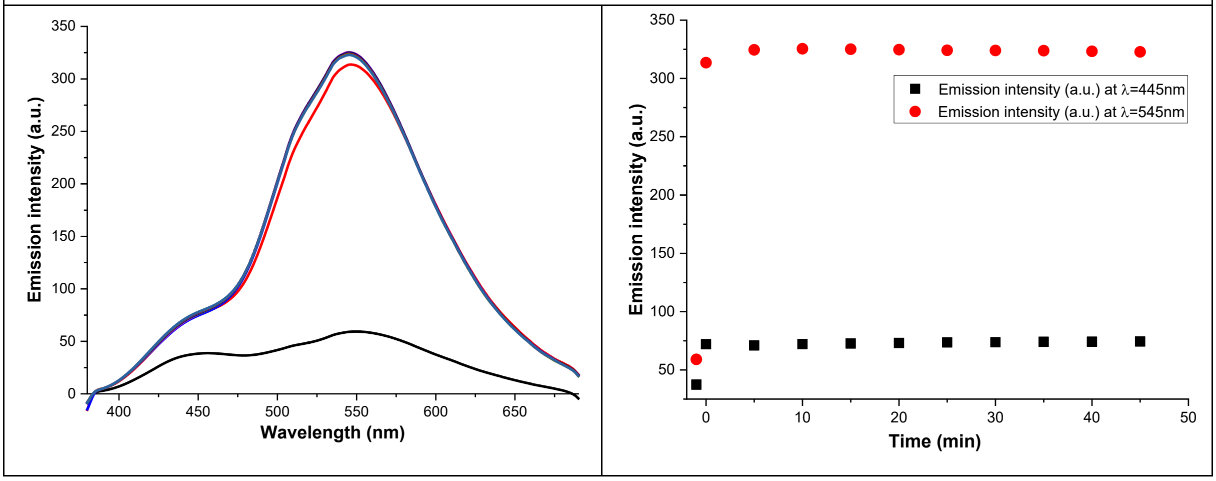

There was an increase in the emission intensity immediately after adding TATP. It was seemed that the probe itself increased its emission in this solvent.

Figure S157. Kinetic study at different times under visible (up) and UV (down) light in $CHCl_3$:MeOH (9:1). In each photo, left vial was contained the probe and right vial was contained the probe and an excess of TATP.

	**t=0**	**t=45 min**	**t=24 h**
**Visible light**	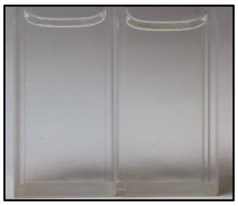	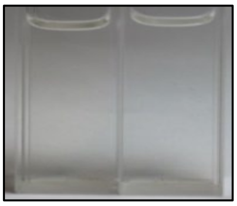	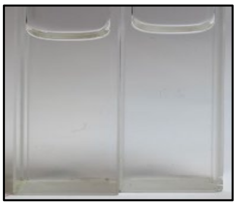
**UV light (366 nm)**	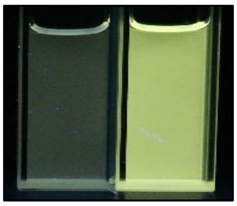	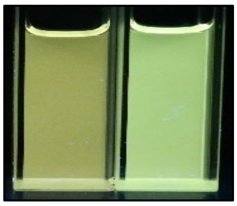	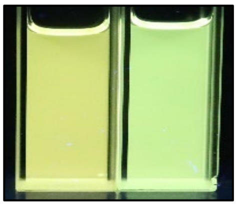

Titrations in toluene and LOD calculations:

Titration of AR82d with TATP in toluene.

The working concentration of AR82d was 1 μM and the TATP was directly added as a solid because when it was added in solution a strange and inconsistent behavior was observed. Its concentration was between 0 and 25 mM.

The measurements were carried out immediately after the addition of TATP. The excitation wavelength was 370 nm and the fluorescence changes were registered at 25ºC.

Figure S158. Titration (up left), fluorescence profile at 470 nm (up right) and calibration for the LOD calculation using emission values at 470 nm (down) of a 1 µM of AR82d solution in toluene under increasing concentrations of TATP.

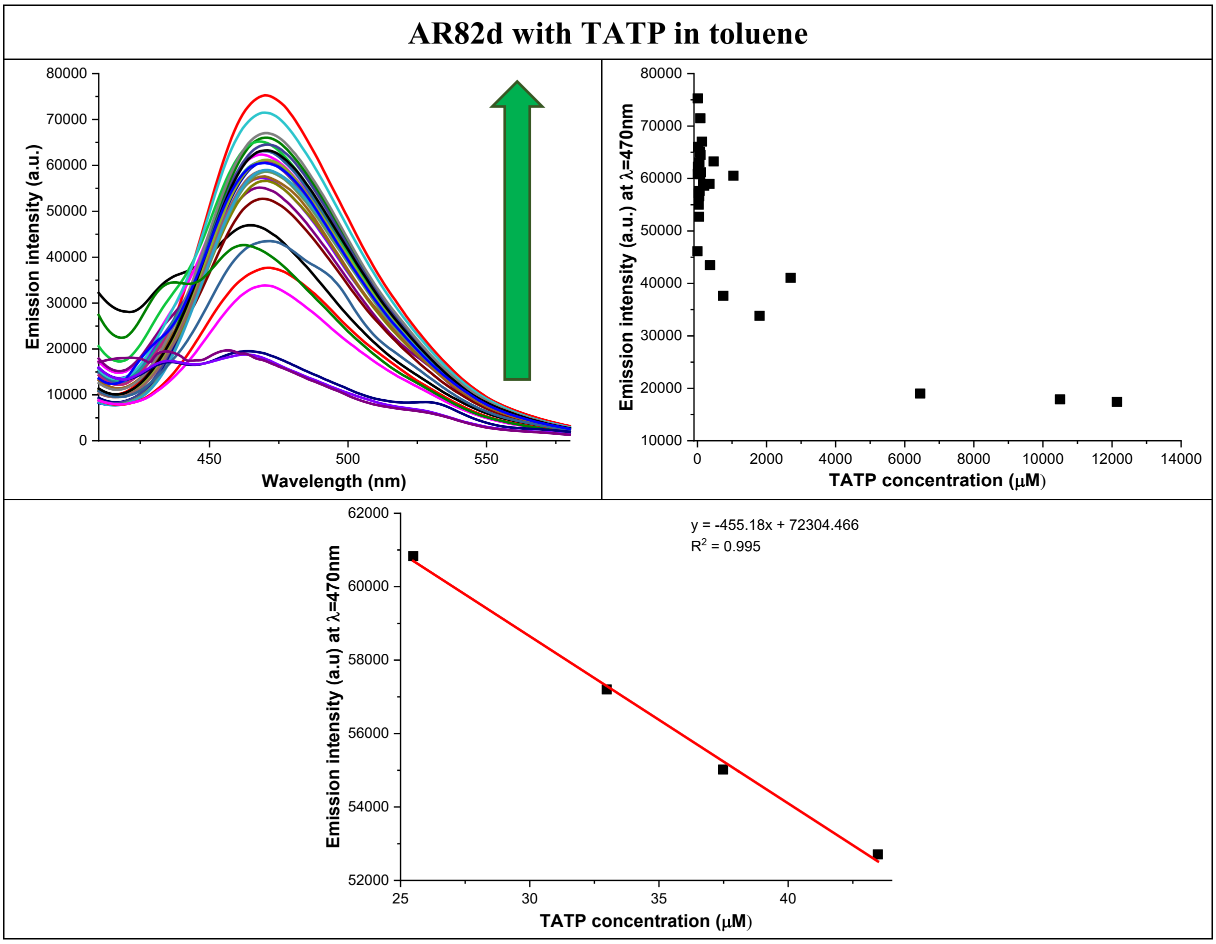

A decrease in the emission intensity was observed until it was stabilized around 6000 µM TATP. The LOD of AR82d with TATP was calculated. Calculation of the LOD by linear regression + false positive and negative at 470 nm.

For this method, the R software was used and the value of the LOD was 9.3 nM.

Finally, titration of 1 μM AR82d was performed with increasing amounts of m-CPBA in toluene. In this case, the oxidant was added in solution and the excitation wavelength was 360 nm. The measurements were also registered at 25ºC.

Figure S159. Titration (up left), fluorescence profile at 475 nm (up right) and calibration for the LOD calculation using emission values at 470 nm (down) of a 1 μM of AR82d solution in toluene under increasing concentrations of m-CPBA.

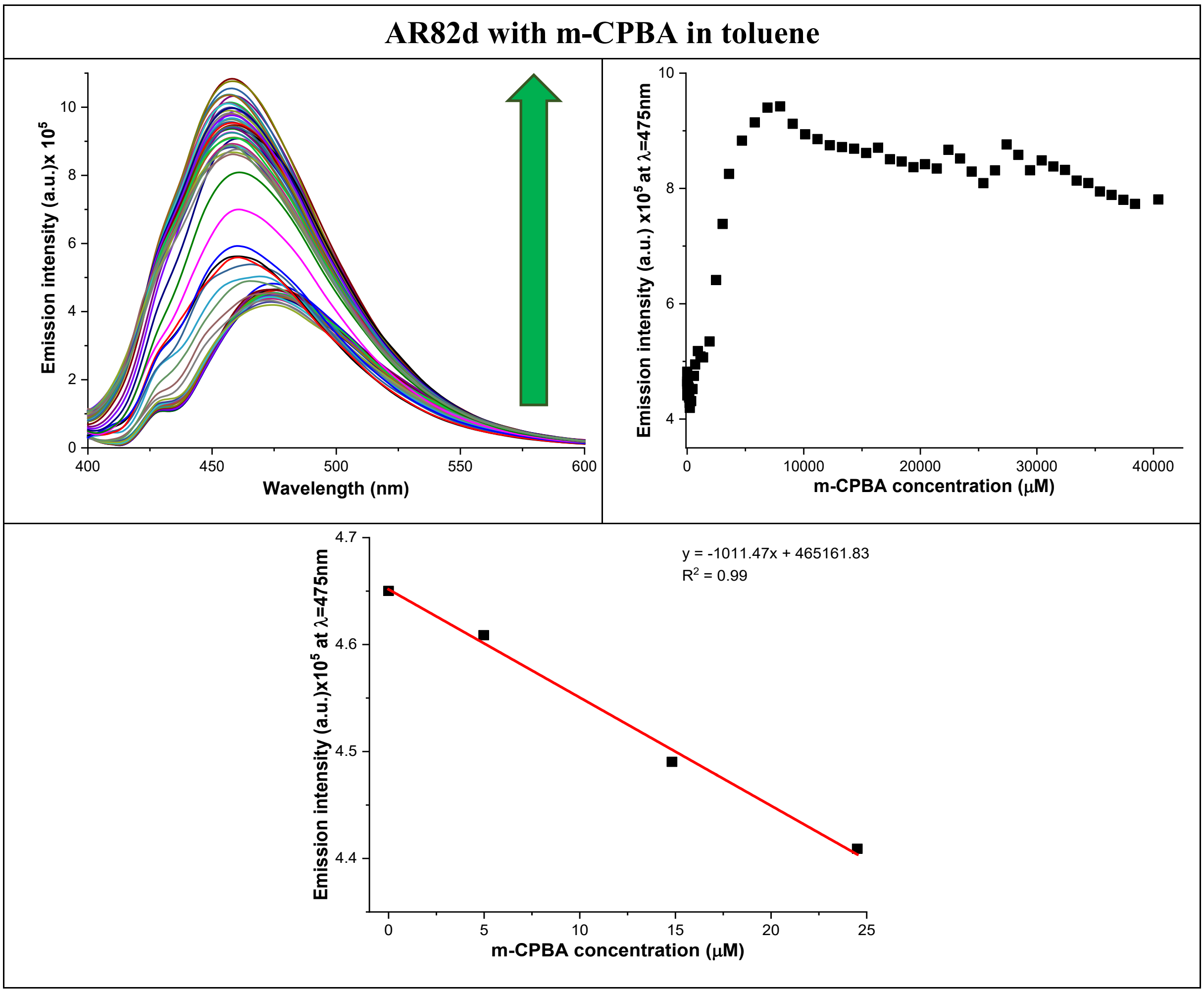

An increase in the emission intensity was observed until it was stabilized around 10000 μM m-CPBA. The LOD of AR82d with m-CPBA was calculated

Calculation of the LOD by linear regression + false positive and negative at 475 nm.

For this method, the R software was used and the value of the LOD was 2.16 nM.

TATP gas detection in modified nanoparticles:

Several nanoparticles samples were modified by adsorption of the probe and tested as potential TATP sensors. In all three cases, 100 mg of the corresponding nanoparticles and 1 mg of the probe were used to adsorption using the method described above:

- Modified silica nanoparticles (AR82d·SiO_2).
- Modified titanium (IV) oxide nanoparticles (AR82d·TiO_2).
- Modified titanium silicon oxide nanoparticles (AR82d·$SiTiO_4$).

The supported probe was evaluated by several methods. In a first instance, the color variation of the nanoparticles before and after exposure to TATP was tested. Afterwards, the detection capacity was evaluated by a more quantitative approach by registering changes in fluorescence intensity (by comparing the fluorescence quantum yield for higher precision and in the position of the maximum of emission. Finally, a quantitative study for the specific detection of TATP vapors was also performed.

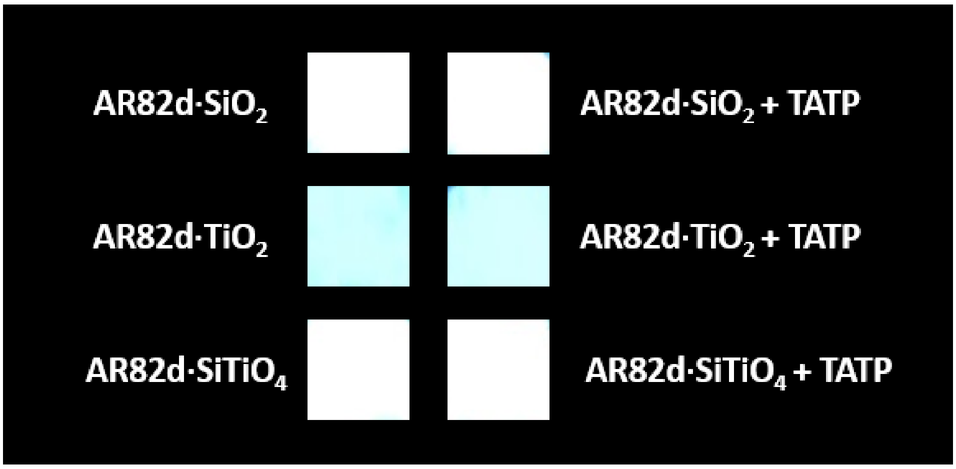

Figure S160. Color comparation between the nanoparticles with the probe over glass plates before and after the exposure to TATP.

The increase in fluorescence of solid was recorded by measuring the solids and determining the increase in fluorescence quantum yield (ØF). The most straightforward way to detect the presence of the analyte was the increase in fluorescence of the modified material.

The excitation wavelength for all experiments was 360 nm and the temperature for all test was 25ºC.

Figure S161. Response of fluorescence (down) and excitation (up) of AR82d adsorbed in SiO_2 nanoparticles.

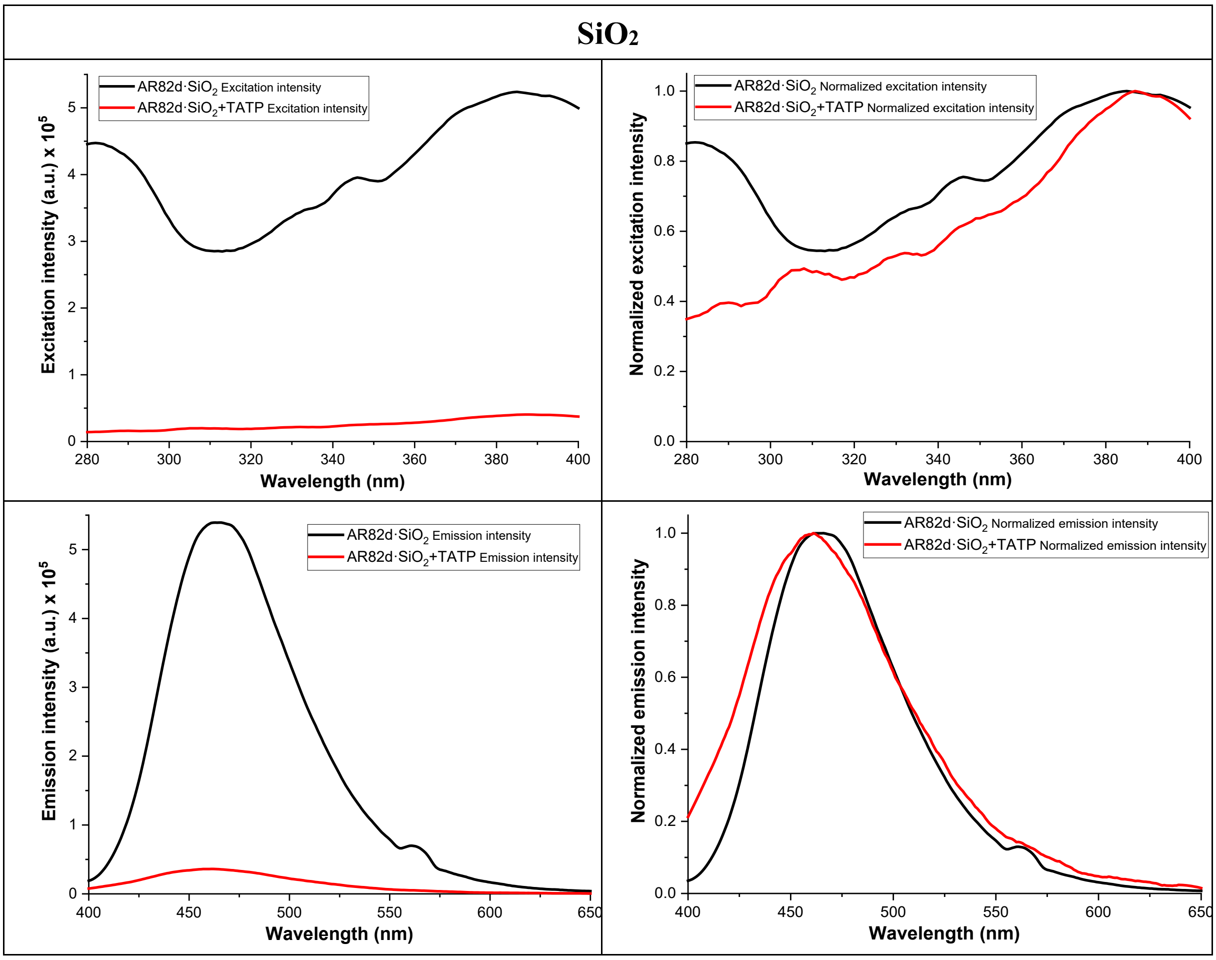

Figure S162. Response of fluorescence (down) and excitation (up) of AR82d adsorbed in TiO_2 nanoparticles.

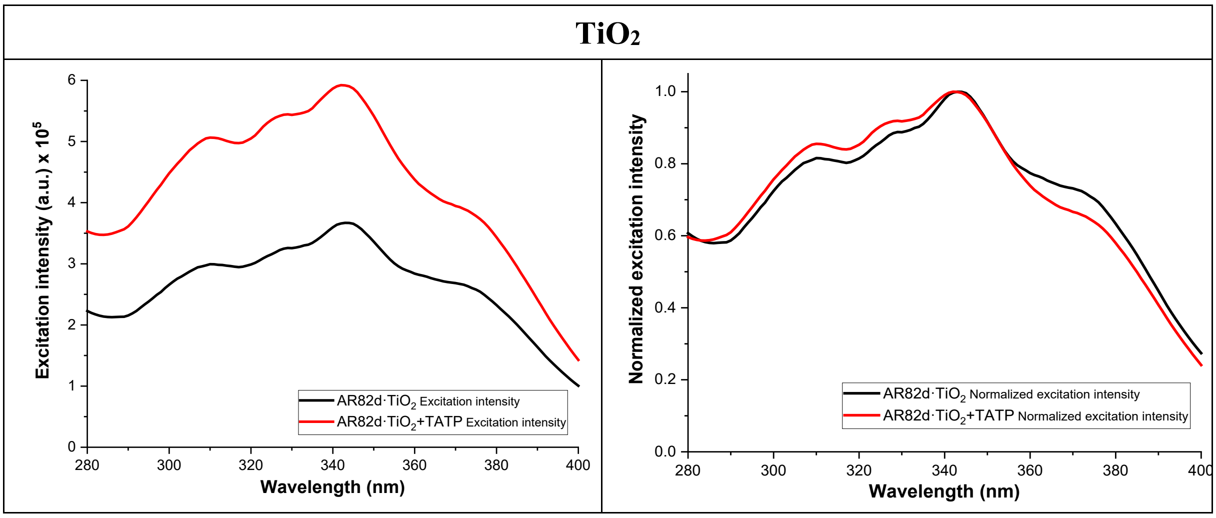

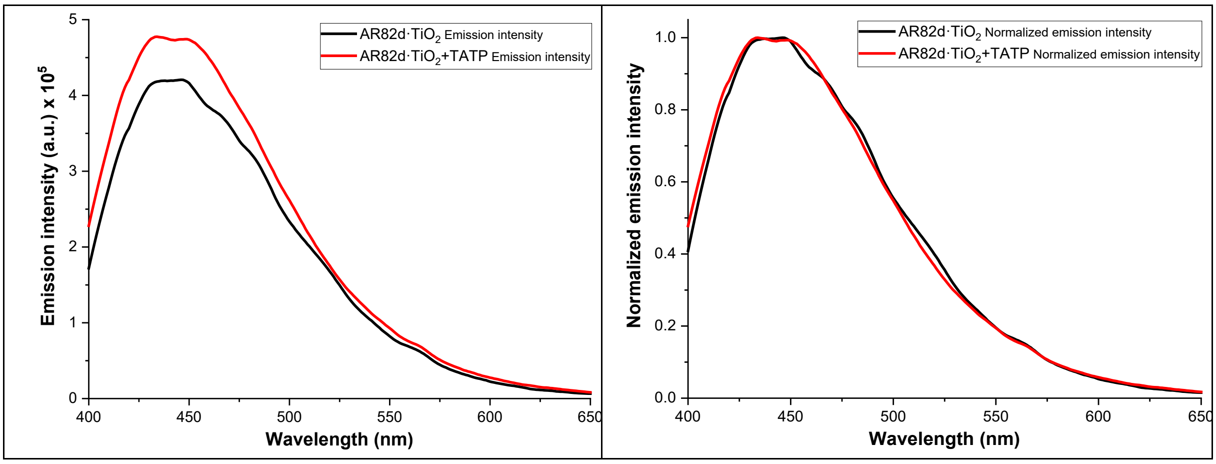

Figure S163. Response of fluorescence(down) and excitation (up) of AR82d adsorbed in $SiTiO_4$ nanoparticles.

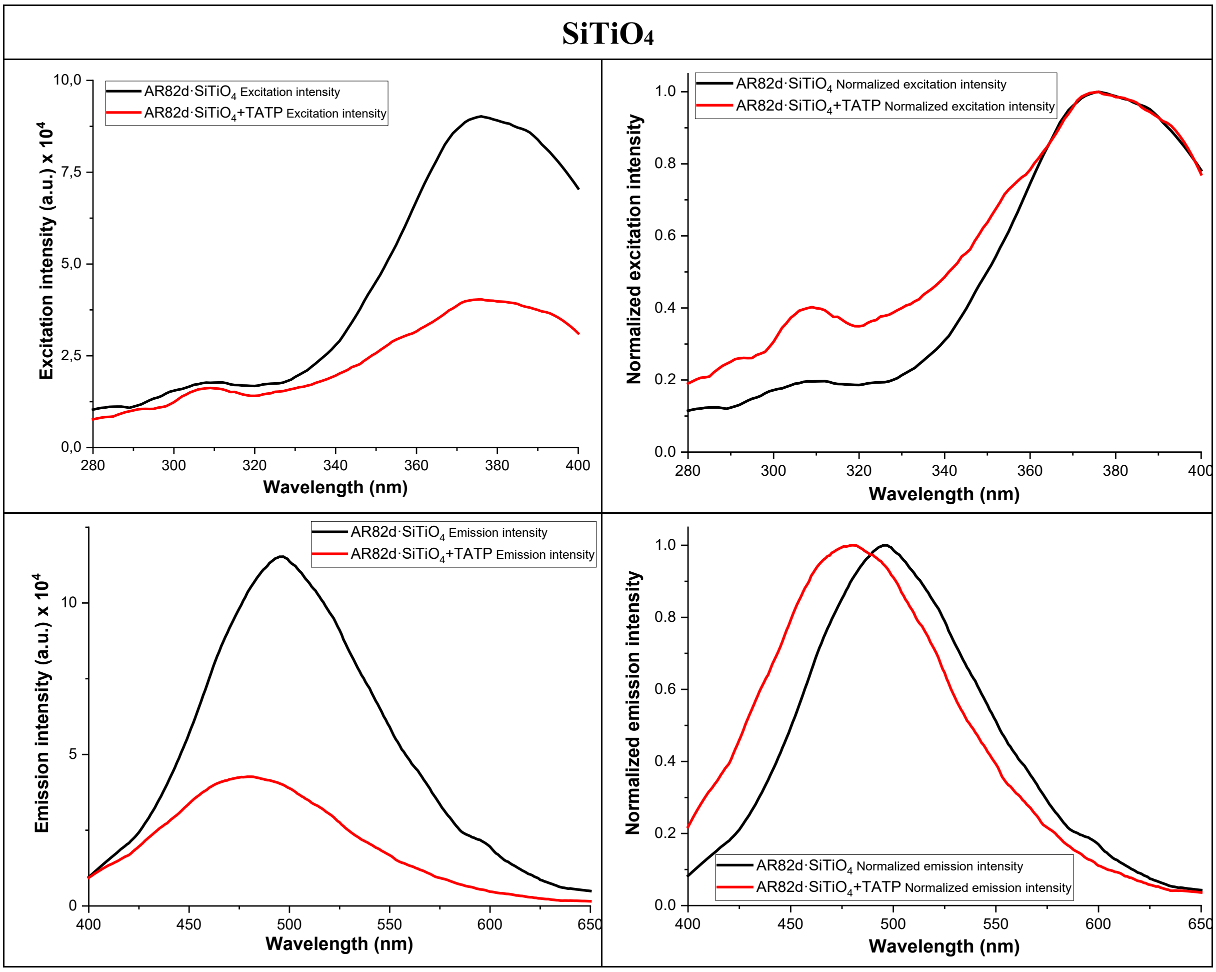

As it could be seen in the excitation spectra, the maximum for AR82d·TiO_2 (342 nm) was differed respecting the maximum for AR82d·SiO_2 and AR82d·$SiTiO_4$ (380 nm) and the one previously obtained for the compound in solution (360 nm). Emission measurements were made at the aforementioned wavelengths with similar results.

In doing so, it was necessary to have a fluorometer provided with an integration sphere. The accuracy of this method was checked by measuring several replicas, obtaining errors of less than 2 %.

Figure S164. Variation in fluorescence between the initial and after being under excess of TATP

	$Ø_{Probe+TATP}$	$Ø_{Probe}$	$Ø_{Probe+TATP}/Ø_{Probe}$
**AR82d·SiO_2**	<2%	18.6%	0.11
**AR82d·TiO_2**	2.75%	2.72%	1.01
**AR82d·$SiTiO_4$**	3.15%	11.1%	0.27

In general, we could conclude that quantum yields followed the pattern seen in the previously shown emission intensity spectra. In the case of AR82d·SiO2 and AR82d·SiTiO4, it decreased after exposure to TATP while in the case of AR82d TiO2, it increased very slightly. However, vapor phase detection tests were not continued due to problems of solubility and subsequent adsorption onto the nanoparticles of the compound AR82d.

Synthesis of *N*-(*N'*-Boc-piperidin-4-yl)-4-[2-(4-Boc-piperazin-1-yl)pyridin-5-yl]naphthalene-1,8-dicarboxylmonoimide (AR83p)

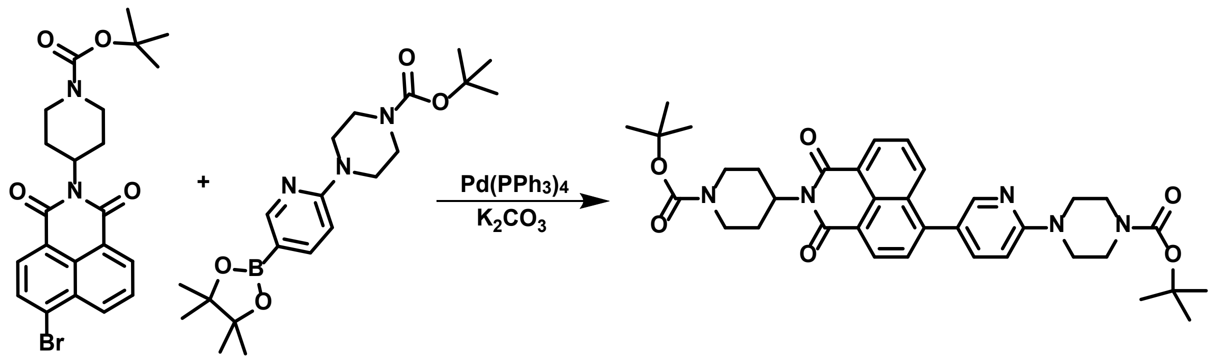

250 mg of *N*-(*N'*-Boc-piperidin-4-yl)-4-bromonaphthalene-1,8-dicarboxylmonoimide (AR43p, 0.54 mmol), 221 mg of 2-(4-Boc-piperazin-1-yl)pyridin-5-yl boronic acid pinacol ester (0.57 mmol) and 752 mg of potassium carbonate (5.44 mmol) were dissolved in a mixture of toluene:butanol:water (4:1:2 ml) under nitrogen atmosphere and the catalyst, $Pd(PPh_3)_4$ (5% mmol) was added. The resulting mixture was stirred overnight at 110 ºC. Afterwards, the solvent was evaporated under reduce pressure and the solid was dissolved in DCM and washed with water (3×100 ml). The organic extracts were combined, dried over anhydrous Na_2SO_4 and the solvent was evaporated under reduce pressure. The organic solid was purified by column chromatography (SiO_2, DCM:MeOH 50:1) getting 256 mg of AR83p, yellow solid, 74% yield. m.p.: 244 – 247 ºC. IR (ATR, cm^{-1}): 2973 – 2841, 1693 (C=O), 1677 (C=O), 1650 (C=O), 1585, 1495, 1453 (C-O), 1408, 1348, 1235, 1159 (C-N), 1103, 1034, 995, 932, 899, 861, 777, 755, 630, 454. ^{1}H-NMR (300 MHz, $CDCl_3$) δ (ppm): 8.49 (d, J = 7.6 Hz, 2H, 2×H_{Ar}), 8.31 – 8.19 (m, 2H, 2×H_{Ar}), 7.67 – 7.52 (m, 3H, 3×H_{Ar}), 6.76 (d, J = 8.8 Hz, 1H, H_{Ar}), 5.11 (m, 1H, CH), 4.24 (s, 2H, CH_2), 3.58 (m, 8H, 4×CH_2), 2.87 – 2.61 (m, 4H, 2×CH_2), 1.61 (m, 2H, CH_2), 1.44 (d, J = 2.4 Hz, 18H, 6×CH_3). ^{13}C-NMR (75MHz, $CDCl_3$) δ (ppm): 164.4 (C=O), 164.2 (C=O), 158.7 (C_{Ar}), 154.7 (C_{Ar}), 148.6 (C_{Ar}H), 143.6 (C_{Ar}), 138.9 (C_{Ar}H), 132.0 (C_{Ar}H), 131.2 (C_{Ar}H), 130.9 (C_{Ar}H), 129.8 (C_{Ar}), 128.8 (C_{Ar}), 127.5 (C_{Ar}H), 126.8 (C_{Ar}H), 123.7 (C_{Ar}), 123.2 (C_{Ar}), 121.6 (C_{Ar}), 106.5 (C_{Ar}H), 80.0 (Cq), 79.4 (Cq), 51.7 (CH), 44.8 (CH_2), 43.6 (CH_2), 28.5 (CH_3), 28.4 (CH_3), 28.2 (CH_2). HRMS (MALDI, DIT-) m/z: calculated for $C_{36}H_{43}N_5O_6$: 641.3208 (M^-); found 641.3232. UV-Vis ($CHCl_3$), λ_{max} nm (log ε): 390 (3.9). Ø (Hex, %): 85.40. τ (375 nm, Hex, ns): 3.199 (χ^2: 1.153).

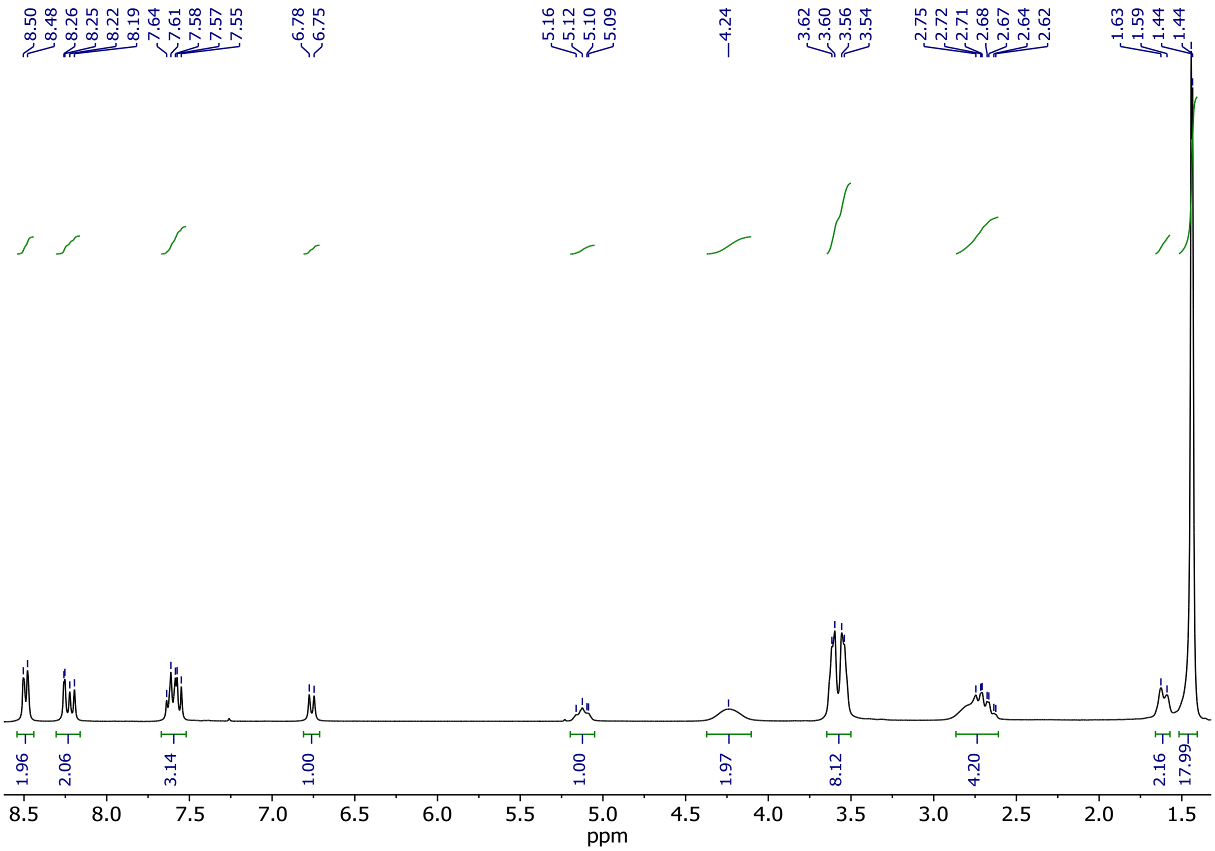

Figure S165. ^{1}H-NMR ($CDCl_3$, 300 MHz) of AR83p.

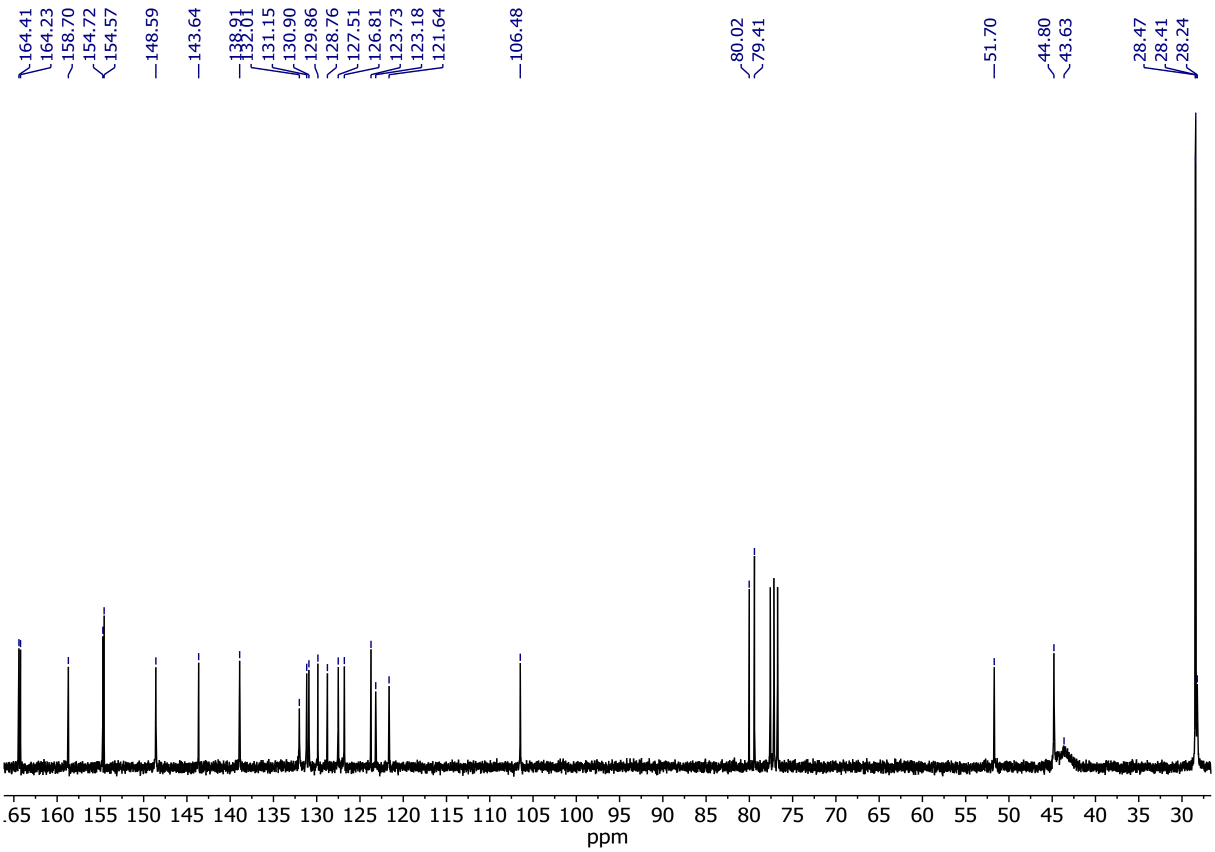

Figure S166. ^{13}C-NMR ($CDCl_3$, 75 MHz) of AR83p.

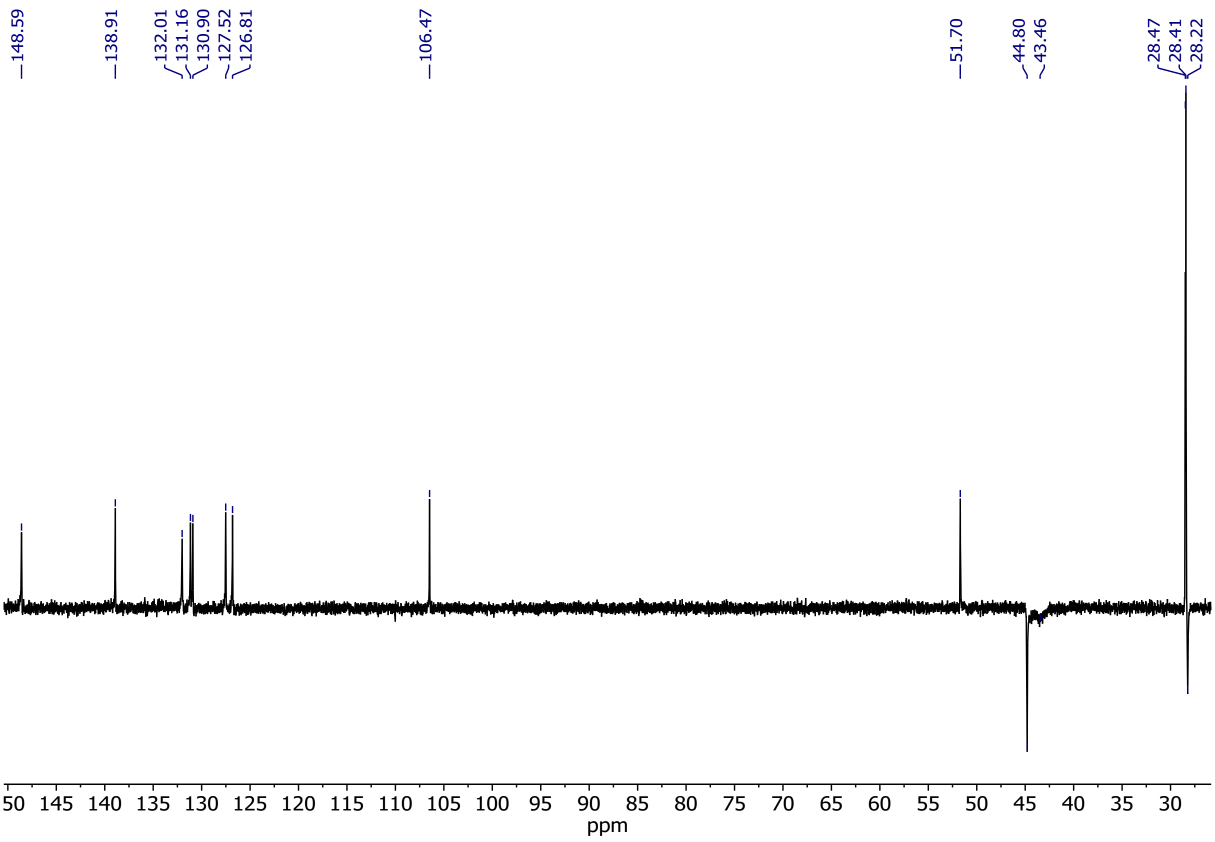

Figure S167. DEPT NMR ($CDCl_3$, 75 MHz) of AR83p.

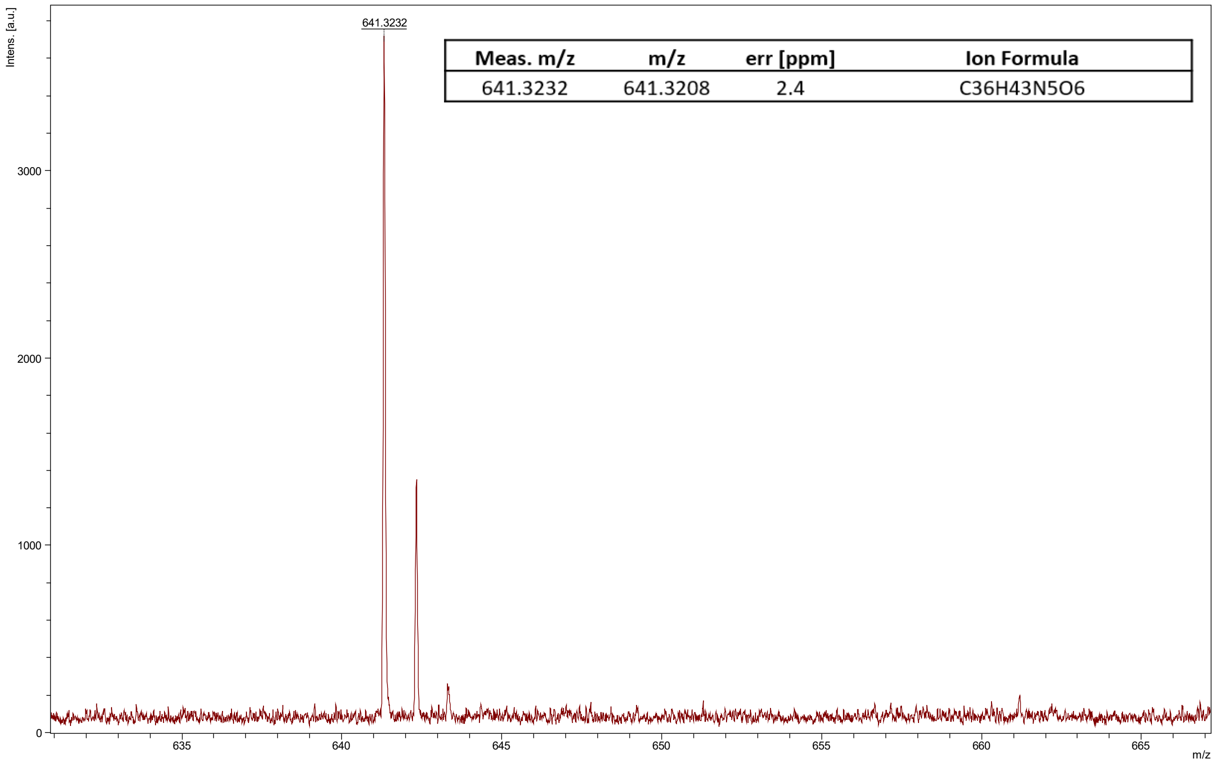

Meas. m/z	m/z	err [ppm]	Ion Formula
641.3232	641.3208	2.4	C36H43N5O6

Figure S168. HRMS (MALDI, DIT-) of AR83p

Solvatochromism:

The concentration of the compound was 20 µM and the excitation wavelength was 380 nm.

Figure S169. Solvatochromism of AR83p.

Absorbance	Normalized absorbance
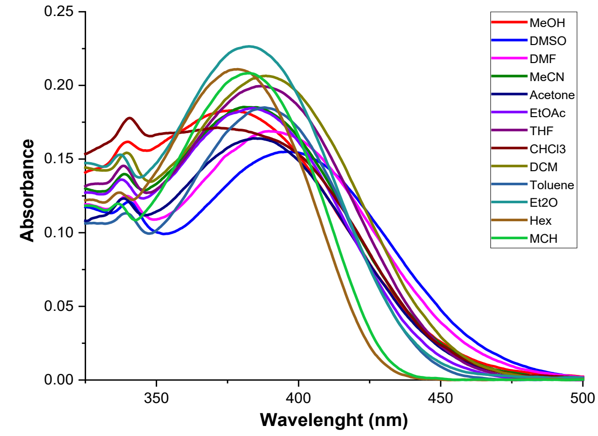	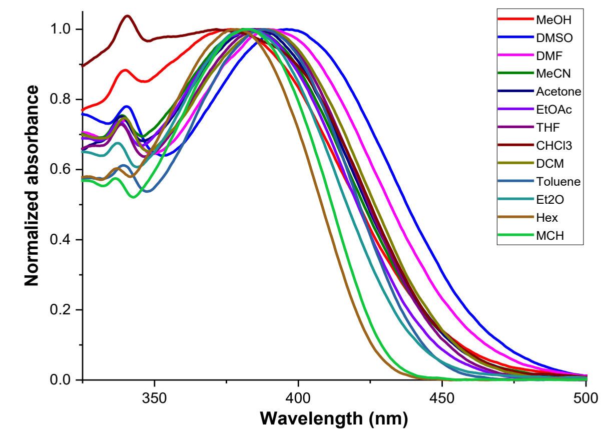
**Emission**	**Normalized emission**
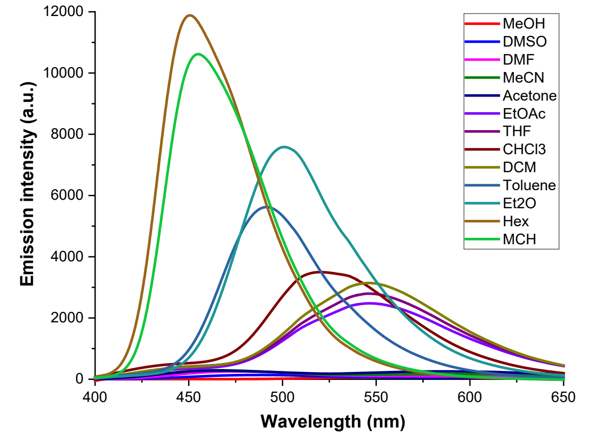	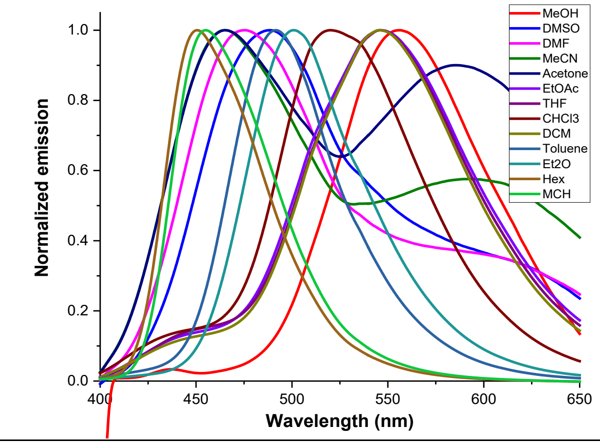

Figure S170. Solvatochromism of AR83p under visible and 366 nm light.

	H_2O MeOH DMSO DMF MeCN Acet. EtOAc THF $CHCl_3$ DCM Tol. Et_2O Hex MCH
**Visible light**	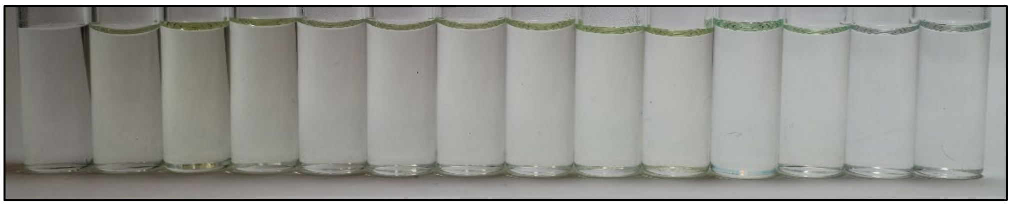
**UV light (366 nm)**	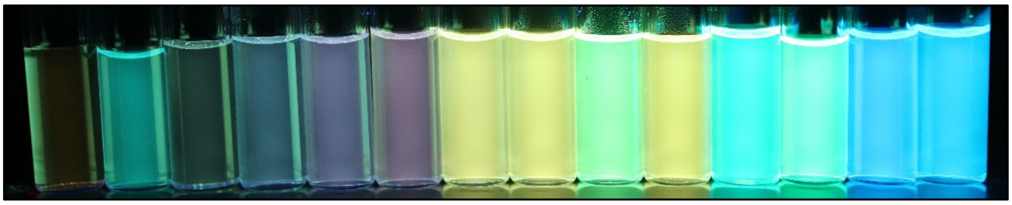

Fluorescence decay lifetime (τ):

The solvent used was hexane. The concentration of the compound was 20 µM.

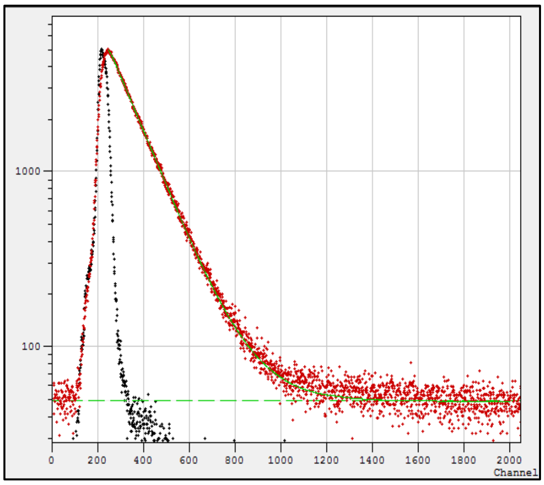

Figure S171. Fluorescence decay lifetime of AR83p.

Water acceptance test:

The solvent used was MeOH and the concentration of the compound was 20 µM.

Figure S172. Water acceptance test, AR83p in MeOH under visible (up) and UV (down) light.

	**R 5% 10% 20% 30% 40% 50% 60% 70% 80% 90%**
**Visible light**	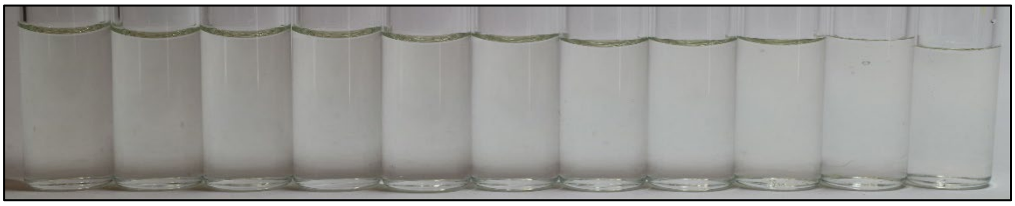
**UV light (366 nm)**	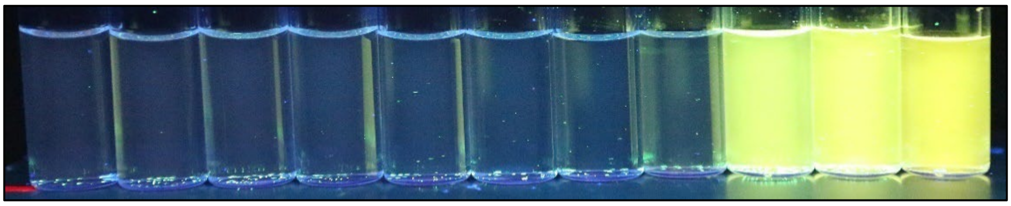

There was a strong increase in emission intensity from 70% water (AIE), which also coincided with what was observed using THF as solvent. In addition, using THF, there was a shift of the emission maximum from the red – purple region (580 – 650 nm) to the yellow-greenish region (550 – 570 nm).

Figure S173. Water acceptance test of AR83p in THF under visible (up) and UV (down) light.

	**R 5% 10% 20% 30% 40% 50% 60% 70% 80% 90%**
**Visible light**	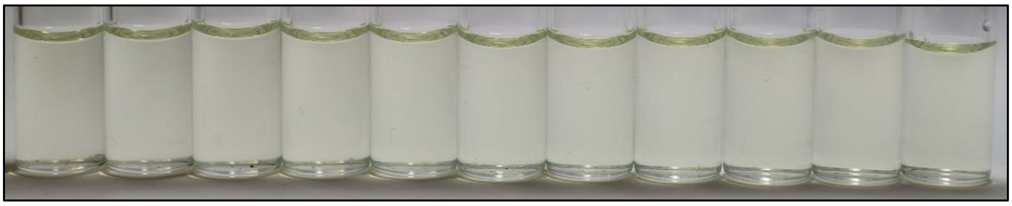

**UV light (366 nm)**	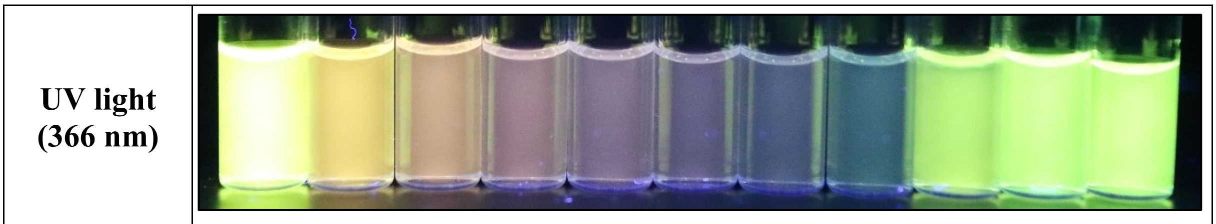

Figure S174. Emission intensity graph (left) and graph of relationship between the emission intensity and the wavelenght of the maximum of emission (right) as a function of the amount of water added for AR83p using THF as a solvent.

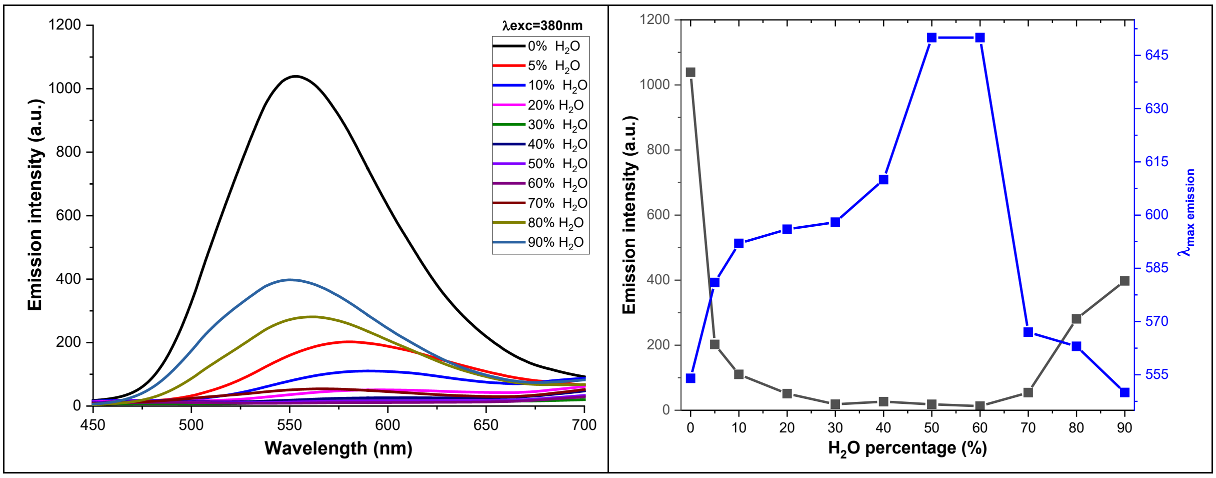

pH test:

The amount of water that the compound could accept was 90%. The concentration of the compound was 20 µM and the solvent used was MeOH.

Figure S175. pH test of AR83p under visible (up) and UV (down) light.

	R	HCl*	3.4	4.8	6.4	7.4	7.9	10.5	KOH
**Visible light**	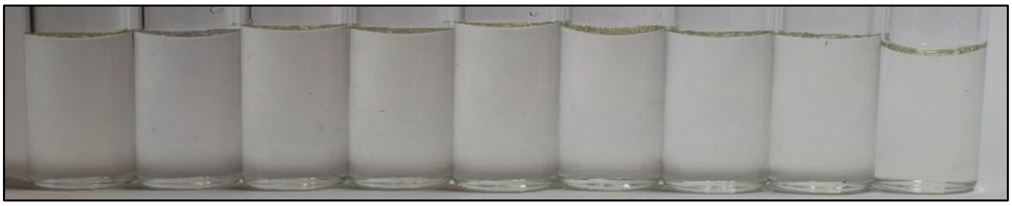								
**UV light (366 nm)**	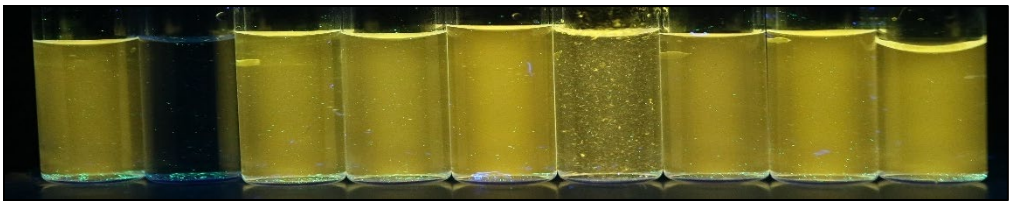								

*HCl is included in all experiments in a close position to HNO_3 to distinguish between the redox action of HNO_3 and a possible effect due only to the concomitant acidity of nitric acid.

There was a quenching in the emission at very acidic pH, while at physiological pH, the compound precipitated.

Cations and anions test:

The concentration of the compound was 20 µM and the solvent used was a mixture of MeOH and water (90%).

Figure S176. Cations test of AR83p under visible (up) and UV (down) light.

	R Ag^{+} Ni^{2+} Sn^{2+} Cd^{2+} Zn^{2+} Pb^{2+} Cu^{2+} Fe^{3+} Sc^{3+} Al^{3+} Hg^{2+} Au^{3+} Co^{2+} Pd^{2+} Ir^{3+} Cu^{+} Ru^{3+} Pt^{2+}
**Visible light**	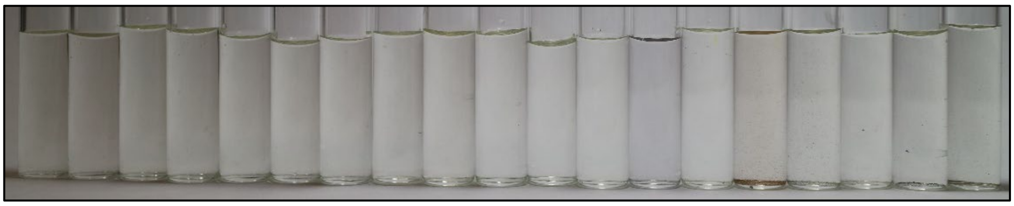
**UV light (366 nm)**	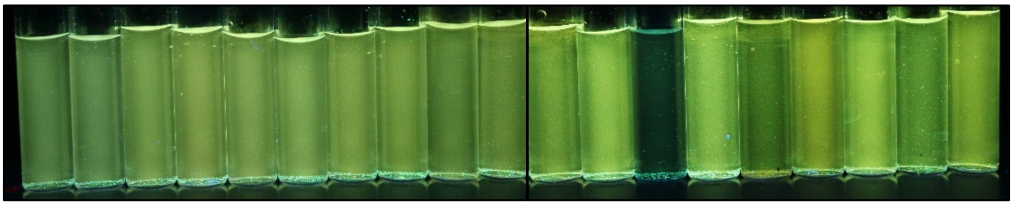

There was the quenching of the fluorescence in the presence of Au^{3+} under UV light.
The concentration was 20 µM and the solvent used was a mixture of MeOH and water (90%).

Figure S177. Anions test of AR83p under visible (up) and UV (down) light.

	R F^{-} Cl^{-} Br^{-} I^{-} BzO^{-} NO_3^{-} $H_2PO_4^{-}$ HSO_4^{-} AcO^{-} CN^{-} SCN^{-}
**Visible light**	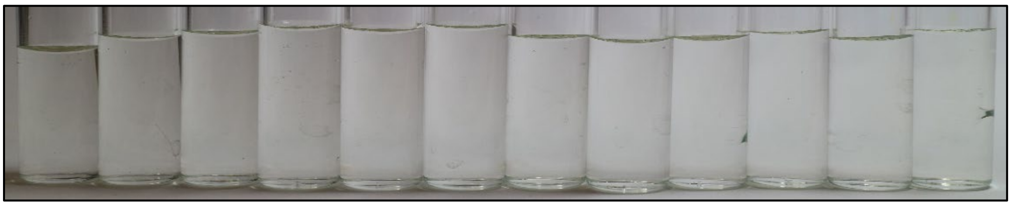
**UV light (366 nm)**	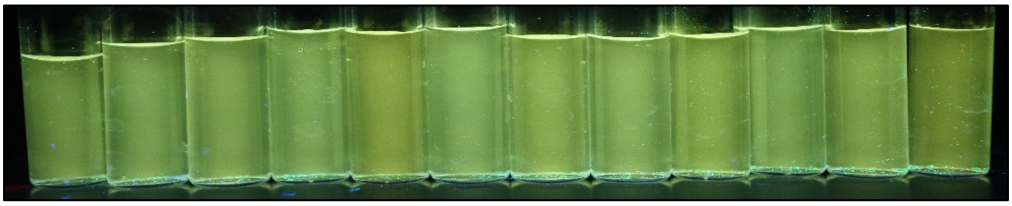

Oxidants and reductants test:

Solvent was a mixture of MeOH-water (90%) and concentration of the compound was 20 µM.

Figure S178. Oxidants and reductants test of AR83p under visible (up) and UV (down) light.

	R HCl* HNO_3 m-CPBA Oxn Hz H_2O_2 TNB TNT TATP HMTD
**Visible light**	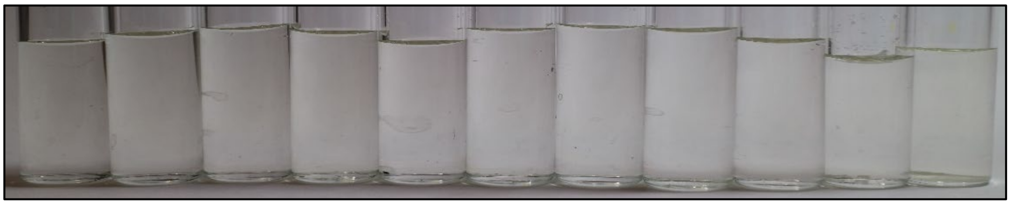
**UV light (366 nm)**	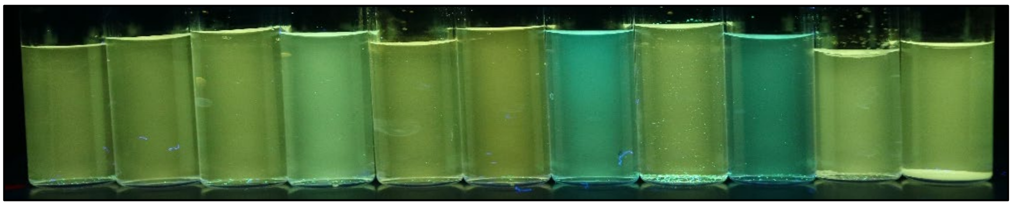

*HCl is included in all experiments in a close position to HNO_3 to distinguish between the redox action of HNO_3 and a possible effect due only to the concomitant acidity of nitric acid. No significant changes were observed.

Synthesis of *N*-(piperidin-4-yl)-4-[2-(4-Boc-piperazin-1-yl)pyridin-5-yl]naphthalene-1,8-dicarboxylmonoimide (AR83s)

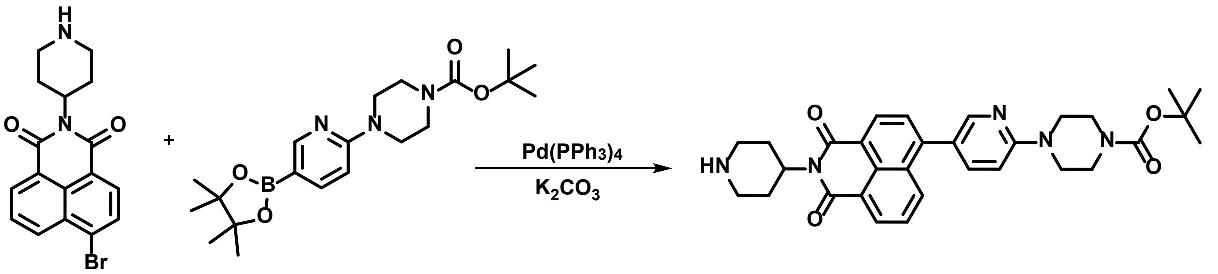

150 mg of *N*-(piperidin-4-yl)-4-bromonaphthalene-1,8-dicarboxylmonoimide (AR43d, 0.42 mmol), 171 mg of 2-(4-Boc-piperazin-1-yl)pyridin-5-yl boronic acid pinacol ester (0.44 mmol) and 451 mg of potassium carbonate (3.26 mmol) were dissolved in a mixture of toluene:butanol:water (4:1:2 ml) under nitrogen atmosphere and the catalyst, $Pd(PPh_3)_4$ (5% mmol)was added. After that it was stirred overnight at 110 ºC, the solvent was evaporated under reduce pressure, the solid was dissolved in CH_2Cl_2 and washed with water (3×100 ml). The organic extracts were combined, dried over anhydrous Na_2SO_4 and the solvent was evaporated under reduce pressure. The organic solid was purified by column chromatography (SiO_2, CH_2Cl_2:MeOH 50:1) getting 113 mg of AR83s, brown solid, 50% yield. m.p.: 216 – 218 ºC (decomposition). IR (ATR, cm^{-1}): 3425 (N-H), 2970 – 2851, 1689 (C=O), 1585, 1405 (C-O), 1351, 1238, 1118 (C-N), 992, 929, 753, 717, 693, 535. ^{1}H-NMR (300 MHz, $CDCl_3$) δ (ppm): 8.60 (d, J = 7.5 Hz, 2H, 2×H_{Ar}), 8.30 (m, 2H, 2×H_{Ar}), 7.72 – 7.61 (m, 3H, 3×H_{Ar}), 6.80 (d, J = 9.0 Hz, 1H, H_{Ar}), 5.40 – 5.25 (m, 1H, CH), 3.83 (s, 2H, CH_2), 3.69 – 3.57 (m, 8H, 4×CH_2), 3.41 – 3.09 (m, 5H, 2×CH_2 y NH), 1.97 (d, J = 11.1 Hz, 2H, CH_2), 1.50 (s, 9H, 3×CH_3). ^{13}C-NMR (75 MHz, $CDCl_3$) δ (ppm): 164.4 (C=O), 164.2 (C=O), 158.9 (C_{Ar}), 154.9 (C_{Ar}), 148.7 (C_{Ar}H), 144.1 (C_{Ar}), 139.1 (C_{Ar}H), 132.4 (C_{Ar}H), 131.7 (C_{Ar}H), 131.4 (C_{Ar}H), 130.1 (C_{Ar}), 128.9 (C_{Ar}), 127.8 (C_{Ar}H), 127.0 (C_{Ar}H), 123.8 (C_{Ar}), 123.0 (C_{Ar}), 121.5 (C_{Ar}), 106.6 (C_{Ar}H), 80.3 (Cq), 48.9 (CH), 44.9 (CH_2), 43.3 (CH_2), 28.5 (CH_3), 25.8 (CH_2). HRMS (MALDI, DIT+) m/z: calculated for $C_{31}H_{35}N_5O_4$: 543.2714 (M^+ + 2); found 543.2748. UV-Vis ($CHCl_3$), λ_{max} nm (log ε): 415 (3.9). Ø ($CHCl_3$, %): 99.41. τ (375 nm, $CHCl_3$, ns): 5.291 (χ^2: 1.040).

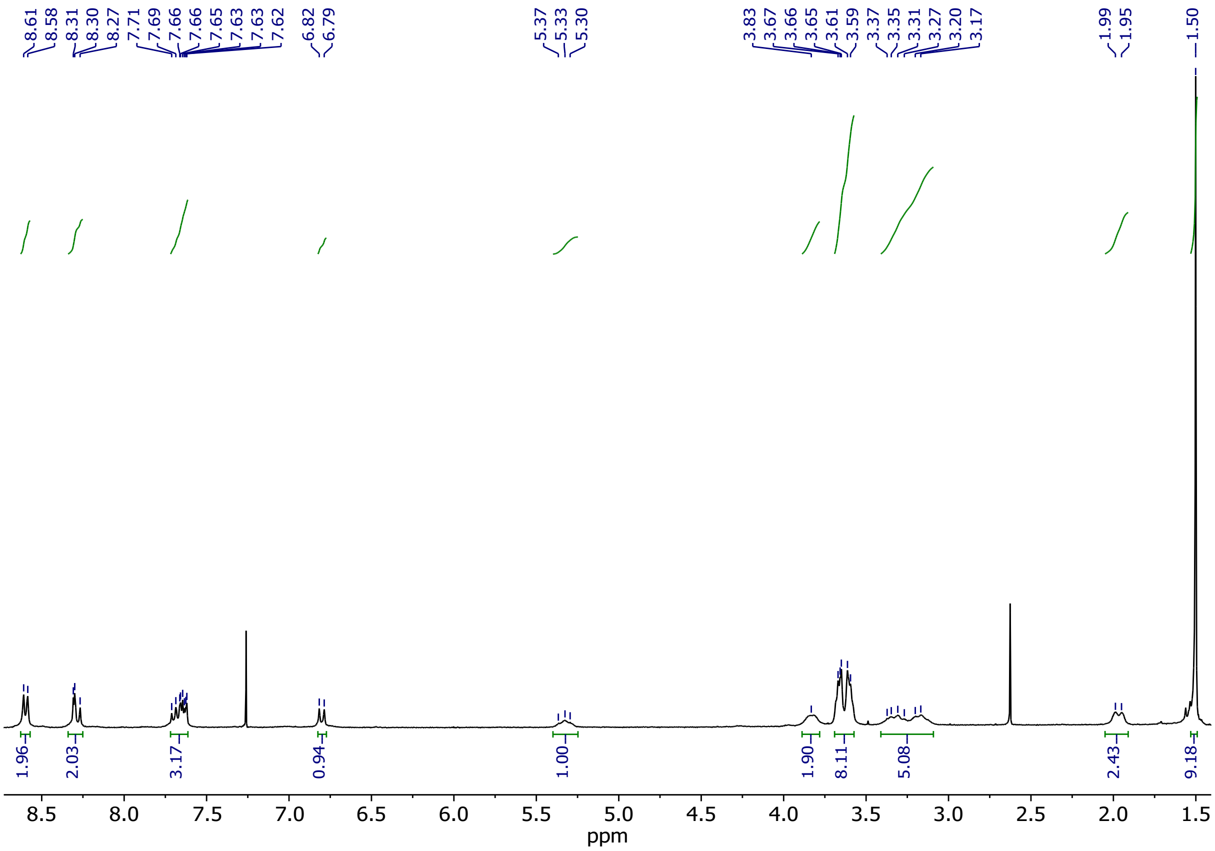

Figure S179. ^{1}H-NMR (CDCl$_3$, 300 MHz) of AR83s.

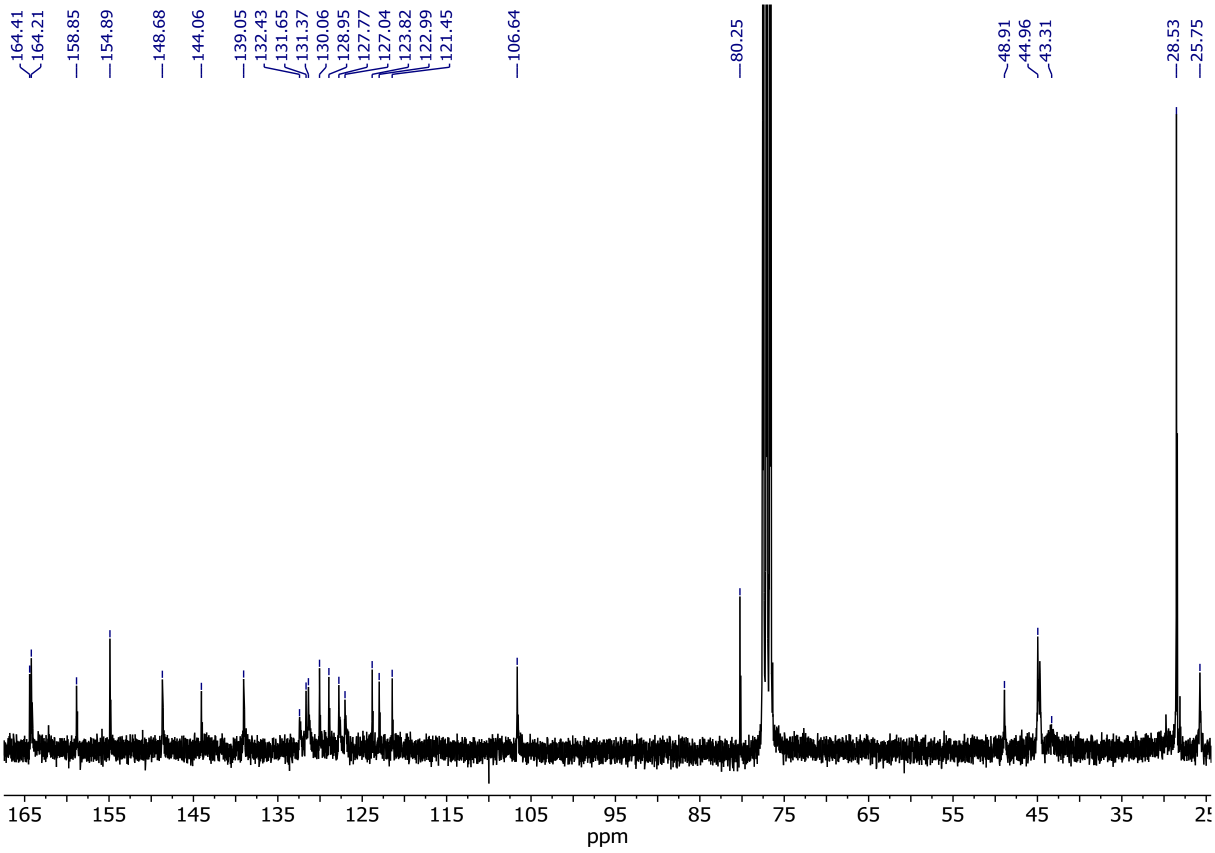

Figure S180. ^{13}C-NMR (CDCl$_3$, 75 MHz) of AR83s.

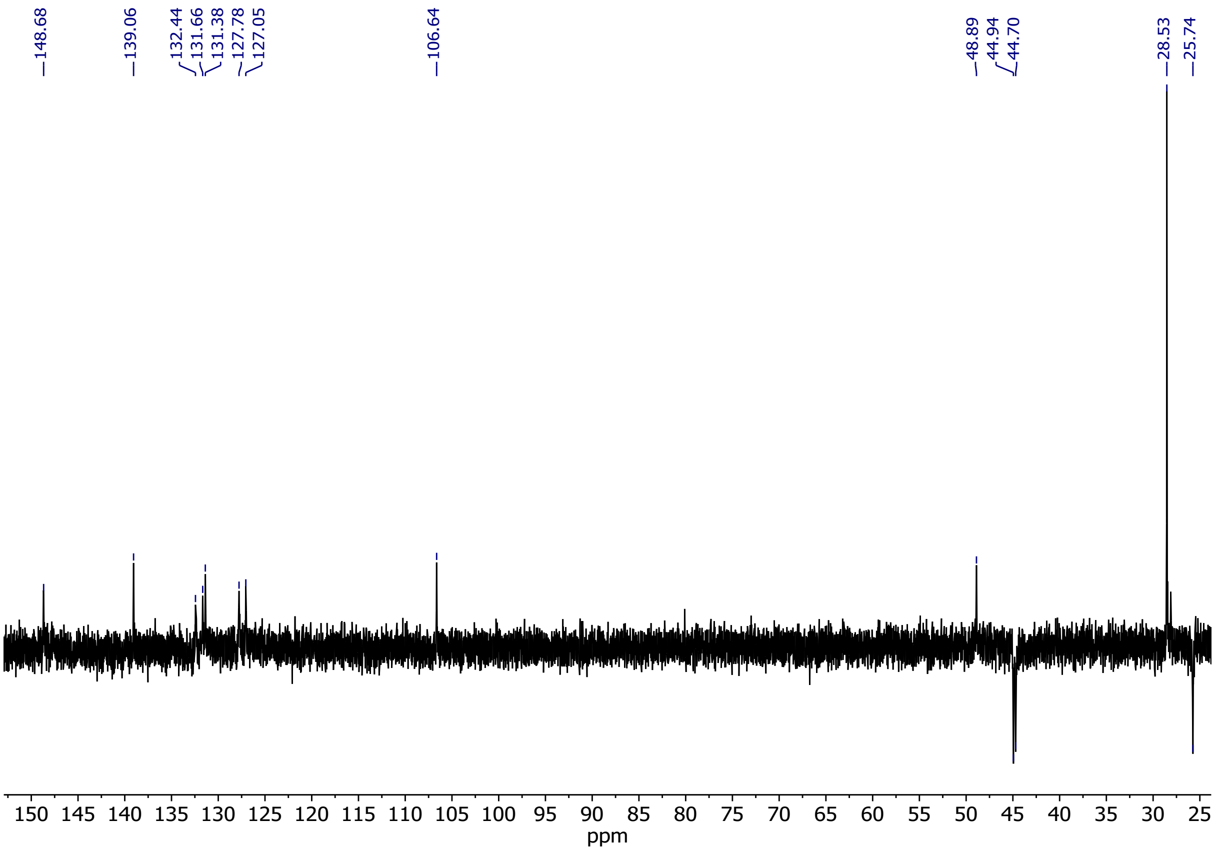

Figure S181. DEPT NMR ($CDCl_3$, 75 MHz) of AR83s.

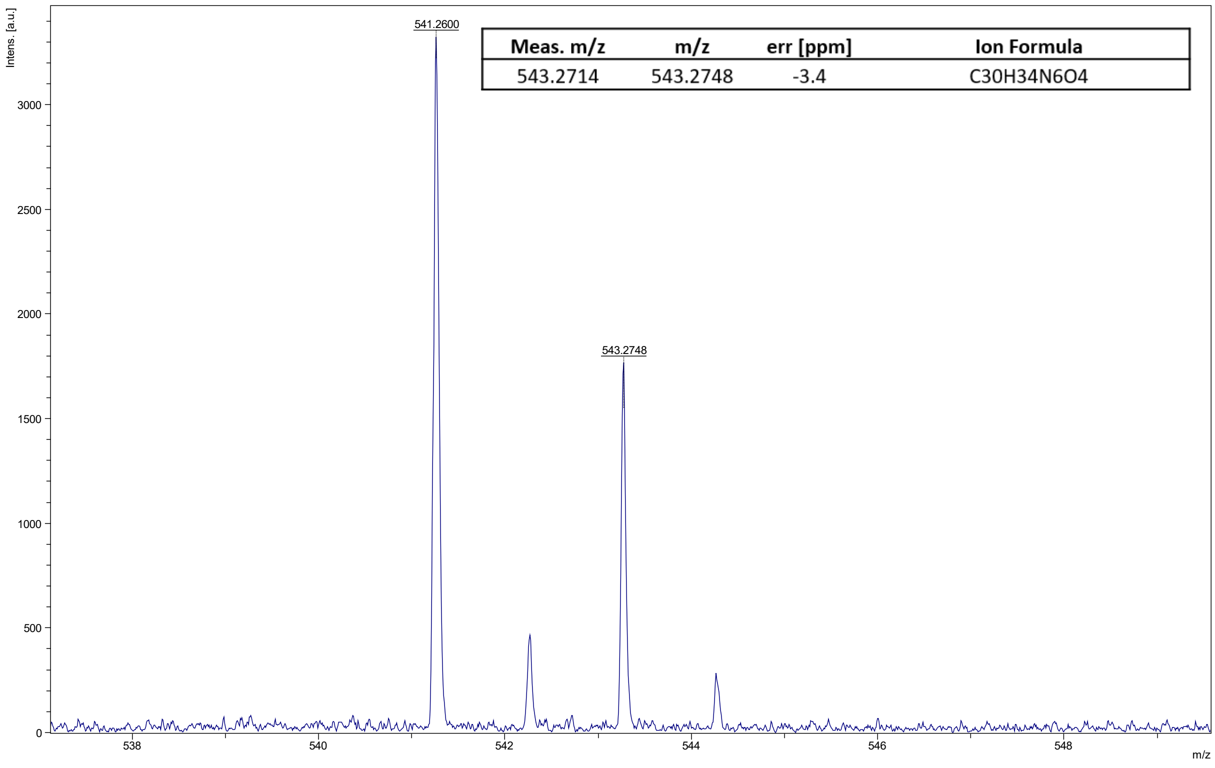

Meas. m/z	m/z	err [ppm]	Ion Formula
543.2714	543.2748	-3.4	C30H34N6O4

Figure S182. HRMS (MALDI, DIT+) of AR83s.

Solvatochromism:

The concentration of the compound was 20 µM and the excitation wavelength was 390 nm.

Figure S183. Solvatochromism of AR83s.

Absorbance	Normalized absorbance
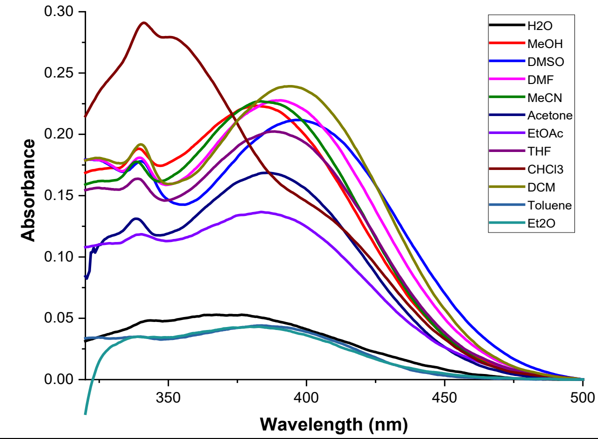	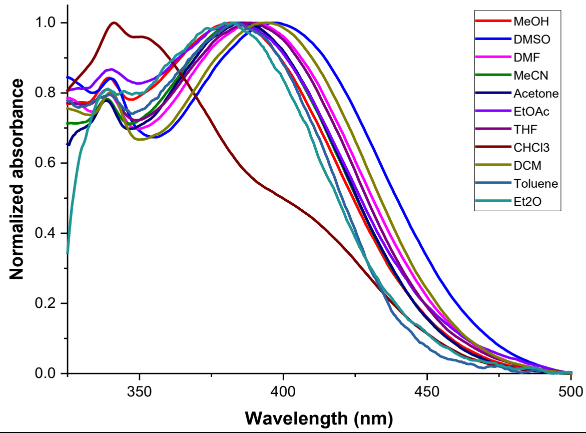
**Emission**	**Normalized emission**
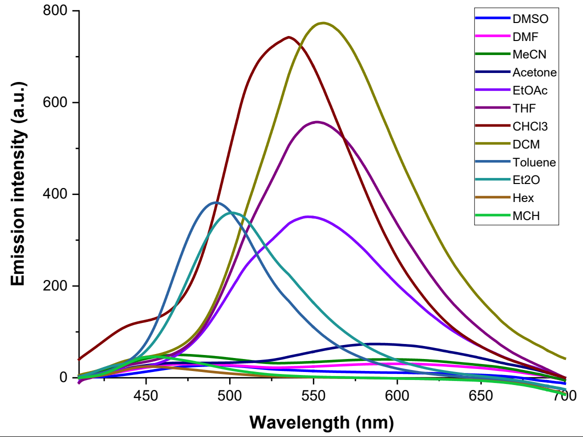	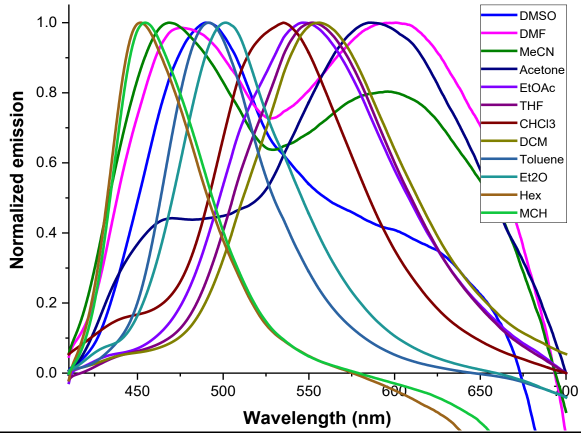

Figure S184. Solvatochromism of AR83s under visible and 366 nm light.

	H_2O MeOH DMSO DMF MeCN Acet. EtOAc THF $CHCl_3$ DCM Tol. Et_2O Hex MCH
**Visible light**	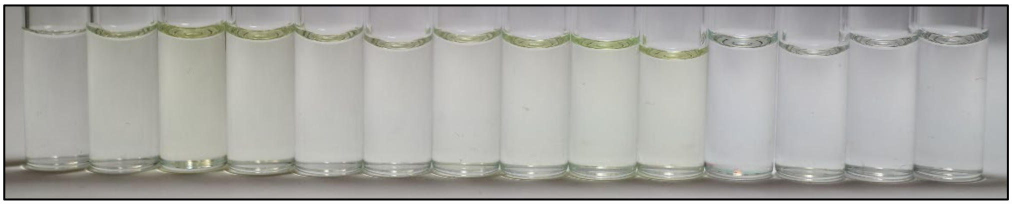
**UV light (366 nm)**	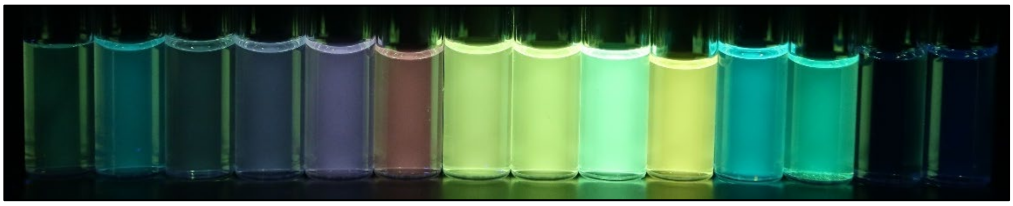

Fluorescence decay lifetime (τ):

The solvent used was chloroform. The concentration of the compound was 20 μM.

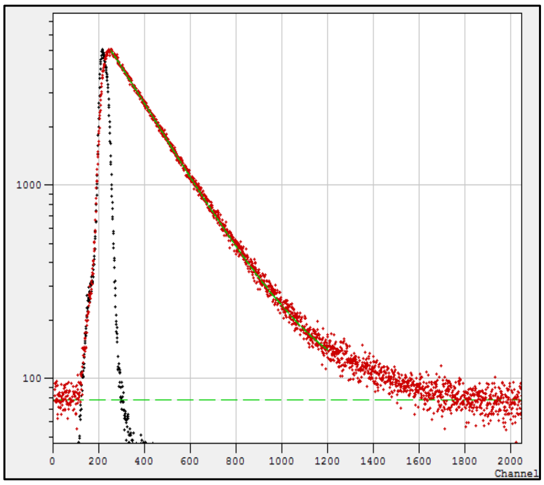

Figure S185. Fluorescence decay lifetime of AR83s.

Water acceptance test:

The solvent used was acetone and the concentration of the compound was 20 μM.

Figure S186. Water acceptance test, AR83s, acetone under visible (up) and UV (down) light.

	R 5% 10% 20% 30% 40% 50% 60% 70% 80% 90%
**Visible light**	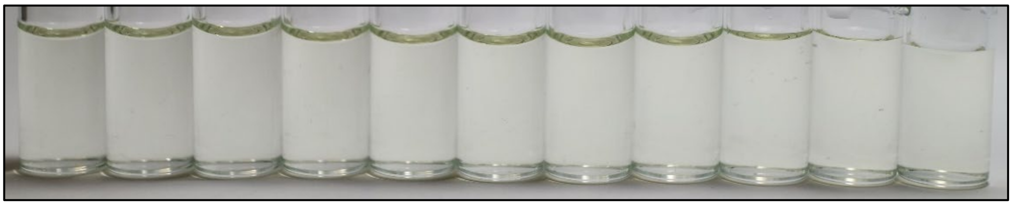
**UV light (366 nm)**	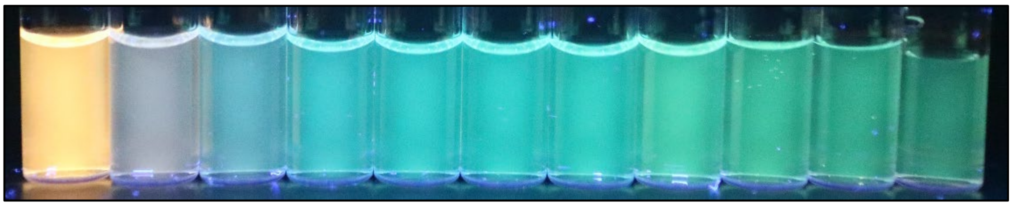

There was a slight increase in emission intensity from 70% water (AIE) using both acetone and THF as solvent. At low amounts of water, the emission maximum was in the red region (about 580 nm) while, between 30% and 60% water, it was shifted to the blue region (480 – 490 nm). At high percentages of water, from 70%, the emission maximum was shifted to the green region (530 – 560 nm).

Figure S187. Water acceptance test of AR83s in THF under visible (up) and UV (down) light.

	R 5% 10% 20% 30% 40% 50% 60% 70% 80% 90%
**Visible light**	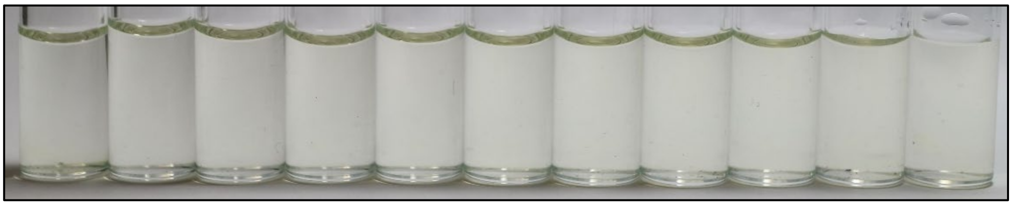

**UV light (366 nm)**	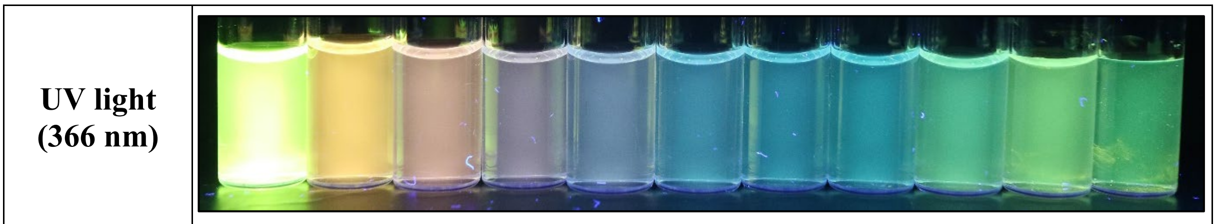

Figure S188. Emission intensity graph (left) and graph of relationship between the emission intensity and the wavelength of the maximum of emission (right) as a function of the amount of water added for AR83s using THF as a solvent.

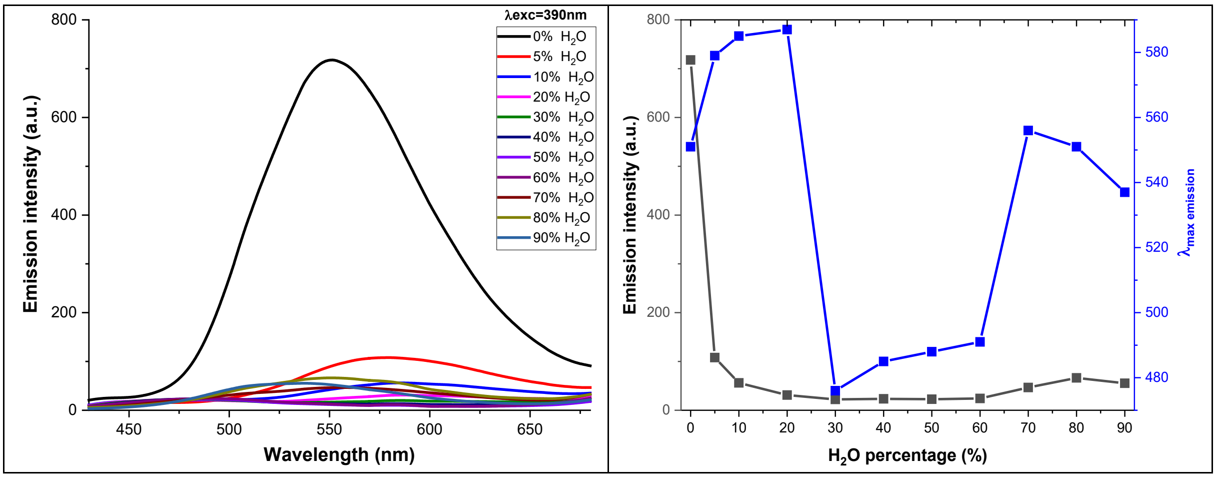

pH test:

The amount of water that the compound could accept was 20%. The concentration of the compound was 20 μM and the solvent used was acetone.

Figure S189. pH test of AR83s under visible (up) and UV (down) light.

	R HCl 3.4 4.8 6.4 7.4 7.9 10.5 KOH
**Visible light**	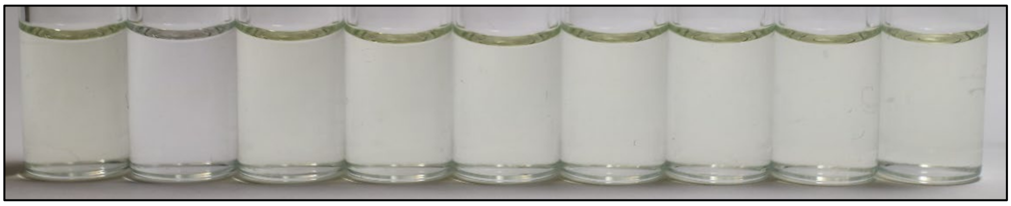
**UV light (366 nm)**	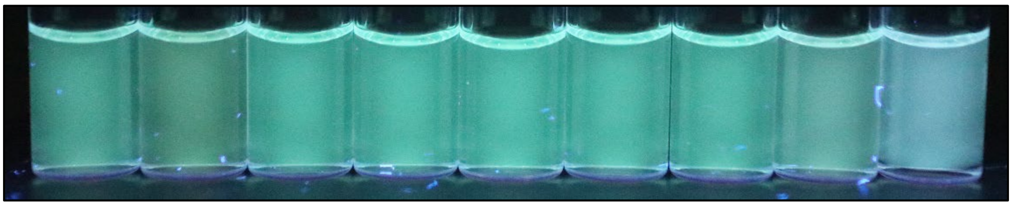

No significant changes were observed.

Cations and anions test:

The concentration of the compound was 20 μM and the solvent used was acetone.

Figure S190. Cations test of AR83s under visible (up) and UV (down) light.

	R Ag^+ Ni^{2+} Sn^{2+} Cd^{2+} Zn^{2+} Pb^{2+} Cu^{2+} Fe^{3+} Sc^{3+} Al^{3+} Hg^{2+} Au^{3+} Co^{2+} Pd^{2+} Ir^{3+} Cu^+ Ru^{3+} Pt^{2+}
**Visible light**	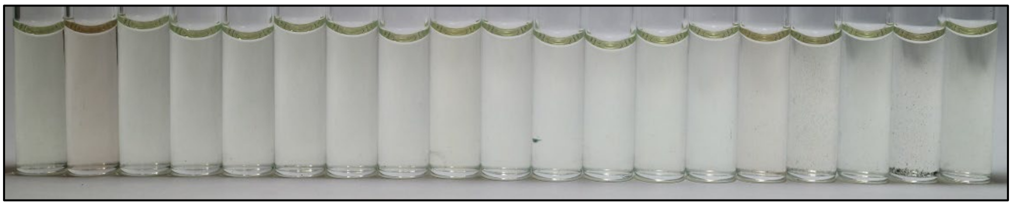
**UV light (366 nm)**	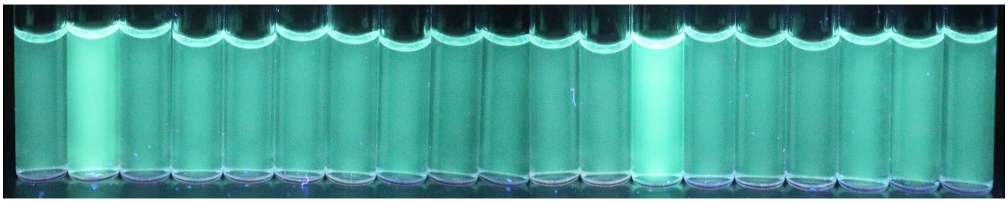

The fluorescence increased with Ag^+ and Au^{3+}. Concentration, 20 μM, solvent, acetone.

Figure S191. Anions test of AR83s under visible (up) and UV (down) light.

	R F^- Cl^- Br^- I^- BzO^- NO_3^- $H_2PO_4^-$ HSO_4^- AcO^- CN^- SCN^-
**Visible light**	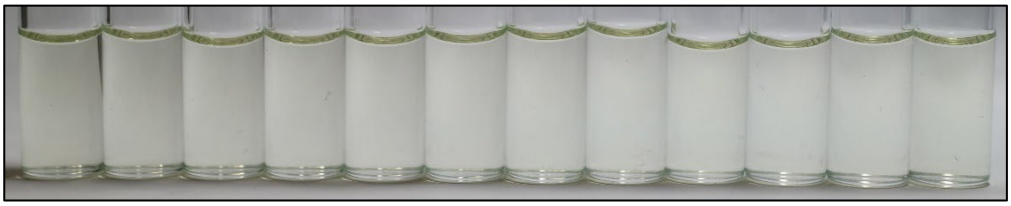
**UV light (366 nm)**	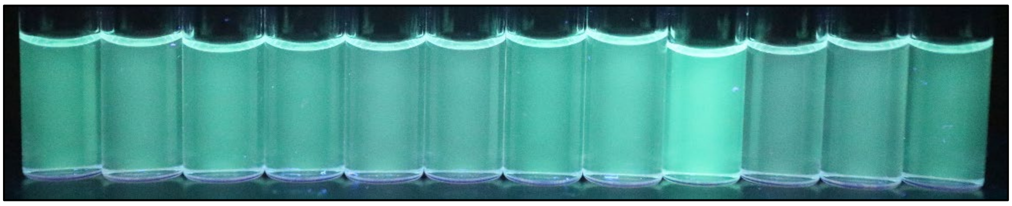

Oxidants and reductants test:

The solvent used was a mixture of MeOH-water and concentration of compound was 20 μM.

Figure S192. Oxidants and reductants test of AR83p under visible (up) and UV (down) light.

	R HCl* HNO_3 Oxn Hz H_2O_2 R m-CPBA TNB TNT R m-CPBA TATP HMTD
**Visible light**	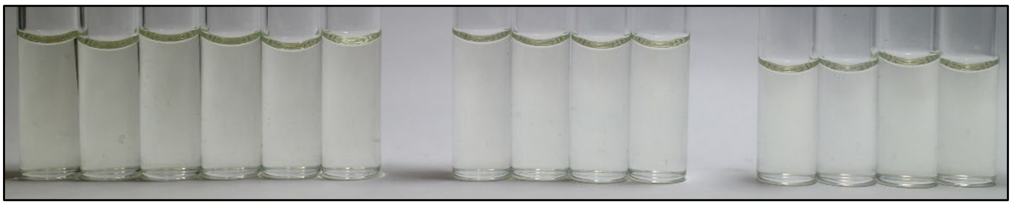
**UV light (366 nm)**	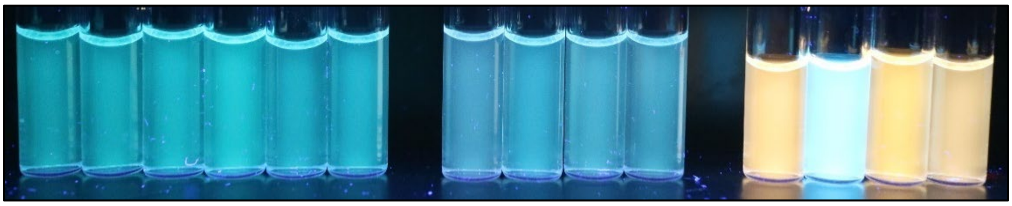

*HCl is included in all experiments in a close position to HNO_3 to distinguish between the redox action of HNO_3 and a possible effect due only to the concomitant acidity of nitric acid.

The most important change was the change in fluorescence from orange to blue with m-CPBA.

Synthesis of *N*-(piperidin-4-yl)-4-[2-(piperazin-1-yl)pyridin-5-yl]naphthalene-1,8-dicarboxylmonoimide (AR83d)

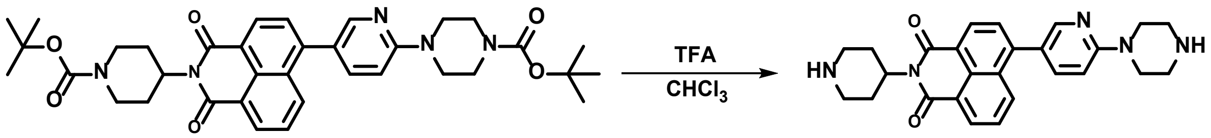

150 mg of *N*-(*N'*-Boc-piperidin-4-yl)-4-[2-(4-Boc-piperazin-1-yl)pyridin-5-yl]naphthalene-1,8-dicarboxylmonoimide (AR83p, 0.23 mmol) were dissolved in 15 ml of chloroform and, then, 2 ml of trifluoroacetic acid (26 mmol) were added. The mixture was stirred 4 hours at room temperature. Afterwards, 10 ml of water were poured into the flask and the mixture was neutralized employing a 40% sodium hydroxide solution until it reached a neutral pH. Then, 20 ml of water were added into the flask and the mixture was extracted with chloroform (3×30 ml). The organic extracts were combined, dried over anhydrous Na_2SO_4 and the solvent was evaporated under reduce pressure, to obtain 100 mg of AR83d, yellow solid, 99% yield. m.p.: 274 – 277 ºC. IR (ATR, cm^{-1}): 3236 (N-H), 2925 – 2737, 1693 (C=O), 1650 (C=O), 1579, 1495, 1348, 1235, 1151 (C-N), 1105, 782, 755, 459. ^{1}H-NMR (300 MHz, $CDCl_3$) δ (ppm): 8.60 (d, *J* = 7.6 Hz, 2H, 2×H_{Ar}), 8.34 – 8.29 (m, 2H, 2×H_{Ar}), 7.73 – 7.63 (m, 3H, 3×H_{Ar}), 6.81 (d, *J* = 8.8 Hz, 1H, H_{Ar}), 5.25 – 5.16 (m, 1H, CH), 3.65 (m, 4H, 2×CH_2), 3.27 (m, 2H, CH_2), 3.04 (m, 4H, 2×CH_2), 2.86 – 2.65 (m, 4H, 2×CH_2), 2.01 (s, 2H, 2×NH), 1.72 (m, 2H, CH_2). ^{13}C-NMR (75 MHz, $CDCl_3$) δ (ppm): 164.7 (C=O), 164.6 (C=O), 159.4 (C_{Ar}), 148.8 (C_{Ar}H), 143.9 (C_{Ar}), 138.9 (C_{Ar}H), 132.2 (C_{Ar}H), 131.3 (C_{Ar}H), 131.1 (C_{Ar}H), 130.1 (C_{Ar}), 129.0 (C_{Ar}), 127.7 (C_{Ar}H), 126.9 (C_{Ar}H), 123.6 (C_{Ar}), 123.5 (C_{Ar}), 121.9 (C_{Ar}), 106.5 (C_{Ar}H), 52.1 (CH), 47.0 (CH_2), 46.3 (CH_2), 46.1 (CH_2), 30.2 (CH_2). HRMS (MALDI, DCTB+) m/z: calculated for $C_{26}H_{27}N_5O_2$: 442.2238 (M^+ + H); found 442.2233. UV-Vis ($CHCl_3$), λ_{max} nm (log ε): 350 (3.3). Ø (MeOH, %): <3. τ (350 nm, MeOH, ns): 1.991/4.406 (χ^2: 1.116).

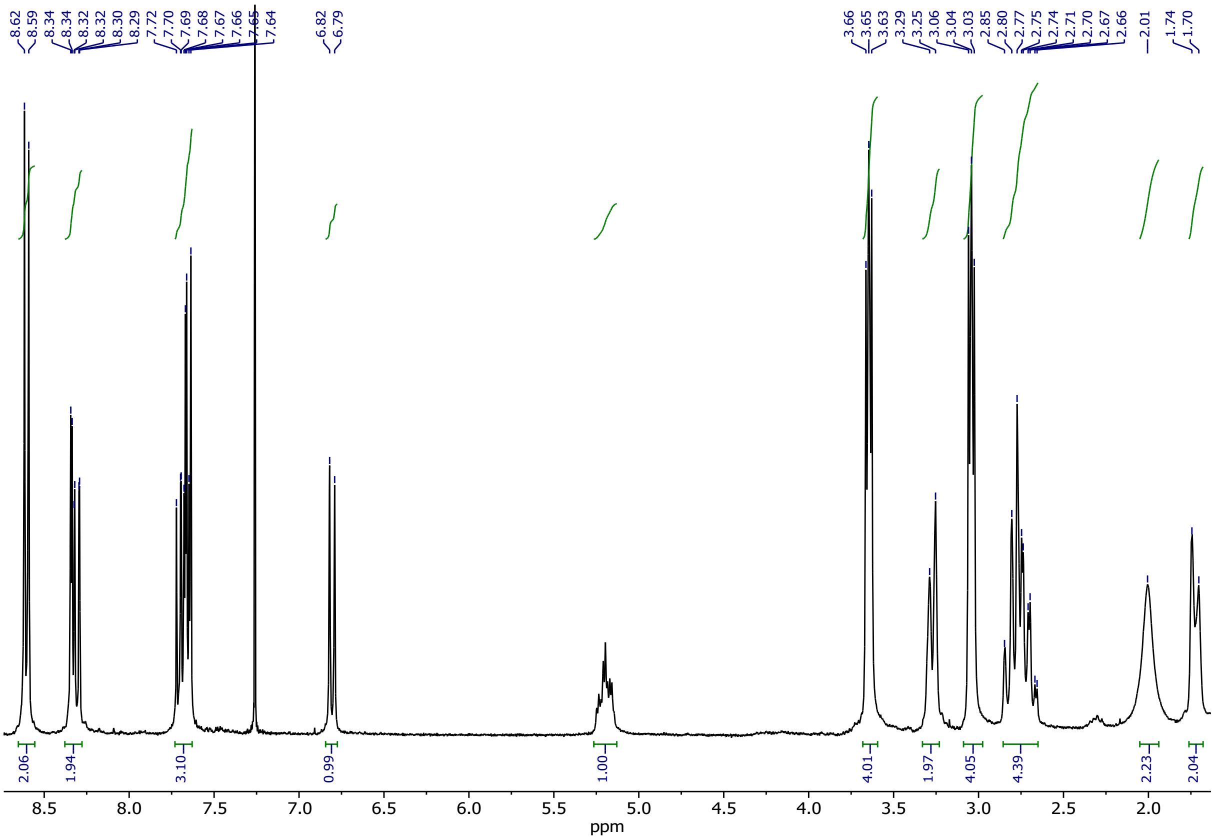

Figure S193. ^{1}H-NMR (CDCl$_3$, 300 MHz) of AR83d.

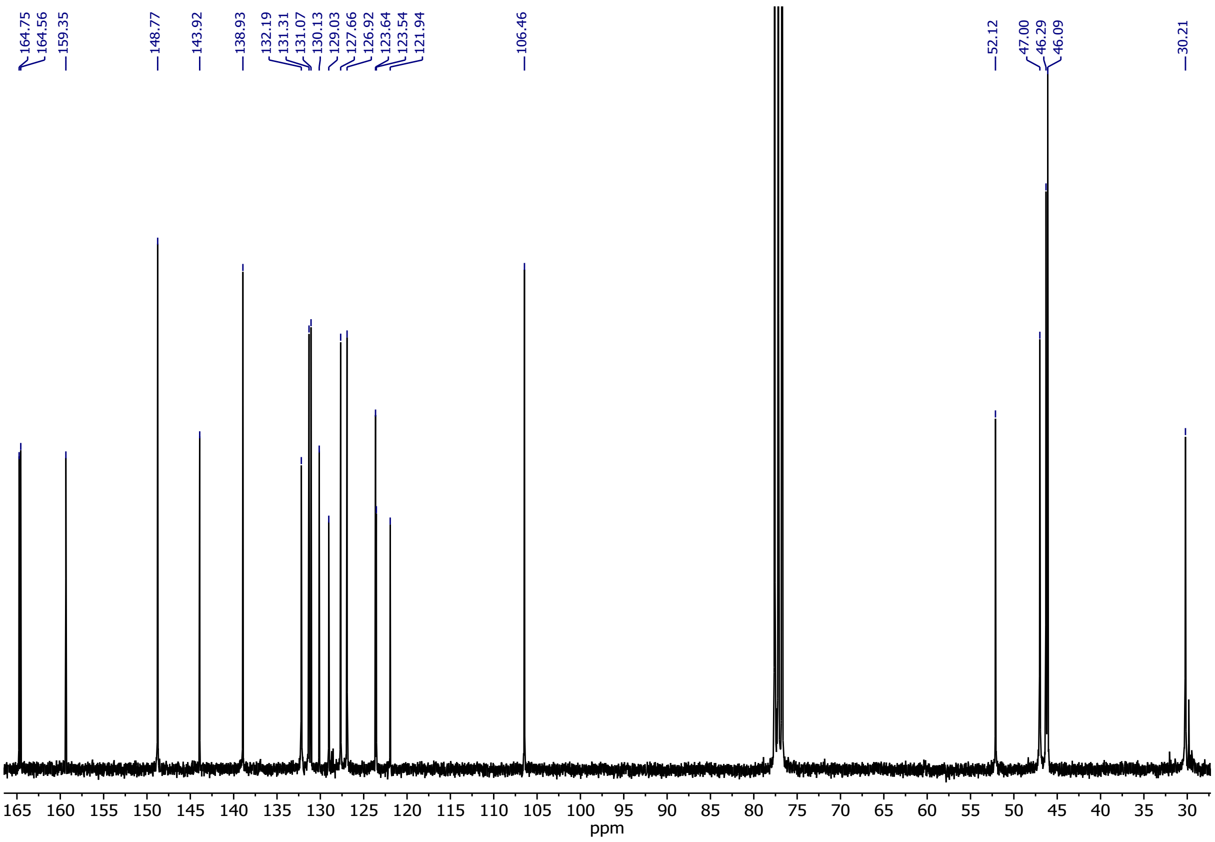

Figure S194. ^{13}C-NMR (CDCl$_3$, 75 MHz) of AR83d.

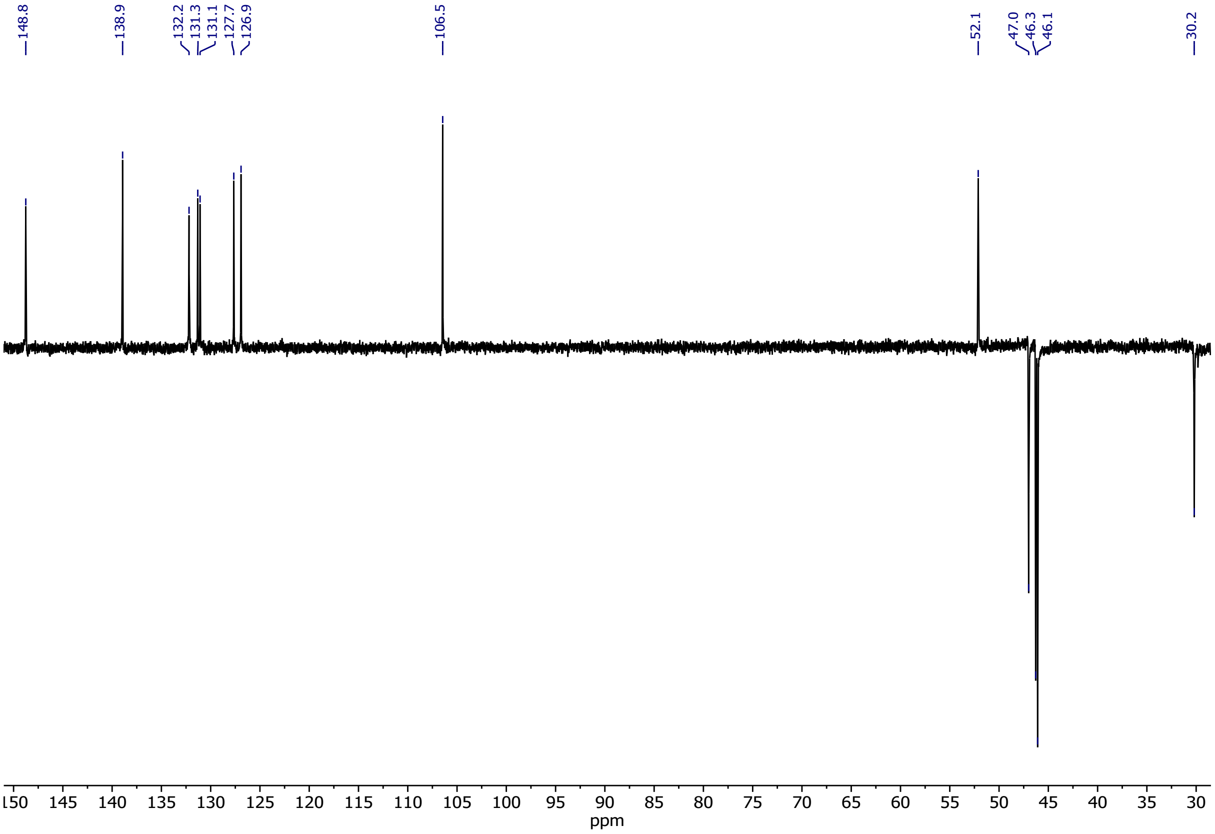

Figure S195. DEPT NMR ($CDCl_3$, 75 MHz) of AR83d.

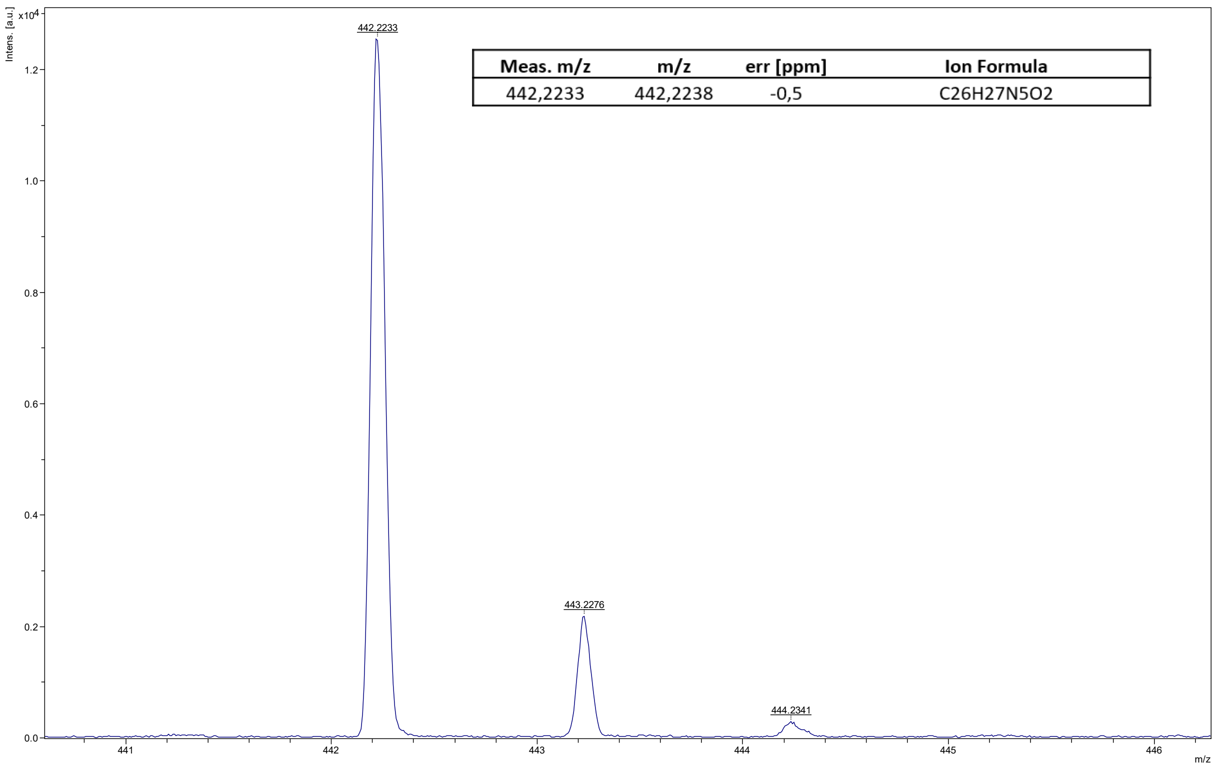

Figure S196. HRMS (MALDI, DCTB+) of AR83d.

Solvatochromism:

The concentration of the compound was 20 µM and the excitation wavelength was 350 nm.

Figure S197. Solvatochromism of AR83d.

**Absorbance**	**Normalized absorbance**
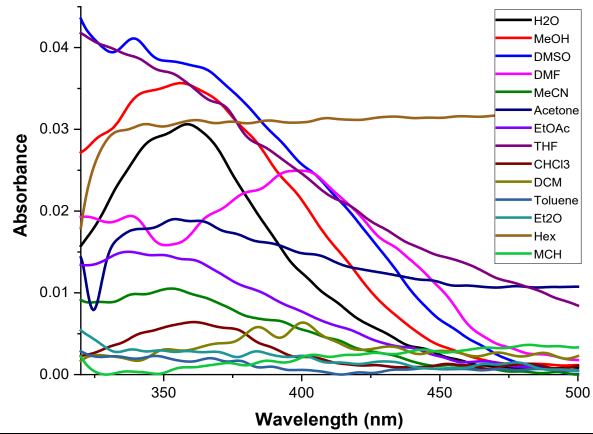	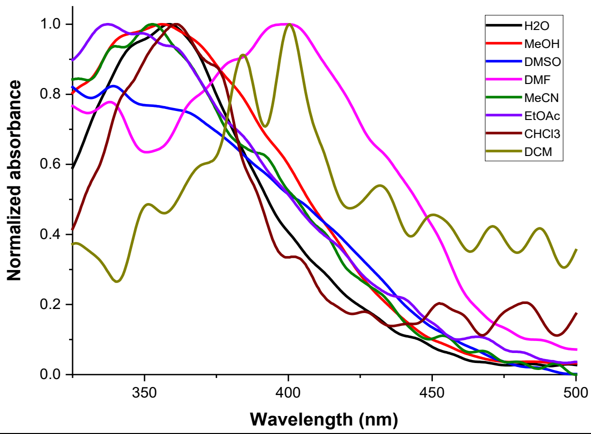
**Emission**	**Normalized emission**
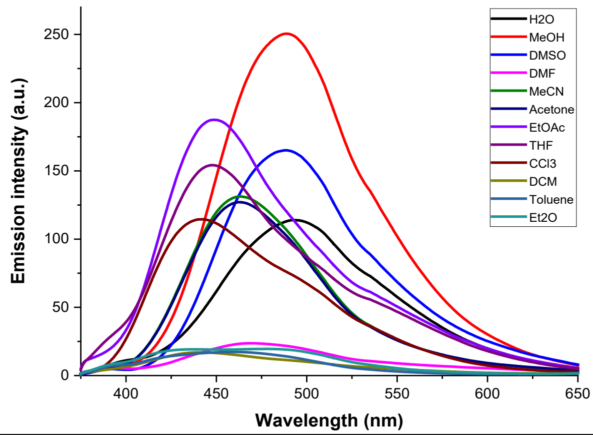	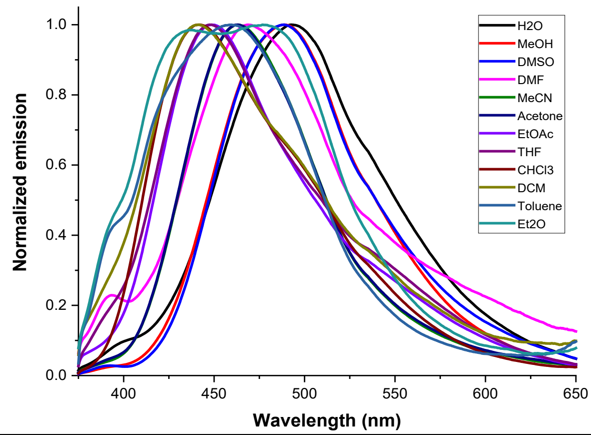

Figure S198. Solvatochromism of AR83d under visible and 366 nm light

	**H_2O MeOH DMSO DMF MeCN Acet. EtOAc THF $CHCl_3$ DCM Tol. Et_2O Hex MCH**
**Visible light**	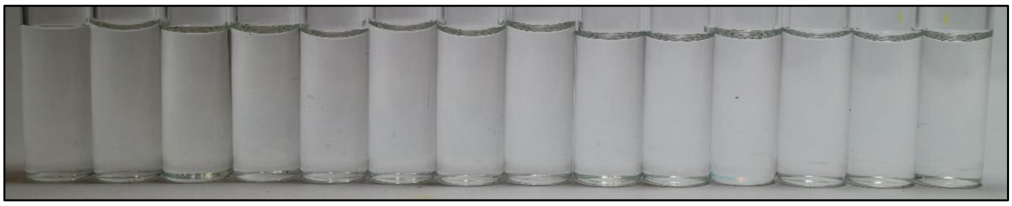
**UV light (366 nm)**	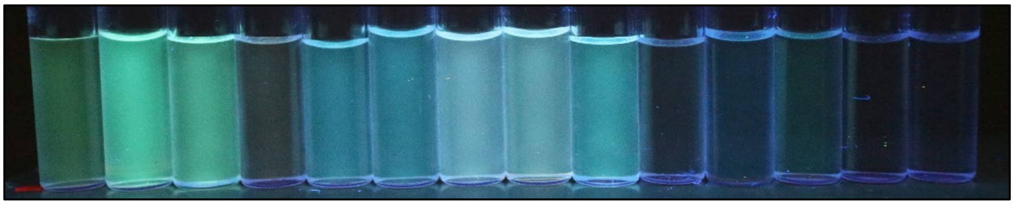

Fluorescence decay lifetime (τ):

The solvent used was MeOH and the concentration of the compound was 20 µM.

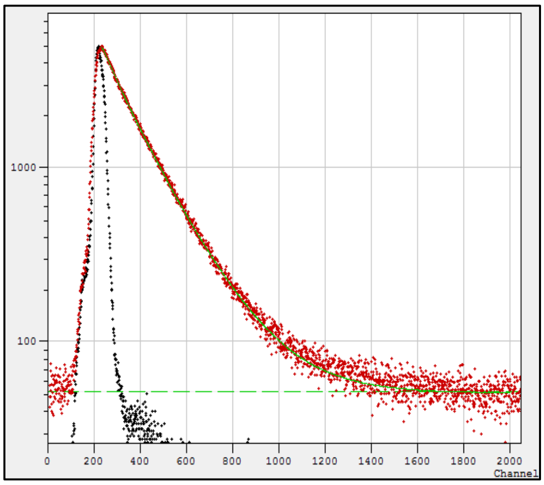

Figure S199. Fluorescence decay lifetime of AR83d.

Water acceptance test:

The solvent used was MeOH and the concentration of the compound was 20 µM.

Figure S200. Water acceptance test of AR83d under visible (up) and UV (down) light.

	R 5% 10% 20% 30% 40% 50% 60% 70% 80% 90%
**Visible light**	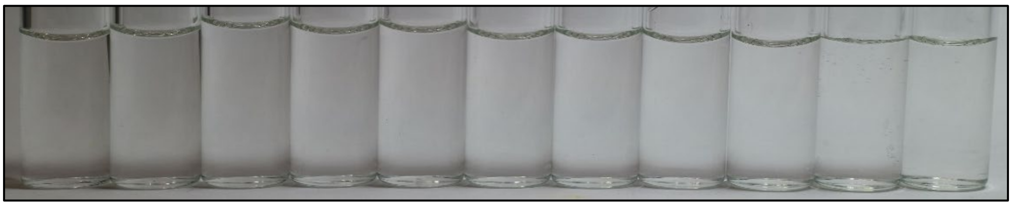
**UV light (366 nm)**	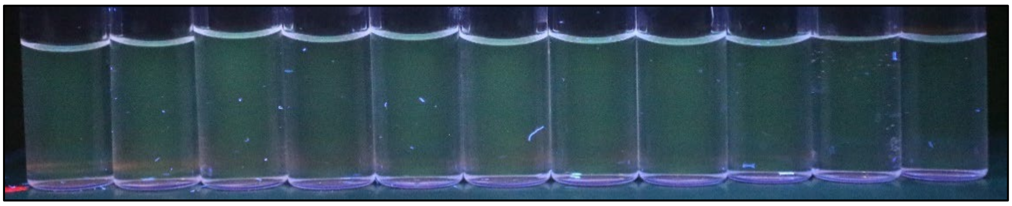

The fluorescent emission was quenched as the amount of water increased (ACQ).

pH test:

The amount of water for the performance of this test was 90%. The concentration of the compound was 20 µM and the solvent used was MeOH.

Figure S201. pH test of AR83d under visible (up) and UV (down) light.

	R HCl 3.4 4.8 6.4 7.4 7.9 10.5 KOH
**Visible light**	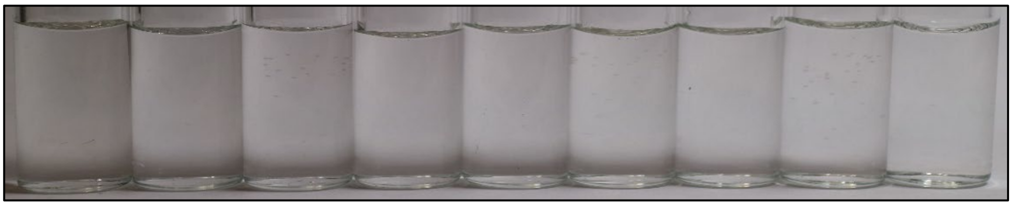

**UV light (366 nm)**	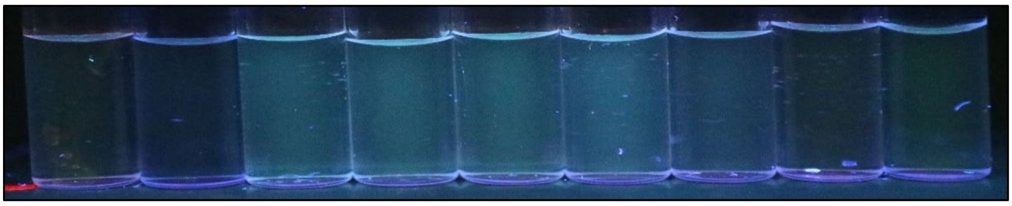

There was a slight increase in the fluorescence intensity at intermediate pHs.

Cations and anions test:

The concentration of the compound was 20 µM and the solvent used was a mixture of MeOH and water (90%).

Figure S202. Cations test of AR83d under visible (up) and UV (down) light.

	**R Ag^{+} Ni^{2+} Sn^{2+} Cd^{2+} Zn^{2+} Pb^{2+} Cu^{2+} Fe^{3+} Sc^{3+} Al^{3+} Hg^{2+} Au^{3+} Co^{2+} Pd^{2+} Ir^{3+} Cu^{+} Ru^{3+} Pt^{2+}**
**Visible light**	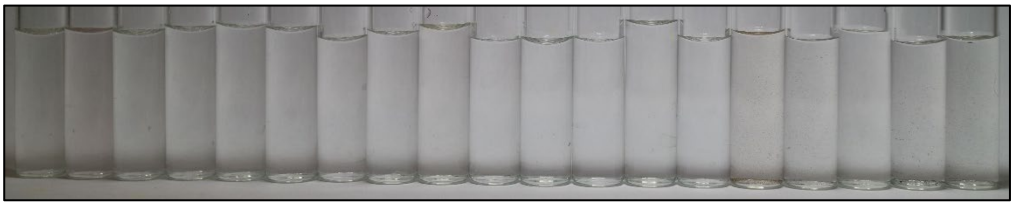
**UV light (366 nm)**	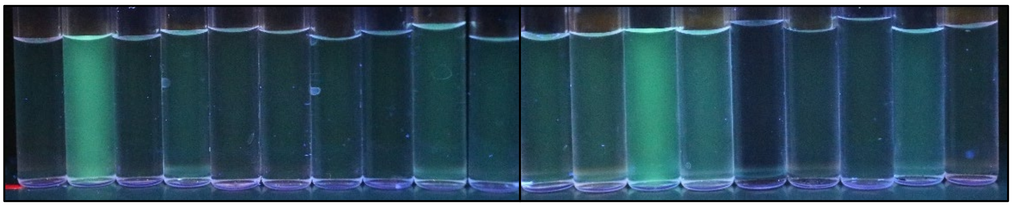

The most significant changes under UV light were the increase in the fluorescence emission when the probe was exposed to Ag^{+} and Au^{3+}.

The concentration of the compound was 20 µM and the solvent used was a mixture of MeOH and water (90%).

Figure S203. Anions test of AR83d under visible (up) and UV (down) light.

	**R F^{-} Cl^{-} Br^{-} I^{-} BzO^{-} NO_3^{-} $H_2PO_4^{-}$ HSO_4^{-} AcO^{-} CN^{-} SCN^{-}**
**Visible light**	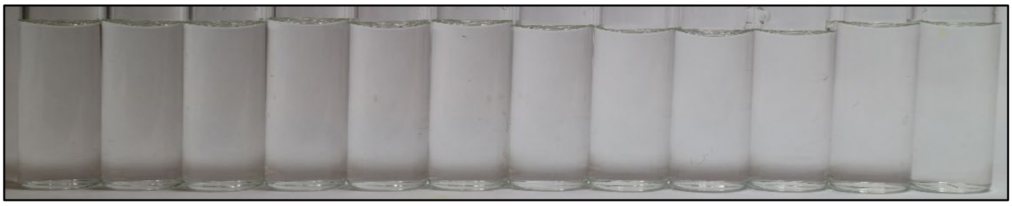
**UV light (366 nm)**	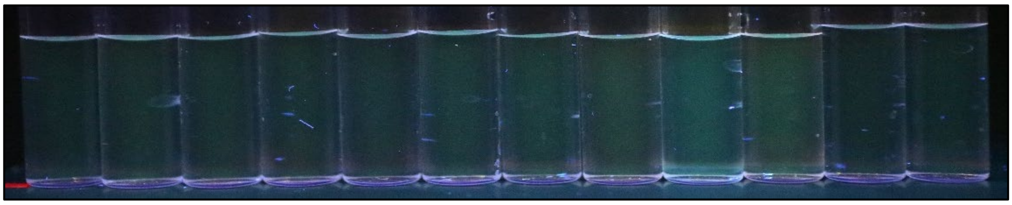

No significant changes were observed.

Oxidants and reductants test:

The solvent used was a mixture of MeOH and water (90%) and concentration of the compound was 20 µM.

Figure S204. Oxidants and reductants test of AR83d under visible (up) and UV (down) light.

	R HCl* HNO_3 m-CPBA Oxn Hz H_2O_2 TNB TNT TATP HMTD
**Visible light**	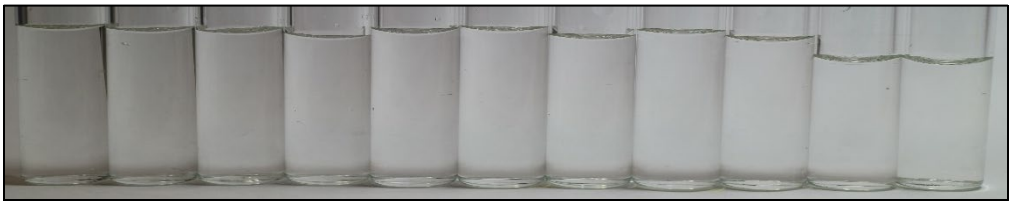
**UV light (366 nm)**	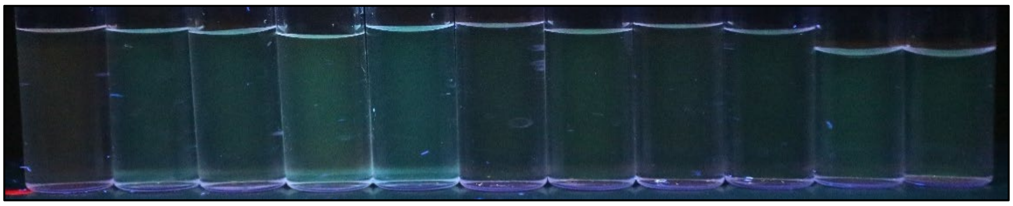

*HCl is included in all experiments in a close position to HNO_3 to distinguish between the redox action of HNO_3 and a possible effect due only to the concomitant acidity of nitric acid. No significant changes were observed.

Preliminary solvents test:

In order to determine the fluorescence variation of the compound under study in the presence of TATP, some test were carried out for the probe in 9 different solvents. The AR83d concentration was 25 µM and the TATP was added in excess (7 mg in each vial). All the tests were performed at room temperature and the photographs were taken immediately after the addition of TATP and again after 24 hours.

Figure S205. Pairs of AR83d (left) and AR83d with excess of TATP (right) in different solvents at time 0 h (up) and 24 h (down).

	DMF MeCN EtOAc THF $CHCl_3$ DCM Tol $CHCl_3$:MeOH DCM:MeOH
**t = 0 h**	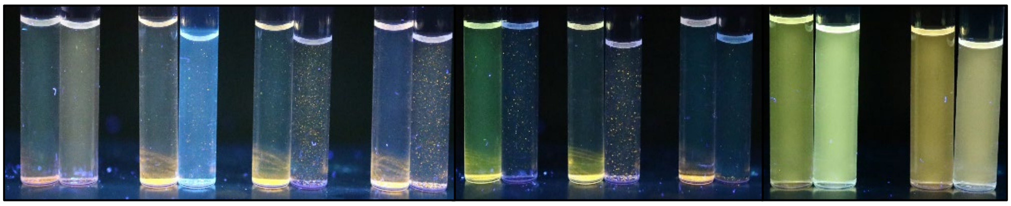
**t = 24 h**	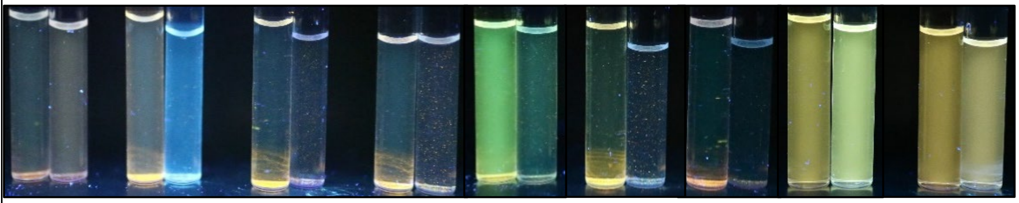

The most noticeable fluorescence changes were seen in DMF, MeCN, $CHCl_3$:MeOH (9:1) and DCM:MeOH (9:1).

Solvents test with TATP:

For all solvents, the working concentration of AR83d was 2.5 μM, the excitation wavelength was 350 nm and the temperature for all tests was 25ºC.

In the kinetic studies, TATP was in excess (20 mM) and the fluorescence emission measurements were made during 40 minutes, at 5 minutes time intervals. In the titration, the TATP concentration was between 0 and 35000 μM and it was added directly as solid. The measurements were carried out immediately after the addition of the probe.

Figure S206. Study of AR83d with TATP in DMF. Kinetic study (up left) and profile as function as time at 390 nm, 460 nm and 550 nm simultaneously (up right) in presence of TATP excess. Titration (down left) and fluorescence profile at 390 nm, 465 nm and 585 nm simultaneously (down right) under increasing concentrations of TATP.

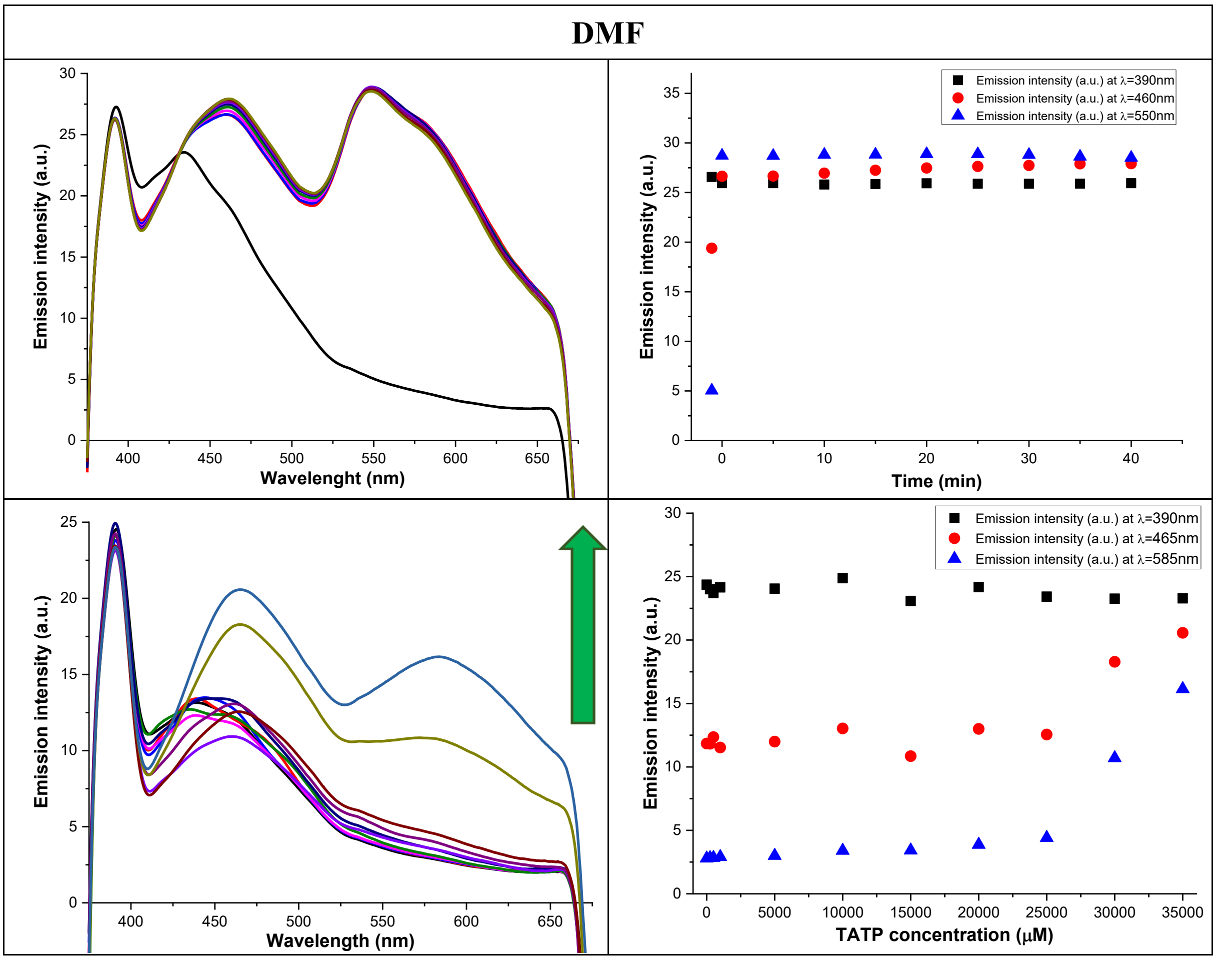

There was an increase in the emission intensity band immediately after adding TATP. The LOD of TATP was somewhere between 25000 and 30000 μM with the appearance of a new maximum peak.

Figure S207. Kinetic study at different times under visible (up) and UV (down) light in DMF. In each photo, left vial was contained the probe and right vial was contained the probe and an excess of TATP.

	**t=0**	**t=60 min**	**t=48 h**
**Visible light**	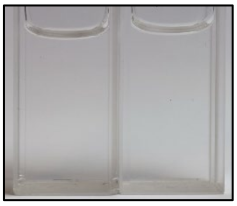	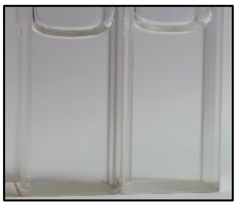	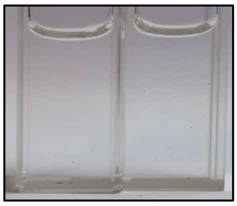
**UV light (366 nm)**	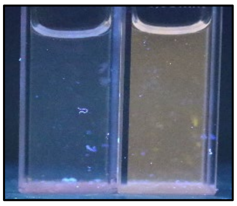	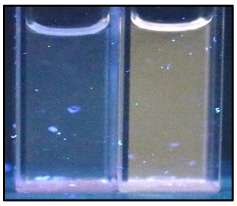	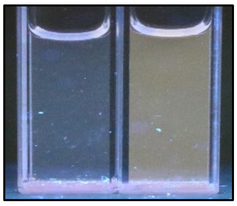

Figure S208. Titration of AR83d with an excess of TATP under visible (up) and UV (down) light in DCM.

µM	**0 250 500 1000 5000 10000 15000 20000 25000 30000 35000**
**Visible light**	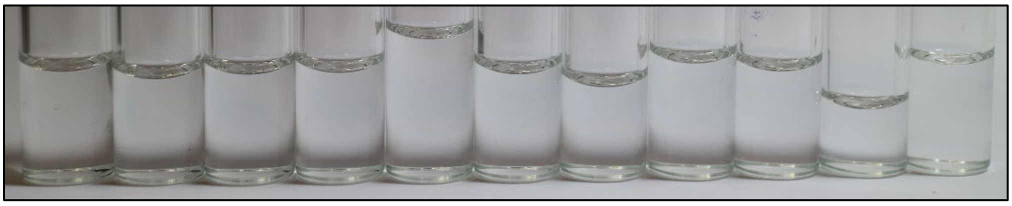
**UV light (366 nm)**	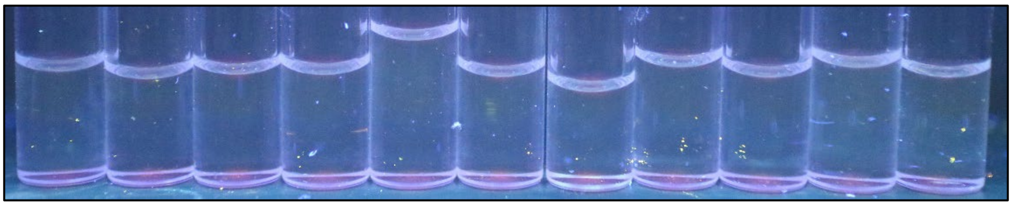

Figure S209. Study of AR83d with TATP in MeCN. Kinetic study (up left) and profile as function as time at 390 nm and 450 nm simultaneously (up right) in presence of TATP excess. Titration (down left) and fluorescence profile at 390 nm, 450 nm and 570 nm simultaneusly (down right) under increasing concentrations of TATP.

MeCN

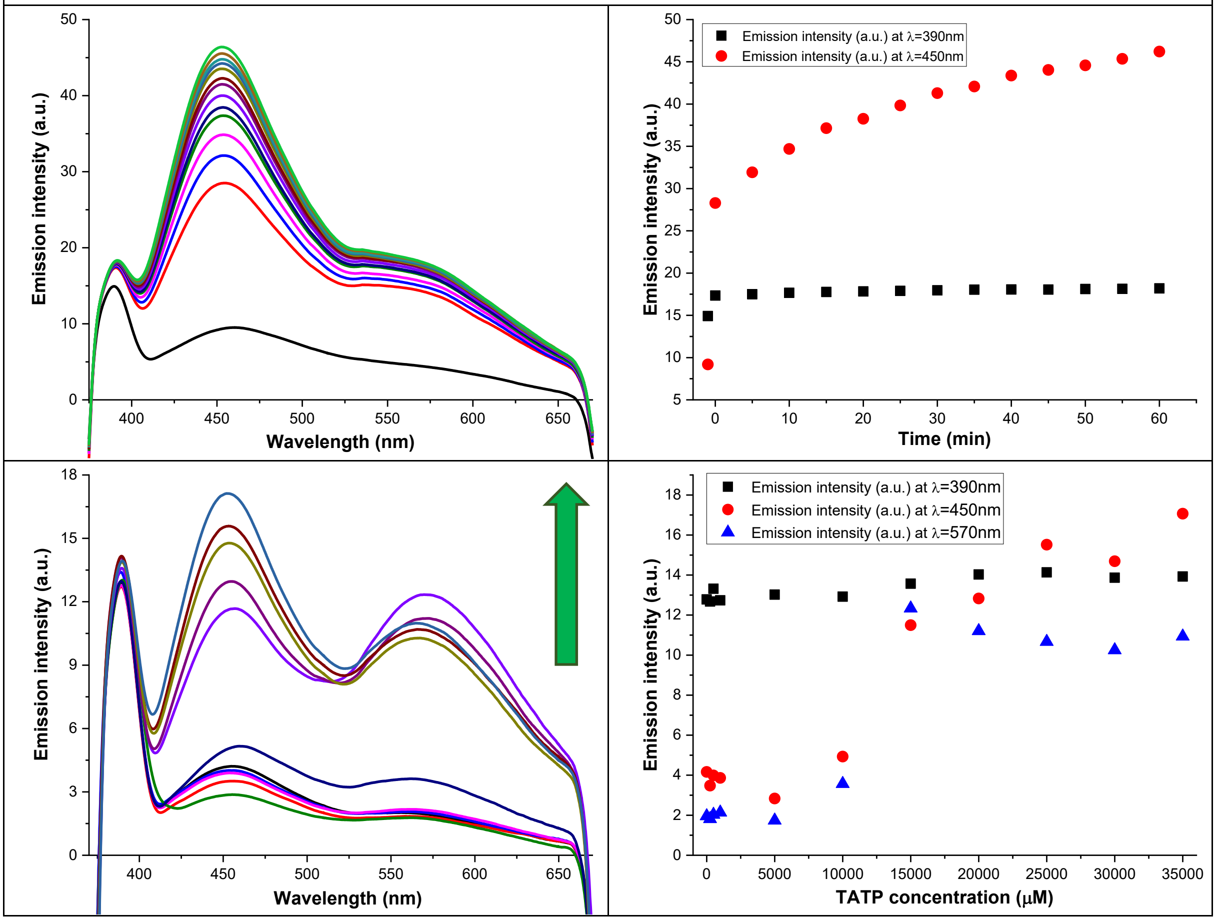

There was a increase change in the emission intensity band immediately after adding TATP. According to the spectra, the LOD of TATP was somewhere between 10000 and 15000 μM, a fairly high limit, with the appearance of a new emission peak.

Figure S210. Kinetic study at different times under visible (up) and UV (down) light in MeCN. In each photo, left vial contained probe and right vial contained probe and excess TATP.

	**t=0**	**t=60 min**	**t=24 h**
**Visible light**	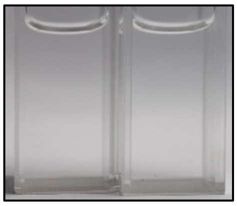	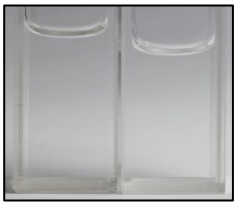	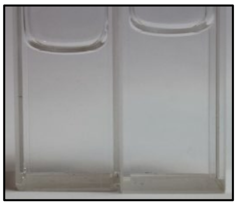
**UV light (366 nm)**	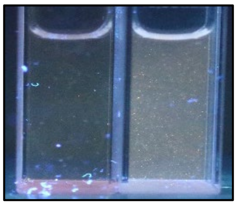	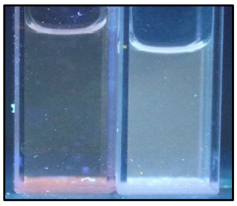	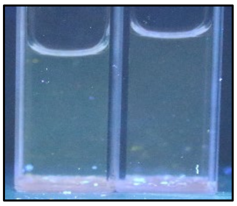

Figure S211. Titration of AR83d with an excess of TATP under visible (up) and UV (down) light in MeCN.

µM	0 250 500 1000 5000 10000 15000 20000 25000 30000 35000
**Visible light**	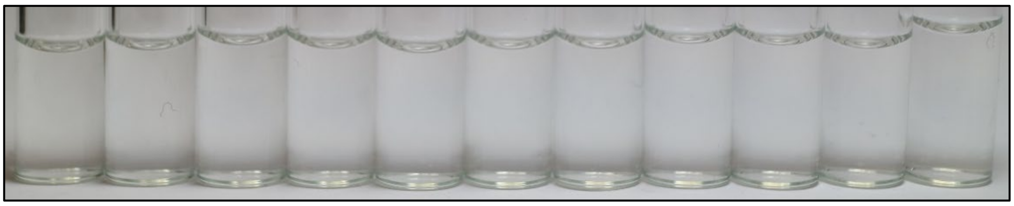
**UV light (366 nm)**	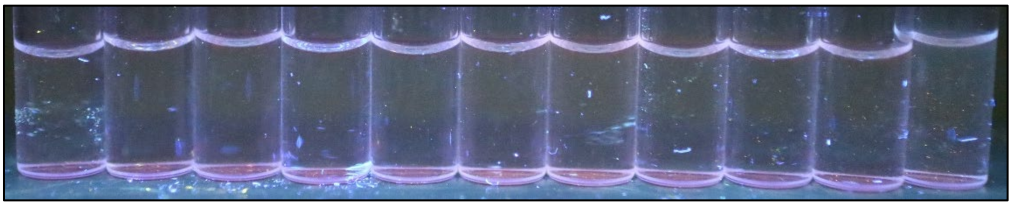

Figure S212. Study of AR83d with TATP in $CHCl_3$. Kinetic study (up left) and profile as function as time at 455 nm and 545 nm simultaneously (up right) in presence of TATP excess. Titration (down left) and fluorescence profile at 465 nm and 540 nm simultaneusly (down right) under increasing concentrations of TATP.

**$CHCl_3$**	
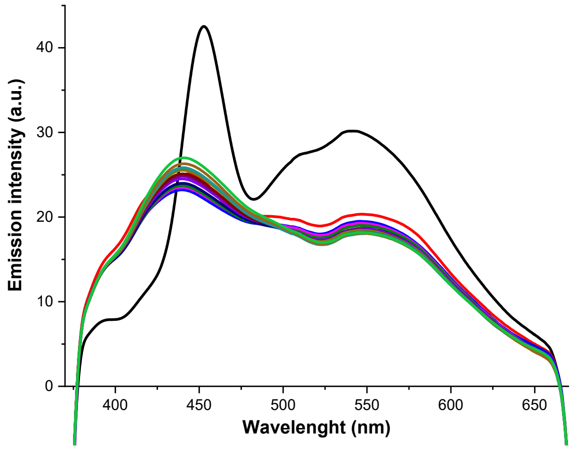	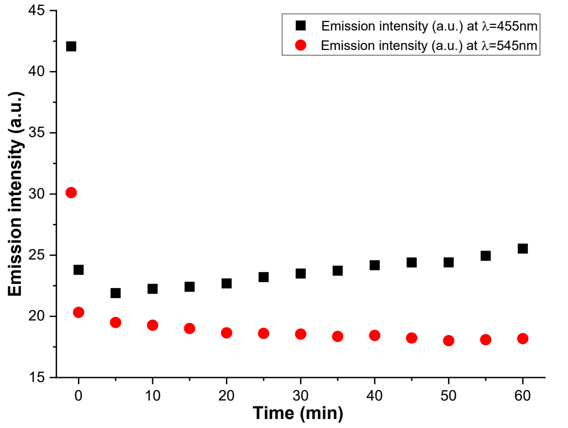
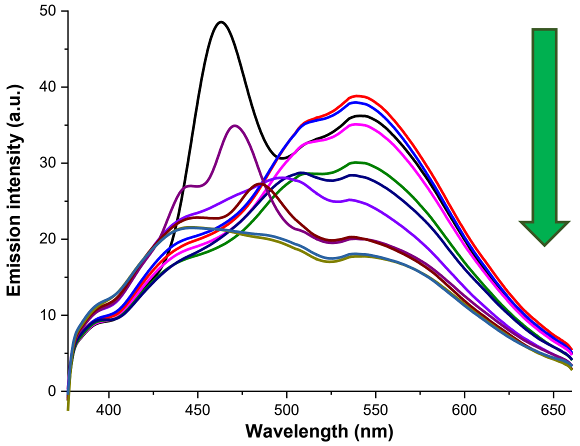	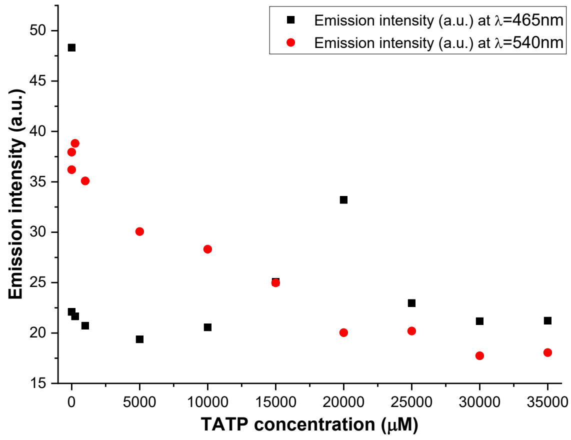

There was a decrease change in the emission intensity band immediately after adding TATP. The LOD of TATP was somewhere between 1000 and 5000 µM with the dissappearance of a maximum emission.

Figure S213. Kinetic study at different times under visible (up) and UV (down) light in $CHCl_3$. In each photo, left vial was contained the probe and right vial was contained the probe and an excess of TATP.

	**t=0**	**t=60 min**	**t=24 h**
**Visible light**	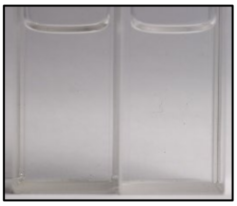	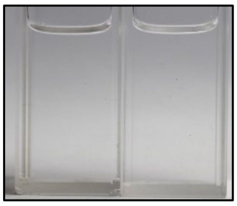	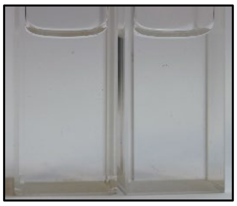
**UV light (366 nm)**	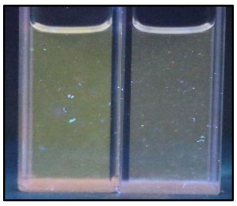	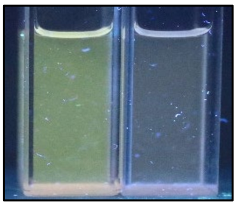	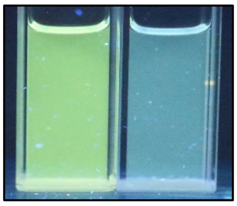

Figure S214. Titration of AR83d with an excess of TATP under visible (up) and UV (down) light in $CHCl_3$.

µM	**0 250 500 1000 5000 10000 15000 20000 25000 30000 35000**
**Visible light**	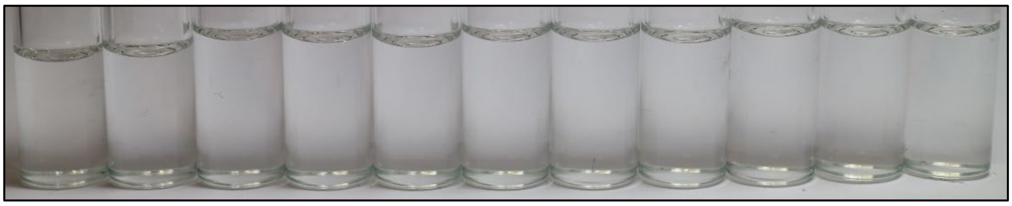
**UV light (366 nm)**	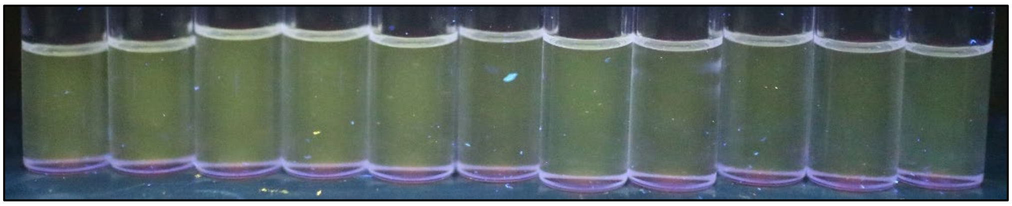

Figure S215. Study of AR83d with TATP in $CHCl_3$:MeOH (9:1). Kinetic study (up left) and profile as function as time at 450 nm and 550 nm simultaneously (up right) in presence of TATP excess. Titration (down left) and fluorescence profile at 450 nm and 550 nm simultaneously (down right) under increasing concentrations of TATP.

$CHCl_3$:MeOH (9:1)

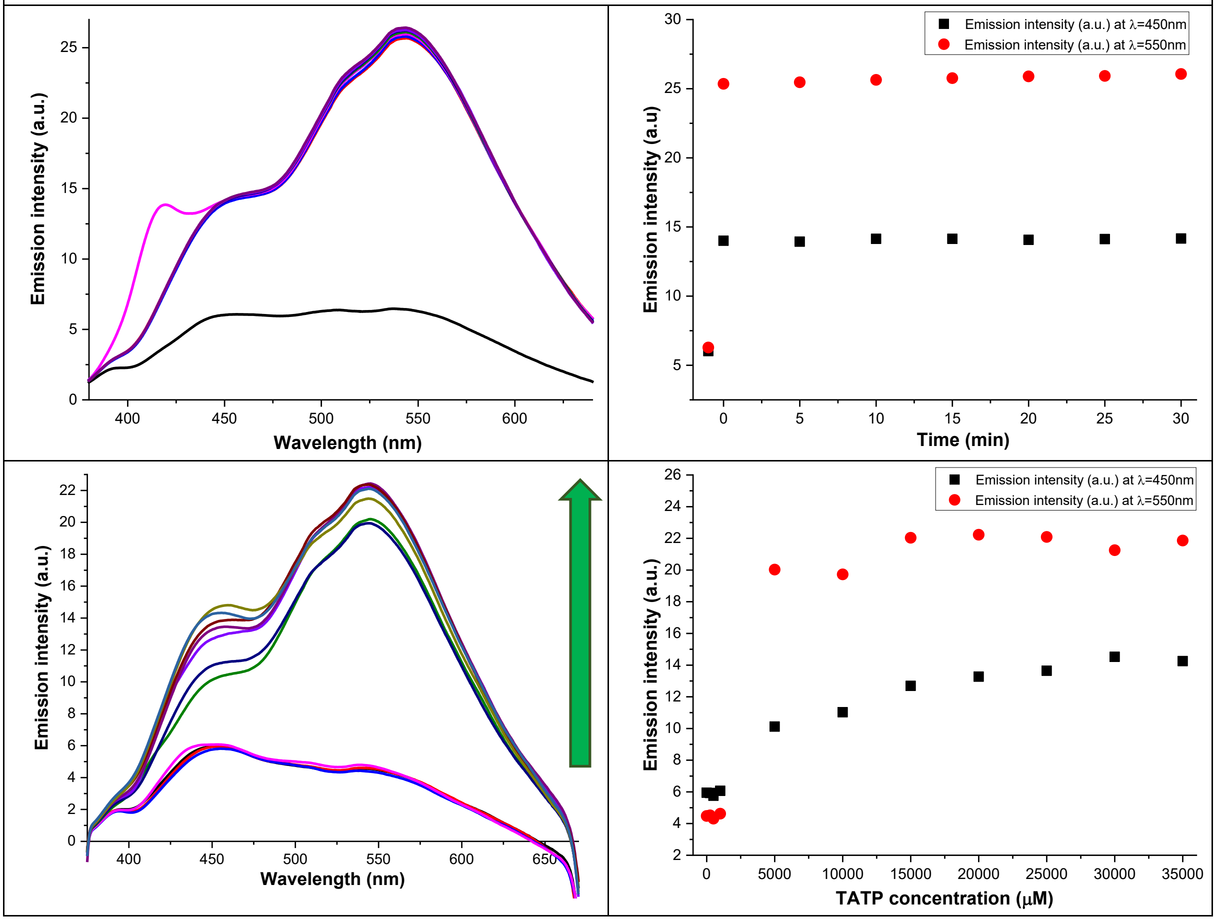

There was an increase in the emission intensity band immediately after adding TATP. According to the spectra, the LOD of TATP was somewhere between 5000 and 10000 μM.

Figure S216. Kinetic study at different times under visible (up) and UV (down) light in $CHCl_3$:MeOH (9:1). In each photo, left vial contained probe and right vial contained probe and excess TATP.

	**t=0**	**t=30 min**	**t=24 h**
**Visible light**	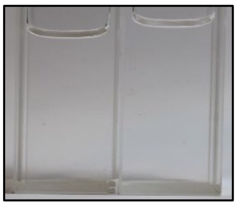	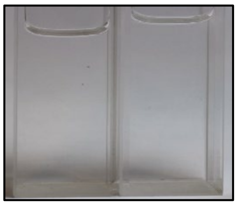	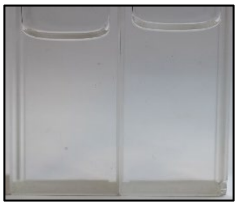
**UV light (366 nm)**	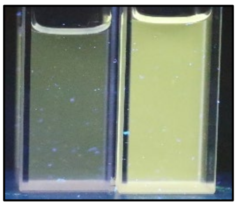	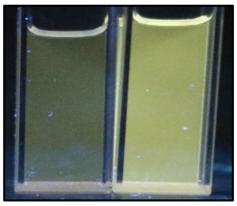	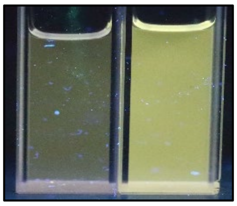

Figure S217. Titration of AR83s with an excess of TATP under visible (up) and UV (down) light in $CHCl_3$:MeOH (9:1).

µM	**0 250 500 1000 5000 10000 15000 20000 25000 30000 35000**
**Visible light**	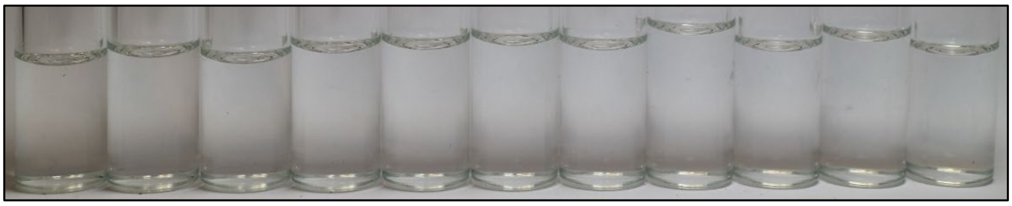
**UV light (366 nm)**	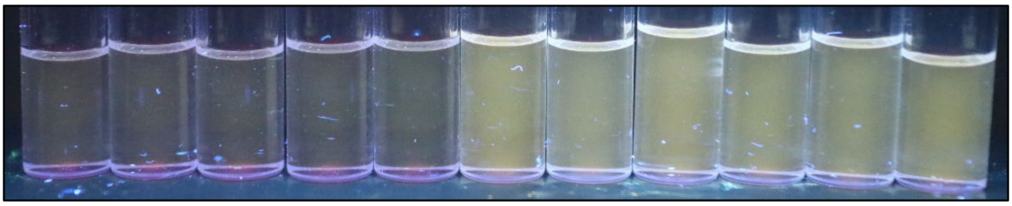

Figure S218. Study of AR83d with TATP in DCM:MeOH (9:1). Kinetic study (up left) and profile as function as time at 450 nm and 550 nm simultaneously (up right) in presence of TATP excess. Titration (down left) and fluorescence profile at 415 nm and 560 nm simultaneously (down right) under increasing concentrations of TATP.

DCM:MeOH (9:1)

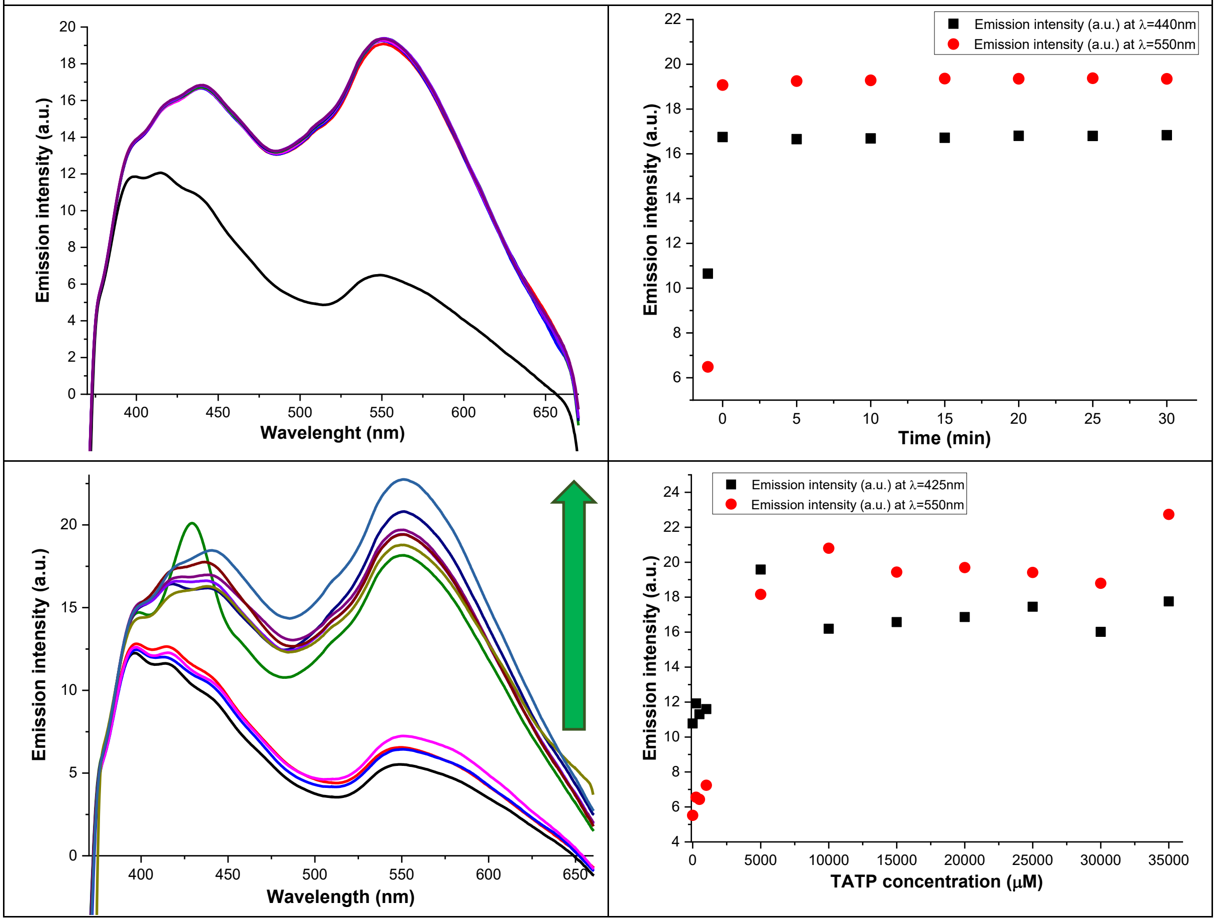

There was a increase change in the emission intensity band immediately after adding TATP. According to the fluorescence spectra, the LOD of TATP was somewhere between 1000 and 5000 µM.

Figure S219. Kinetic study at different times under visible (up) and UV (down) light in DCM:MeOH (9:1). In each photo, left vial was contained the probe and right vial was contained the probe and an excess of TATP.

	**t=0**	**t=30 min**	**t=24 h**
**Visible light**	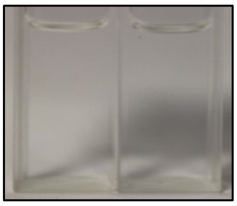	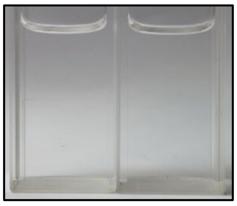	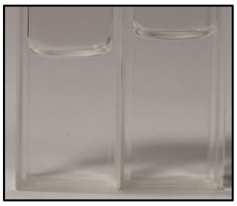
**UV light (366 nm)**	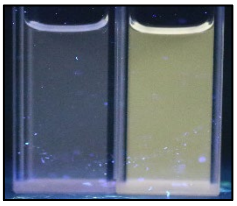	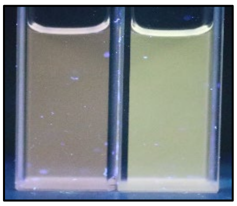	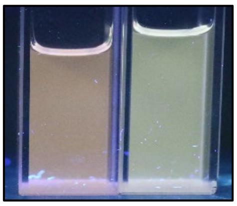

Figure S220. Titration of AR83d with an excess of TATP under visible (up) and UV (down) light in DCM:MeOH (9:1).

µM	**0 250 500 1000 5000 10000 15000 20000 25000 30000 35000**
**Visible light**	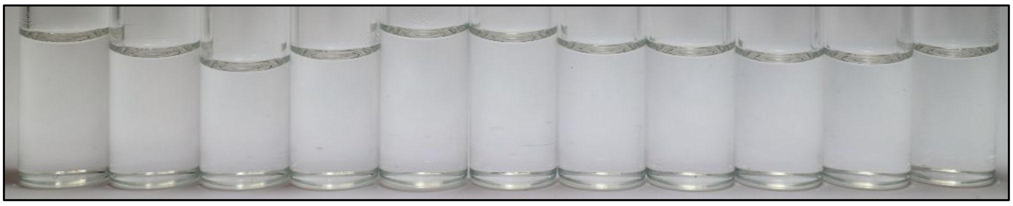
**UV light (366 nm)**	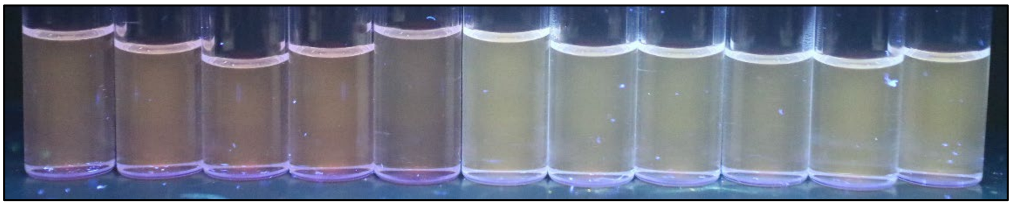

Because of the low sensitivity the study was discontinued.

Synthesis of *N*-(*N'*-Boc-piperidin-4-yl)-4-[(4-Boc-piperazin-1-yl)phenyl]naphthalene-1,8-dicarboxylmonoimide (AR90p)

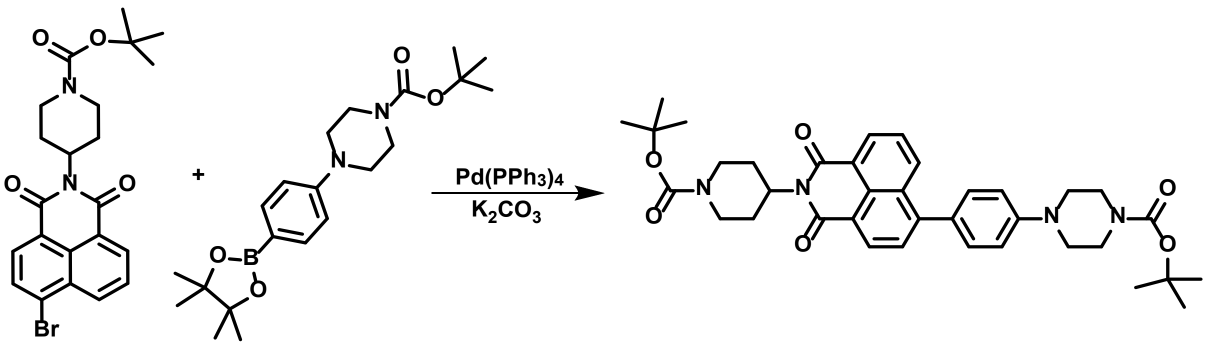

250 mg of *N*-(*N'*-Boc-piperidin-4-yl)-4-bromonaphthalene-1,8-dicarboxylmonoimide (AR43p, 0.54 mmol), 220 mg of 4-(4-Boc-piperazin-1-yl)phenylboronic acid pinacol ester (0.57 mmol) and 752 mg of potassium carbonate (5.44 mmol) were dissolved in a mixture of toluene:butanol:water (4:1:2 ml) under nitrogen atmosphere and, then, the catalyst, $Pd(PPh_3)_4$ (5% mmol) was added. The resulting mixture was stirred overnight at 110 ºC. After that, the solvent was evaporated under reduce pressure, the solid was dissolved in CH_2Cl_2 and washed with water (3×100 ml). The organic extracts were combined, dried over anhydrous Na_2SO_4 and the solvent was evaporated under reduce pressure. The organic solid was purified by column chromatography (SiO_2, CH_2Cl_2:MeOH) getting 242 mg of AR90p as a yellow solid, 70% yield. m.p.: 250 – 252ºC. IR (ATR, cm^{-1}): 3006 – 2832, 1686 (C=O), 1650 (C=O), 1582, 1507, 1420 (C-O), 1351, 1232, 1159 (C-N), 1124, 1097, 1037, 977, 915, 902, 861, 812, 780, 755, 663, 639, 531, 457. ^{1}H-NMR (300 MHz, $CDCl_3$) δ (ppm): 8.58 (d, J = 7.7 Hz, 2H, 2×H_{Ar}), 8.32 (d, J = 8.5 Hz, 1H, H_{Ar}), 7.67 (t, J = 8.0 Hz, 2H, 2×H_{Ar}), 7.42 (d, J = 8.5 Hz, 2H, 2×H_{Ar}), 7.07 (d, J = 8.5 Hz, 2H, 2×H_{Ar}), 5.20 (m, 1H, CH), 4.30 (s, 2H, CH_2), 3.64 (m, 4H, 2×CH_2), 3.27 (m, 4H, 2×CH_2), 2.94 – 2.69 (m, 4H, 2×CH_2), 1.68 (m, 2H, CH_2), 1.49 (s, 18H, 6×CH_3). ^{13}C-NMR (75MHz, $CDCl_3$) δ (ppm): 164.8 (C=O) , 164.6 (C=O), 154.8 (C=O), 154.7 (C=O), 151.2 (C_{Ar}), 146.8 (C_{Ar}), 132.8 (C_{Ar}H), 131.2 (C_{Ar}), 131.1 (C_{Ar}), 131.0 (C_{Ar}H), 130.1 (C_{Ar}), 129.0 (C_{Ar}), 127.7 (C_{Ar}H), 126.8 (C_{Ar}H), 123.3 (C_{Ar}), 121.5 (C_{Ar}), 116.1 (C_{Ar}H), 80.2 (Cq), 79.6 (Cq), 51.9 (CH), 48.9 (CH_2), 43.8 (CH_2), 28.6 (CH_3), 28.5 (CH_3), 28.4 (CH_2). HRMS (MALDI, DIT-) m/z: calculated for $C_{37}H_{44}N_4O_6$: 640.3255 (M^-); found 640.3261. Ø (MCH, %): 93.19. τ (375 nm, MCH, ns): 3.542 (χ^2: 0.975).

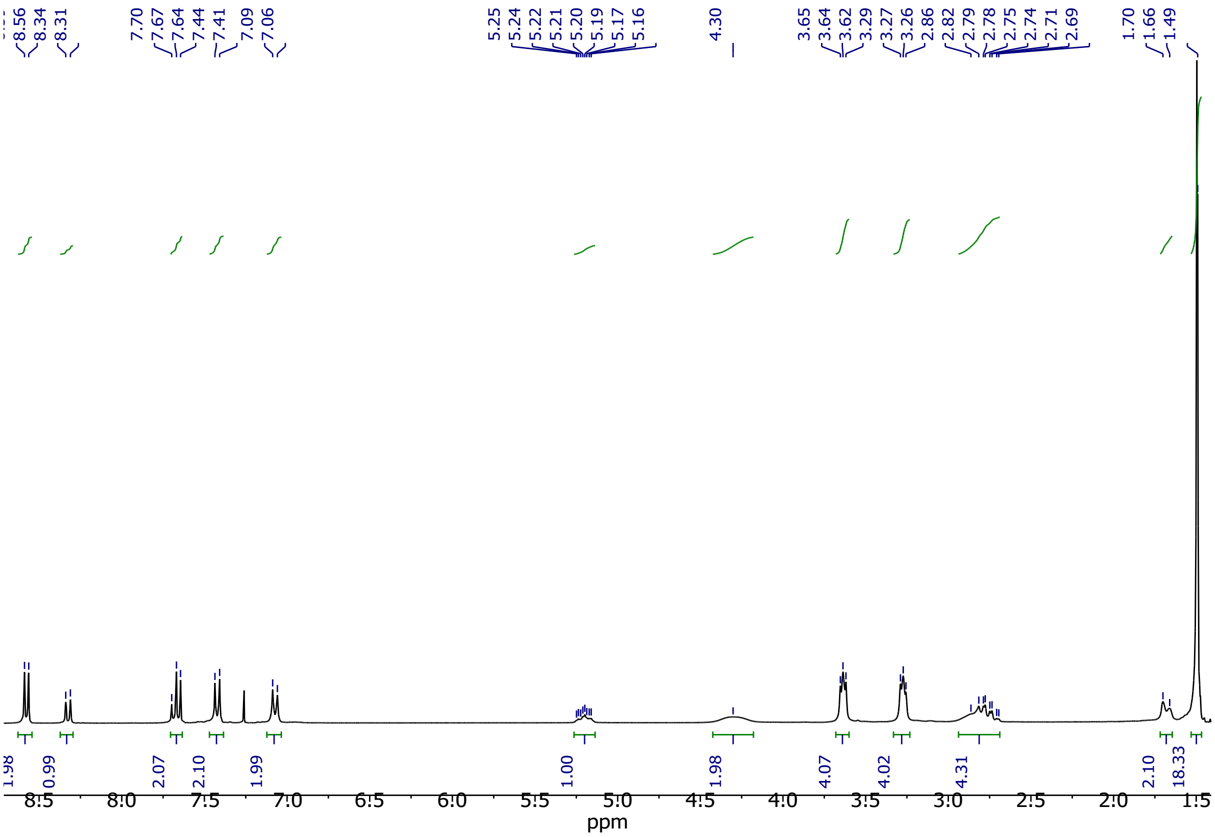

Figure S221. ^{1}H-NMR ($CDCl_3$, 300 MHz) of AR90p.

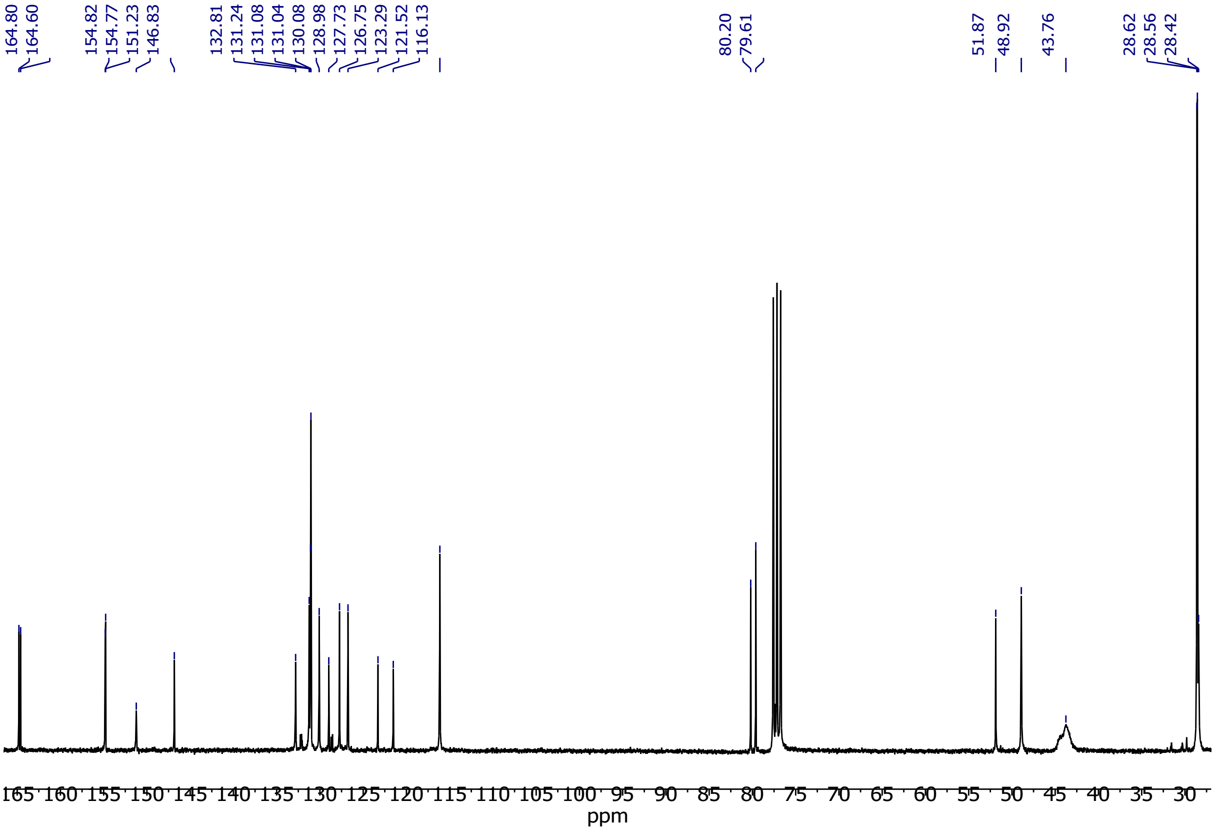

Figure S222. ^{13}C-NMR ($CDCl_3$, 75 MHz) of AR90p.

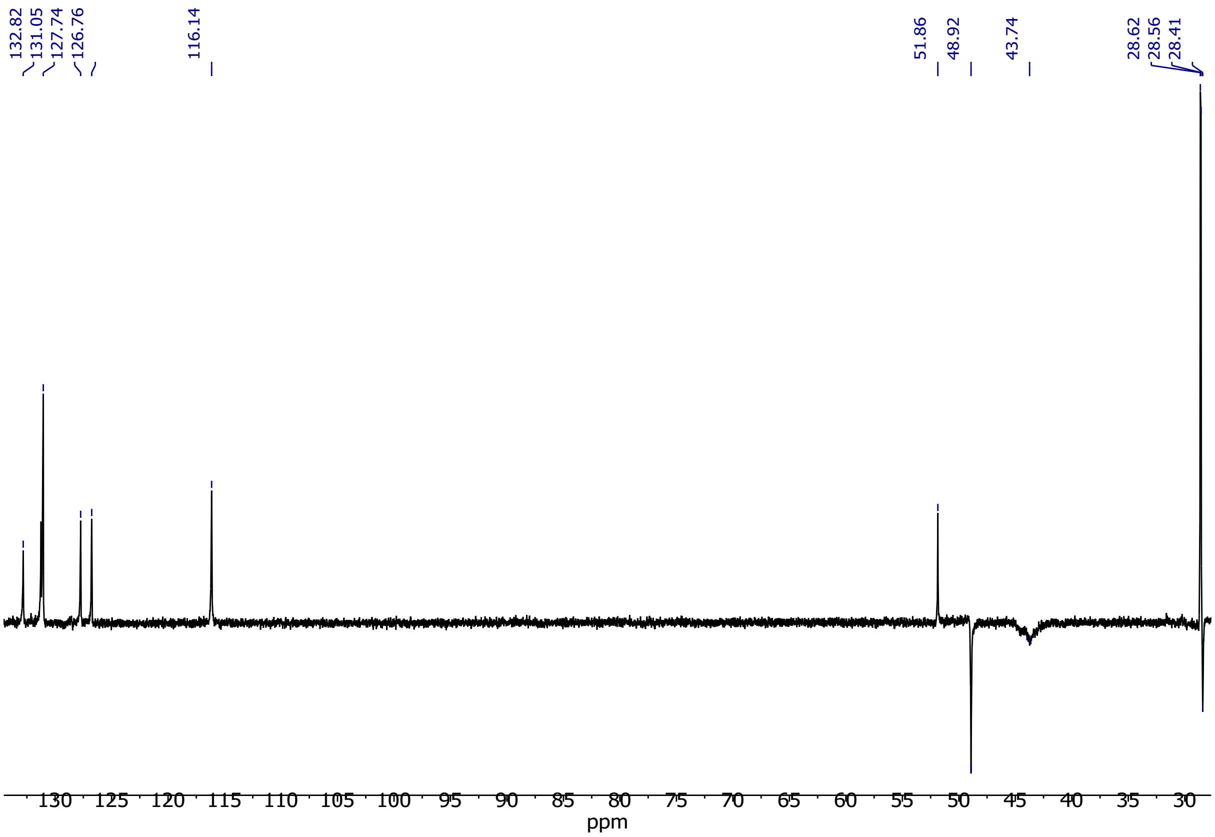

Figure S223. DEPT NMR ($CDCl_3$, 75 MHz) of AR90p.

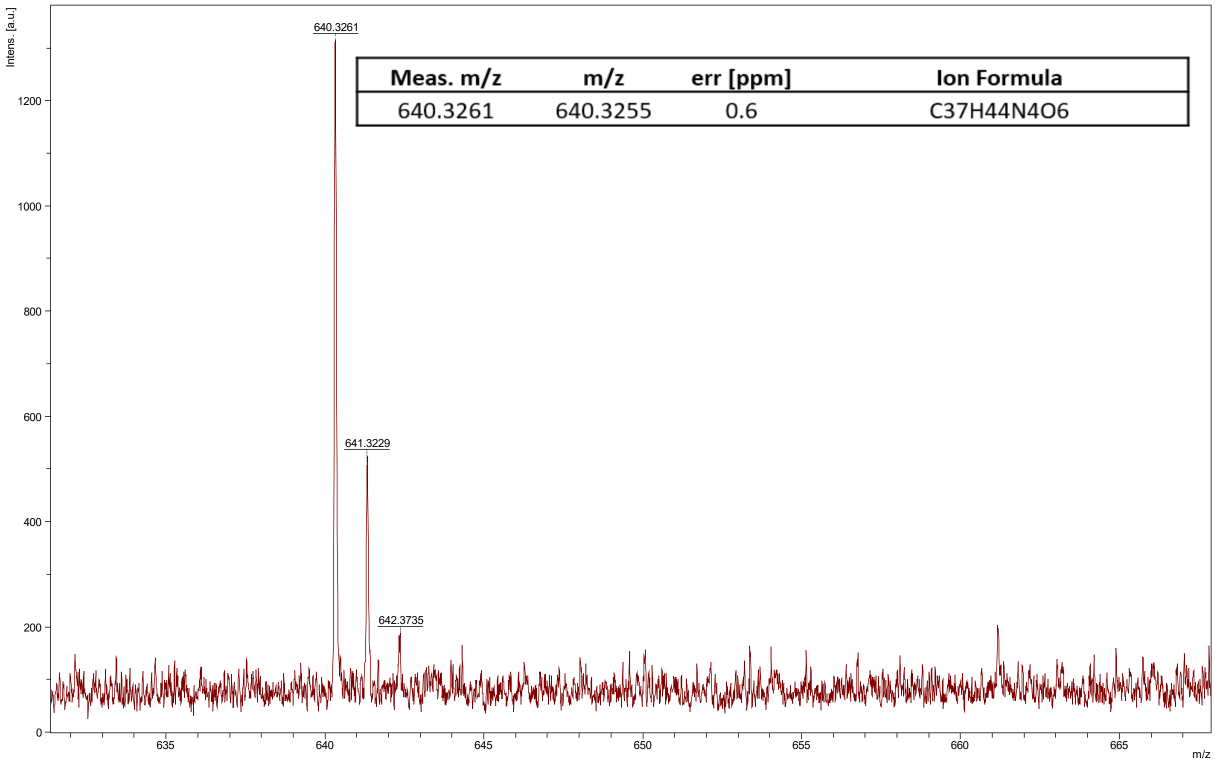

Meas. m/z	m/z	err [ppm]	Ion Formula
640.3261	640.3255	0.6	C37H44N4O6

Figure S224. HRMS (MALDI, DIT+) of AR90p

Solvatochromism:

The concentration of the compound was 20 µM and the excitation wavelength was 380 nm.

Figure S225. Solvatochromism of AR90p.

Absorbance

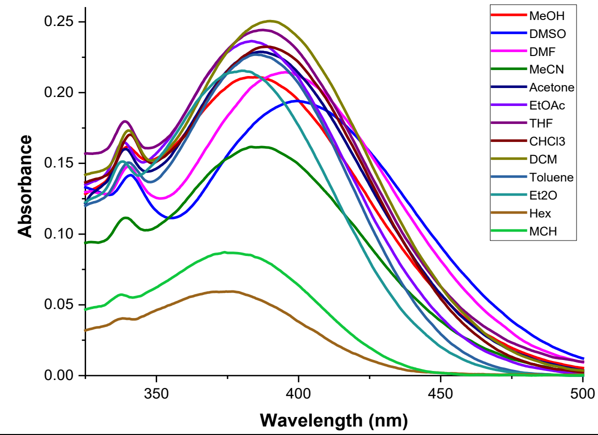

Normalized absorbance

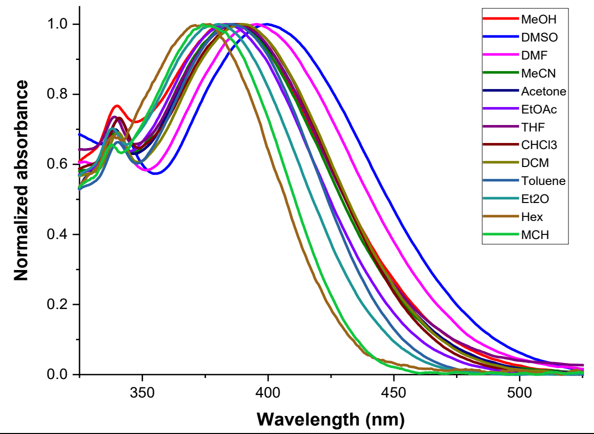

Emission

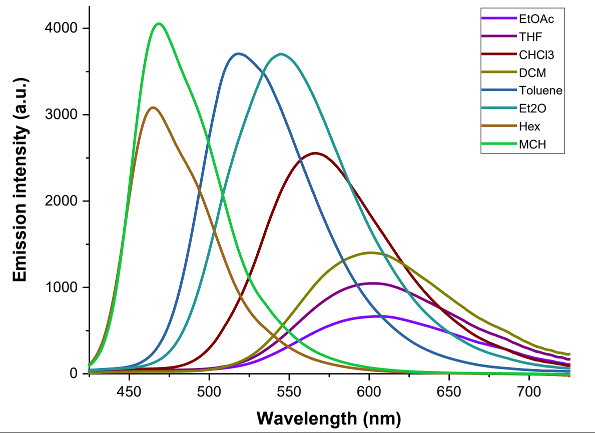

Normalized emission

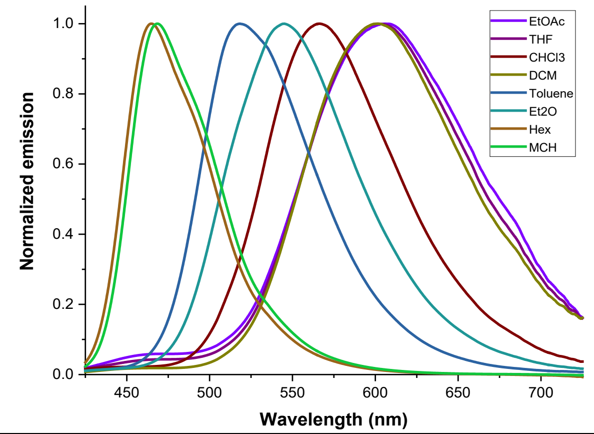

Figure S226. Solvatochromism of AR90p under visible and 366 nm light.

	H_2O MeOH DMSO DMF MeCN Acet. EtOAc THF $CHCl_3$ DCM Tol. Et_2O Hex MCH
**Visible light**	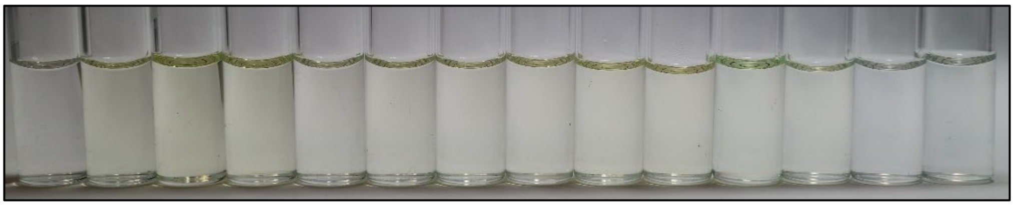
**UV light (366 nm)**	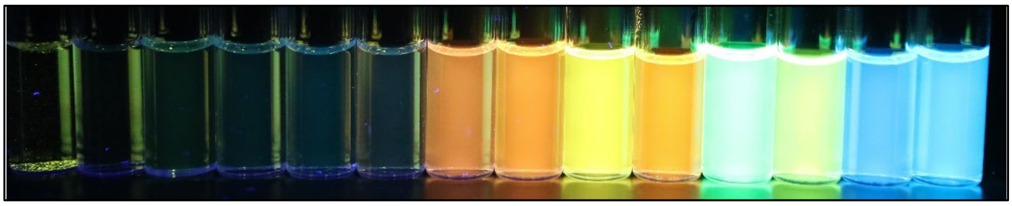

Fluorescence decay lifetime (τ):

The solvent used was MCH. The concentration of the compound was 20 µM.

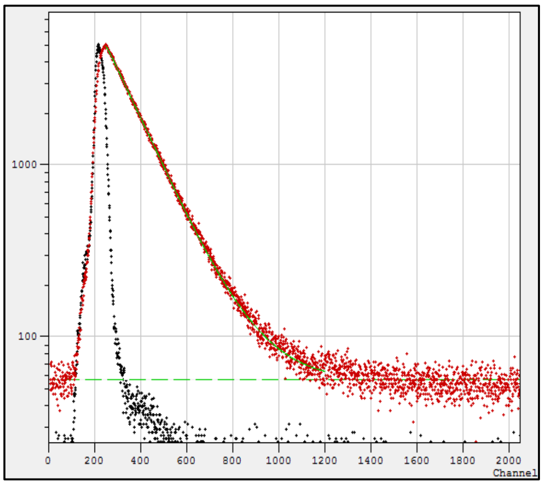

Figure S227. Fluorescence decay lifetime of AR90p.

Water acceptance test:

The solvent used was THF and the concentration of the compound was 20 µM.

Figure S228. Water acceptance test of AR90p under visible (up) and UV (down) light.

	**R 5% 10% 20% 30% 40% 50% 60% 70% 80% 90%**
**Visible light**	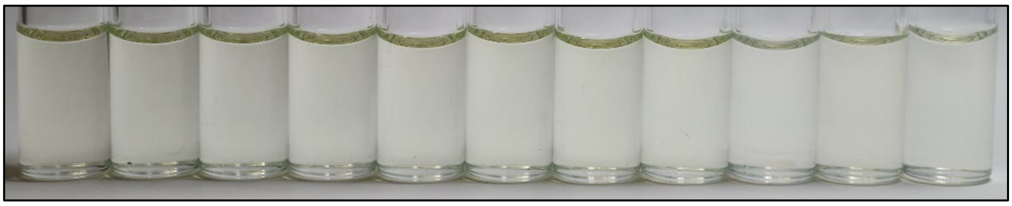
**UV light (366 nm)**	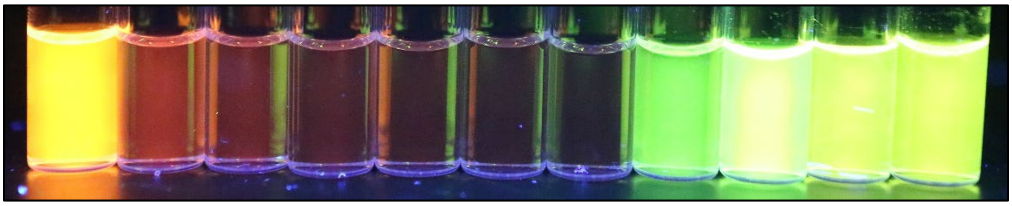

There was an increase in emission intensity from 60% water (AIE). At low amount of water, the maximum emission was shifted from red region to yellow greenish region.

pH test:

The amount of water for the performance of this test were 10% and 60%. The concentration of the compound was 20 µM and the solvent used was THF.

Figure S229. pH test of AR90p at 10% of water under visible (up) and UV (down) light.

	R HCl 3.4 4.8 6.4 7.4 7.9 10.5 KOH
**Visible light**	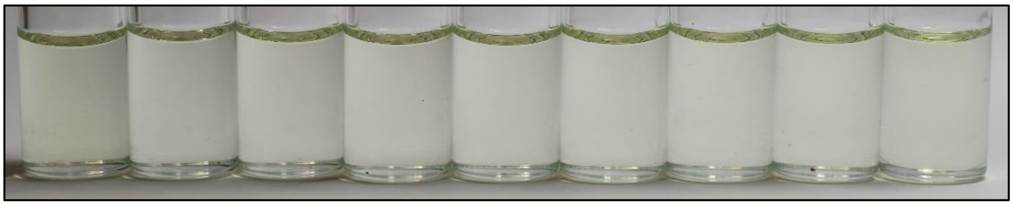
**UV light (366 nm)**	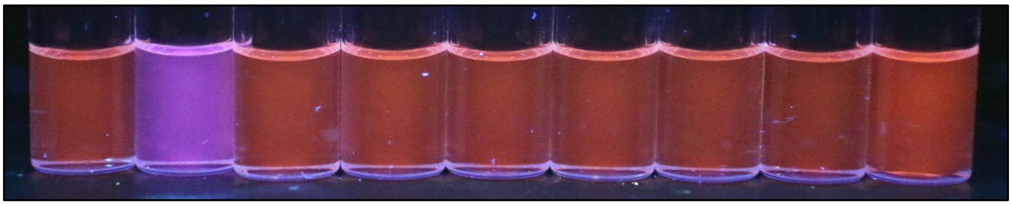

Figure S230. pH test of AR90p at 60% of water under visible (up) and UV (down) light.

	R HCl 3.4 4.8 6.4 7.4 7.9 10.5 KOH
**Visible light**	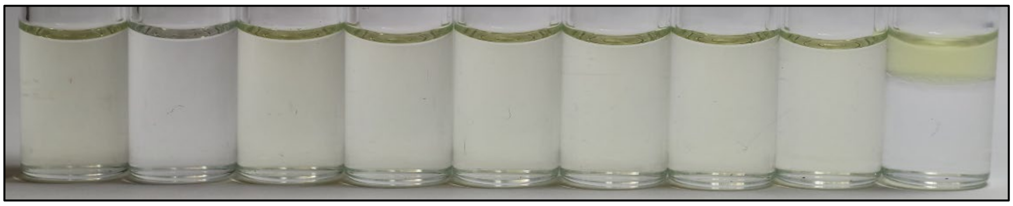
**UV light (366 nm)**	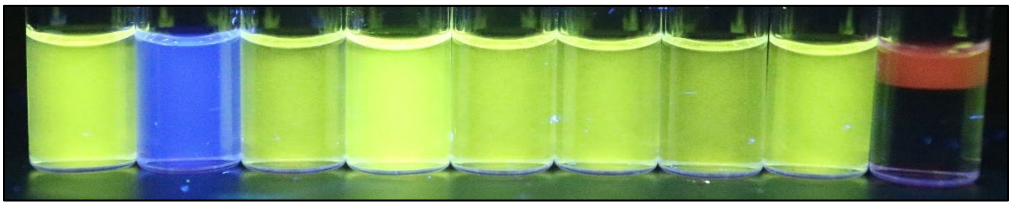

The most important change was the response to highly acidic media in both amounts of water.

Cations and anions test:

The concentration of the compound was 20 µM and the solvent used was THF.

Figure S231. Cations test of AR90p under visible (up) and UV (down) light.

	R Ag^{+} Ni^{2+} Sn^{2+} Cd^{2+} Zn^{2+} Pb^{2+} Cu^{2+} Fe^{3+} Sc^{3+} Al^{3+} Hg^{2+} Au^{3+} Co^{2+} Pd^{2+} Ir^{3+} Cu^{+} Ru^{3+} Pt^{2+}
**Visible light**	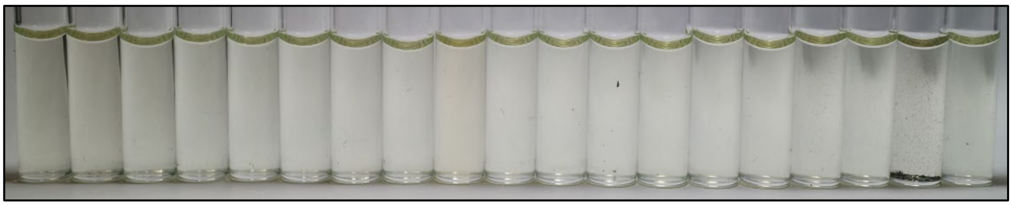

**UV light (366 nm)**	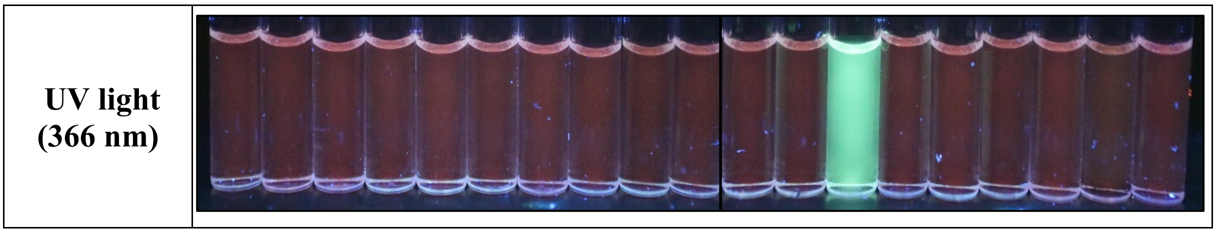

The only change observed was the change in fluorescence from red to green in the presence of Au^{3+}.

The concentration of the compound was 20 µM and the solvent used was THF.

Figure S232. Anions test of AR90p under visible (up) and UV (down) light.

	**R F^- Cl^- Br^- I^- BzO^- NO_3^- $H_2PO_4^-$ HSO_4^- AcO^- CN^- SCN^-**
**Visible light**	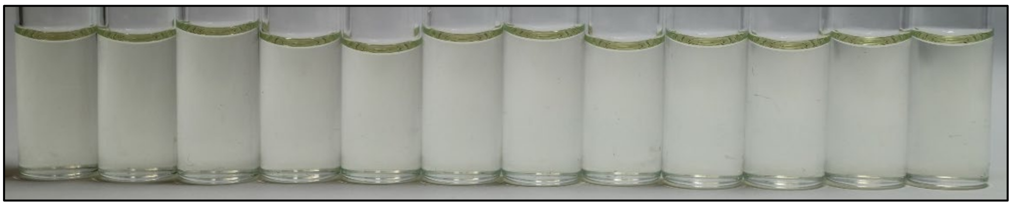
**UV light (366 nm)**	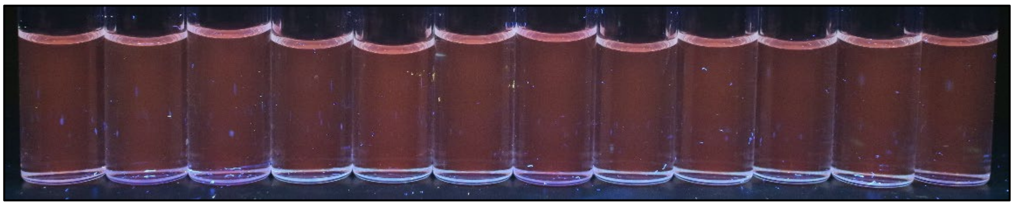

No significant changes were observed.

Oxidants and reductants test:

The solvent used was THF and concentration of the compound was 20 µM.

Figure S233. Oxidants and reductants test of AR90p under visible (up) and UV (down) light.

	**R HCl* HNO_3 Oxn Hz H_2O_2 R m-CPBA TNB TNT**
**Visible light**	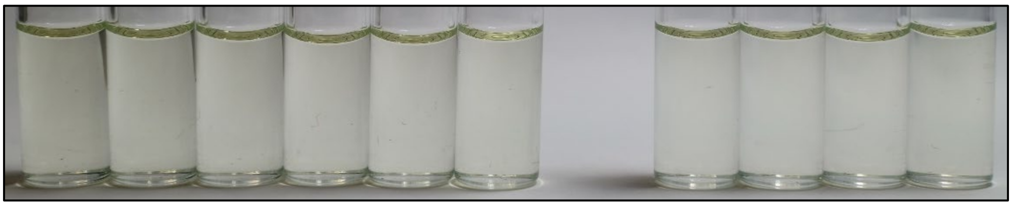
**UV light (366 nm)**	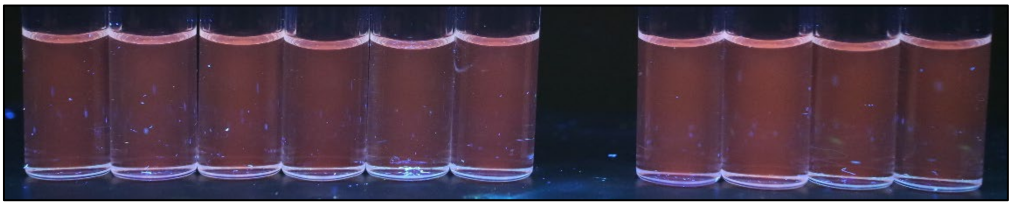

*HCl is included in all experiments in a close position to HNO_3 to distinguish between the redox action of HNO_3 and a possible effect due only to the concomitant acidity of nitric acid.
No significant changes were observed.

Synthesis of *N*-(piperidin-4-yl)-4-[(4-Boc-piperazin-1-yl)phenyl]naphthalene-1,8-dicarboxylmonoimide (AR90s)

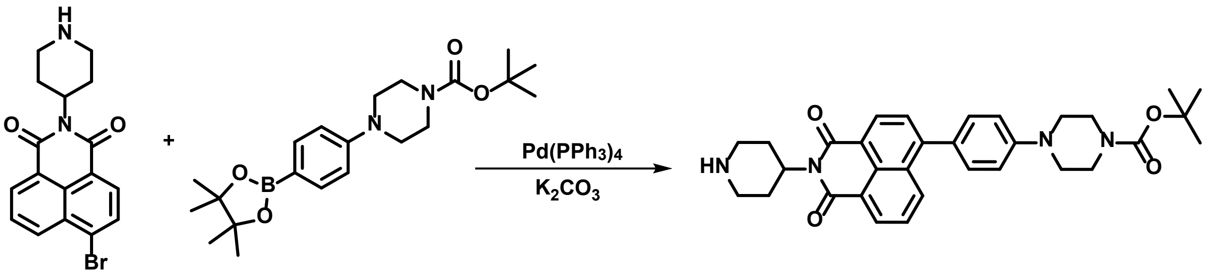

150 mg of *N*-(piperidin-4-yl)-4-bromonaphthalene-1,8-dicarboxylmonoimide (AR43d, 0.42 mmol), 179 mg of 4-(4-Boc-piperazin-1-yl)phenylboronic acid pinacol ester (0.44 mmol) and 451 mg of potassium carbonate (3.26 mmol) were dissolved in a mixture of toluene:*n*-butanol:water (4:1:2 ml) under nitrogen atmosphere and, then, the catalyst, $Pd(PPh_3)_4$ (5% mmol) was added and the resulting mixture was stirred overnight at 110ºC. After that, the solvent was evaporated under reduce pressure, the solid was dissolved in CH_2Cl_2 and washed with water (3×100 ml). The organic extracts were combined, dried over anhydrous Na_2SO_4 and the solvent was evaporated under reduce pressure. The organic solid was purified by column chromatography (SiO_2, CH_2Cl_2:MeOH) obtaining 190 mg of AR90s as a brown solid, 84% yield. m.p.: 188 – 190ºC. IR (ATR, cm^{-1}): 3407 (N-H), 3054 – 2851, 1689 (C=O), 1590, 1489, 1399, 1363, 1235, 1159 (C-N), 1118, 992, 929, 809, 750, 717, 693, 540. ^{1}H-NMR (300 MHz, $CDCl_3$) δ (ppm): 8.59 (d, *J* = 7.5 Hz, 2H, 2×H_{Ar}), 8.33 (dd, *J* = 8.5, 0.9 Hz, 1H, H_{Ar}), 7.68 (t, *J* = 8.0 Hz, 2H, 2×H_{Ar}), 7.43 (d, *J* = 8.6 Hz, 2H, 2×H_{Ar}), 7.07 (d, *J* = 8.7 Hz, 2H, 2×H_{Ar}), 5.21 (m, 1H, CH), 3.64 (m, 4H, 2×CH_2), 3.27 (m, 6H, 3×CH_2), 2.86 – 2.68 (m, 4H, 2×CH_2), 2.14 (s, 1H, NH), 1.73 (m, 2H, CH_2), 1.50 (s, 9H, 3×CH_3). ^{13}C-NMR (75MHz, $CDCl_3$) δ (ppm): 164.6 (C=O), 164.4 (C=O), 154.8 (C_{Ar}), 151.3 (C_{Ar}), 147.1 (C_{Ar}), 133.1 (C_{Ar}H), 131.5 (C_{Ar}H) , 131.4 (C_{Ar}H), 131.0 (C_{Ar}H), 130.1 (C_{Ar}), 129.9 (C_{Ar}), 129.0 (C_{Ar}), 127.8 (C_{Ar}H), 126.8 (C_{Ar}H), 122.9 (C_{Ar}), 121.1 (C_{Ar}), 116.1 (C_{Ar}), 80.2 (Cq), 48.8 (CH_2), 44.7 (CH_2), 43.6 (CH_2), 28.6 (CH_3), 25.8 (CH_2). UV-Vis ($CHCl_3$), λ_{max} nm (log ε): 310 (4.0). HRMS (MALDI, DIT+) m/z: calculated for $C_{32}H_{36}N_4O_4$+H: 541.2815 (M^++H); found 541.2807. Ø (THF, %): 3.07. τ (280 nm, THF, ns): 1.786/6.278 (χ^2: 1.114).

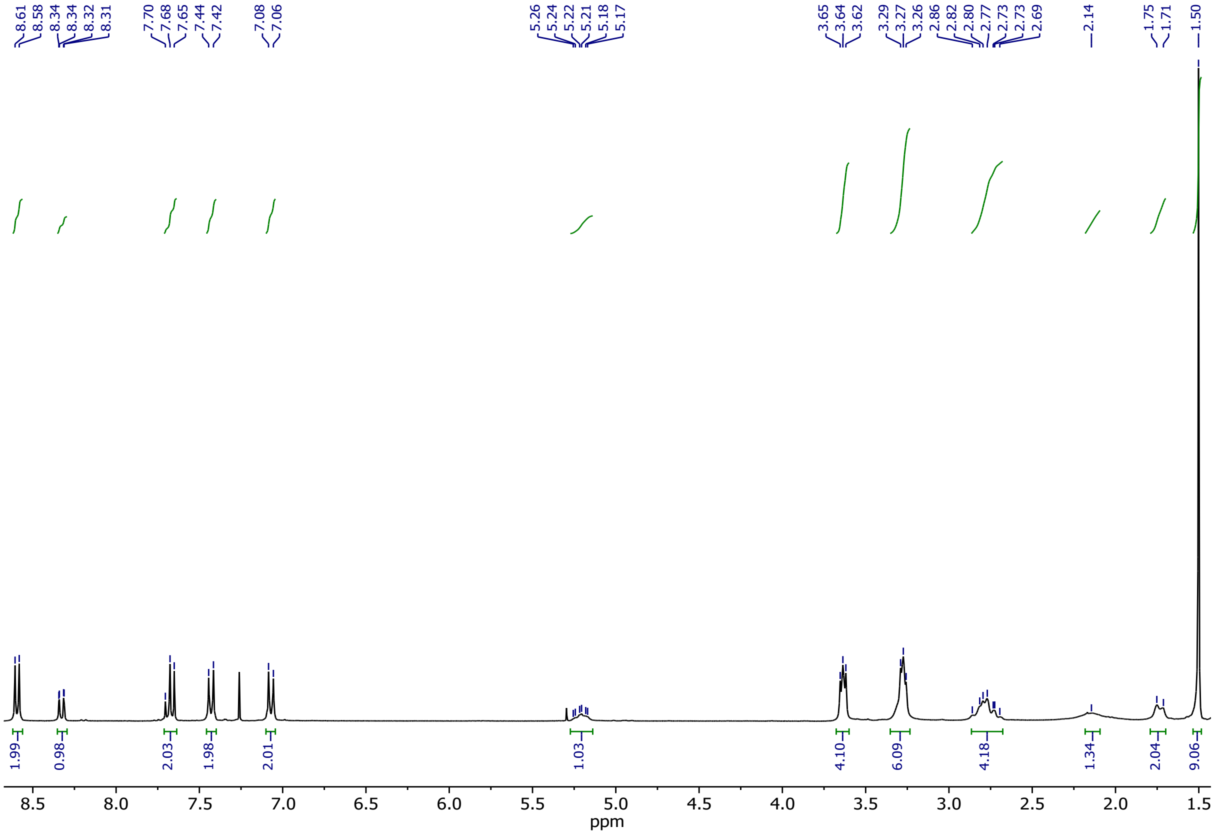

Figure S234. ¹H-NMR ($CDCl_3$, 300 MHz) of AR90s.

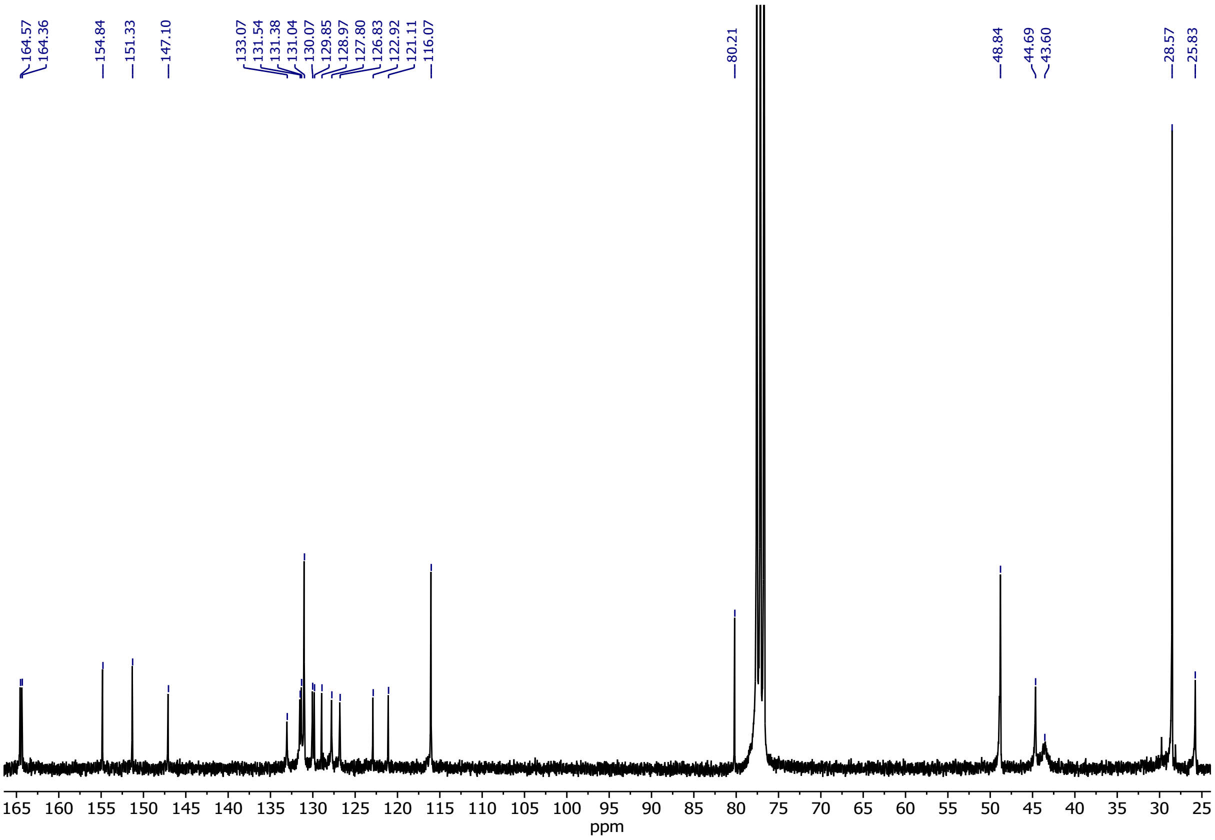

Figure S235. ¹³C-NMR ($CDCl_3$, 75 MHz) of AR90s.

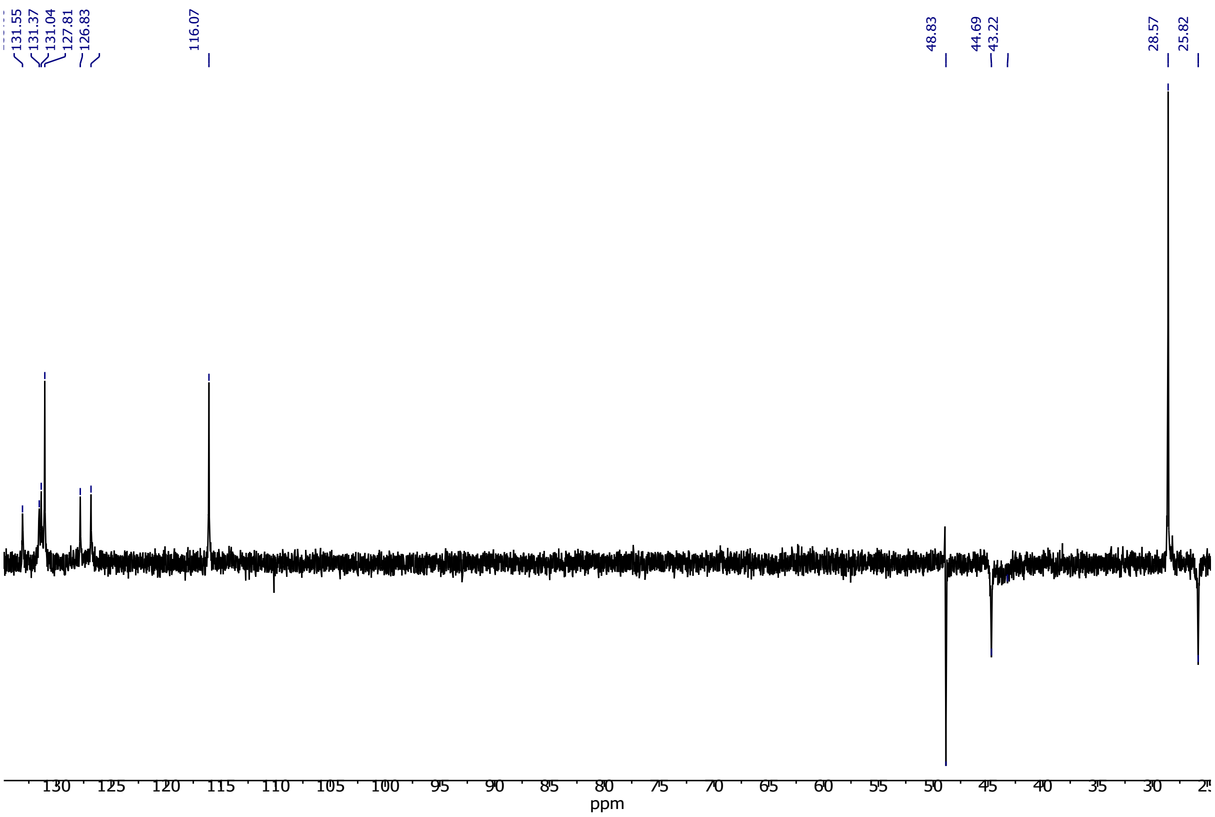

Figure S236. DEPT NMR ($CDCl_3$, 75 MHz) of AR90s.

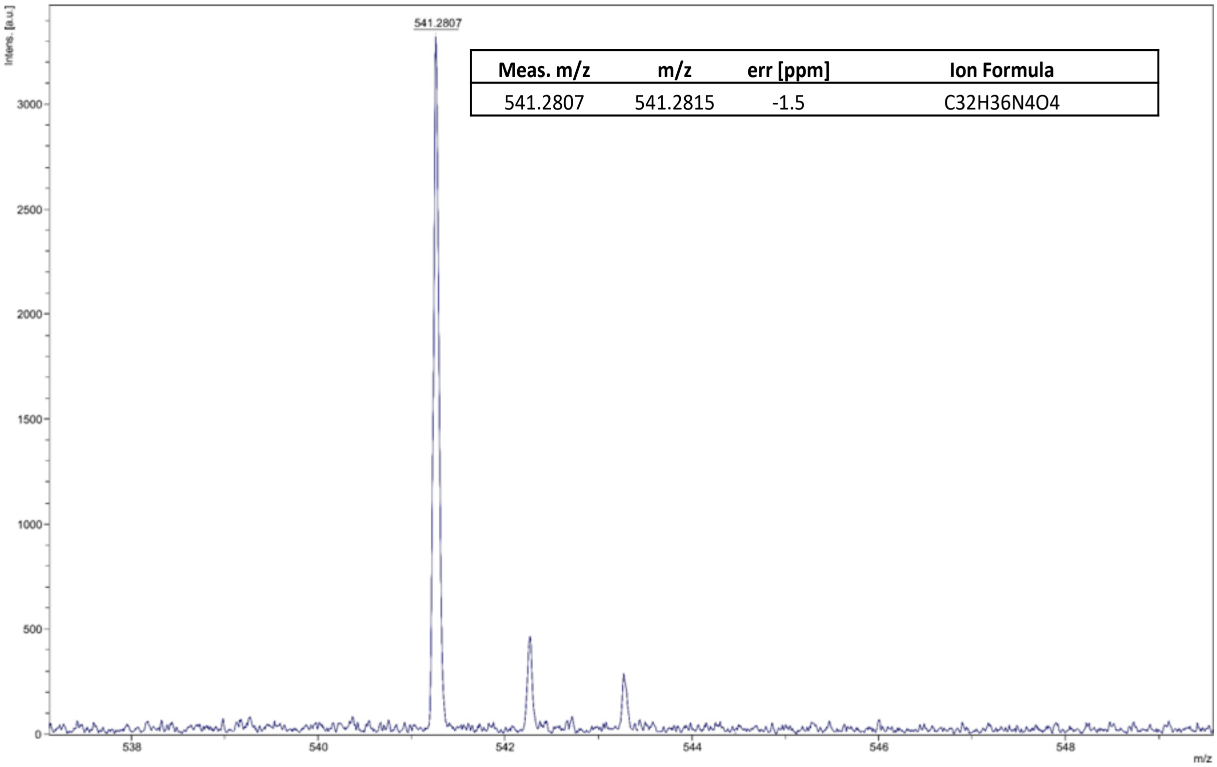

Figure S237. HRMS (MALDI, DIT+) of AR90s

Fluorescence decay lifetime (τ):

The solvent used was THF. The concentration of the compound was 20 µM.

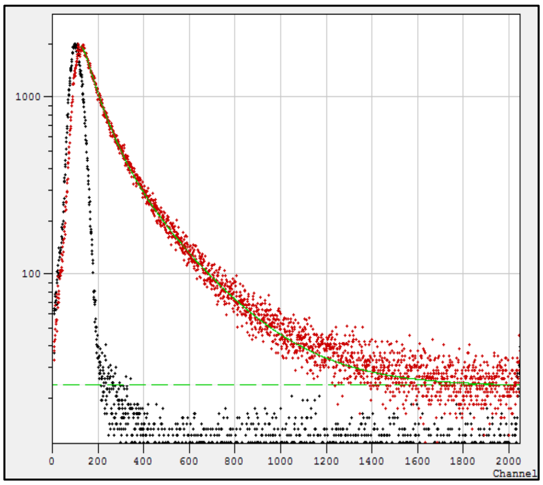

Figure S238. Fluorescence decay lifetime of AR90s.

Solvatochromism:

The concentration of the compound was 20 µM and the excitation wavelength was 310 nm.

Figure S239. Solvatochromism of AR90s.

**Absorbance**	**Normalized absorbance**
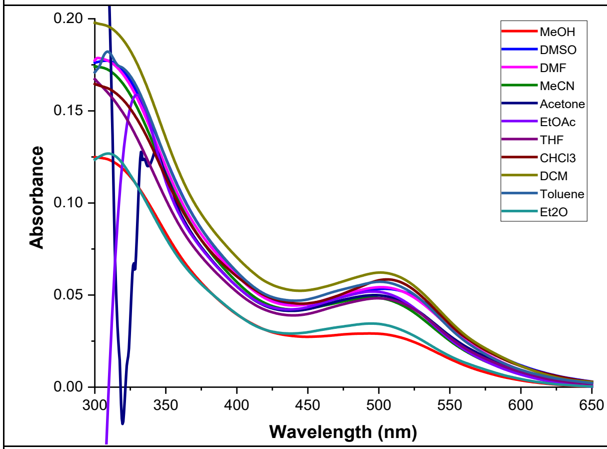	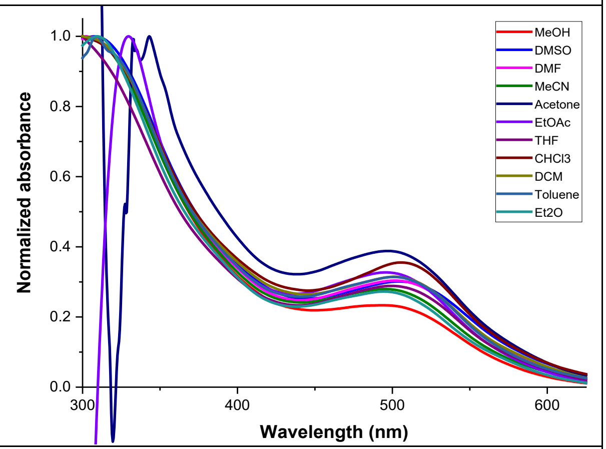
**Emission**	**Normalized emission**

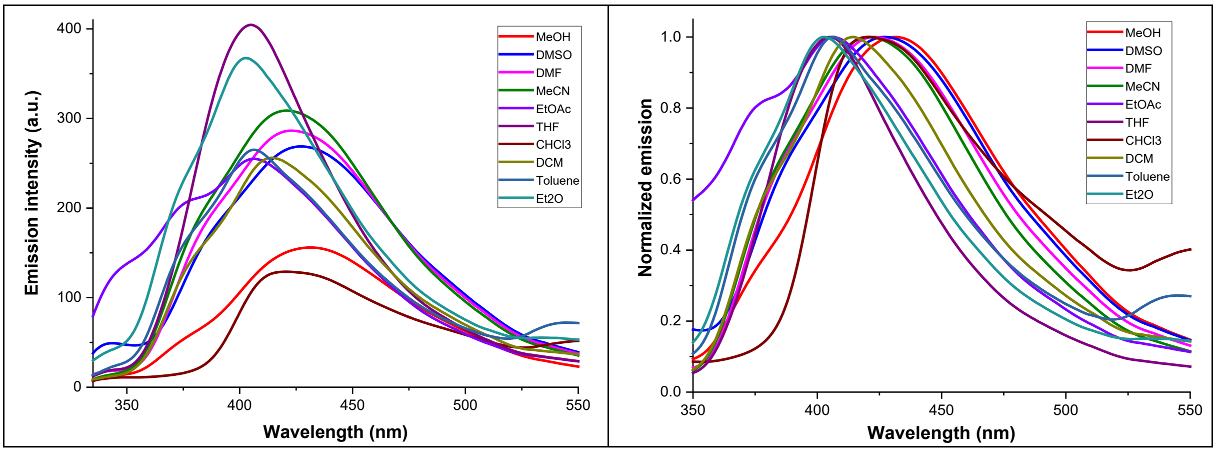

Figure S240. Solvatochromism of AR90s under visible and 366 nm light

	H_2O MeOH DMSO DMF MeCN Acet. EtOAc THF $CHCl_3$ DCM Tol. Et_2O Hex MCH
**Visible light**	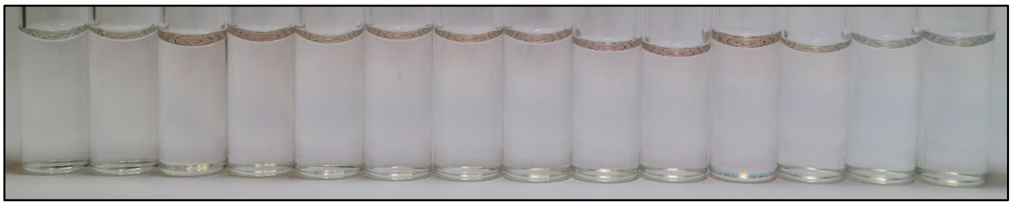
**UV light (366 nm)**	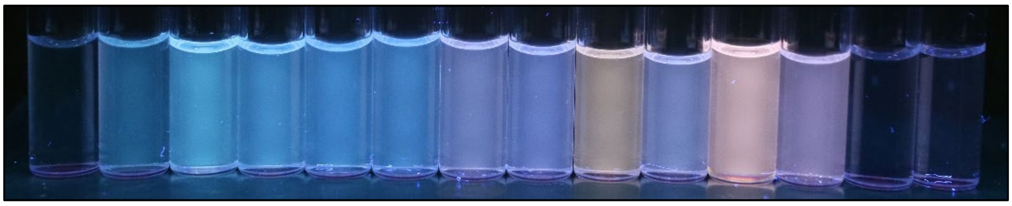

Water acceptance test:

The solvent used was MeOH and the concentration of the compound was 20 μM.

Figure S241. Water acceptance test of AR90s under visible (up) and UV (down) light.

	R 5% 10% 20% 30% 40% 50% 60% 70% 80% 90%
**Visible light**	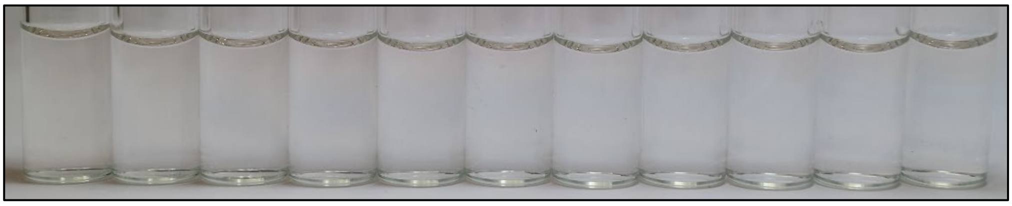
**UV light (366 nm)**	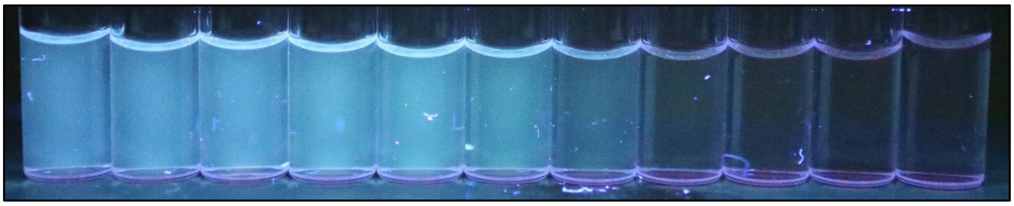

The maximum amount of water that was admitted by the product was 40%. From 60% water, the fluorescence was quenched (ACQ).

pH test:

The amount of water for the performance of this test was 40%. The concentration of the compound was 20 µM and the solvent used was MeOH.

Figure S242. pH test of AR90s under visible (up) and UV (down) light.

	R HCl 3.4 4.8 6.4 7.4 7.9 10.5 KOH
**Visible light**	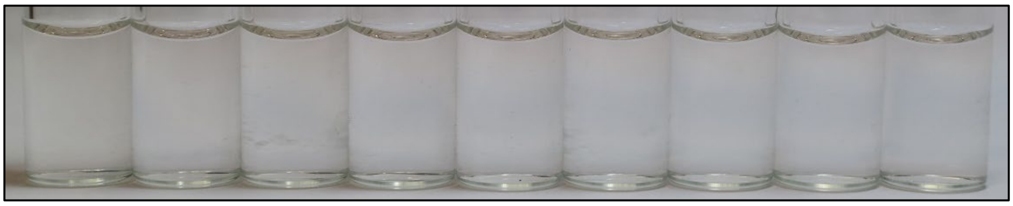
**UV light (366 nm)**	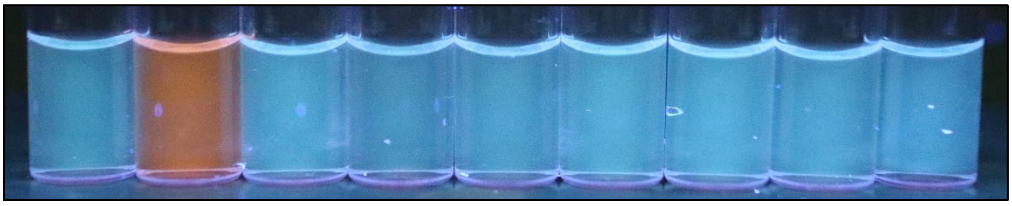

The most important change in fluorescence was the response to highly acidic media.

Cations and anions test:

The concentration of the compound was 20 µM and the solvent used was MeOH.

Figure S243. Cations test of AR90s under visible (up) and UV (down) light.

	R Ag^{+} Ni^{2+} Sn^{2+} Cd^{2+} Zn^{2+} Pb^{2+} Cu^{2+} Fe^{3+} Sc^{3+} Al^{3+} Hg^{2+} Au^{3+} Co^{2+} Pd^{2+} Ir^{3+} Cu^{+} Ru^{3+} Pt^{2+}
**Visible light**	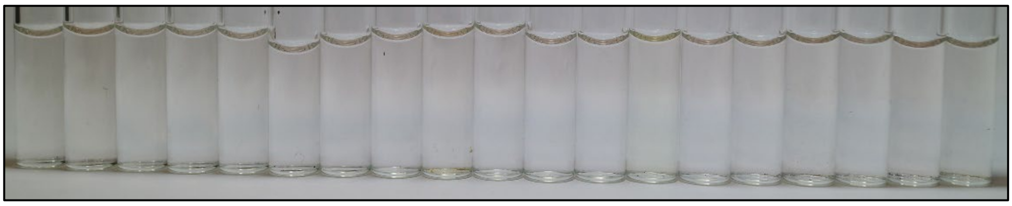
**UV light (366 nm)**	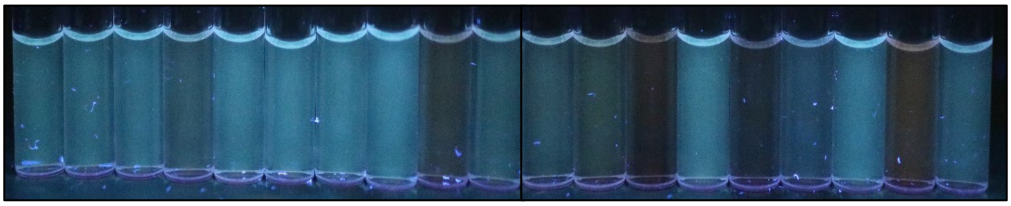

There was fluorescence quenching in the presence of Fe^{3+}, Au^{3+}, Pd^{2+} and Ru^{3+}.
The concentration of the compound was 20 µM and the solvent used was MeOH.

Figure S244. Anions test of AR90s under visible (up) and UV (down) light.

	R F^{-} Cl^{-} Br^{-} I^{-} BzO^{-} NO_3^{-} $H_2PO_4^{-}$ HSO_4^{-} AcO^{-} CN^{-} SCN^{-}
**Visible light**	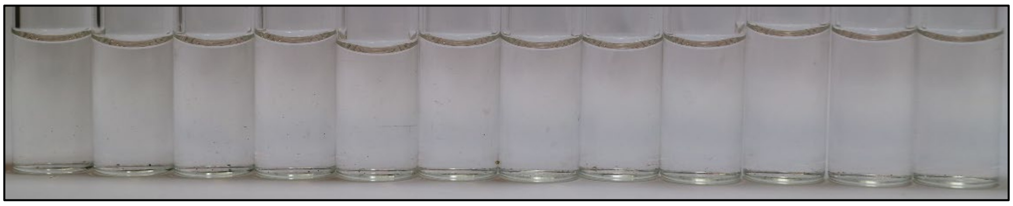

**UV light (366 nm)**	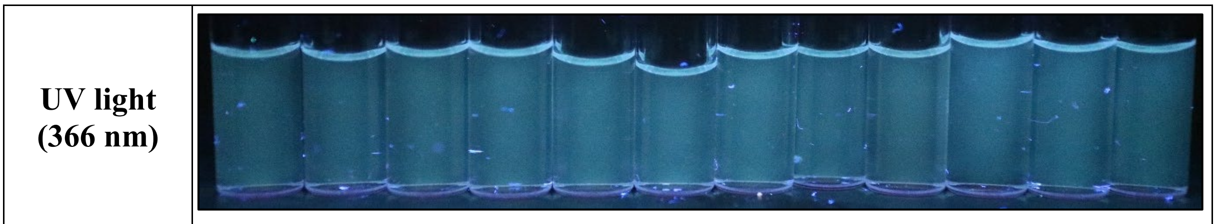

No significant changes were observed.

Oxidants and reductants test:

The solvent used was THF and concentration of the compound was 20 μM.

Figure S245. Oxidants and reductants test of AR90s under visible (up) and UV (down) light.

	R HCl* HNO_3 Oxn Hz H_2O_2 R m-CPBA TNB TNT R m-CPBA TATP HMTD
**Visible light**	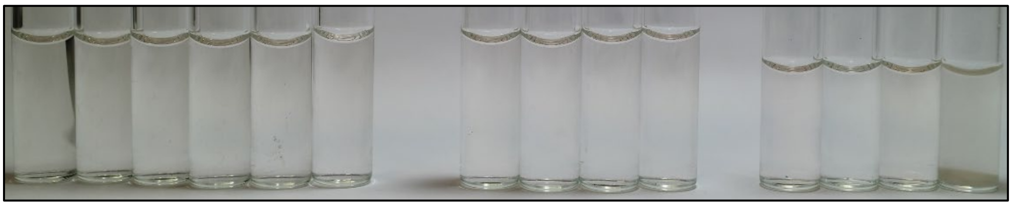
**UV light (366 nm)**	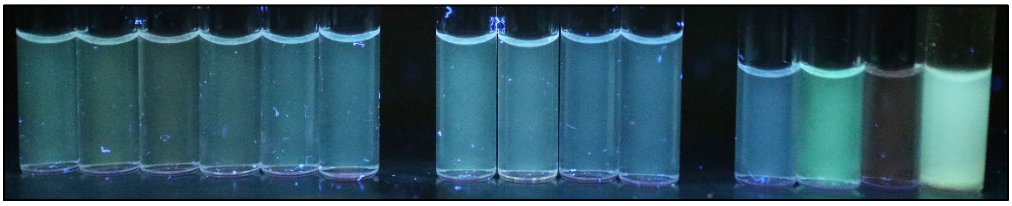

*HCl is included in all experiments in a close position to HNO_3 to distinguish between the redox action of HNO_3 and a possible effect due only to the concomitant acidity of nitric acid.

The most significant change was the fluorescence quenching when exposed to TATP. In addition, in front of HMTD the color of the fluorescence became turquoise blue.

Preliminary solvents test:

In order to determine the fluorescence variation of the compound under study in the presence of TATP, some test were carried out for the probe in 9 different solvents. The AR90s concentration was 20 μM and the TATP was added in excess (7 mg in each vial). All the test were performed at room temperature and the photographs were taken immediately after the addition of TATP and again after 72 hours.

Figure S246. Pairs of AR90s (left) and AR90s with excess of TATP (right) in different solvents at time 0 h (up) and 72 h (down).

	DMF MeCN EtOAc THF $CHCl_3$ DCM Tol $CHCl_3$:MeOH DCM:MeOH
**t = 0 h**	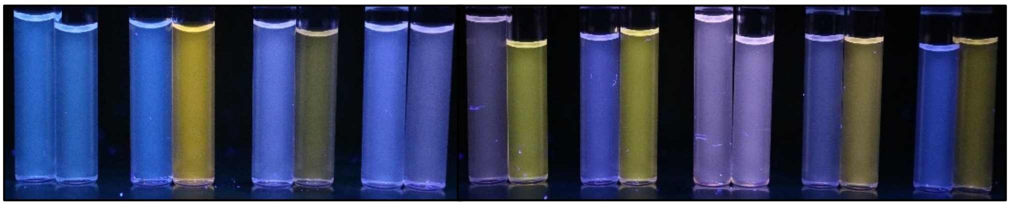
**t = 72 h**	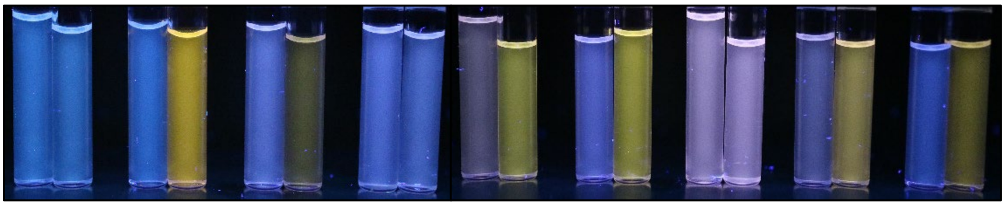

The most noticeable fluorescence changes were seen in EtOAc, MeCN, $CHCl_3$, DCM, $CHCl_3$:MeOH (9:1) and DCM:MeOH (9:1) so kinetic studies and TATP titrations were carried out in these solvents.

Solvents test with TATP:

For all solvents, the working concentration of AR90s was 2.5 µM, the excitation wavelenght was 310 nm and the temperature for all test was 25ºC.

In the kinetic studies, TATP was in excess (20 mM) and the fluorescence emission measurements were made during the subsequent minutes at 5 min, 10 min and 30 min time intervals. In the titration, the TATP concentration was between 0 and 35000 µM and it was added directly as solid. The measurements were carried out inmediately after the adittion of the probe.

Figure S247. Study of AR90s with TATP in MeCN. Kinetic study (up left) and profile as function as time at 420 nm (up right) in presence of TATP excess. Titration (down left) and fluorescence profile at 415 nm and 560 nm simultaneously (down right) under increasing concentrations of TATP.

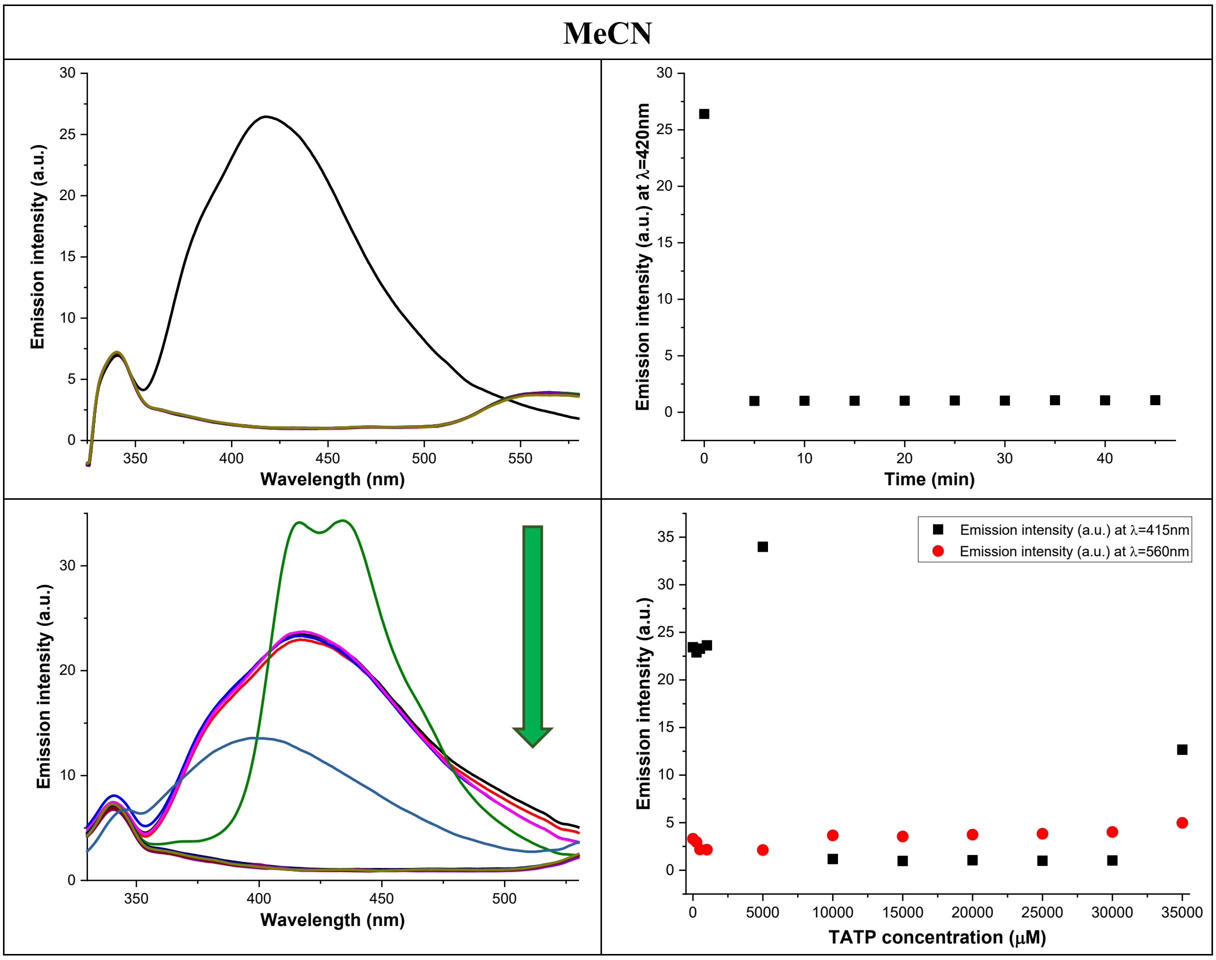

There was a decrease change in the emission intensity band immediately after adding TATP. The LOD of TATP was somewhere between 5000 and 10000 µM, a fairly high limit, with a decrease in emission intensity.

Figure S248. Kinetic study at different times under visible (up) and UV (down) light in MeCN. In each photo, left vial was contained the probe and right vial was contained the probe and an excess of TATP.

	**t=0**	**t=40 min**	**t=24 h**
**Visible light**	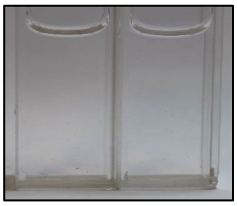	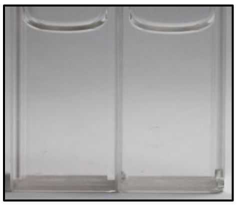	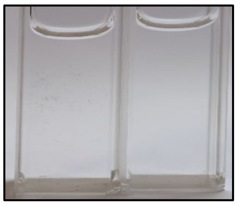
**UV light (366 nm)**	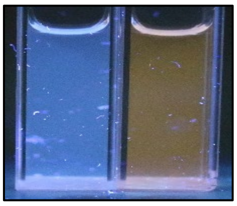	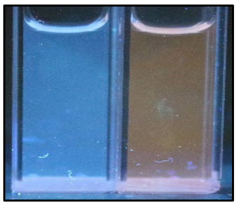	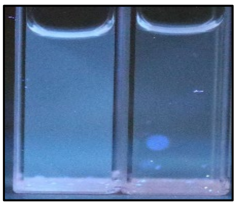

Figure S249. Titration of AR90s with an excess of TATP under visible (up) and UV (down) light in MeCN.

μM	**0 250 500 1000 5000 10000 15000 20000 25000 30000 35000**
**Visible light**	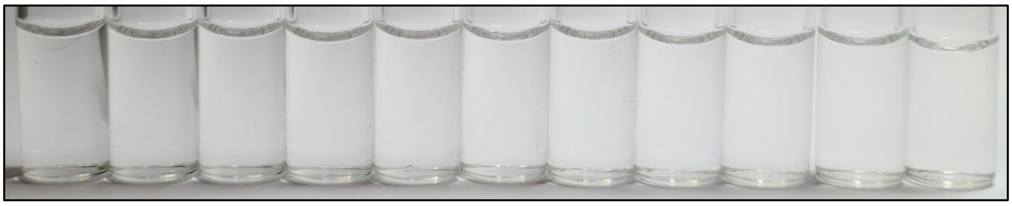
**UV light (366 nm)**	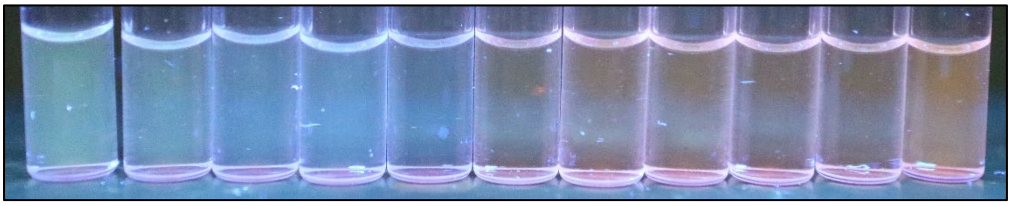

Figure S250. Study of AR90s with TATP in DCM. Kinetic study (up left) and profile as function as time at 415 nm and 550 nm simultaneously (up right) in presence of TATP excess.

Titration (down left) and fluorescence profile at 415 nm and 560 nm simultaneously (down right) under increasing concentrations of TATP.

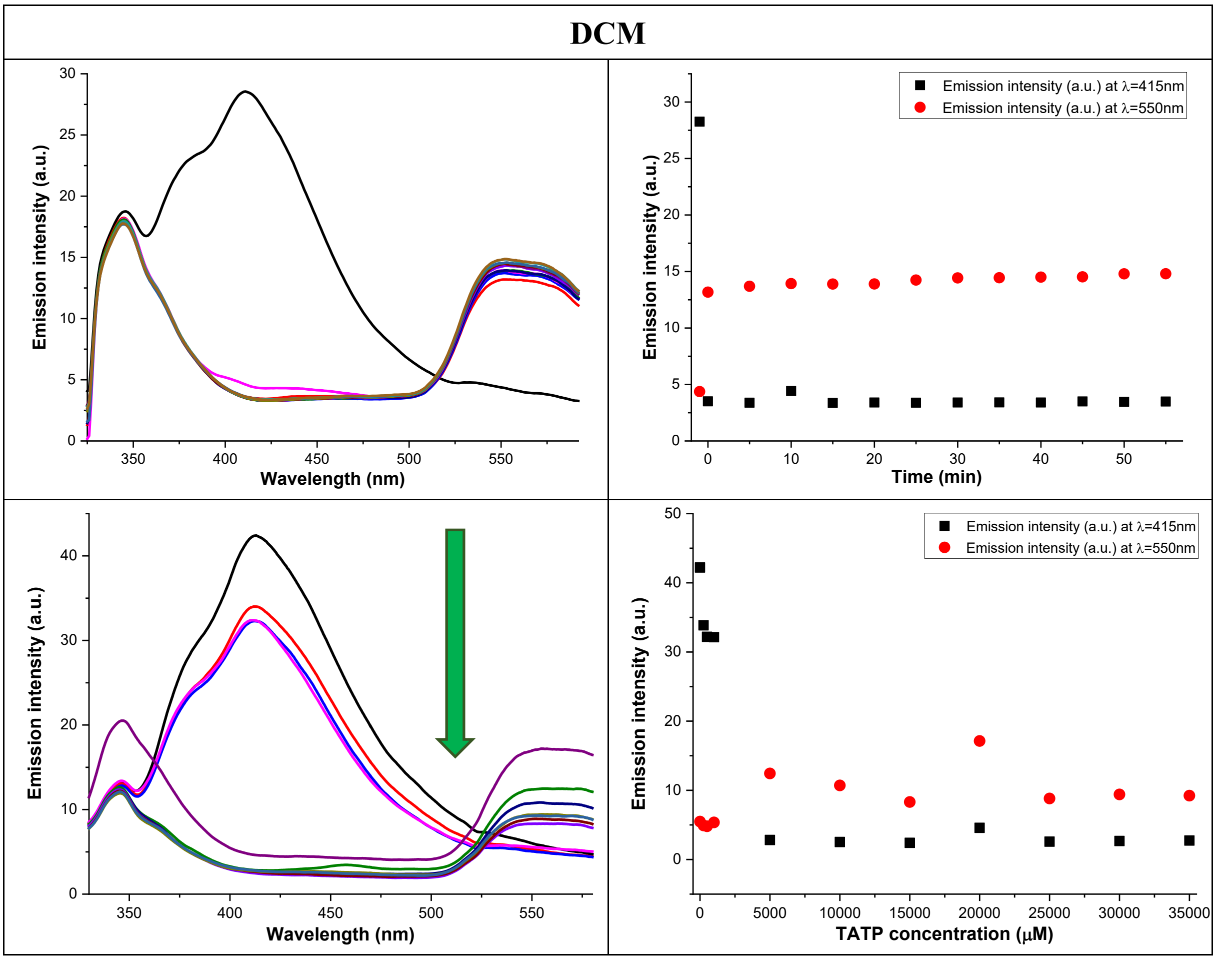

There was a decrease in the emission intensity band immediately after adding TATP. The LOD of TATP was somewhere between 1000 and 5000 μM, slightly less than in the previous solvent, but still too high a range, with a decrease in emission intensity.

Figure S251. Kinetic study at different times under visible (up) and UV (down) light in DCM. In each photo, left vial was contained the probe and right vial was contained the probe and an excess of TATP.

	**t=0**	**t=24 h**
**Visible light**	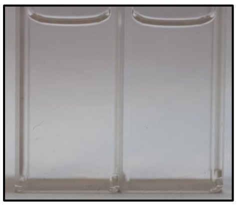	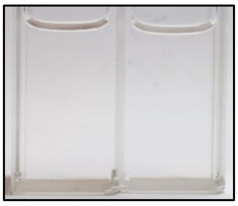
**UV light (366 nm)**	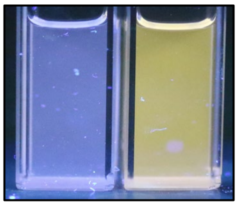	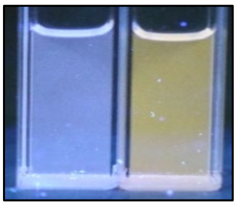

Figure S252. Titration of AR90s with an excess of TATP under visible (up) and UV (down) light in DCM.

µM	0 250 500 1000 5000 10000 15000 20000 25000 30000 35000
**Visible light**	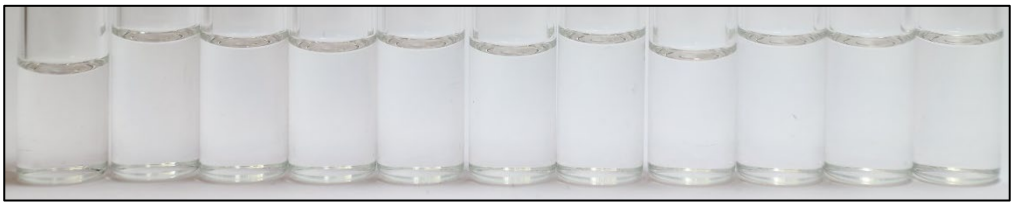
**UV light (366 nm)**	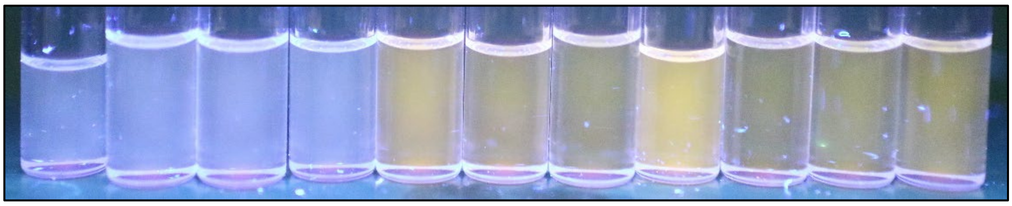

Figure S253. Study of AR90s with TATP in $CHCl_3$. Kinetic study (up left) and profile as function as time at 420 nm and 545 nm simultaneously (up right) in presence of TATP excess. Titration (down left) and fluorescence profile at 420 nm and 545 nm simultaneously (down right) under increasing concentrations of TATP.

$CHCl_3$

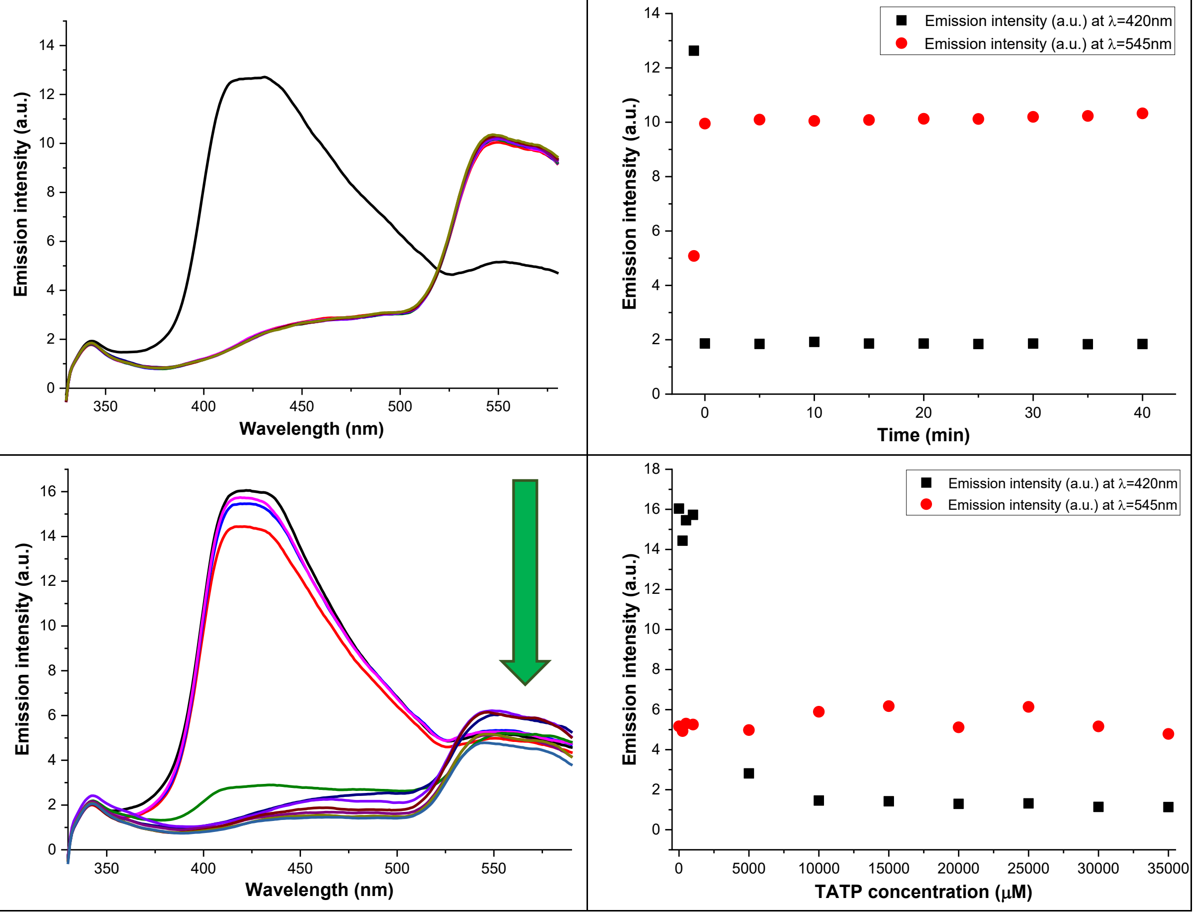

There was a decrease in the emission intensity band immediately after adding TATP. According to the fluorescence spectra, the LOD of TATP was somewhere between 1000 and 5000 µM, as in the previous solvent, with a strong decrease in emission intensity.

Figure S254. Kinetic study at different times under visible (up) and UV (down) light, $CHCl_3$. In each photo, left vial contained probe and right vial contained probe and excess TATP.

	**t=0**	**t=40 min**	**t=24 h**
**Visible light**	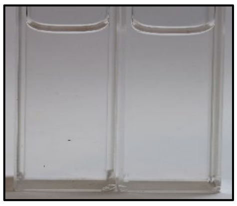	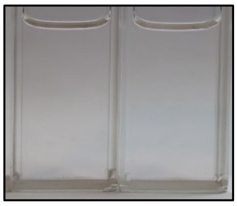	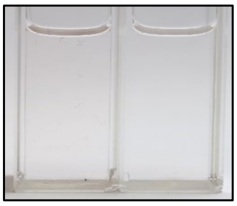
**UV light (366 nm)**	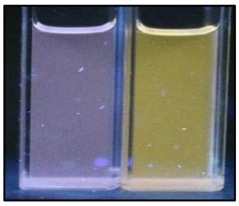	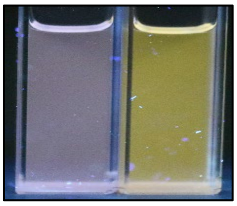	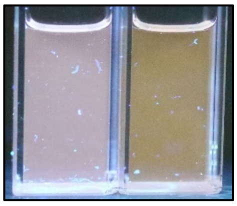

Figure S255. Titration of AR90s with an excess of TATP under visible (up) and UV (down) light in $CHCl_3$.

µM	0 250 500 1000 5000 10000 15000 20000 25000 30000 35000
**Visible light**	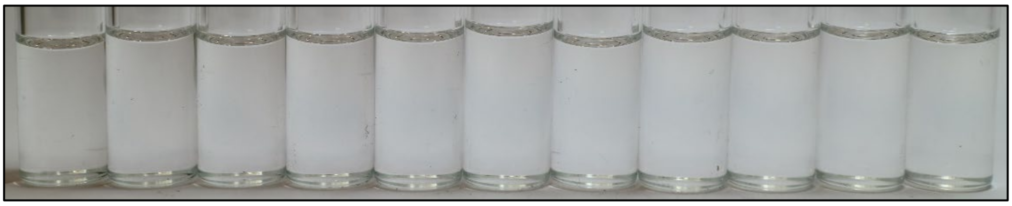
**UV light (366 nm)**	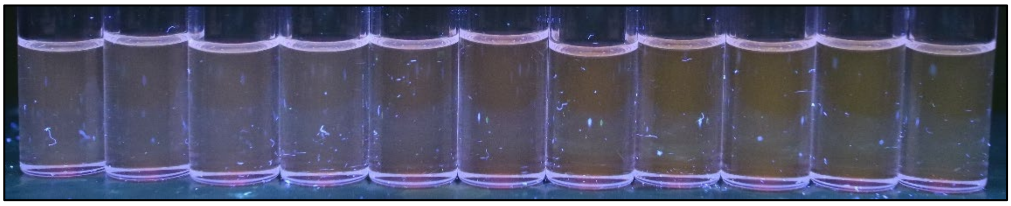

Figure S256. Study of AR90s with TATP in EtOAc. Kinetic study (up left) and profile as function as time at 355 nm and 405 nm simultaneously (up right) in presence of TATP excess. Titration (down left) and fluorescence profile at 355 nm and 405 nm simultaneously (down right) under increasing concentrations of TATP.

**EtOAc**	
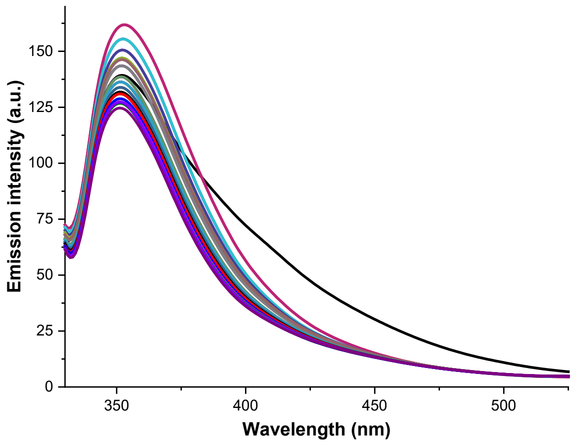	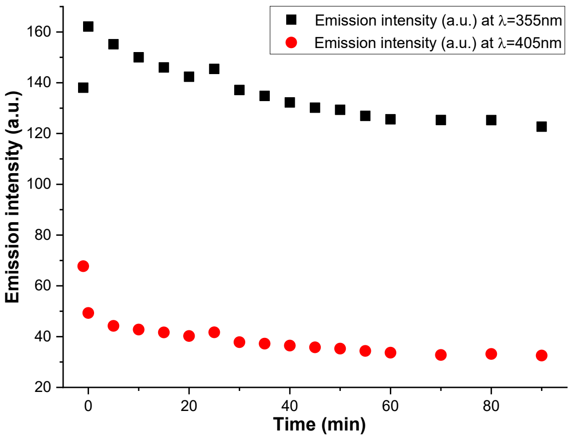

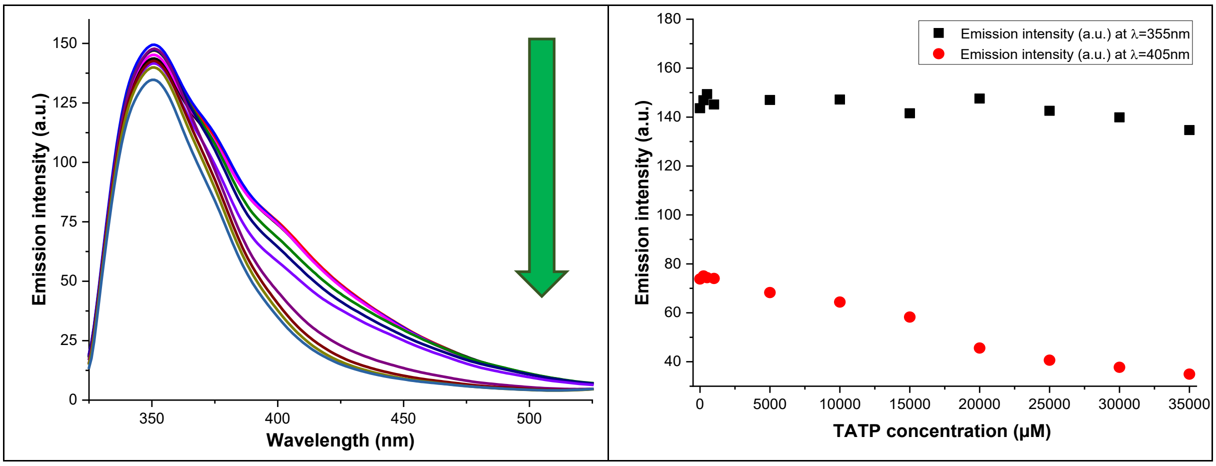

There was a decrease in emission intensity as time elapsed until stabilization was reached at around 60 minutes. According to the fluorescence spectra, the LOD was somewhere between 1000 and 5000 μM with a small decrease in emission intensity.

Figure S257. Kinetic study at different times under visible (up) and UV (down) light in EtOAc. In each photo, left vial was contained the probe and right vial was contained the probe and an excess of TATP.

	**t=0**	**t=90 min**	**t=24 h**
**Visible light**	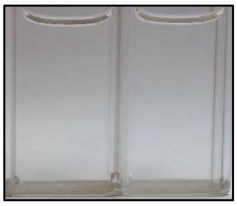	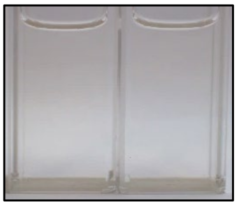	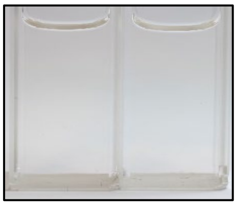
**UV light (366 nm)**	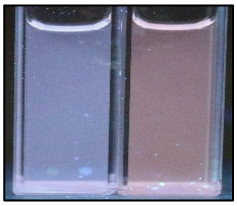	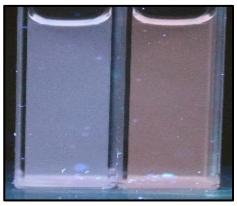	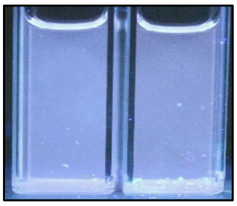

Figure S258. Titration of AR90s with an excess of TATP under visible (up) and UV (down) light in EtOAc.

	**0 250 500 1000 5000 10000 15000 20000 25000 30000 35000**
**Visible light**	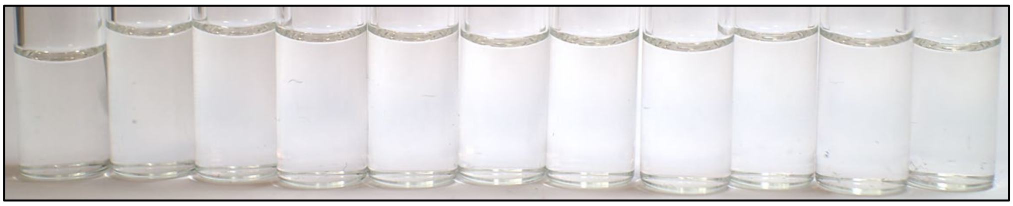
**UV light (366 nm)**	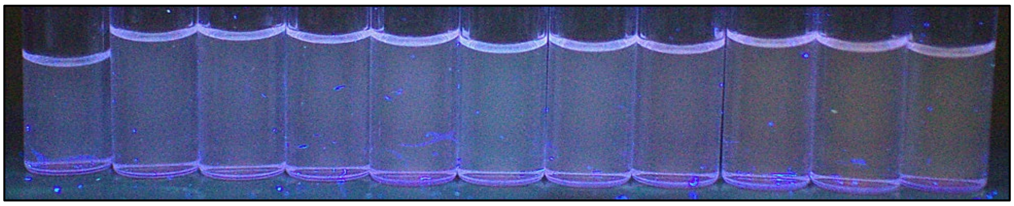

Figure S259. Study of AR90s with TATP in $CHCl_3$:MeOH (9:1). Kinetic study (up left) and profile as function as time at 445 nm and 555 nm simultaneously (up right) in presence of TATP excess. Titration (down left) and fluorescence profile at 425 nm and 560 nm simultaneously (down right) under increasing concentrations of TATP.

$CHCl_3$:MeOH (9:1)

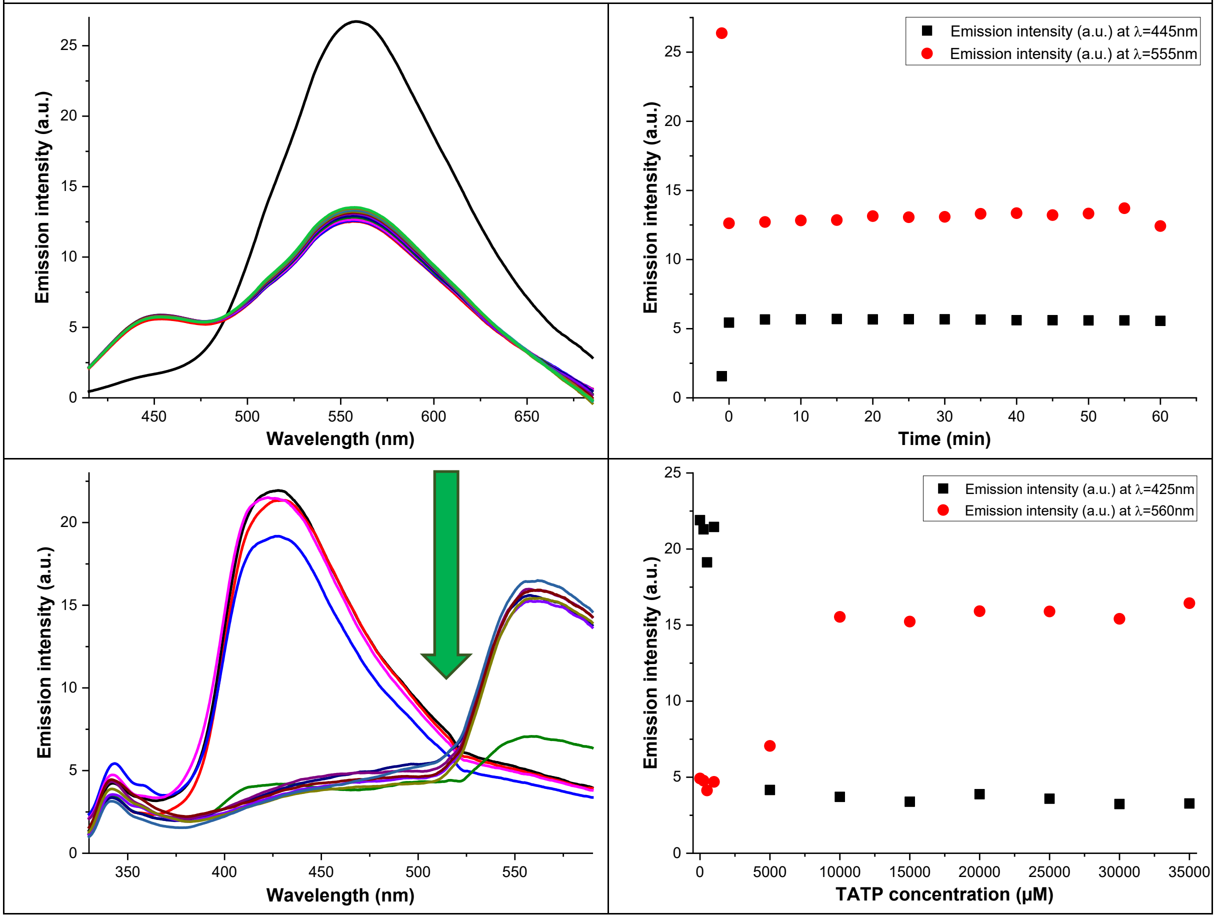

There was a decrease in the emission intensity band immediately after adding TATP but, in the 445 nm band, an increase in the intensity was observed. According to the fluorescence spectra, the LOD of TATP was somewhere between 1000 and 5000 µM with an increase in emission intensity in the 560 nm band stronger than the decrease in the 425 nm band.

Figure S260. Kinetic study at different times under visible (up) and UV (down) light in $CHCl_3$:MeOH (9:1). In each photo, left vial contained probe and right vial contained probe and an excess of TATP.

	**t=0**	**t=60 min**	**t=72 h**
**Visible light**	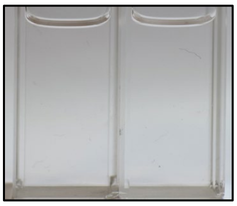	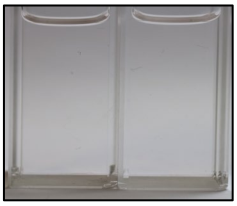	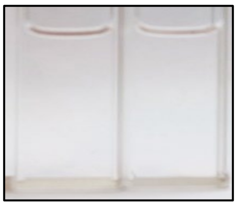
**UV light (366 nm)**	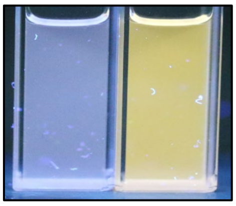	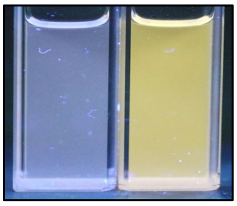	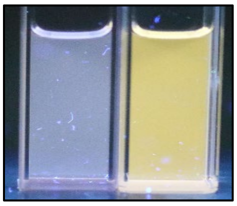

Figure S261. Titration of AR90s with an excess of TATP under visible (up) and UV (down) light in $CHCl_3$:MeOH (9:1).

µM	**0 250 500 1000 5000 10000 15000 20000 25000 30000 35000**
**Visible light**	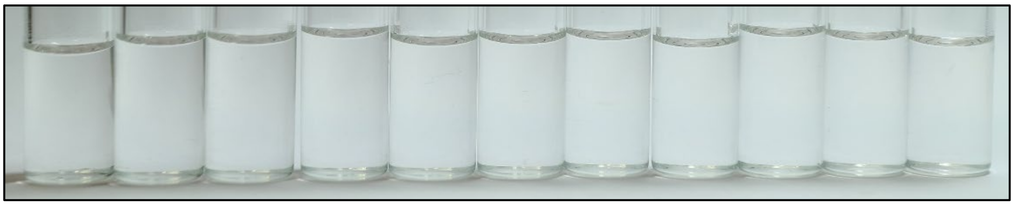
**UV light (366 nm)**	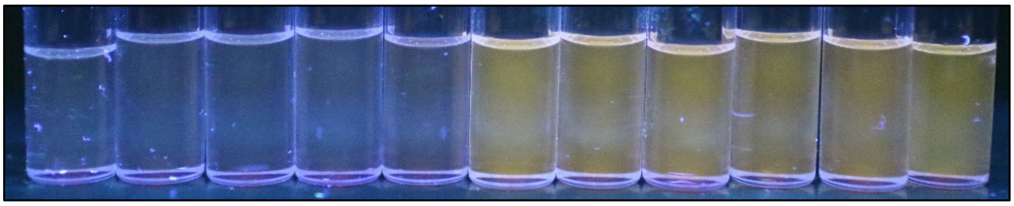

Because of the low sensitivity the study was discontinued.

Synthesis of *N*-(piperidin-4-yl)-4-[(4-piperazin-1-yl)phenyl]naphthalene-1,8-dicarboxylmonoimide (AR90d)

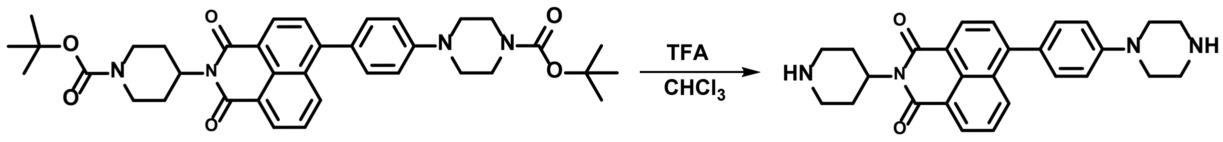

150 mg of *N*-(4-Boc-piperidin-1-yl)-4-[(4-Boc-piperazin-1-yl)phenyl]naphthalene-1,8-dicarboxylmonoimide (AR90p, 0.34 mmol) were dissolved in 15 ml of chloroform and, then, 2 ml of trifluoroacetic acid (26 mmol) was added. The mixture was stirred for 3 hours at room temperature. After that, 10 ml of water were poured into the flask and the mixture was neutralized employing a 40 % sodium hydroxide solution until it reached a neutral pH. Then, 20 ml of water were added into the flask and the mixture was extracted three times with chloroform (3×30 ml). The organic extracts were combined, dried over anhydrous Na_2SO_4 and the solvent was evaporated under reduce pressure, to obtain 148 mg of AR90d as a yellow solid, 99% yield. m.p.: 285 – 288ºC. IR (ATR, cm^{-1}): 3422 (N-H), 2940 – 2827, 1696 (C=O), 1650 (C=O), 1585, 1507, 1396, 1351, 1232, 1154 (C-N), 1115, 912, 777, 755, 583. ^{1}H-NMR (300 MHz, $CDCl_3$) δ (ppm): 8.59 (d, *J* = 7.5 Hz, 2H, 2×H_{Ar}), 8.34 (dd, *J* = 8.5, 1.1 Hz, 1H, H_{Ar}), 7.66 (m, 2H, 2×H_{Ar}), 7.42 (d, *J* = 8.8 Hz, 2H, 2×H_{Ar}), 7.07 (d, *J* = 8.8 Hz, 2H, 2×H_{Ar}), 5.20 (m, 1H, CH), 3.32 – 3.22 (m, 6H, 2×CH_2 + 2×NH), 3.07 – 3.10 (m, 4H, 2×CH_2), 2.81 – 2.68 (m, 4H, 2×CH_2), 1.72 (m, 4H, 2×CH_2). ^{13}C-NMR (75MHz, $CDCl_3$) δ (ppm): 164.9 (C=O), 164.7 (C=O), 151.9 (C_{Ar}), 146.9 (C_{Ar}), 132.8 (C_{Ar}H), 131.2 (C_{Ar}H), 131.1 (C_{Ar}H), 131.0 (C_{Ar}H), 130.1 (C_{Ar}), 129.5 (C_{Ar}), 129.0 (C_{Ar}), 127.7 (C_{Ar}H), 126.7 (C_{Ar}H), 123.4 (C_{Ar}), 121.5 (C_{Ar}), 115.6 (C_{Ar}H), 52.1 (CH), 49.9 (CH_2), 47.1 (CH_2), 46.2 (CH_2), 30.3 (CH_2). UV-Vis ($CHCl_3$), λ_{max} nm (log ε): 390 (3.6). HRMS (MALDI, DIT-) m/z: calculated for $C_{27}H_{28}N_4O_2$+H: 441.2285 (M^++H); found 441.2306. Ø (Toluene, %): 96.12. τ (375 nm, Toluene, ns): 5.278 (χ^2: 1.025).

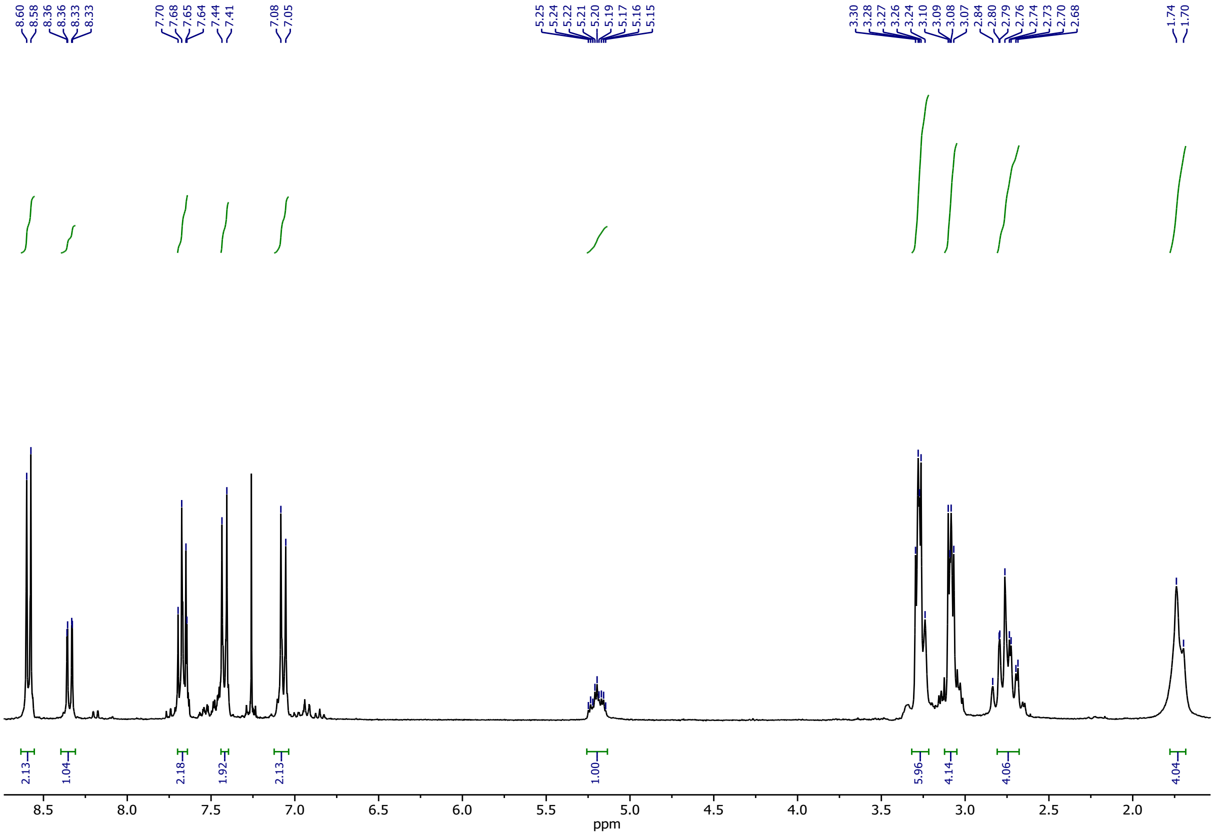

Figure S262. ^{1}H-NMR (CDCl$_3$, 300 MHz) of AR90d.

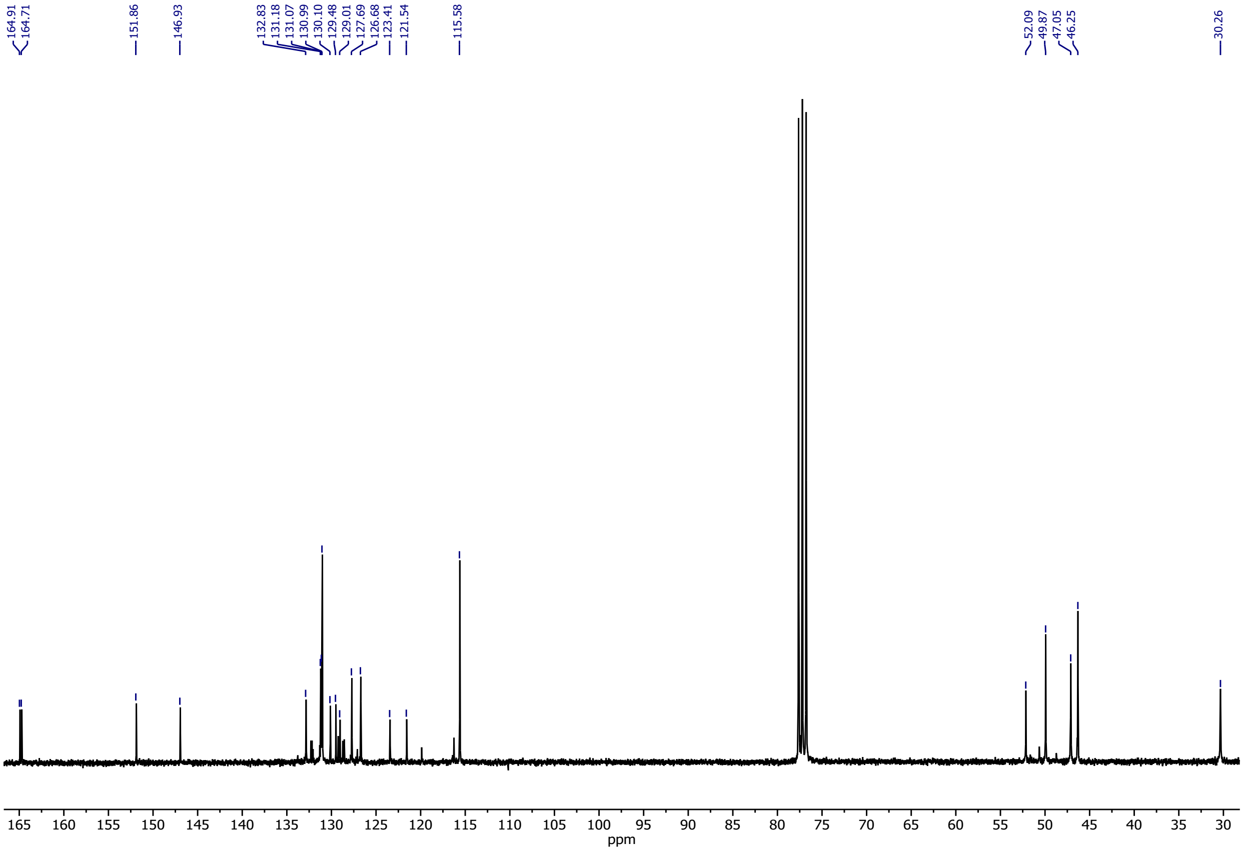

Figure S263. ^{13}C-NMR (CDCl$_3$, 75 MHz) of AR90d.

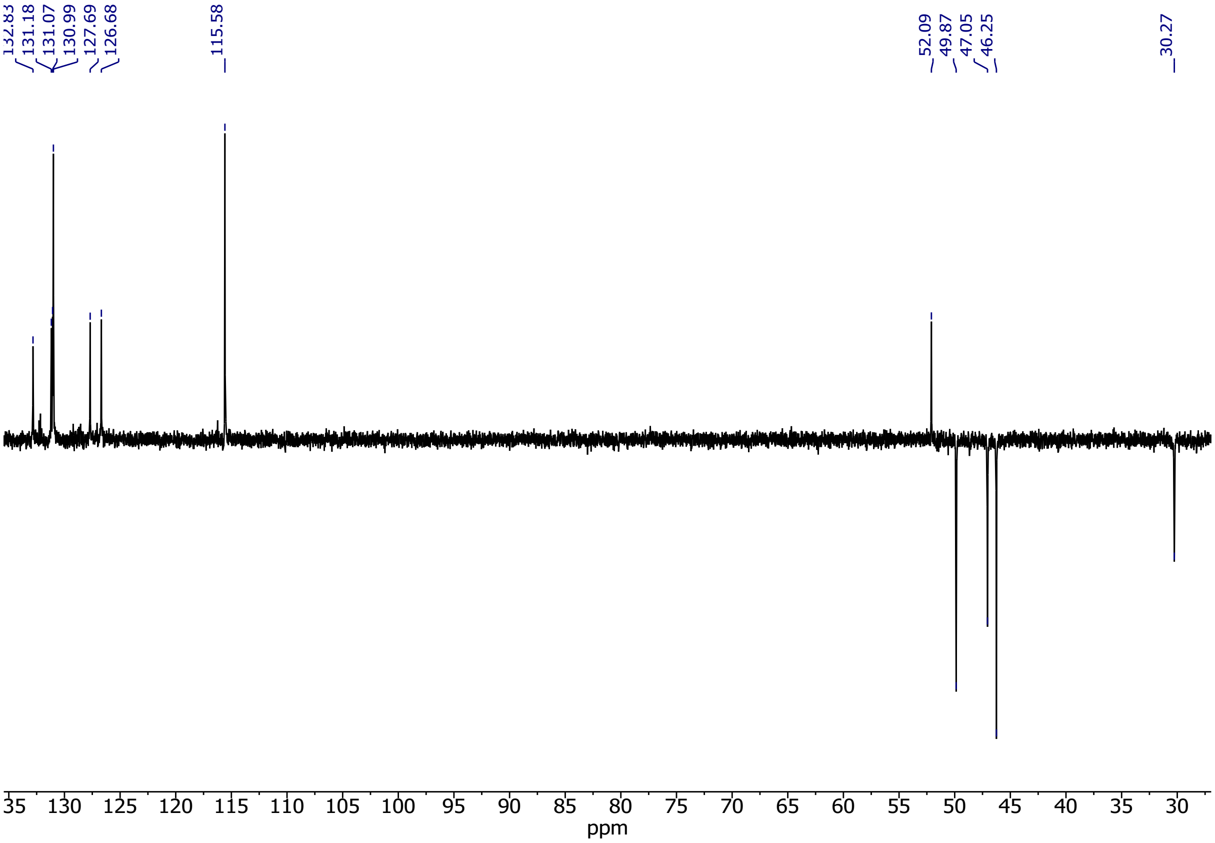

Figure S264. DEPT NMR ($CDCl_3$, 75 MHz) of AR90d.

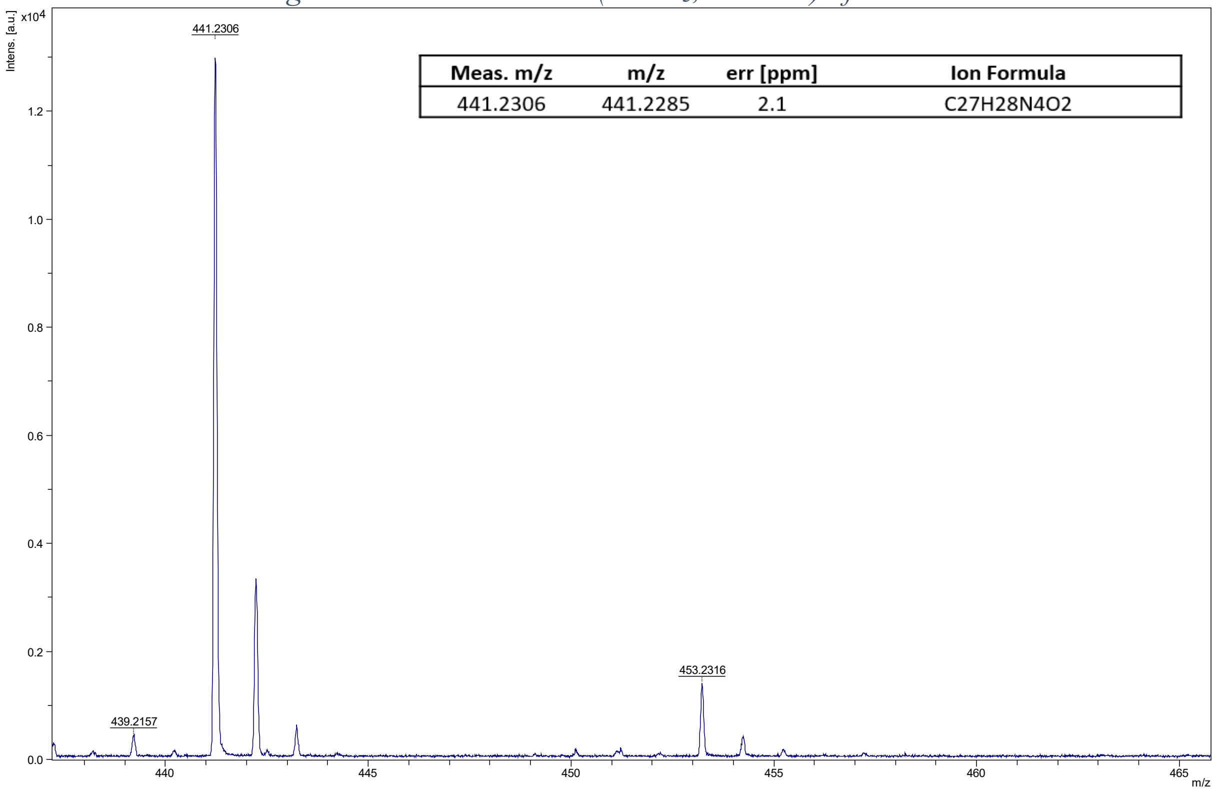

Figure S265. HRMS (MALDI, DIT+) of AR90d.

Fluorescence decay lifetime (τ):

The solvent used was toluene. The concentration of the compound was 20 µM.

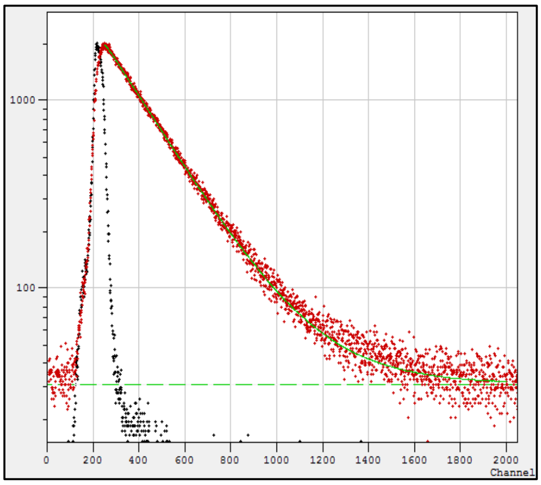

Figure S266. Fluorescence decay lifetime of AR90d.

Solvatochromism:

The concentration of the compound was 20 µM and the excitation wavelength was 385 nm.

Figure S267. Solvatochromism of AR90d.

<table>
<tr><th>Absorbance</th><th>Normalized absorbance</th></tr>
<tr><td>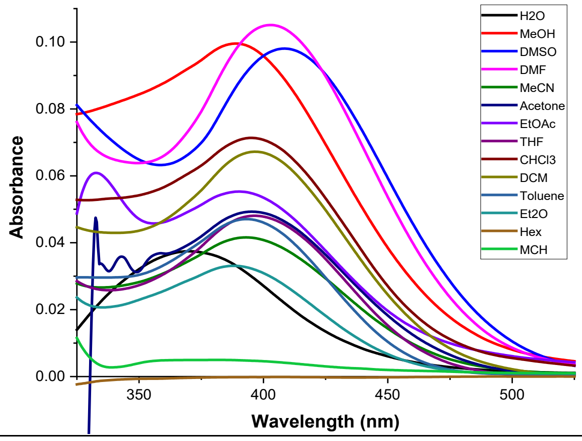
</td><td>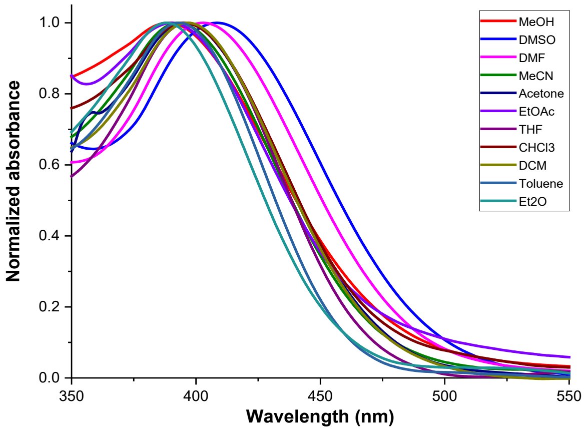
</td></tr>
<tr><th>Emission</th><th>Normalized emission</th></tr>
</table>

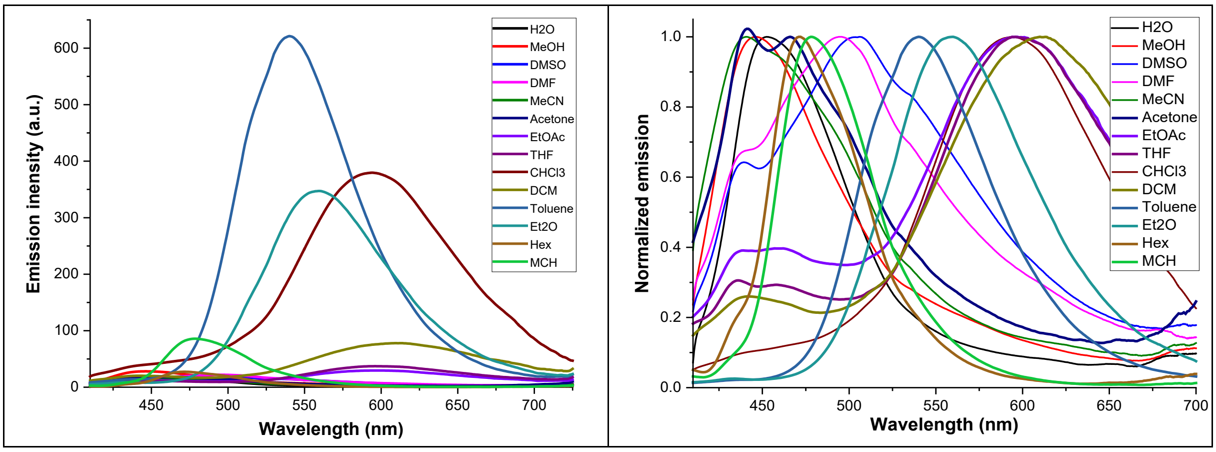

Figure S268. Solvatochromism of AR90d under visible and 366 nm light

	H_2O MeOH DMSO DMF MeCN Acet. EtOAc THF $CHCl_3$ DCM Tol. Et_2O Hex MCH
**Visible light**	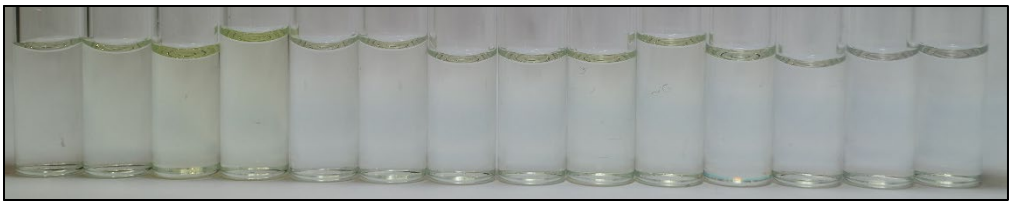
**UV light (366 nm)**	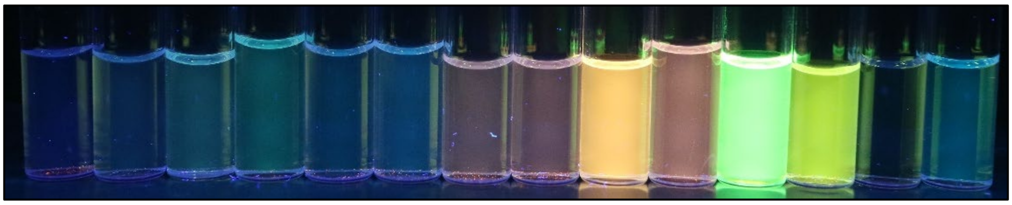

Water acceptance test:

The solvent used was MeOH and the concentration of the compound was 20 µM.

Figure S269. Water acceptance test of AR90d under visible (up) and UV (down) light.

	R 5% 10% 20% 30% 40% 50% 60% 70% 80% 90%
**Visible light**	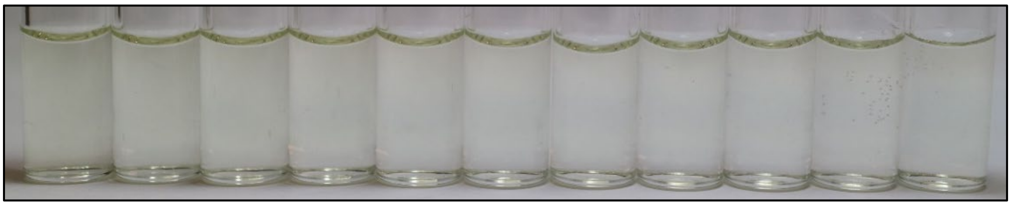
**UV light (366 nm)**	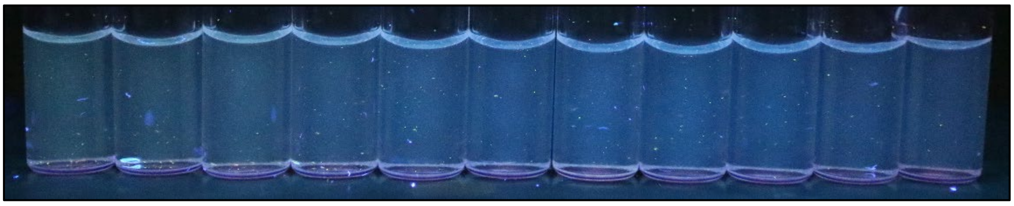

There was no increase in emission intensity.

pH test:

The amount of water for the performance of this test was 20%. The concentration of the compound was 20 µM and the solvent used was MeOH.

Figure S270. pH test of AR90d under visible (up) and UV (down) light.

	R HCl 3.4 4.8 6.4 7.4 7.9 10.5 KOH
**Visible light**	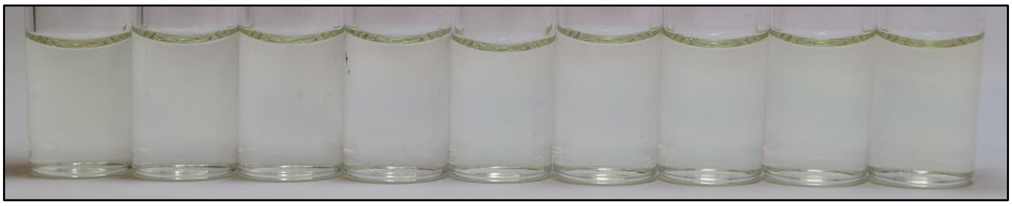
**UV light (366 nm)**	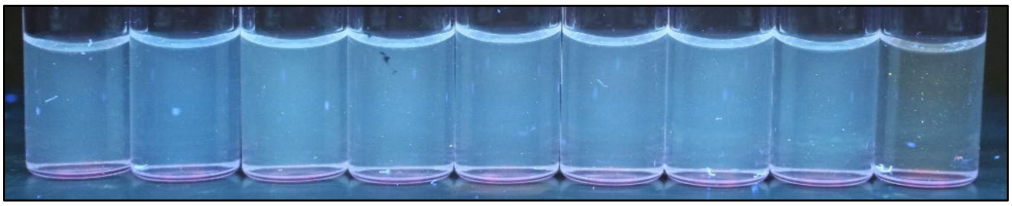

The most important change was the fluorescence quenching at very basic pH.

Cations and anions test:

The concentration of the compound was 20 µM and the solvent used was MeOH.

Figure S271. Cations test of AR90d under visible (up) and UV (down) light.

	R Ag^{+} Ni^{2+} Sn^{2+} Cd^{2+} Zn^{2+} Pb^{2+} Cu^{2+} Fe^{3+} Sc^{3+} Al^{3+} Hg^{2+} Au^{3+} Co^{2+} Pd^{2+} Ir^{3+} Cu^{+} Ru^{3+} Pt^{2+}
**Visible light**	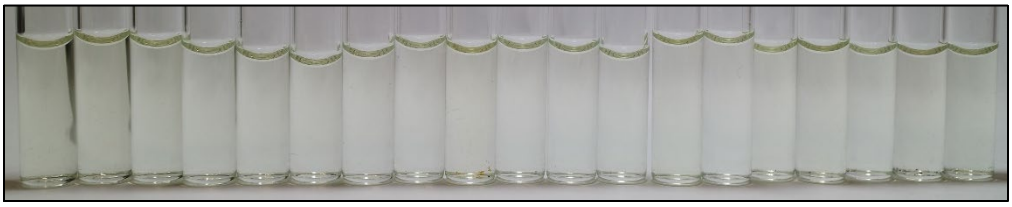
**UV light (366 nm)**	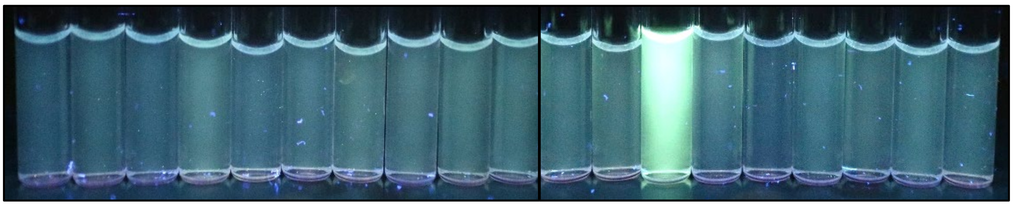

The most significant change was the green color acquired by the fluorescence when was confronted with Au^{3+}.

The concentration of the compound was 20 µM and the solvent used was MeOH.

Figure S272. Anions test of AR90d under visible (up) and UV (down) light.

	R F^{-} Cl^{-} Br^{-} I^{-} BzO^{-} NO_3^{-} $H_2PO_4^{-}$ HSO_4^{-} AcO^{-} CN^{-} SCN^{-}
**Visible light**	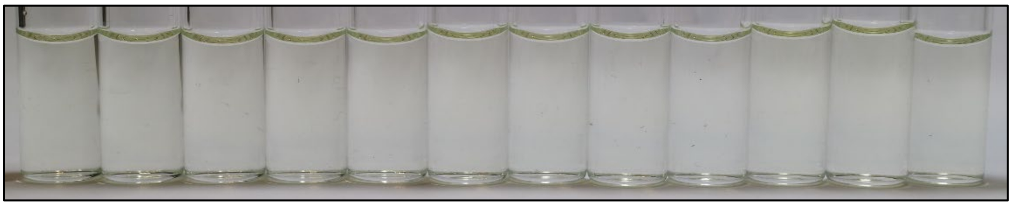

**UV light (366 nm)**	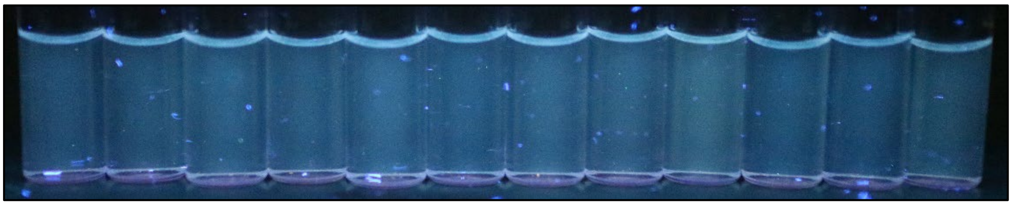

No significant changes were observed.

Oxidants and reductants test:
The solvent used was THF and concentration of the compound was 20 μM.

Figure S273. Oxidants and reductants test of AR90d under visible (up) and UV (down) light.

	R HCl* HNO_3 Oxn Hz H_2O_2 R m-CPBA TNB TNT R m-CPBA TATP HMTD
**Visible light**	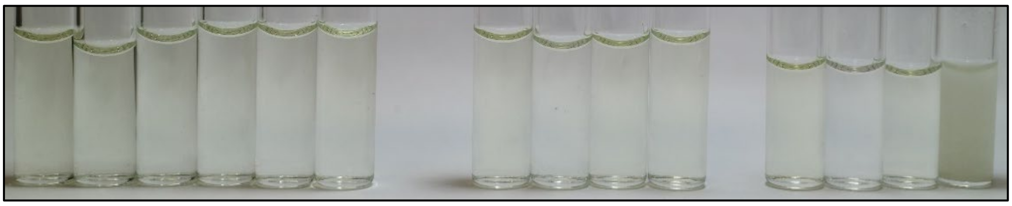
**UV light (366 nm)**	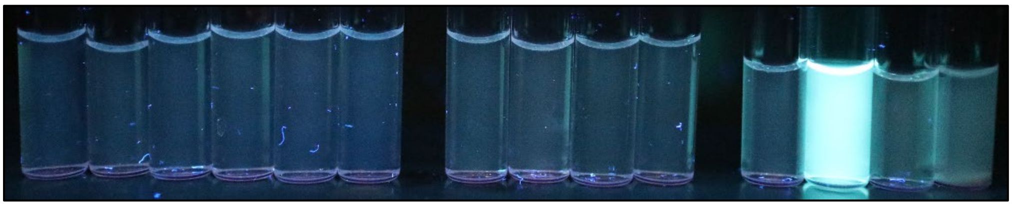

*HCl is included in all experiments in a close position to HNO_3 to distinguish between the redox action of HNO_3 and a possible effect due only to the concomitant acidity of nitric acid. No significant changes were observed except for an increase in fluorescence with m-CPBA.

Preliminary solvents test:
In order to determine the fluorescence variation of the compound under study in the presence of TATP, some tests were carried out for the probe in 9 different solvents. The AR90d concentration was 20 μM and the TATP was added in excess (7 mg in each vial). All the tests were performed at room temperature and the photographs were taken immediately after the addition of TATP and again after 72 hours.

Figure S274. Pairs of AR90d (left) and AR90d with excess of TATP (right) in different solvents at time 0 h (up) and 72 h (down).

	DMF MeCN EtOAc THF $CHCl_3$ DCM Tol $CHCl_3$:MeOH DCM:MeOH
**t = 0 h**	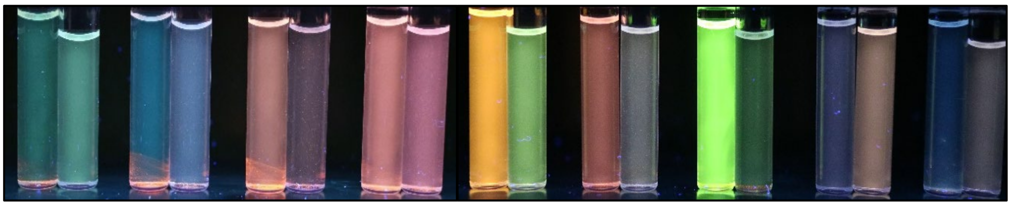
**t = 72 h**	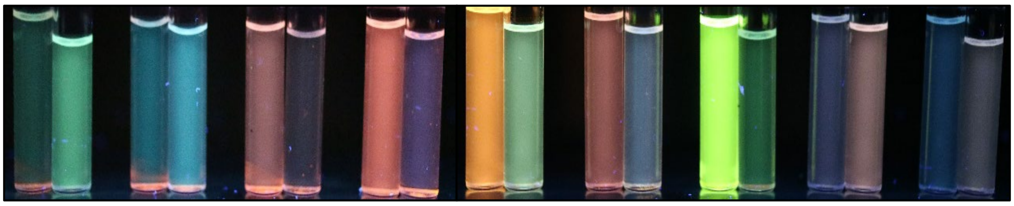

The biggest fluorescence changes were seen in $CHCl_3$, toluene and $CHCl_3$:MeOH (9:1) so these were the chosen solvents to perform the kinetic studies and TATP titrations experiments.

Solvents test with TATP:

For all solvents, the working concentration of AR90d was 2.5 µM, the excitation wavelength was 385 nm and the temperature for all test was 25ºC.

In the kinetic studies, TATP was in excess (20 mM) and the fluorescence emission measurements were made during the subsequent minutes at 5 min, 10 min and 30 min time intervals. In the titration, the TATP concentration was between 0 and 35000 µM and it was added directly as solid. The measurements were carried out immediately after the addition of the probe.

Figure S275. Study of AR90d with TATP in $CHCl_3$. Kinetic study (up left) and profile as function as time at 555 nm (up right) in presence of TATP excess. Titration (down left) and fluorescence profile at 550 nm and 600 nm simultaneusly (down right) under increasing concentrations of TATP.

$CHCl_3$

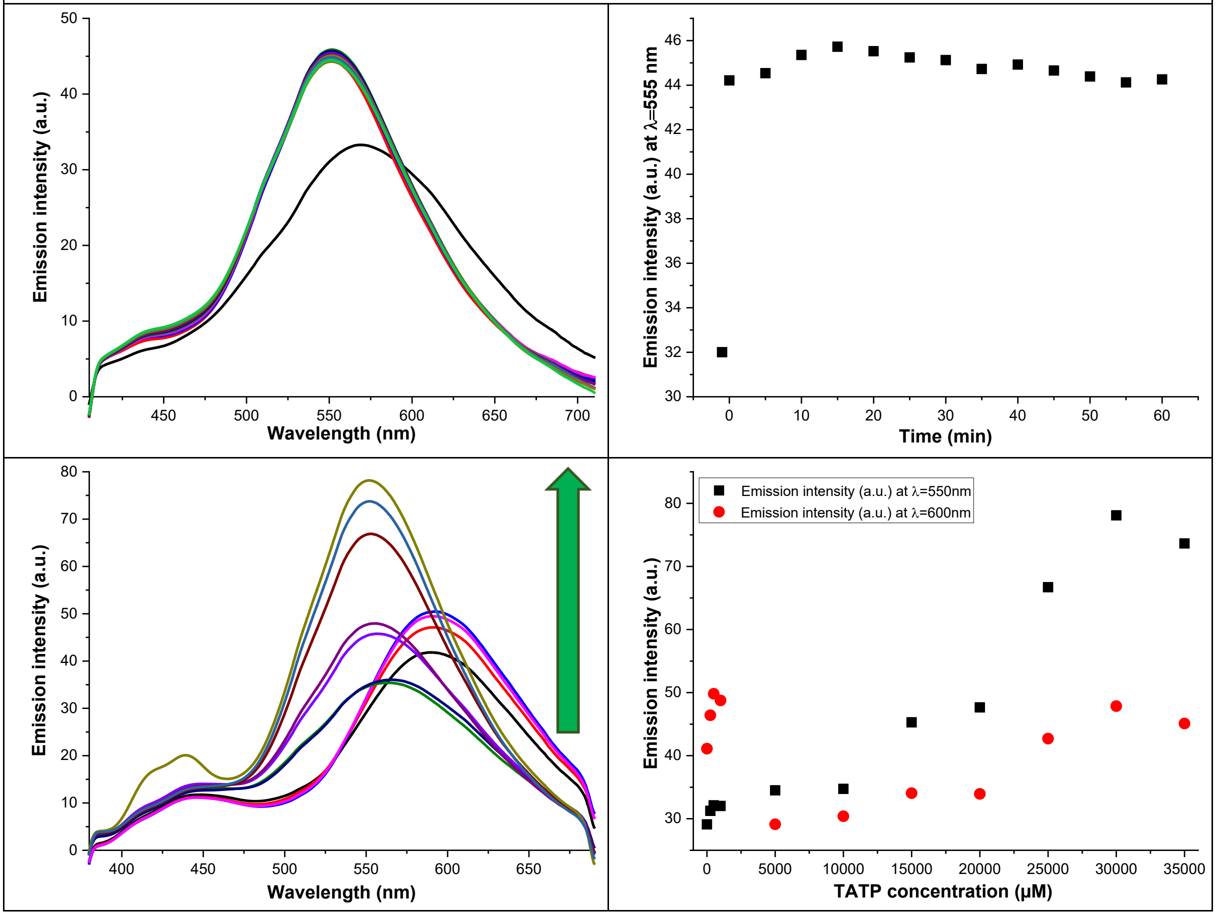

There was a change in the emission intensity band immediately after adding TATP. According to the fluorescence spectra, the LOD was somewhere between 1000 and 5000 µM.

Figure S276. Kinetic study at different times under visible (up) and UV (down) light in $CHCl_3$. In each photo, left vial was contained the probe and right vial was contained the probe and an excess of TATP.

	**t=0**	**t=60 min**	**t=24 h**
**Visible light**	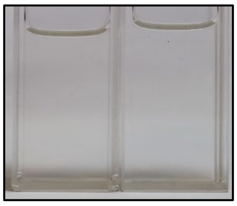	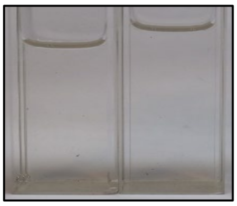	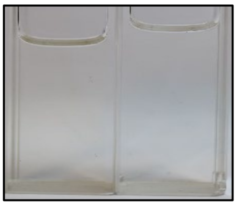
**UV light (366 nm)**	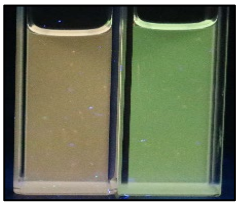	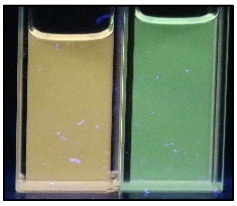	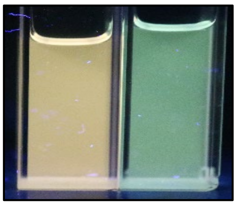

Figure S277. Titration of AR90d with an excess of TATP under visible (up) and UV (down) light in $CHCl_3$.

μM	**0 250 500 1000 5000 10000 15000 20000 25000 30000 35000**
**Visible light**	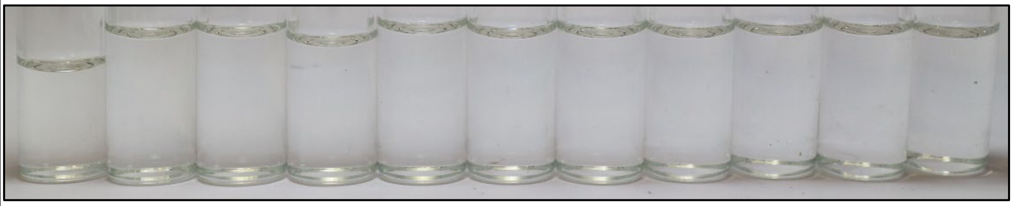
**UV light (366 nm)**	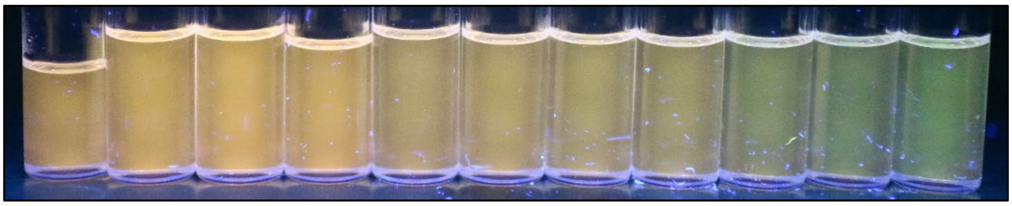

Figure S278. Study of AR90d with TATP in toluene. Kinetic study (up left) and profile as function as time at 555 nm (up right) in presence of TATP excess. Titration (down left) and fluorescence profile at 535 nm (down right) under increasing concentrations of TATP.

Toluene

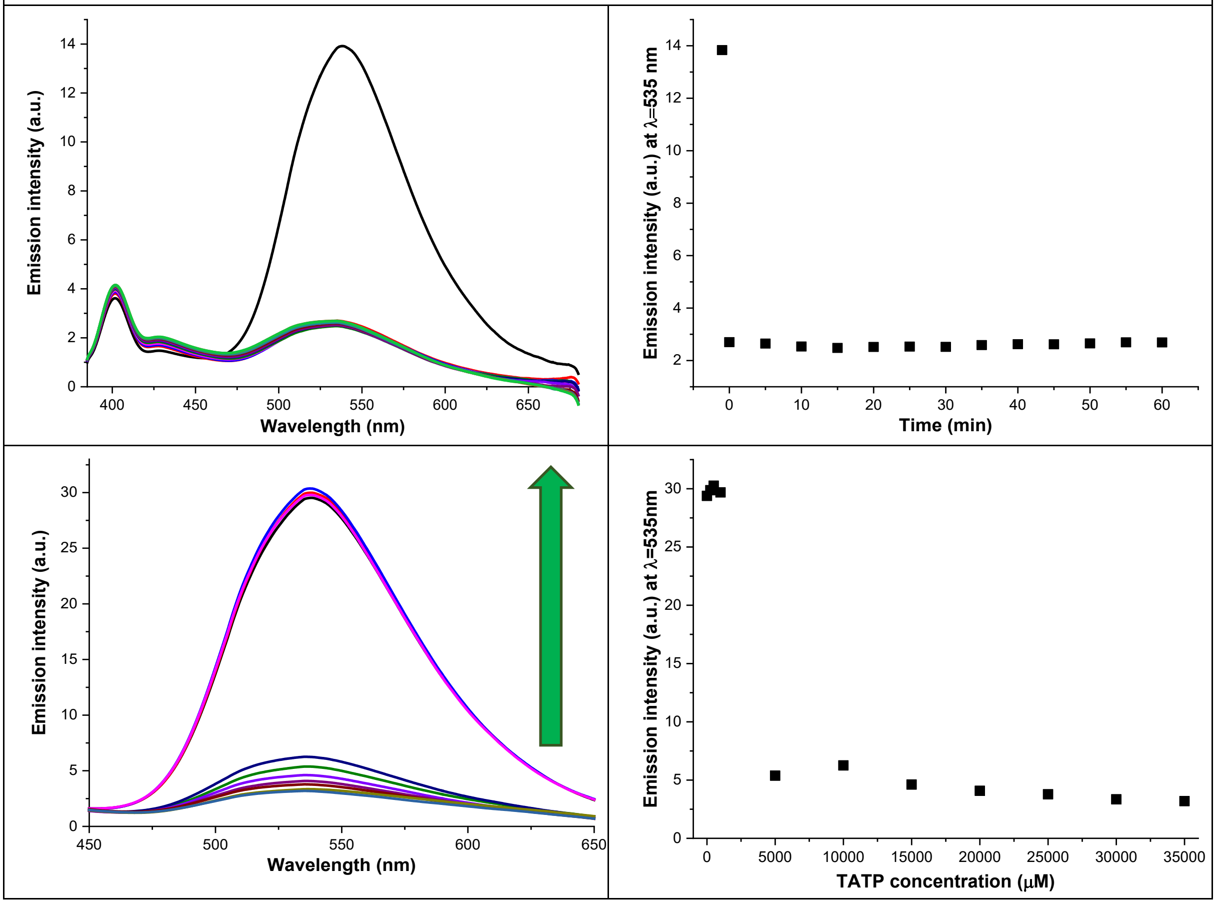

There was a marked decrease change in the emission intensity band immediately after adding TATP. According to the fluorescence spectra, the LOD was somewhere between 1000 and 5000 μM with a decrease of the intensity.

Figure S279. Kinetic study at different times under visible (up) and UV (down) light in toluene. In each photo, left vial was contained the probe and right vial was contained the probe and an excess of TATP.

	t=0	t=60 min	t=24 h
**Visible light**	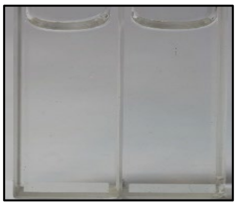	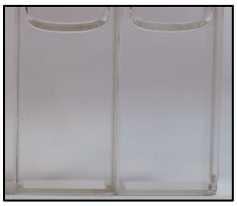	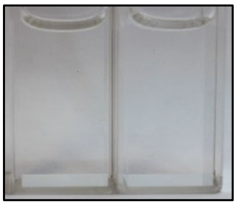
**UV light (366 nm)**	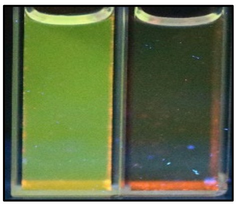	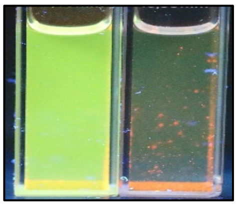	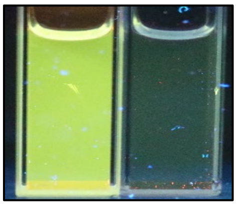

Figure S280. Titration of AR90d with an excess of TATP under visible (up) and UV (down) light in toluene.

µM	0 250 500 1000 5000 10000 15000 20000 25000 30000 35000
**Visible light**	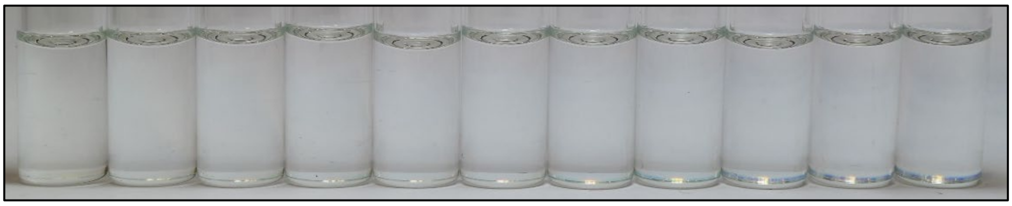
**UV light (366 nm)**	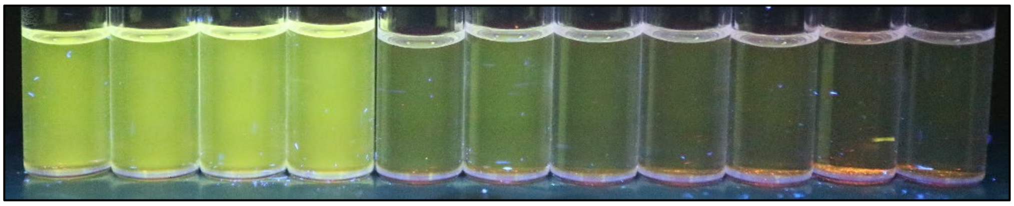

Figure S281. Study of AR90d with TATP in $CHCl_3$:MeOH (9:1). Kinetic study (up left) and profile as function as time at 500 nm and 585 nm simultaneusly (up right) in presence of TATP excess. Titration (down left) and fluorescence profile at 500 nm and 575 nm simultaneously (down right) under increasing concentrations of TATP.

$CHCl_3$:MeOH (9:1)

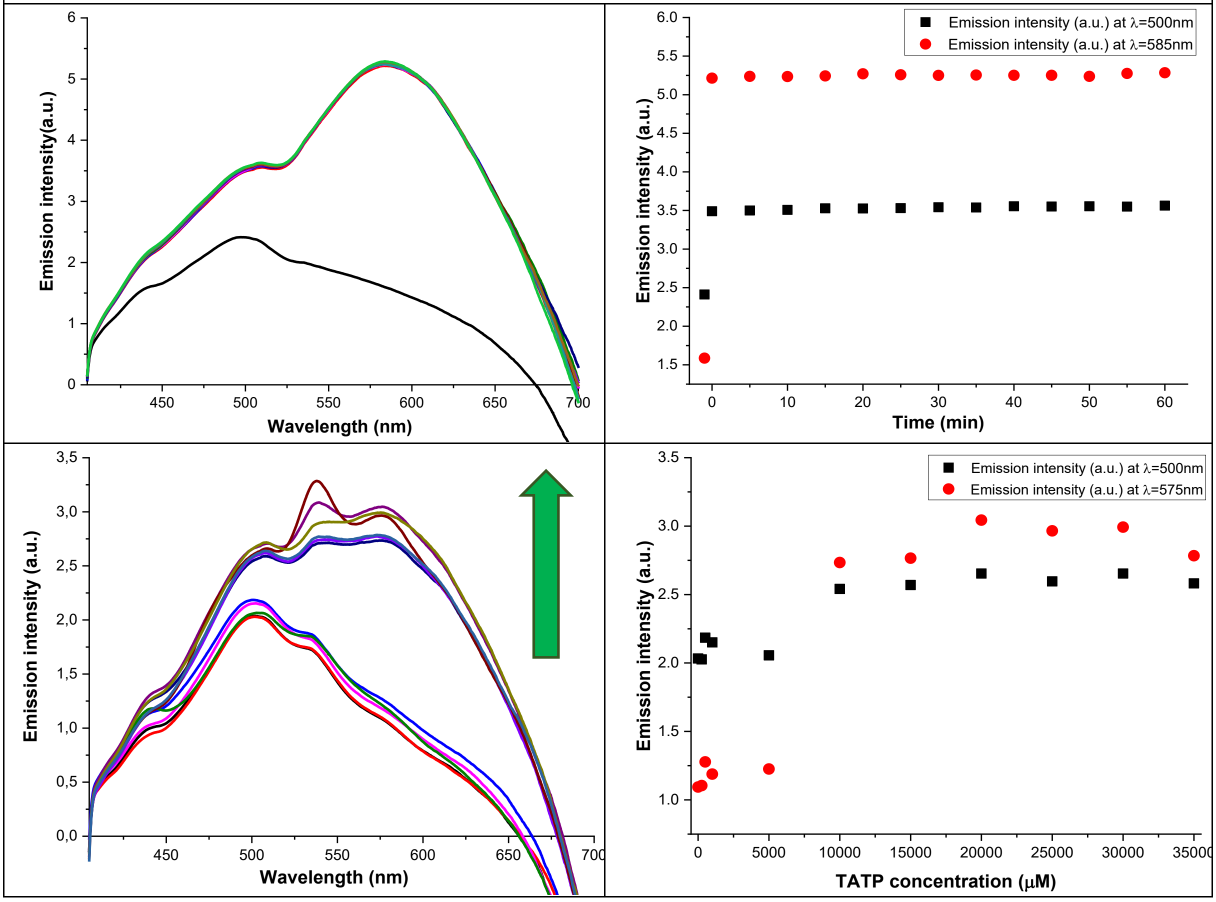

There was an increase in the emission intensity band immediately after adding TATP but emission intensity was remained stable. According to the fluorescence spectra, the LOD was somewhere between 1000 and 5000 µM with an increase of the intensity.

Figure S282. Kinetic study at different times under visible (up) and UV (down) light in $CHCl_3$:MeOH (9:1). In each photo, left vial was contained the probe and right vial was contained the probe and an excess of TATP.

	**t=0**	**t=60 min**	**t=24 h**
**Visible light**	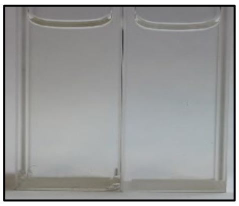	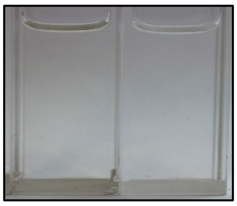	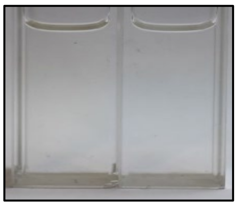
**UV light (366 nm)**	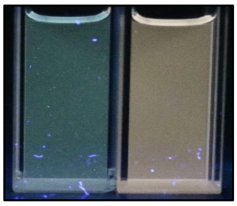	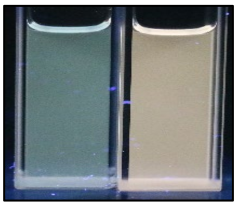	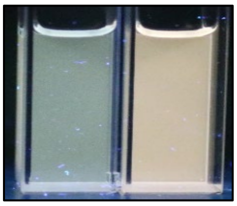

Figure S283. Titration of AR90d with an excess of TATP under visible (up) and UV (down) light in $CHCl_3$:MeOH (9:1).

	**0 250 500 1000 5000 10000 15000 20000 25000 30000 35000**
**Visible light**	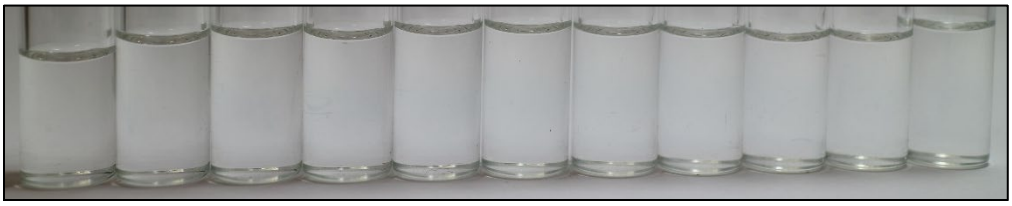
**UV light (366 nm)**	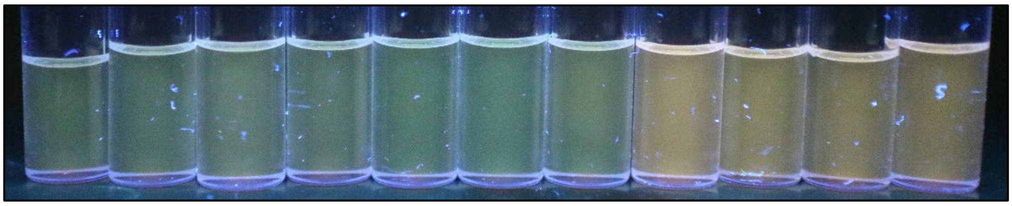

Because of the low sensitivity the study was discontinued.
