# Supplementary material for: Silica Nanoparticle/Fluorescent Dye Assembly Capable of Ultrasensitively Detecting Airborne Triacetone Triperoxide: Proof-of-Concept Detection of Improvised Explosive Devices in the Workroom
Source: ACS Appl Mater Interfaces. 2023 Jun 21;15(26):32024–36. doi: 10.1021/acsami.3c05931 (PMC10326811; doi:10.1021/acsami.3c05931)
Supplement: Supplementary file 4 — am3c05931_si_004.pdf [file am3c05931_si_004.pdf]

Supporting Information 2

Silica Nanoparticle/Fluorescent Dye Assembly Capable of Ultrasensitively Detecting Airborne Triacetone Triperoxide: Proof-of-Concept Detection of Improvised Explosive Devices in the Workroom.

*Andrea Revilla-Cuesta,[a] Irene Abajo-Cuadrado,[a] María Medrano,[a] Mateo M. Salgado,[a] Manuel Avella,[a] María Teresa Rodríguez,[a] José García-Calvo,[#a] and Tomás Torroba[a]**

[a]Department of Chemistry, Faculty of Science, University of Burgos, 09001 Burgos, Spain.

[b]Electron Microscopy Lab, IMDEA Materials Institute, Eric Kandel, 2, Tecnogetafe, 28906 Getafe (Madrid), Spain.

*E-mail: ttorroba@ubu.es

Present Address: [#]J.G.C.: IMDEA Nanociencia Institute, Faraday 9, 28049 Madrid, Spain.

Validation of the app: Once you access the application with a username and password, the Analysis option is selected. The application asks to select the photograph to analyze; which can come from the photo gallery or be taken at that very moment. Image is properly centered inside the box and the exact point to analyze is selected. Then, the Analyze button located in the lower area of the screen is pressed. After this, the application offers a result, either negative or positive for the presence of TATP. Below, 8 examples are shown (6 of them with a positive detection result coming from the 6 different experiments and also different distances to the TATP source and 2 of them with a negative result).

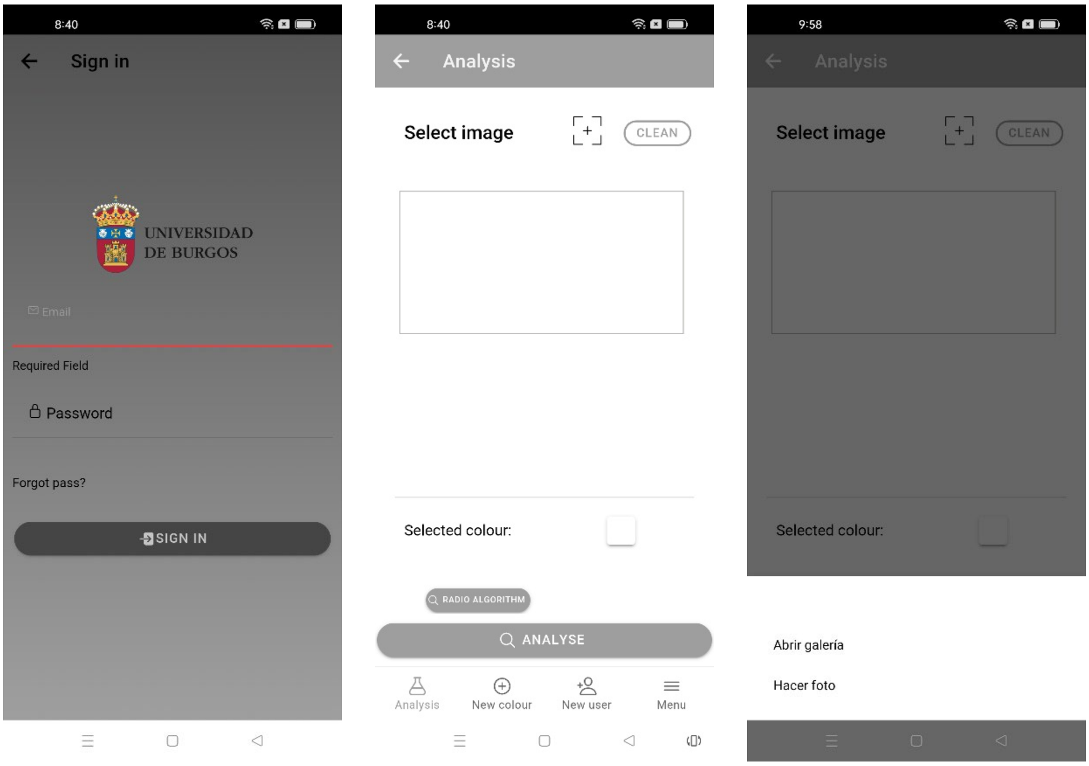

Figure S(2)1. The app. initial screens.

- **Case 1: positive result (photograph from Experiment 7, 25 cm to the TATP source after exposure to TATP vapors).**

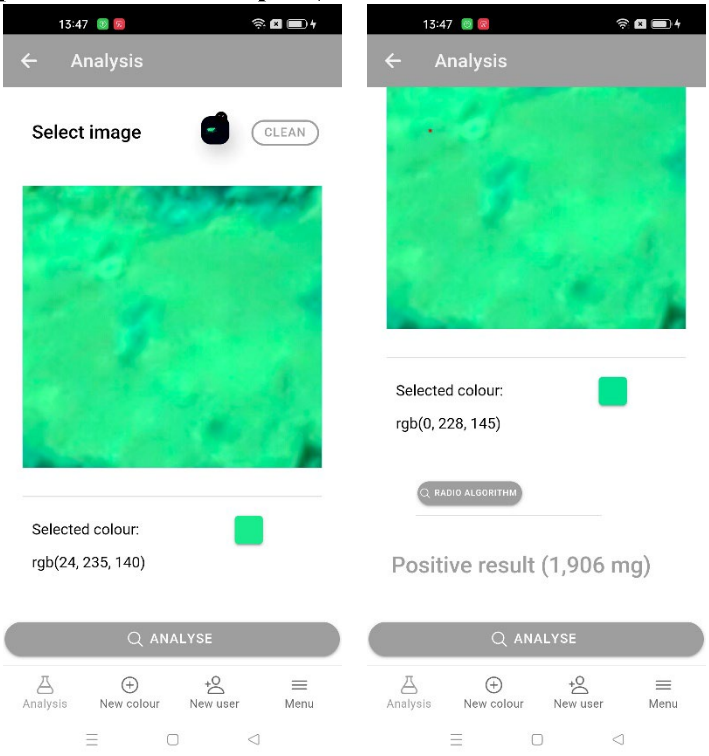

Figure S(2)2. Case 1: positive result.

- **Case 2: positive result (photograph from Experiment 8, 10 cm to the TATP source after exposure to TATP vapors).**

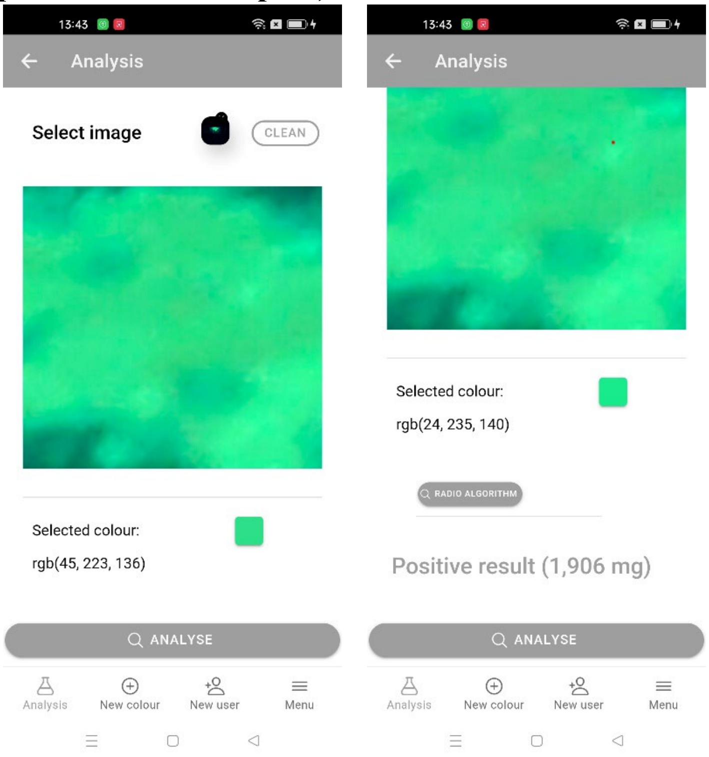

Figure S(2)3. Case 2: positive result.

- **Case 3: positive result (photograph from Experiment 9, 100 cm to the TATP source after exposure to TATP vapors).**

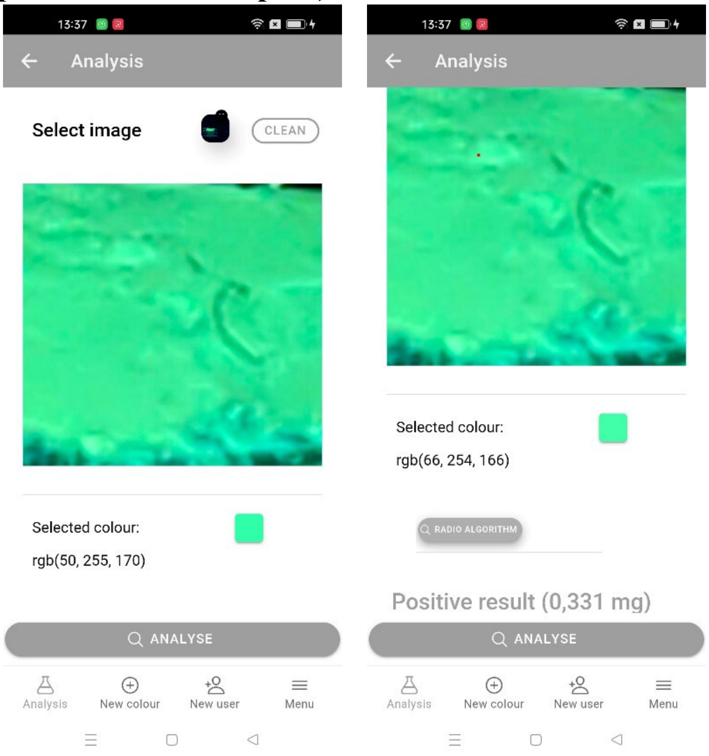

Figure S(2)4. Case 3: positive result.

- **Case 4: positive result (photograph from Experiment 10, 50 cm to the TATP source after exposure to TATP vapors).**

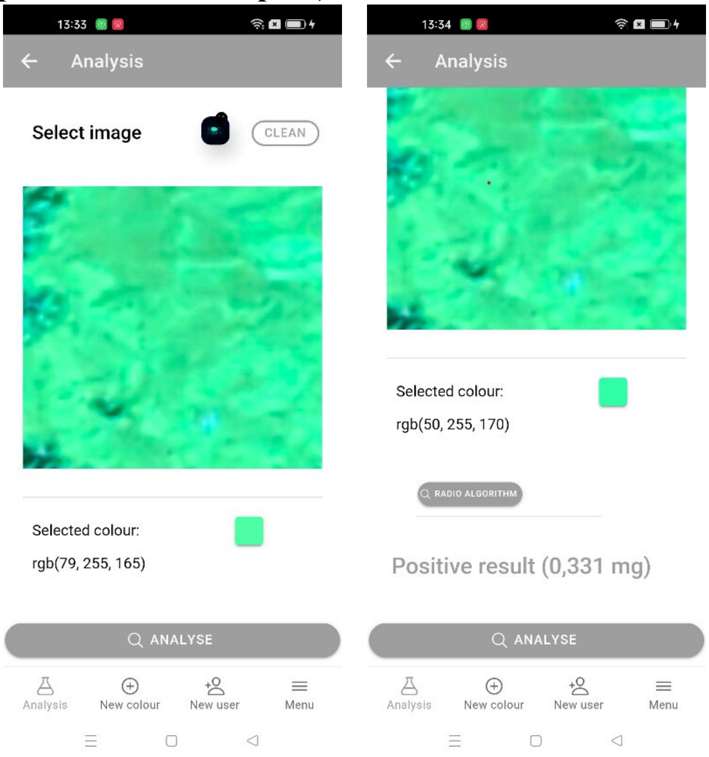

Figure S(2)5. Case 4: positive result.

- **Case 5: positive result (photograph from Experiment 11, 200 cm to the TATP source after exposure to TATP vapors).**

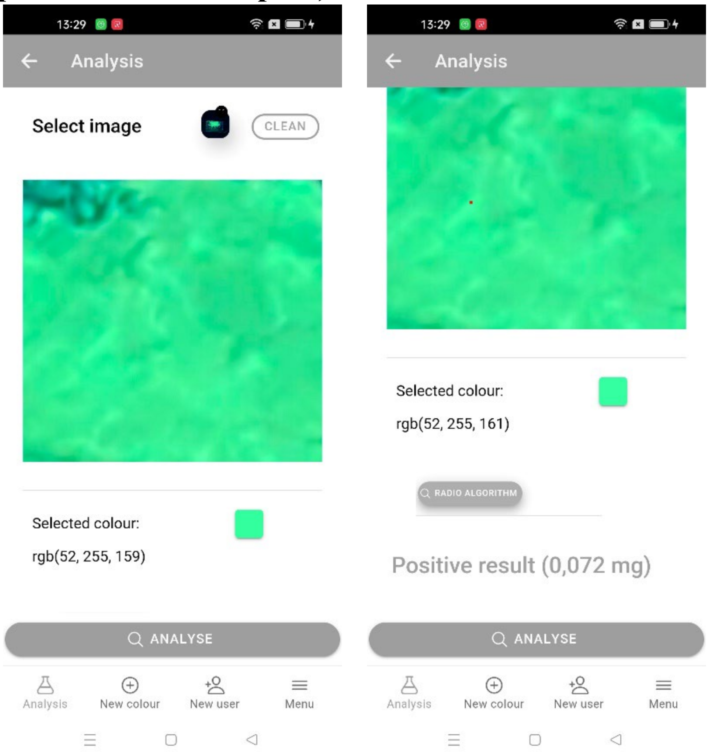

Figure S(2)6. Case 5: positive result.

- **Case 6: positive result (photograph from Experiment 12, 150 cm to the TATP source after exposure to TATP vapors).**

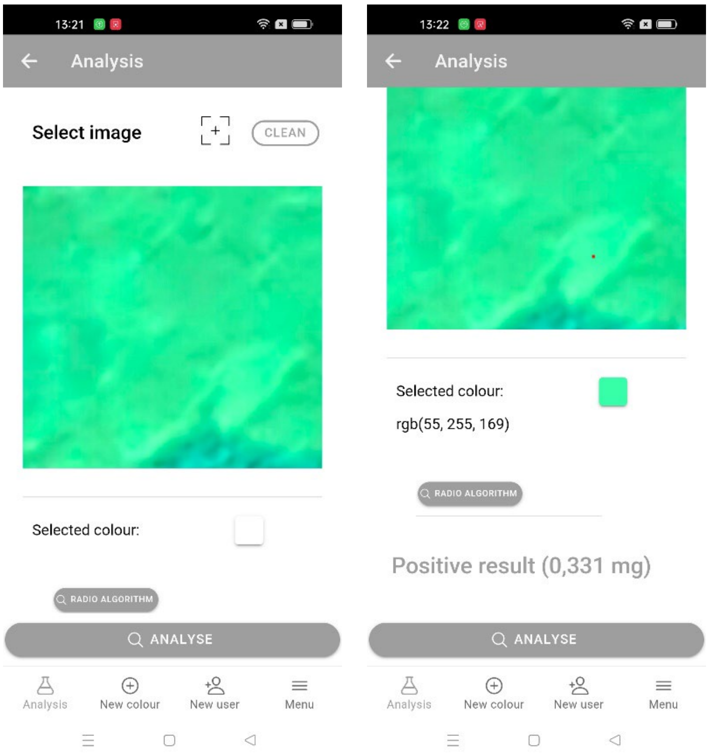

Figure S(2)7. Case 6: positive result.

- **Case 7: negative result (photograph from Experiment 7, 10 cm to the TATP source before exposure to TATP vapors).**

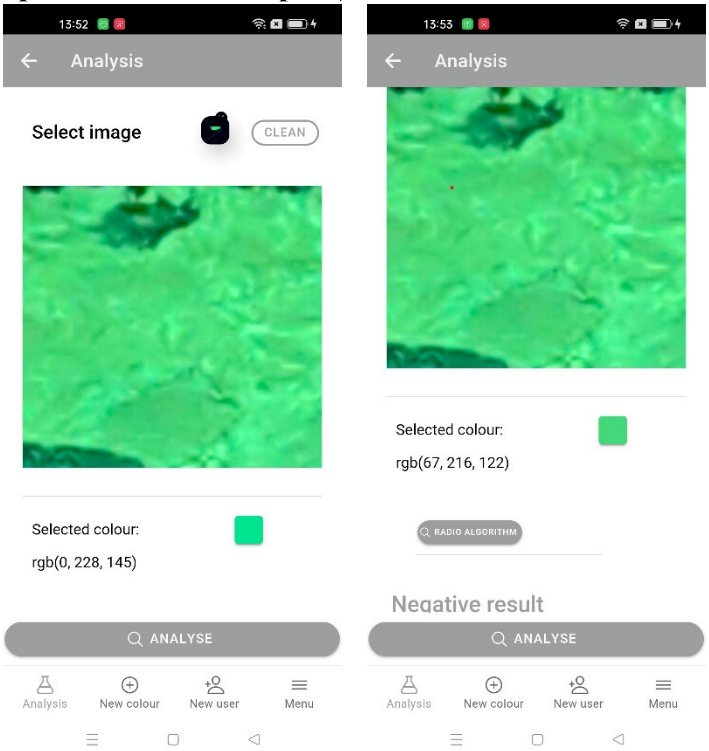

Figure S(2)8. Case 7: negative result.

- **Case 8: negative result (photograph from Experiment 10, 100 cm to the TATP source before exposure to TATP vapors).**

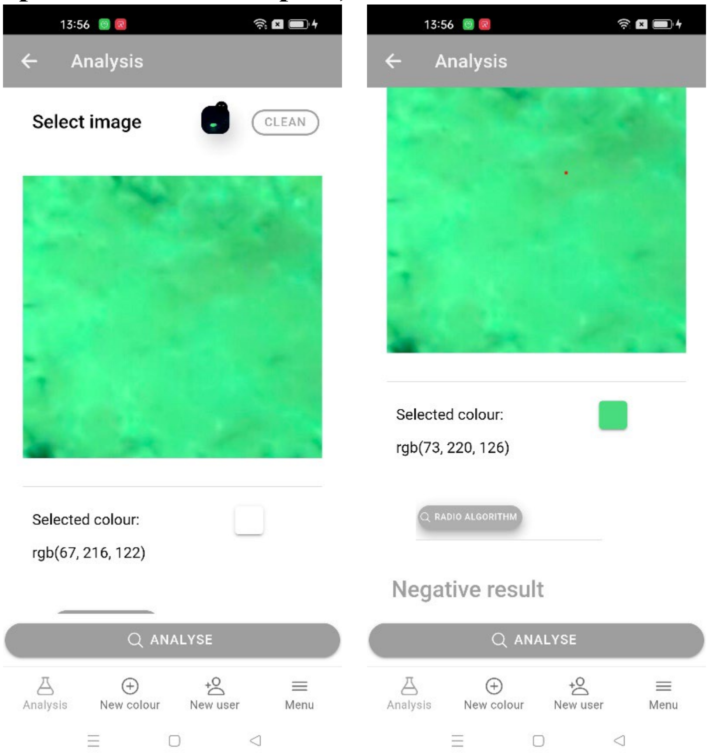

Figure S(2)9. Case 8:negative result.
